# The *pos-1* 3′ untranslated region governs germline specification and proliferation to ensure reproductive robustness

**DOI:** 10.1371/journal.pgen.1012129

**Published:** 2026-04-27

**Authors:** Haik V. Varderesian, Juliet N. Utaegbulam, Hannah E. Brown, Beverly Ramirez, Melina Velcani, Sean P. Ryder

**Affiliations:** Department of Biochemistry and Molecular Biotechnology, University of Massachusetts Chan Medical School, Worcester, Massachusetts, United States of America; University of California San Diego, UNITED STATES OF AMERICA

## Abstract

During fertilization, haploid gametes combine to form a zygote. The male (sperm) and female (oocyte) gametes contribute a similar amount of DNA, but the oocyte contributes nearly all the cytoplasm. Oocytes are loaded with maternal mRNAs thought to be essential for embryonic patterning after fertilization. A conserved suite of RNA-binding proteins (RBPs) regulates the spatiotemporal translation and stability of maternal mRNAs. POS-1 is a CCCH-type tandem zinc finger RBP expressed in fertilized *Caenorhabditis elegans* zygotes from maternally supplied mRNA. POS-1 accumulates in the posterior of the embryo where it promotes posterior cell fate. Here, we show that the *pos-1* 3′ untranslated region (UTR) is essential for POS-1 patterning and contributes to maximal reproductive fecundity. We engineered a *pos-1* mutant where most of the endogenous *pos-1* 3′UTR was removed using CRISPR genome editing. Our results show that the 3′UTR represses POS-1 expression in the maternal germline but increases POS-1 protein levels in embryos after fertilization. In a wild-type background, POS-1 repression via the 3′UTR has little impact on fertility. In a sensitized background, the deletion mutant has a complex pleiotropic phenotype where most adult homozygous progeny lack either one or both gonad arms. Most phenotypes become more penetrant at elevated temperature. Together, our results support an emerging model where the 3′UTRs of maternal transcripts, rather than being essential, contribute to reproductive robustness during stress.

## Author summary

According to the World Health Organization, infertility affects one out of six people worldwide. Infertility problems arise from defects in sperm or egg production, mutations in the sperm or egg DNA that impact embryo viability, and a variety of other causes. It is estimated that 15% of pregnancies end with miscarriage, defined as spontaneous loss of pregnancy prior to 24 weeks of gestation. Environmental factors such as smoking, excessive alcohol consumption, and toxin exposure contribute to the miscarriage rate. A thorough characterization of the molecular pathways that govern fertilization, early development, and embryonic robustness is necessary to mitigate the impact of these issues. The *Caenorhabditis elegans pos-1* gene is conserved from nematodes to humans and is required for embryo viability in both species. Here we describe how mutation of the *pos-1* 3′ untranslated region leads to a massive increase in POS-1 protein, which in turn causes a variety of defects in germline development, including sterility of some of the mutant progeny. Stress increases the frequency of the apparent phenotypes. Further work is needed to understand how loss of POS-1 regulation and stress conditions reduce reproductive capacity.

## Introduction

In plants and animals, mRNAs produced in the maternal germline are used by the embryo after fertilization to ensure success of the embryo [1]. Proteins produced from these transcripts guide early developmental decisions such as axis polarization, segregation of germline and soma, and cell fate specification prior to the onset of zygotic transcription [2–6]. Consistent with this model, forward genetic screens of the nematode *Caenorhabditis elegans* identified numerous RNA-binding proteins (RBPs) essential for germline and early embryonic development [7–11]. Many of the factors involved are conserved from humans to worms [12]. In addition, the 3′ untranslated regions (UTRs) of maternal transcripts were shown in a series of reporter studies to be essential for patterned expression in the germline and embryos [13,14].

There are multiple CCCH-type tandem zinc finger (TZF) family RBPs encoded in the *C. elegans* genome, several of which contribute to oocyte maturation and early embryonic patterning [9–11], as do their human and murine orthologs [15,16]. Here, we focused on *pos-1*, required for posterior cell fate specification in the embryo [9,17]. Null mutation of the *pos-1* gene causes maternal effect embryonic lethality with terminal embryos that fail to specify intestine and germ cells while developing a disorganized region of pharyngeal tissue [9]. POS-1 is thought to direct repression of maternal transcripts in the posterior of early embryos through binding to their 3′UTR [17]. POS-1 binds with high affinity to a linear, partially degenerate sequence motif known as the POS-1 repression element (PRE: UAU_2–3_RDN_1-3_G), though presence of a PRE is not sufficient to confer POS-1-mediated repression in animals [18]. The two well characterized targets of POS-1 repression include *glp-1* mRNA, a Notch receptor gene required for cell-to-cell signaling that drives anterior patterning decisions [17], and *neg-1*, a nuclear-localized protein expressed in anterior lineage cells that blocks mesoderm cell fates [19].

**Fig 1:**
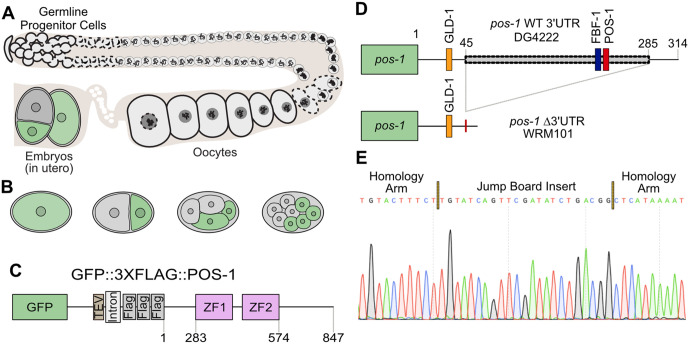
POS-1 is a germline RNA binding protein required for posterior cell fate specification in the early embryo [9]. **A.** Diagram of a *C. elegans* hermaphrodite gonad [1]. **B.** The pattern of POS-1 expression in early embryos is shown in green. In older embryos POS-1 is eventually restricted to the P-lineage [9]. **C.** In the genetic background used in this study (DG4222), the endogenous *pos-1* locus, which contains two CCCH-type zinc finger domains (labeled in pink), has an N-terminal *gfp::tev::3xflag* tag [26]. **D.** The ΔUTR allele replaces 241 of the 314 nucleotides of the *pos-1* 3′UTR with a 23 nucleotide jump board sequence [27]. GLD-1, FBF-1, and POS-1 binding motifs are shown. **E.** Chromatogram of the mutant confirming the deletion and jump board insertion. A portion of each flanking homology arm are shown on either side of the incorporated sequence.

POS-1 protein is produced from maternal mRNA shortly after fertilization (Fig 1A and 1B) [9]. It accumulates in the posterior of the one-cell zygote [9]. At the first cell division, POS-1 is asymmetrically inherited in the P1 blastomere. In four-cell embryos, POS-1 is observed in the P2 (germline) and EMS (intestine, muscle) blastomeres. POS-1 is observable for a few more rounds of cell division, where it accumulates in the p-granules of germline lineage cells. The anti-correlated pattern of POS-1 with NEG-1 and GLP-1 suggests that it functions to repress expression of these genes in the posterior [9,19]. Transgenic reporter studies in live embryos confirmed that POS-1 represses both genes through sequence specific interaction with their 3′UTRs [19,20].

Spatial and temporal patterning of POS-1 is thought to involve both post-translational and post-transcriptional regulatory mechanisms. Contributing factors that have been identified include RNA-binding proteins and Polo-like kinases [21,22]. To test the hypothesis that *pos-1* mRNA is regulated at the post-transcriptional level, we used CRISPR-Cas9 genome editing to generate a large deletion allele in the endogenous *pos-1* 3′UTR (Fig 1C and 1D). Worms that harbor this allele are viable as homozygotes. However, in a sensitized background, they display a complex pleiotropic phenotype that is distinct from the *pos-1* null mutant phenotype. Most progeny are sterile with absent or undersized gonads that do not undergo oogenesis. Others show defects in oocyte development or maturation. Most of the phenotypes become more penetrant at higher temperatures. Fertile animals show high POS-1 expression throughout the germline but reduced POS-1 expression in embryos compared to wild-type. In this regard, the *pos-1* 3′UTR mutant behaves similar to a 3′UTR deletion mutation recently characterized in the maternal *mex-3* gene [23,24]—another maternally-supplied RBP that determines cell fate [7]—though the reproductive phenotypes are different. This parallel adds support to an emerging model where the 3′UTRs of maternal mRNAs contribute to silencing in the maternal germline and buffering expression in embryos, especially during periods of stress.

## Results

### Mutating the *pos-1* 3′UTR using CRISPR-Cas9 genome engineering

We set out to test whether the endogenous *pos-1* 3′UTR plays a role in reproductive fecundity. Following the method of Ghanta et al., we used CRISPR-Cas9 genome engineering to remove the majority of the *pos-1* 3′UTR [25] in both the wild-type N2 (Bristol) strain and in strain DG4222 (Table 1), which includes an N-terminal *gfp::tev::3xflag* fusion tag knocked into the endogenous *pos-1* locus [26] (Fig 1C). We used a repair template that includes a jump board sequence containing a non-native guide RNA (gRNA) site and protospacer adjacent motif (PAM) site to simplify further editing [27]. The resulting alleles are identical in both genetic backgrounds, removing 239 of the 314 nucleotides found in the *pos-1* 3′UTR and replacing them with the 23 nucleotide jump board (Fig 1D). The deletion removes several motifs predicted to be recognized by RNA-binding proteins including OMA-1/2, FBF-1, as well as POS-1 itself [28–30]. Both alleles (denoted as GFP-ΔUTR and ΔUTR hereafter) were confirmed by PCR, gel electrophoresis, and sequencing (Fig 1E). Mutant worms harboring the 3′UTR deletion can be propagated as homozygotes under standard growth conditions in both genetic backgrounds. (NGM Agar, *Escherichia coli* OP50 food, 20ºC) [31].

**Table 1:** Genotype.

Strain ID	Genotype	Technology
Published Strains		
N2	*wild isolate*	*n/a*
DG4222	*pos-1(tn1730[gfp::tev::3xflag::pos-1]) V*	*CRISPR*
Strains Produced for this Study		
WRM85	*pgl-1(spr20[mCherry::pgl-1]) IV*	*CRISPR*
WRM101	pos-1(spr28[tn1730*(gfp::tev::3xFlag::pos-1::ΔUTR)]) V	*CRISPR*
WRM102	*pos-1(spr29 [ΔUTR])* V	*CRISPR*
WRM104	pgl-1(spr20[mCherry::pgl-1]) IV; pos-1(spr28[tn1730*(gfp::tev::3xFlag::pos-1::ΔUTR)]) V	*CRISPR*
WRM105	*pgl-1(spr20[mCherry::pgl-1]) IV; pos-1(tn1730[gfp::tev::3xflag::pos-1]) V*	*CRISPR*

### Deleting the *pos-1* 3′UTR impacts reproductive fecundity

We set out to quantify the reproductive health of untagged ΔUTR worms compared to the parent strain (N2: WT UTR hereafter). We also assessed the fertility of each worm’s F1 offspring. To do so, we measured the brood size (total embryos laid), hatch rate (hatched embryos/total brood), and sterility rate (number of sterile progeny/total viable hermaphrodite progeny) of ΔUTR worms compared to WT UTR controls. All three parameters were determined at two culture conditions—20ºC, the optimal temperature for *C. elegans* growth [31], and 25º, a mild stress condition that can enhance reproductive phenotypes [32].

**Fig 2:**
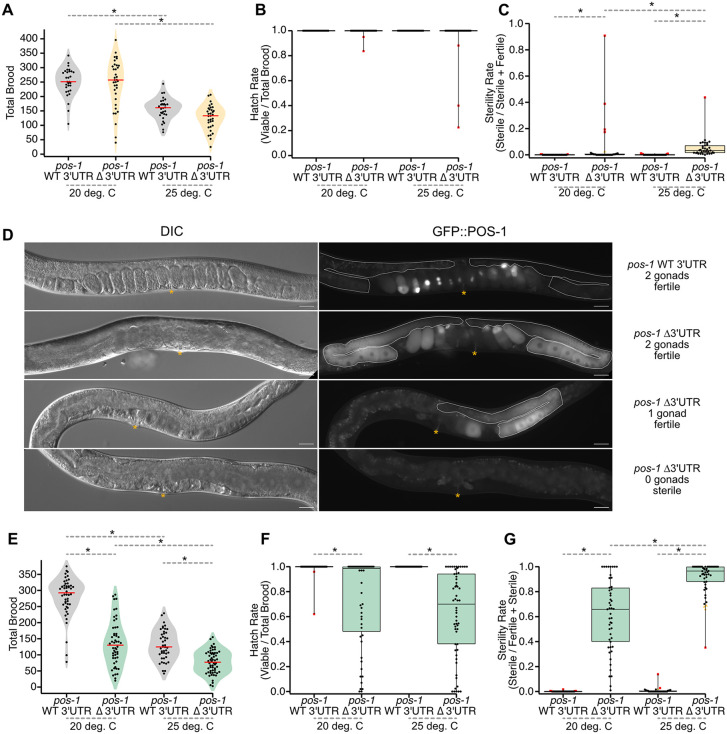
The *pos-1* 3′UTR contributes to reproductive robustness and germline formation. **A.** Violin plots of total brood for WT UTR (gray) and ∆UTR (orange) strains. The red lines indicate the median. Each point represents the total number of embryos produced by a single hermaphrodite. Asterisks indicate statistical significance in a one-way ANOVA with Bonferroni correction for multiple hypothesis testing. **B.** Box-and-whisker plot representing the hatch rate (viable/total brood) of embryos from panel **A.** Each dot represents the hatch rate of embryos from a single hermaphrodite. Red points indicate far outliers in a Tukey analysis (far outliers are at least 3X larger or smaller than the inner quartile range). **C.** Box-and-whisker plot depicting the sterility rate (sterile/sterile + fertile) of viable worms from panel **B** once they reach adulthood. Each dot represents the ratio of sterile F1 hermaphrodites to total viable hermaphrodites. Orange points indicate outliers in a Tukey analysis (1.5X larger or smaller than the inner quartile range). Far outliers and statistical significance are represented as in panel **A. D.** 20X DIC and GFP images of GFP-WT UTR and GFP-∆UTR mutants at 20°C. The yellow asterisk indicates the vulva, and the dotted white line represents each gonad arm, if present. Scale bars: 30 μm. **E.** Violin plot of total brood comparing between GFP-WT UTR (gray) and the GFP-∆UTR (green) at 20°C and 25°C. Median bars, statistical significance, and individual points are represented as in panel **A. F.** Box-and-whisker plot depicting the hatch rate of embryos from panel **E.** Each dot represents the hatch rate of embryos produced from a single worm as in panel **B. G.** Box-and-whisker plot depicting the sterility rate of viable progeny from panel **F** at adulthood. The representation is as described for panel **C.**

The average brood produced by untagged ΔUTR hermaphrodites is similar to N2 worms at 20ºC (Average Brood_ΔUTR_ = 245 ± 14, Average Brood_N2_ = 250 ± 9, p_adj_ = 1.0) and 25ºC (Average Brood_ΔUTR_ = 127 ± 7, Average Brood_N2_ = 157 ± 7, p_adj_ = 0.25, Fig 2A). Similarly, the average hatch rate between WT N2 and the untagged *pos-1* ∆3′UTR strain is indistinguishable at both temperatures. One hundred percent of the brood of N2 hermaphrodites hatched under standard growth conditions or mild temperature stress (N2; Average Hatch_WT-20°C_ = 100% ± 0% and Average Hatch_WT-25°C_ = 100% ± 0%, p_adj_ = 1.0). The ΔUTR strain had a minor reduction in hatchings from total brood at both temperatures, but neither was statistically significant (untagged ΔUTR; Average Hatch_∆UTR-20°C_ = 99.4% ± 0.5%, and Average Hatch_∆UTR-25°C_ = 95% ± 3%, p_adj_ = 0.39, Fig 2B). These data indicate that mutating the *pos-1* 3′UTR has an insignificant impact on the brood size and hatch rate of *C. elegans* embryos in an otherwise wild-type background.

By contrast, some of the viable progeny of ΔUTR worms are sterile at both temperatures. At 20ºC, approximately half of the worms produced at least some sterile F1 progeny (n = 17/35). Four animals had relatively high sterility rates (red dots, Fig 2C), including one animal that produced 91% sterile progeny. WT UTR animals also produced some sterile progeny at 20ºC, but the sterility rate was much lower (n = 4/30, max sterility rate = 0.42%, p_adj_ = 0.015). At 25ºC, nearly all ΔUTR animals produced sterile progeny (n = 32/33), with a maximum sterility rate of 44%. WT UTR animals had a much lower sterility rate with a maximum sterility rate of 1.2% (n = 5/30, p_adj_ < 6.04e-10). The data show that loss of the *pos-1* 3′UTR causes a partially penetrant F1 sterility phenotype that is exacerbated by elevated temperature.

In the *gfp::tev::3xflag* tagged background, the ΔUTR phenotypes appear to be much stronger. While observing homozygous GFP-ΔUTR mutants on plates, we noticed that a large fraction of adult animals appears to lack gonads and do not contain embryos (Fig 2D). These worms are easily distinguished from fertile animals under a light microscope by the appearance of a “dark gut” morphology and the absence of embryos [33]. We also noted an accumulation of dead embryos on the plate.

To quantify the fecundity of fertile GFP-ΔUTR worms, we repeated the brood size, hatch rate, and sterility rate measurements, comparing GFP-ΔUTR mutants to GFP-WT UTR (DG4222, Table 1) controls. Fertile GFP-ΔUTR worms had a lower total brood than GFP-WT UTR controls at both temperatures (Fig 2E). At 20ºC, GFP-WT UTR worms produced an average of 281 ± 9 embryos. By contrast, GFP-ΔUTR worms produced an average of 135 ± 10 total embryos (Fold Effect_ΔUTR/WT UTR_ = 0.48, p_adj_ < 1e-8). At 25ºC, the total brood is further reduced in both strains. GFP-WT UTR worms produced an average of 130 ± 7 total embryos, while GFP-ΔUTR worms produced 78 ± 5 (Fold Effect_ΔUTR/WT UTR_ = 0.6, p_adj_ = 5.6e-7).

The fraction of hatching embryos was also impacted in the GFP-ΔUTR mutant at both temperatures. At 20ºC, the fraction of GFP-WT UTR embryos that hatch is 0.99 ± 0.008, while the fraction of GFP-ΔUTR embryos that hatch is 0.73 ± 0.05 (Fig 2F, p_adj_ = 2e-5). Similarly, at 25ºC, all the GFP-WT UTR embryos hatched, while only 0.62 ± 0.04 GFP-ΔUTR embryos hatched (p_adj_ = 3e-10). As such, adult GFP-WT UTR animals produce an average of 278 viable progeny at 20ºC and 130 viable progeny at 25ºC, while GFP-ΔUTR adults produce an average of 99 viable progeny at 20ºC and just 48 at 25ºC. This reduction in fecundity encompasses both reduced embryo production and reduced embryo viability, suggesting problems in the germline as well as the embryo.

In addition to reduced brood size and hatch rate, F1 sterility is a common phenotype in GFP-ΔUTR homozygotes (Fig 2G). At 20ºC, GFP-WT UTR animals produced at least some sterile progeny 15% of the time (n = 7/48), but the average sterility rate was very low (0.1%). In contrast, all GFP-ΔUTR animals produced at least some sterile progeny (n = 49/49, p_adj_ < 1e-10), and the average sterility rate was much higher (68%, p_adj_ < 1e-10), with all sterile animals lacking both gonad arms. At 25ºC, the fraction of GFP-WT UTR worms producing sterile animals increased (GFP-WT UTR = 34%, n = 15/44), but the sterility rate remained low (0.7%). In GFP-ΔUTR animals, once again all worms produced at least some sterile progeny (n = 52/52, padj < 1e-10), but the average sterility rate increased to 91% (p_adj_ < 1e-10). The results suggest a defect in germline development or germline maintenance in mutant F1 animals compared to genotype-matched controls.

### Additional defects in *pos-1* 3′UTR mutants

**Fig 3:**
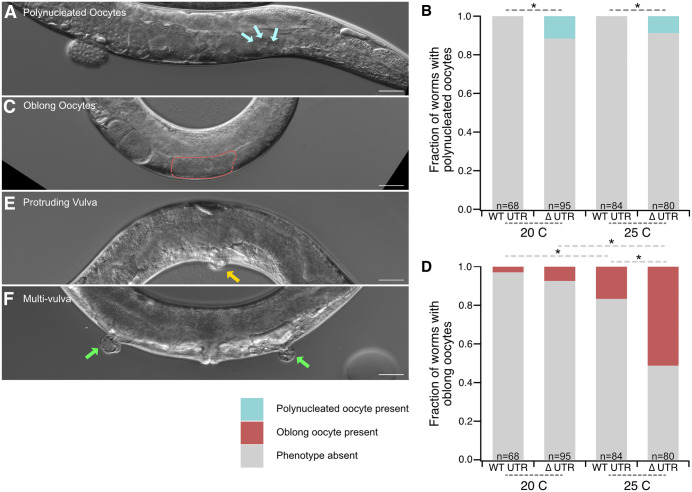
Germline phenotypes in the *pos-1* ∆3′UTR mutant. **A.** DIC image of a GFP-∆UTR mutant with a polynucleated oocyte (cyan arrows–each points to a different nucleus within the same oocyte). **B.** Stacked bar graph showing the fraction of worms with polynucleated oocytes (cyan) or normal oocytes (gray) in germline images. Asterisks indicate statistical significance in a Pearson’s chi square test with Bonferroni correction for multiple hypothesis testing. **C.** DIC image of a GFP-∆UTR oblong oocyte phenotype. The length of most proximal oblong oocyte is marked in red. **D.** Stacked bar graph showing fraction of worms with oblong oocytes (red) or normal oocytes (gray) in germline images. Statistical significance is denoted as in **B. E.** DIC image of a representative GFP-∆UTR mutant with protruding vulva phenotype (pvul, yellow arrow). **F.** DIC image of a GFP-∆UTR mutant with a multiple vulva (muv) phenotype. Each abnormal vulva location is marked with a green arrow. The scale bars represent 30 μm in all images.

We observed two additional low penetrance phenotypes in fertile GFP-ΔUTR adults compared to GFP-WT UTR controls. The first is the presence of immature oocytes with multiple nuclei (polynucleated oocytes, Fig 3A and 3B), suggesting errors in cytokinesis, apoptosis, or gamete fusion during oogenesis [34]. GFP-WT UTR worms do not form polynucleated oocytes (20ºC, n = 0/68; 25ºC, n = 0/84), but the GFP-ΔUTR mutants show this phenotype at both growth temperatures (20ºC, n = 11/95 [11.6%], p_adj_ΔUTR20vsWTUTR20_ = 0.022; 25ºC, n = 7/80 [8.75%], p_adj_ΔUTR25vsWTUTR25_ = 0.034). We also observed the presence of unusually oblong oocytes, wider than they are tall (Fig 3C and 3D). Oblong oocytes were present in both GFP-ΔUTR and GFP-WT UTR control animals, but they were most prevalent in the GFP-ΔUTR mutants at elevated temperature. At 20ºC, oblong oocytes are found in 2.9% (20ºC, n = 2/68) of GFP-WT UTR control animals and 7.4% of GFP-ΔUTR animals (20ºC, n = 7/95), a nonsignificant difference (p_adj_ΔUTR20vsWTUTR20_ = 1). At 25ºC, the phenotype becomes more prevalent, with 51.3% of GFP-ΔUTR animals (25ºC, n = 41/80) and 16.7% (25ºC, n = 14/84) of GFP-WT UTR animals showing oblong oocytes. This increase is statistically significant (p_adj_ΔUTR25vsWTUTR25_ = 1.7e-5). The results are consistent with partially penetrant oogenesis and/or oocyte maturation phenotypes in the fertile subset of GFP-ΔUTR mutant worms compared to genotype matched control. Some phenotypes depend on the temperature of growth, but the impact of temperature appears unique for each phenotype observed.

In addition to these, we observed a variety of incompletely penetrant somatic phenotypes including protruding vulva and multivulva animals (Fig 3E and 3F). These phenotypes only occurred in GFP-ΔUTR mutants. We did not quantify their penetrance or temperature dependence. We also note that vulval morphogenesis defects may be the root cause of our difficulty in outcrossing the ΔUTR allele.

### Whole genome sequencing of ΔUTR strains

We were surprised that the phenotypes appeared much stronger in the GFP-tagged background than in the untagged mutants. We also noted that some phenotypes manifest at low penetrance in the GFP-WT UTR control strain, including low penetrance sterility (Fig 2G) and oblong oocyte (Fig 3C) phenotypes. Homozygous GFP-WT UTR animals were outcrossed three times prior to CRISPR mutagenesis. However, the resultant GFP-ΔUTR mutant animals mate inefficiently, and as such could not be outcrossed. Attempted matings resulted in animals dying due to bursting through the vulva, potentially due to the vulval morphogenesis defects described above. As such, it remained possible that background mutations arising during CRISPR mutagenesis could be responsible for the strong phenotypes we observed.

To identify potential confounds in the GFP-tagged genetic background, we performed whole genome sequencing on the ΔUTR strain (WRM102), the GFP-ΔUTR strain (WRM101), and the GFP-WT UTR strain (DG4222). The results confirmed the presence of the ΔUTR allele in the *pos-1* locus of both the ΔUTR and GFP-ΔUTR strains (S1 Data and S1A Fig). The data also identify 105 exonic single nucleotide polymorphisms (SNPs) in the GFP-ΔUTR animals with an allele frequency of one compared to the reference genome (S1B Fig). All 105 are also found in the GFP-WT UTR background, although six are found at lower allele frequency. Fifty of the 105 SNPs are also found in the untagged ΔUTR mutant. The data also revealed 32 exonic indels in the GFP-ΔUTR strain (S1C Fig). All but one are also found in the GFP-WT UTR background. The sole exception is found in the untagged ΔUTR background, which shares 26 of the 31 exonic indels with the GFP-ΔUTR strain, and does not display strong phenotypes. Because none of the SNPs or indels are unique to the GFP-ΔUTR strain, it is unlikely that the phenotypes are caused solely by a background mutation. However, the differences in phenotype between both GFP-tagged strains and the untagged ΔUTR strain suggest that the GFP-tagged background is sensitized, leading to higher penetrance phenotypes. It is possible that the *gfp::tev::3xflag* tag itself contributes to the stronger phenotype. The data show that the GFP-WT UTR strain is a suitable control for the GFP-ΔUTR strain.

### Mutation of the endogenous *pos-1* 3′UTR causes strong germline expression

**Fig 4:**
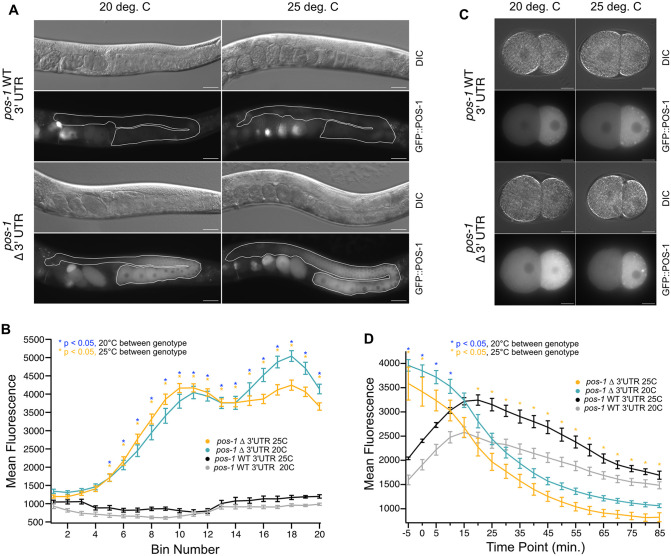
POS-1 protein expression is affected in the germline and embryos of 3′∆UTR mutants. **A.** DIC and GFP images of GFP-WT UTR and GFP-∆UTR germlines (white outline) revealing POS-1 protein overexpression differences between genotype and condition. The scale bars represent 30 μm. **B.** Plot of germline fluorescence intensity as a function of distance from the distal end, with average GFP::POS-1 fluorescence averaged across twenty equal width bins representing the full length of the germline. The points represent the average fluorescence intensity from multiple images within a given genotype and condition; the error bars represent the standard error of the mean. Statistical significance is shown with asterisks as labeled in the key. P values were calculated with a one-way ANOVA with Bonferroni correction for multiple hypothesis testing. **C.** DIC and GFP image of GFP-WT UTR and GFP-∆UTR embryo at the two-cell stage. The posterior pole is oriented to the right. Scale bars: 10 μm. **D.** Average GFP::POS-1 fluorescence intensity across the entire embryo is plotted as a function of time from embryogenesis movies collected in five-minute intervals over 90 minutes of early embryonic development. Statistical significance is represented as in panel **B.**

To define the pattern of GFP::POS-1 expression, GFP-WT UTR and GFP-ΔUTR young adult worms were imaged under a fluorescence microscope at both temperatures. Though both sterile and fertile progeny of GFP-ΔUTR worms were imaged (Fig 2D), the comparisons described below focus on the subset of ΔUTR animals that appear fertile. In GFP-WT UTR worms, the GFP::POS-1 expression pattern was restricted to young embryos (Fig 4A) as expected [9]. By contrast, GFP::POS-1 protein expression is strongly dysregulated in fertile GFP-ΔUTR mutants that contain intact gonads. High GFP::POS-1 expression is observed in the germline pachytene region (Bin 11, Fold Increase _∆UTR/WT_ = 5.7, p_adj_ < 0.00001, Fig 4B), in the loop region (Bin 13, Fold Increase _∆UTR/WT_ = 4.1, p_adj_ < 0.00001), and in oocytes (Bin 18, Fold Increase _∆UTR/WT_ = 5.2, p_adj_ < 0.00001). A smaller increase is observed in mitotic germline progenitor cells at the distal end of the germline (Bin 0, Fold Increase _∆UTR/WT_ = 1.4, p_adj_ = 0.00001). The results suggest that the *pos-1* 3′UTR is essential for repressing POS-1 protein production in the maternal germline.

Next, we sought to assess the impact of temperature stress on this pattern of expression. We repeated our imaging experiments with worms cultured at 25ºC. As expected, increasing the temperature caused no change in the pattern of expression in GFP-WT UTR worms—germline expression is not observed at either temperature. GFP-ΔUTR mutants continued to display strong fluorescence throughout the germline (Fig 4B). There are no statistically significant differences within a genotype as temperature increases.

We noted that a subset of fertile GFP-ΔUTR young adults had only one gonad arm. For GFP-WT UTR worms cultured at 20ºC or 25ºC, none of the imaged worms had fewer than two gonad arms (20ºC, n = 0/68; 25ºC, n = 0/84). For ΔUTR animals cultured at 20ºC, 11.4% had just one gonad arm (20ºC, n = 10/95, p_adj_ = 0.00051, Fig 2D). Surprisingly, this phenotype appears to be absent at 25ºC. None of the fertile GFP-ΔUTR worms had just one gonad arm (25ºC, n = 0/80, p_adj_ΔUTR25vs20_=0.00024). This change corresponds with an increase in sterility (Fig 2G), suggesting that at elevated temperature, more animals have zero gonads. The data indicate that *pos-1* 3′UTR mutants produce a mixed population of progeny where some animals lack one or both gonad arms, and that elevated temperature alters the distribution.

### GFP::POS-1 abundance rapidly decreases in ∆3′UTR mutant embryos post-fertilization

To determine whether GFP::POS-1 protein abundance remains high after fertilization, we recorded images of embryogenesis under a fluorescence microscope as a function of time at five-minute intervals (Fig 4C and S1 and S2 Movies). For each frame, the total fluorescence intensity of GFP::POS-1 within the embryo was measured. The first frame in which cellular division is observed was arbitrarily set to time zero to facilitate comparisons between embryos. Total fluorescence values were averaged at each time point over multiple movies (n = 7–10) within each genotype and growth condition (20ºC or 25ºC).

At early time points (first 15 minutes), GFP-ΔUTR mutant embryos have higher average POS-1 fluorescence values than similarly aged GFP-WT UTR embryos (p_adj_ < 3.5e-6, Fig 4D). At t = 0 and 20ºC, ∆UTR mutant embryos have a mean pixel intensity twice that of WT UTR embryos of the same age (t = 0 minutes, 20ºC, Fold Increase _∆UTR/WT_ = 2.0, p_adj_ < 1e-10). The fold increase comparing the GFP-ΔUTR to the GFP-WT UTR animals at 25ºC is not as high, but remains statistically significant (t = 0 minutes, 25ºC, Fold Increase _∆UTR/WT_ = 1.5, p_adj_ = 0.01, Fig 4D). The data indicate that the POS-1 protein found within mutant embryos is higher in abundance compared to WT expression at both temperature conditions immediately following fertilization.

The pattern of GFP::POS-1 accumulation does not appear to be affected by the 3′UTR mutation. In two-cell embryos, most of the GFP::POS-1 protein accumulates in the P1 cell (posterior) in both genotypes at both temperatures (Fig 4C). However, the total abundance of GFP::POS-1 appears to be impacted by both genotype and temperature. Though GFP::POS-1 is initially much higher in mutant embryos than control embryos, it declines to a level lower than GFP-WT UTR embryos over the course of early embryogenesis (Fig 4D). The decay of GFP::POS-1 in GFP-ΔUTR embryos follows a sigmoidal curve. The time with half maximal concentration of GFP::POS-1 (incorporating the rates of both new GFP::POS-1 translation and decay) is t = 20.3 ± 3 minutes at 20ºC and t = 15 ± 8 minutes at 25ºC. By contrast, GFP::POS-1 abundance increases in GFP-WT UTR animals following a modified Gaussian curve with a peak at t = 12 ± 2.4 minutes at 20ºC and t = 16.3 ± 1.2 minutes at 25ºC. After 20 minutes, GFP::POS-1 levels are lower in GFP-ΔUTR_25ºC_ embryos than in GFP-WT UTR_25ºC_ embryos and remain lower for the duration of the recording (t = 85 min, 25ºC, Fold Decrease _ΔUTR/WT_ = 0.49, p_adj_ = 0.009). The trend is similar at 20ºC, but the apparent differences are not statistically significant (p_adj_ > 0.05). Together with our previous observations, the data suggest a model where the *pos-1* 3′UTR represses POS-1 expression in the germline but enhances POS-1 expression after fertilization. Temperature also appears to impact GFP::POS-1 differently in GFP-ΔUTR embryos compared to GFP-WT UTR embryos.

### Embryonic germ cell specification defects in the *pos-1* Δ3′UTR mutant

The frequent absence of gonad arms in the GFP-ΔUTR mutant could be caused by germ cell misspecification during embryogenesis, failure of germline proliferation during larval growth, or both. PGL-1 is a core component of germ granules, found exclusively in germline lineage cells, and makes a convenient marker for germ cell differentiation in embryos and germline morphology in adults. In early embryos, PGL-1 is found in the P-lineage cell (P1-P4) [35]. Around three hours post fertilization, the P4 cell divides to produce both the Z2 and Z3 cell, the primordial germ cells (PGCs) that ultimately form the germline in both gonads. The Z2 and Z3 cells remain in a quiescent state until the L1 larval stage, when they begin to divide again.

**Fig 5:**
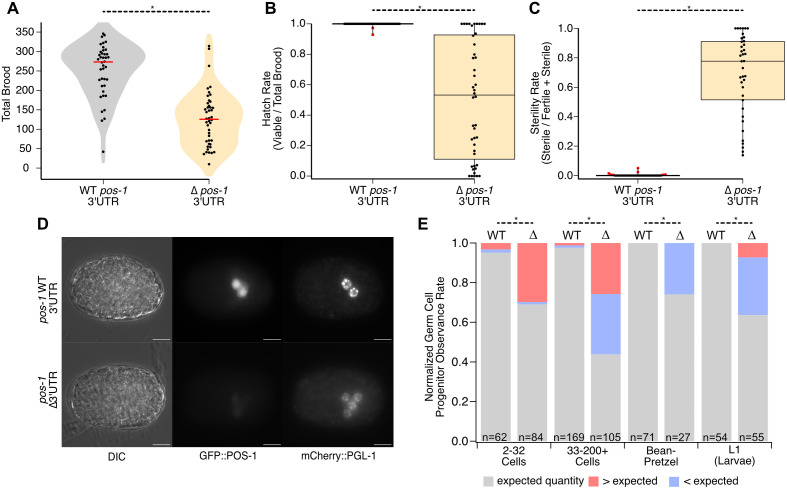
Germline progenitor specification is aberrant in *pos-1* Δ3′UTR mutants. **A.** Brood size of *mCherry::pgl-1* marked GFP-WT UTR and GFP-ΔUTR strains. The representations are as in Fig 2A. **B.** Hatch rate of the embryos from panel **A.** The representation is as in Fig 2B. **C.** Sterility rate of the viable progeny from panel **B.** The graph is represented as in Fig 2C. **D.** DIC, GFP, and mCherry images of embryos from GFP-WT UTR or GFP-ΔUTR animals tagged with mCherry::PGL-1 showing an example of increased germline progenitor cells in embryos in the 3′UTR deletion mutant. Scale Bars: 20 μm. **E.** Fraction of animals with less than (blue), equal to (gray), or more than (red) the expected number of germline progenitor cells in embryo images binned by developmental stage. Statistically significant differences from a Pearson’s Chi Square test with Bonferroni correction are marked with asterisks.

To test our hypotheses, we crossed the GFP-ΔUTR strain and the GFP-WT UTR control strain with worms expressing mCherry::PGL-1 from the endogenous *pgl-1* locus (WRM85). The resultant strains (WRM104 and WRM105, Table 1) have similar brood size, hatch rate, and sterility rate compared to genotype-matched strains lacking mCherry::PGL-1, suggesting that the mCherry tag on PGL-1 does not modify the ΔUTR phenotypes (Fig 5A-5C).

We first measured p-granule abundance in early embryos, GFP-WT UTR and GFP-∆UTR embryos harboring the mCherry::PGL-1 marker were imaged (S2A Fig). Embryos were categorized based upon appearance into three groups: one-cell, two-cell, and four-cell embryos. The quantity of p-granules was counted per embryo in each group for both genotypes. One-cell mutant embryos appear to contain fewer p-granules than genotype matched controls lacking the UTR mutation (one-cell, Fold Effect _∆UTR/WT_ = 0.52, p_adj_ = 0.004, S2B Fig). No difference is observed at the two-cell or four-cell stage.

Next, to count primordial germ cells within embryos, older embryos recovered from the same strains were imaged (Fig 5D). The images were binned by genotype and by age—estimated from the cell count or morphological appearance—and binned into three groups: (1) early embryos from the 2–32 cell stage, where we expect exactly one mCherry::PGL-1 positive cell, (2) 33–200 + intermediate age embryos where both Z2 and Z3 should score positive for mCherry::PGL-1, and (3) older gastrulating embryos in the “bean” through “pretzel” stages that should also contain two mCherry::PGL-1 cells [35]. The quantity of mCherry positive cells was determined per embryo in each group for both genotypes. We also determined the number of germ cells in synchronized L1 larvae that were recovered by bleaching and allowing embryos to hatch overnight in the absence of food. L1 larvae were randomly selected for imaging shortly after plating.

Most of the embryos from GFP-WT UTR adults in the 2–32 cell stage had the expected number of mCherry positive cells (91%, one cell expected, n = 58/64, Fig 5E). In contrast, just 69% (n = 58/84, p_adj_ = 0.00028) of GFP-ΔUTR embryos in this stage had the expected number of cells. Nearly 30% of GFP-ΔUTR mutants had more than one cell, with the highest single-embryo quantity at seven (Fig 5D). This phenotype contrasts with *pos-1* null embryos, which fail to specify germ cells [9]. In the 33–200 + cell embryos, we observed something different. Again, nearly all the GFP-WT UTR embryos had the expected number of cells (98%, two cells expected, n = 165/169). However, only 44% of GFP-ΔUTR embryos at this age had two cells (n = 46/105, p_adj_ < 1e-10). The remaining embryos were split between those with fewer than expected germ cells (30%, 0–1 mCherry positive cells, n = 33/105) and those with more than expected germ cells (26%, > 2 mCherry positive cells, n = 26/105).

As embryos aged, the distribution changed again. By the bean–pretzel stage, all the GFP-WT UTR embryos had the two expected PGCs (n = 71), while 74% of GFP-ΔUTR embryos at this stage had the expected amount (two cells, n = 20/27, p_adj_ = 8.5e-6). We note that it was relatively difficult to recover mutant embryos of this age, presumably due to arrest or death prior to gastrulation, consistent with the hatch rate data (Figs 2F and 5B). We also observed that all the remaining embryos had fewer than the expected number of germ cells (0–1), and none had more than expected (Fig 5E). This suggests that embryos that specified extra germ cells failed embryogenesis before entering gastrulation, while those that specified 0–2 germ cells continued to develop. Interestingly, 62% of the L1 larvae that hatched from the GFP-ΔUTR mutant embryos contained the expected two germ cell progenitors (n = 35/55) while all the GFP-WT UTR larvae had the expected number (n = 54/54, p_adj_ = 6.0e-6). Most of the remaining mutant larva had fewer than two germ cells (29%, n = 16/55), but we note that a few had more than expected (7%, n = 4/55). These animals could represent embryos that successfully hatched after mis-specifying extra germ cells—contrary to our hypothesis—or they could be animals that exited quiescence to initiate germline proliferation early.

### Germline proliferation defects in the *pos-1* 3′UTR mutant

**Fig 6:**
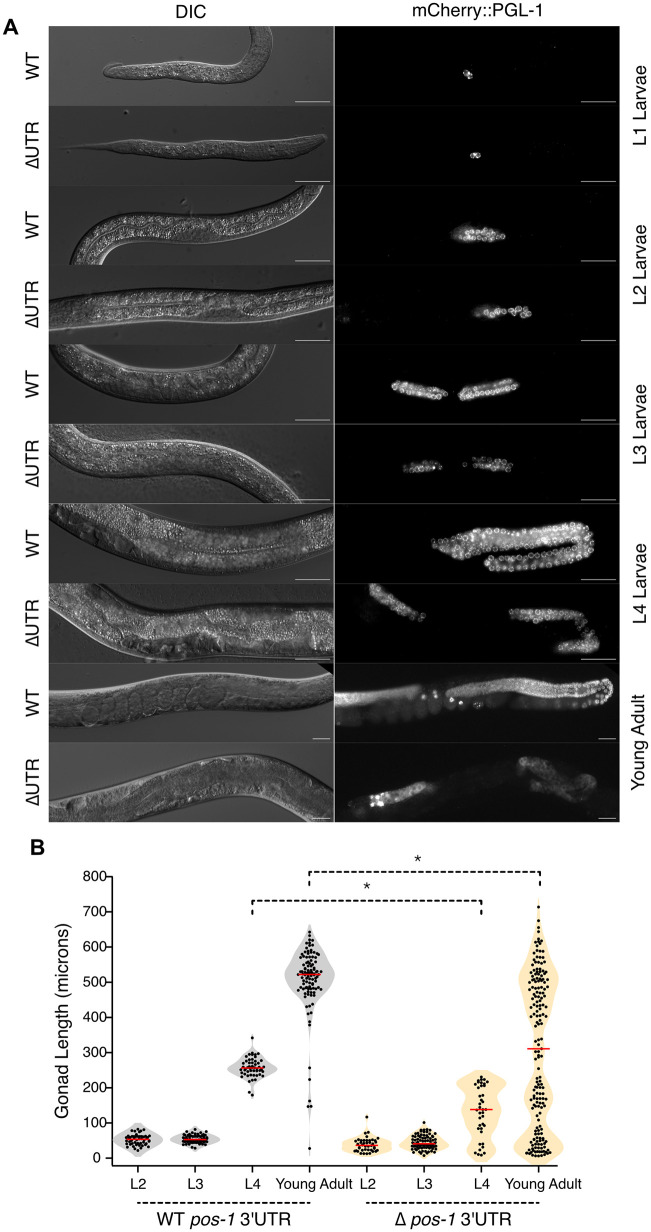
Germline proliferation is impacted in *pos-1* 3′UTR mutants. **A.** DIC and mCherry images of mCherry::PGL-1 marked GFP-WT UTR or GFP-ΔUTR mutants as a function of stage from L1 larva to adults. Scale bars: 20 μm. **B.** Violin plots of germline length as a function of developmental stage. Each point is the length of a single gonad arm as defined by the zone of mCherry::PGL-1 expression. Gray represents GFP-WT UTR animals, orange denotes GFP-ΔUTR animals. The red bar indicates the median. The asterisks indicate statistical significance in a one-way ANOVA with Bonferroni correction for multiple hypothesis testing.

In *C. elegans*, there are four larval stages prior to adulthood. During each of these four stages, the two germ cell progenitors (Z2 & Z3) proliferate, eventually forming the characteristic U-shaped gonad arms [36]. To monitor germ cell proliferation as a function of developmental stage, we synchronized mCherry::PGL-1 expressing GFP-ΔUTR and GFP-WT UTR animals and collected images across development (Fig 6A). The length of the germline was determined from mCherry::PGL-1 positive images using the segmented line tool in Fiji (ImageJ).

At the L2 and L3 larval stages, the average gonad length of GFP-ΔUTR larvae is not significantly different than GFP-WT UTR larvae (Average Length_WT-L2_ = 52 µm ± 2, Average Length_∆UTR-L2_ = 38 µm ± 3, p_adj_ = 1.0; Average Length_WT-L3_ = 54 µm ± 1.5, Average Length_∆UTR-L3_ = 45 µm ± 2, p_adj_ = 1.0, Fig 6A and 6B). However, in L4 larvae, GFP-ΔUTR mutant germline arms are half the length of GFP-WT UTR controls (Average Length_WT-L4_ = 257 µm ± 4, Average Length_∆UTR-L4_ = 130 µm ± 12, p_adj_ = 0.00008, Fig 6A and 6B). In young adults, the GFP-ΔUTR mutant germline arms are on average 40% shorter than their WT UTR counterparts (Average Length_WT-YA_ = 503 µm ± 10, Average Length_∆UTR-YA_ = 303 µm ± 16, p_adj_ < 1e-10). Some mutant gonads appeared to have unusual morphology. These data suggest that dysregulation of GFP::POS-1 in the germline impacts germline proliferation in the L4–young adult stages but not at earlier stages. The reduced size of the germline could also possibly explain the lower average brood size observed in Fig 2F.

### Dysregulation of *pos-1* impacts somatic and germline genes in multiple categories

**Fig 7:**
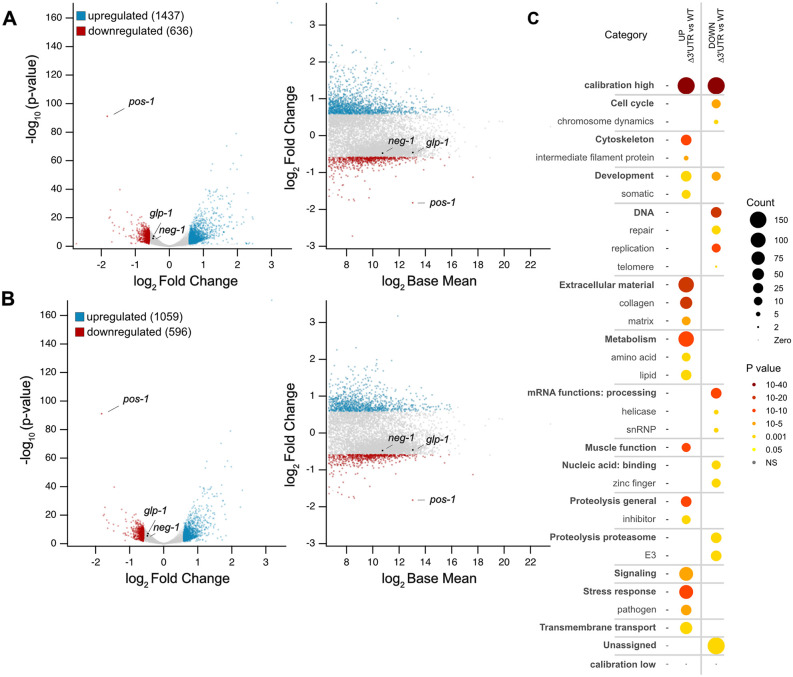
RNA sequencing reveals transcriptome-wide changes in *pos-1* 3′UTR mutants. **A.** Volcano and MA plots of differentially expressed genes from GFP-tagged *pos-1* WT and 3′UTR mutants grown at 20ºC. Downregulated genes that meet filtering criteria are shown in red, upregulated genes are shown in blue. Genes that are unchanged or do not meet the filtering criteria are shown in gray. The *pos-1*, *glp-1*, and *neg-1* genes are labeled in both plots. **B.** Volcano and MA plots of the differentially regulated germline genes. The representation is the same as in panel **A. C.** Differentially expressed gene sets from WormCat analyses of both up and downregulated genes. The WormCat categories are organized by function. The colors denote statistical significance in WormCat 2.0, and the size of the circle represents the number of genes in the category impacted.

To evaluate the transcriptome-wide impacts of disrupting the *pos-1* 3′UTR, we performed RNA-seq on synchronized young adult WT UTR and ΔUTR animals cultured at 20ºC. We used DESeq2 to identify differentially expressed transcripts across three biological replicates of each genotype [37]. This analysis identified 1437 significantly upregulated transcripts and 636 significantly downregulated transcripts with a log2 fold change cut-off of 0.585, a p_adj_ threshold of 0.05, and a base mean expression level of 100 (Fig 7A and S2 Data). The large number of differentially expressed genes likely reflects both direct effects and indirect impacts from the complex pleiotropic phenotype—including animals missing one or both gonads, oogenesis defects, and vulval differentiation issues.

Next, we compared the list of differentially expressed genes to an annotated library of all germline transcripts [38]. The comparison identified both upregulated and downregulated germline associated transcripts in GFP-ΔUTR mutants compared to GFP-WT UTR controls using the same cut offs (up=1059, down=596, Fig 7B). Most differentially expressed transcripts are germline associated genes (74% of upregulated genes, 94% of downregulated genes). The data suggest that loss of gonad arms in a large fraction of GFP-ΔUTR animals accounts for most of the downregulated transcripts. However, this phenotype cannot explain the preponderance of upregulated germline genes. We hypothesize that these changes are caused by the expanded germline expression pattern of POS-1 in the GFP-ΔUTR mutant. It is not clear why these transcripts are upregulated.

Next, we used WormCat 2.0 to perform a gene ontology analysis to identify gene sets that are dysregulated in the GFP-ΔUTR mutant [39]. Upregulated germline genes include those involved in extracellular material production (collagen, extracellular matrix), metabolism (lipids, amino acids), and stress response to pathogens, among others. Downregulated germline genes include those involved in DNA replication and repair, mRNA functions, zinc-finger genes, and cell cycle regulation. The data show that dysregulation of POS-1 has widespread impacts on germline genes involved in multiple pathways. How POS-1 exerts these impacts remains unknown.

POS-1 has been shown to regulate both *glp-1* and *neg-1* expression through direct binding to elements found in the 3′UTR of each gene [17,19,20]. The current model suggests that POS-1 regulates both transcripts at the level of translation, reducing the amount of protein produced per available mRNA without impacting mRNA abundance [19,20]. Consistent with that model, the amount of *glp-1* and *neg-1* mRNA remains unchanged in the GFP-ΔUTR mutant compared to the GFP-WT UTR control (Fig 7A and 7B). Intriguingly, the amount of *pos-1* mRNA is much lower in the mutant compared to the control (log2 fold change = -1.82, p_adj_ = 4.3e-88). Yet we observe much more GFP::POS-1 protein produced throughout the germline (Fig 4A). The data suggests that the *pos-1* 3′UTR contains elements that stabilize *pos-1* mRNA as well as elements that repress its translation in the germline. It is not yet clear what the elements are, or which trans-acting factors are involved in regulation. Several RBPs are predicted to bind to the *pos-1* 3′UTR, including FBF-1/2, GLD-1, OMA-1/2, and POS-1 itself.

## Discussion

Spirin first hypothesized in 1966 that oocytes contain “masked” mRNAs, produced and stored in the maternal germline, to be used by embryos after fertilization prior to the onset of zygotic gene activation [40]. The idea was that these mRNAs are necessary to ensure robust cell physiology at a time when DNA replication occurs rapidly and repeatedly. We now know that multiple embryonic cell fate determinants are transmitted from the maternal germline to embryos in mRNA form. The regiospecific activation of these transcripts after fertilization is thought to guide early developmental decisions such as cell fate specification, anterior-posterior axis formation, and differentiation of the germline from the soma [1]. In this study we show that the *pos-1* 3′UTR plays multiple roles in controlling POS-1 expression in the germline and in early embryos. The results shown here, as with another cell fate determining transcript *mex-3* [23,24], suggest that regulation of maternal mRNAs may not be as essential for embryonic survival as previously thought. Similar to *pos-1*, loss of the *mex-3* 3′UTR has a modest impact on reproductive success [23]. Only under stress conditions is its importance revealed [24]. The 3′UTRs of both transcripts appear to enhance reproductive robustness in conditions that are less than ideal, but neither is essential to reproduction. Additional studies on more maternal transcripts are needed to assess whether the lessons learned from *mex-3* and *pos-1*—two important maternal genes required for very early pattern formation—hold for most maternal mRNAs.

The most striking consequence of the *pos-1* 3′UTR deletion mutant is strong overexpression of POS-1 protein throughout the germline, a tissue where *pos-1* mRNA is normally transcribed but remains silenced at the translational level [9]. The consequence of dysregulated POS-1 translation appears to depend both on the genetic background and the growth condition. Under optimal growth in an otherwise healthy, wild-type background, worms tolerate dysregulation of *pos-1* without much impact on reproductive fecundity. However, when worms are stressed by temperature or sensitized by their genetic background, loss of the 3′UTR results in a complex, pleiotropic phenotype that impacts the brood size, hatch rate, and the fraction of progeny that are sterile. Specific defects include misspecification of germ lineage cells in the embryo, including some that have too few and others that have too many, as well as germline proliferation defects that manifest in L4 and young adult animals. We also detected multiple issues with oogenesis which could impact all three outcomes. The exact mechanism of how the *pos-1* 3′UTR ensures reproductive robustness during environmental or genotoxic stress remains unknown.

POS-1 has been shown to negatively regulate expression from two target mRNAs, *glp-1* and *neg-1*, through direct association to their 3′UTRs [17,19]. Neither mRNA changes in abundance when POS-1 is dysregulated, suggesting that POS-1 does not regulate the stability of either transcript mRNA. POS-1 competes with GLD-1 for binding to *glp-1* mRNA through a pair of overlapping binding sites in the *glp-1* 3′UTR [20]. GLD-1 is a STAR/GSG domain RNA-binding protein normally expressed in the pachytene region of the germline that promotes entry into meiosis and the switch from spermatogenesis to oogenesis [41,42]. Raising the concentration of POS-1 in the germline might impact GLD-1 repression of its mRNA targets for transcripts where POS-1 and GLD-1 binding motifs overlap [20]. The POS-1 motif also partially overlaps with motifs recognized by PUF domain RBPs including FBF-1/2, and MEX-3 [30,43], both of which are expressed in the distal germline and promote mitosis [30,44]. Elevated POS-1 could disrupt regulation by these RBPs, too. It is likely that POS-1 regulates additional transcripts beyond *glp-1* and *neg-1*, but there are no data sets available yet that identify all POS-1-associated mRNAs.

Why does the GFP-tagged *pos-1* 3′UTR allele yield stronger phenotypes than an identical mutation in an untagged background? There are two possibilities. In the first, genetic background differences between the tagged and untagged strains could account for the enhanced phenotypes. Because the tagged strain mates very inefficiently, we could not outcross the strain through the untagged background to eliminate unlinked mutations. Instead, we used whole genome sequencing to identify potential confounds. These data catalog all the background differences between strains and reveal that none are unique to the GFP-tagged 3′UTR deletion. Thus, the phenotypes we observed cannot be solely explained by background differences. The second possibility is that GFP itself could be influencing POS-1 function. GFP is known to be stabilizing, which could limit GFP::POS-1 turnover [45]. That, coupled to dysregulation from the 3′UTR deletion, could cause the stronger phenotypes observed in the tagged versus untagged backgrounds.

How does the 3′UTR contribute to *pos-1* repression in the germline? An alignment of *pos-1* homologs and paralogs from other *Caenorhabditis* species reveals regions of strong conservation (S3 Fig). One of the most well conserved regions contains binding motifs for FBF/PUF and POS-1 itself, suggesting that FBF and/or other PUF proteins could play a role in POS-1 repression, and that POS-1 itself may autoregulate, perhaps by competition for binding with the overlapping FBF motif. In contrast, a putative GLD-1 binding site, which is not impacted by our deletion, does not appear to be conserved across *Caenorhabditis.* More work will be needed to determine whether precise mutations that disrupt any of these motifs independently leads to reproductive consequences.

How does deletion of the *pos-1* 3′UTR manifest somatic phenotypes in a subset of the progeny? The imaging data show that POS-1 is expressed in the germline and in germline progenitor cells in the early embryo. Yet we observe two different types of somatic vulval morphogenesis defects, including protruding vulva and multivulva phenotypes in a subset of progeny. We also observe a dark gut phenotype. The RNA-seq data helps rationalize these observations. Loss of the *mrt-2* gene causes a dark gut phenotype like the one observed in some GFP-ΔUTR animals [33]. Transcripts encoding *mrt-2* are downregulated in GFP-ΔUTR compared to GFP-WT UTR controls. This gene encodes a DNA repair enzyme that regulates telomere length and causes a transgenerational mortal germline phenotype [46]. As such, reduction of this gene could contribute to both the dark gut and the loss of gonad phenotypes that we observed.

The genetic pathways that define vulval morphogenesis have been well characterized [47]. The protruding vulva (pvul) and multivulva (muv) phenotypes could be caused by myriad changes to transcript abundance for genes involved in regulating this pathway. We note that *lin-3*, an EGF-like ligand necessary for vulva cell induction [48], is downregulated in the RNA-seq data set. We also observe an increase in *lin-15a*, *lin-15b*, *cwn-1*, *cwn-2*, and *bar-1* transcripts, all genes involved in specifying vulval differentiation in vulva precursor cells. LIN-15A and LIN-15B are transcriptional regulators that negatively regulate vulva cell fate [49]. Simultaneous loss-of-function mutation of *lin-15A* and *lin-15B* causes a muv phenotype. CWN-1 and CWN-2 are both wnt signaling pathway ligands that contribute to vulval precursor cell polarity [50]. BAR-1 (beta-catenin) is the major effector of wnt signaling pathways [51]. When activated, this protein accumulates in the nucleus of vulval precursor cells to promote vulva fate. The complex mixture of pvul and muv animals in our mutants probably results from the dysregulation of multiple genes involved in vulval morphogenesis. It is not yet known how these genes are impacted by POS-1 dysregulation.

## Materials and methods

### Strains, Nematode Culture, and CRISPR mutagenesis

All *C. elegans* strains used in this study were propagated on Nematode Growth Media (NGM) plates seeded with *Escherichia coli* OP50 under standard growth conditions unless otherwise noted [31]. The strains referenced within this study are listed in Table 1. WRM101 and WRM102 were generated using CRISPR-Cas9 mutagenesis following the procedure of Ghanta and Mello [25]. Custom Alt-R modified crRNA guides and single stranded oligonucleotide donor (ssODN) homology directed repair (HDR) templates were designed and ordered using the Integrated DNA Technologies (IDT, Coralville, IA) Alt-R CRISPR HDR design tool on the IDT website (https://www.idtdna.com/pages/tools/alt-r-crispr-hdr-design-tool). All primer, crRNA, and ssODN sequences used for strain generation, PCR confirmation, or sequencing are listed in S1 Table. Strain DG4222 was obtained from the *Caenorhabditis* Genetics Center (CGC, Minneapolis, MN) [26]. This strain was outcrossed three times through the wild-type N2 strain, and the *gfp::tev::3xflag* integration at the *pos-1* locus was confirmed by Sanger sequencing, prior to use in experiments.

To generate strain WRM101, 3x outcrossed DG4222 hermaphrodites were injected with a mixture containing 10 µg/µL S*treptococcus pyogenes* Alt-R modified Cas9 (IDT, Cat. #1081058), 0.4 µg/µL tracrRNA (IDT, Cat. #1073189), 2nmol crRNA (CD.Cas9.BZLP9054.AB and CD.Cas9.BZLP9054.AM) resuspended to 0.4 µg/µL with IDTE buffer (IDT, Cat. #11-01-02-02), 1 µg/µL ssODN (pos-1_ssODN_UTR-Delete), 500 ng/µL pRF4 (*rol-6*) plasmid as an injection marker, and nuclease free water (IDT, Cat. #11-04-02-01). Because two crRNAs were included, 1.4 µL of each was used. WRM102 was generated using the same injection mix with N2 hermaphrodites. Mutants were identified through PCR using primers that span the entire *pos-1* 3′UTR (pos-1_PCR004F and pos-1_PCR005R). Heterozygotes of the mutant alleles were propagated, and progeny were isolated and reconfirmed via PCR to identify homozygotes. Both strains can be maintained as homozygotes at 20°C on NGM plates.

WRM85 was generated using the same CRISPR-Cas9 protocol described above with the following exceptions. One crRNA (CD.HC9.YJVG2684.AB) was used instead of two, and an HDR template DNA containing the mCherry coding sequence was amplified from a plasmid (pCCM953, a gift from Craig Mello, UMass Chan Medical School, Worcester, MA, USA) using primers Linker1_pgl1_HA_F and Linker1_pgl1_HA_R (S1 Table). The PCR product was purified using the Zymo Research DNA Clean & Concentrator kit (Catalog Number D4034, Irvine, CA) prior to use in injections. The genotype of WRM85 was confirmed by PCR and Sanger sequencing before experiments were performed.

WRM105 was generated by crossing DG4222 males with WRM85 hermaphrodites. Upon signs of successful mating, young hermaphrodite F1 cross progeny were isolated and allowed to propagate. F2 progeny were allowed to mature, and fluorescence imaging was used to identify plates where mutants contained both GFP::POS-1 and mCherry::PGL-1. The isolation and fluorescence confirmation process was continued until homozygosity was achieved for both alleles. WRM104 was generated using the same approach, except WRM101 males were crossed with WRM85 hermaphrodites. Both WRM104 and WRM105 can be propagated as homozygotes at 20°C on NGM plates.

### Brood size, hatch rate, and sterility assays

Brood size, hatch rate, and sterility assays were performed for strains DG4222, WRM101, WRM102, WRM104, WRM105, and N2. L1 larval synchronization was achieved by bleaching young adult worms with 20% alkaline hypochlorite solution (3 mL Clorox bleach, 3.75 mL filtered 1M NaOH, 8.25 mL ddH_2_O) to recover embryos, which were extensively washed with M9 buffer and allowed to hatch at room temperature overnight. L1 hatchlings were plated onto NGM agar plates seeded with OP50 *E. coli* and allowed to mature to L4 larvae at 20°C. For each genotype, L4 larvae were isolated onto NGM plates with OP50 and placed under standard growth temperature (20°C) or mild temperature stress (25°C). Each worm was transferred daily to a new NGM plate with OP50, and the number of embryos laid onto the previous day’s plate was counted. Viable F1 progeny that hatched were counted 24 hours after each embryo count. Once larval hatchlings matured to adulthood, approximately 72 hours after larval count, F1 hermaphrodites were scored for the presence (fertile) or absence (sterile) of a uterus containing oocytes and/or embryos. For each strain, this entire assay procedure was repeated in triplicate.

The total number of embryos laid per worm (brood size) was calculated by summing the embryo counts over the course of each worm’s reproductive window. The total viable progeny was calculated by summing the larval counts over the same period. The number of viable progeny was divided by the total brood to determine the hatch rate for each animal. The sterility rate was calculated by dividing the total number of F1 hermaphrodite progeny categorized sterile by the total number of hermaphrodite F1 progeny assessed. Comparisons of the brood size, hatch rate, and sterility rate were made using a one-tailed ANOVA with Bonferroni correction for multiple hypothesis testing using StatPlus software (V8.0.4.0, AnalystSoft, Alexandria, VA). For yes or no assessments concerning the presence of sterile progeny, comparisons between genotypes and growth conditions were analyzed using a Pearson’s Chi-Square test in StatPlus, and post-hoc pairwise p-values were adjusted with Bonferroni correction for multiple hypothesis testing.

### Whole genome sequencing

Worm DNA samples were harvested from two freshly starved medium plates per genotype (WRM101, WRM102, DG4222). Samples were lysed using 200 µL of 1X Worm Lysis Buffer (10 mM Tris-Cl pH 8.0, 50 mM KCl, 2 mM MgCl_2_). DNA was purified from crude lysate using a Qiagen DNeasy blood and tissue kit (Cat. #69504, Germantown, MD) following the manufacturers protocol. DNA samples were analyzed using a QuBit fluorometer (ThermoFisher, Waltham, MA) to determine concentration and quality before being sent to Novogene (Davis, CA) for library construction, sequencing, and data analysis through their whole genome sequencing service. The entire report from Novogene, including quality control, data filtering, and SNP/Indel characterization pipelines, is provided as S1 Data. Briefly, FastP v.0.20.0 was used to assess sequencing quality, BWA v.0.7.17 was used to map the reads to the reference genome (WBcel235 GCF_000002985.6), and SamTools v.1.3.1 was used to map indels and single nucleotide polymorphisms.

### Gonad fluorescence imaging and quantitation

For gonad imaging, synchronized DG4222 and WRM101 larvae were propagated at standard growth temperature (20°C) or under mild temperature stress (25°C) and selected as young adult hermaphrodites for imaging. Worms were mounted onto 2% agarose pads (Fisher BioReagents Cat. #BP160–500) on plain glass slides (Corning Cat. #2947-75x25). A volume of 1 µL of 1 mM levamisole was pipetted onto the pad to paralyze the worms, and an additional 1–2 µL of M9 buffer was added before the cover slip (Fisherbrand Cat. No. 12–542-B). DIC and GFP images of gonads were taken using a Zeiss AxioObserver 7 microscope (Oberkochen, Germany) with a 20X objective. Images collected with identical microscope settings were analyzed using the segmented line tool in Fiji V2.14.0 (ImageJ) software with a 20 µm width to define the average GFP::POS-1 fluorescence intensity profile from the distal tip to the most proximal oocyte, measuring through the center of the gonad while avoiding nuclei in oocytes. A control line of approximately equal length and width was drawn outside of the worm and used as a background control. The background corrected intensity values were binned into 20 equal partitions per gonad and averaged across multiple images of worms with the same genotype and growth conditions. Statistical significance between genotypes and growth conditions was assessed using a one-tailed ANOVA with Bonferroni correction for multiple hypothesis testing using StatPlus software, as above.

### Embryogenesis movies and quantitation

DG4222 and WRM101 worms were propagated at standard growth temperature (20°C) or under mild temperature stress (25°C) for at least one generation before being selected for dissection. Worms were mounted onto 2% agarose pads on plain glass slides in 1–2 µL of levamisole and dissected at the vulva using a 26-gauge needle (BD PrecisionGlide 305110) to release the embryos. A volume of 1–2 µL of M9 buffer is added before application of the coverslip, and then an additional 2–3 µL of M9 was added under the cover slip to prevent the slide from drying out. One-cell embryos were identified under DIC optics, and then DIC and GFP images were collected at five-minute intervals for a total of 90 minutes using a Zeiss AxioObserver 7 microscope with a 63X oil immersion objective (Zeiss Immersion Oil Cat. #444960-0000-000). Focus was maintained with a Zeiss Definite Focus module. All images were collected using identical microscope settings.

The total embryonic fluorescence intensity was measured at each time point using the oval selection tool in Fiji (ImageJ). An oval of equal area was drawn outside of the embryo and was used to determine the background fluorescence. Due to the inability to synchronize fertilization, the first frame where cytokinesis is observed during the first cellular division was arbitrarily set to time point zero. The time axis of all embryos was synchronized to this morphological feature to enable comparison of multiple embryos. The average fluorescence intensity across multiple embryos and multiple movies was plotted as a function of time. A one-tailed ANOVA with Bonferroni correction for multiple hypothesis testing was conducted using StatPlus software to assess statistical significance in intensity between time points.

To define the time point with half maximal fluorescence intensity in strain WRM101, the data were fit to a sigmoid equation using IgorPro software (v9.02, Wavemetrics, Lake Oswego, OR)


$$I=base+\;\left[\text{max }/\;\left(\text{1}+e^{\frac{{(t}_{half}-t}{rate)}}\right)\right]$$ Equation 1

where I is the average fluorescence intensity, t is the time, t_half_ is the time with half maximal fluorescence intensity, rate defines the shape of the sigmoid, and base and max describe the minimal and maximal bounds of the fluorescence intensity. To define the time of peak fluorescence in strain DG4222, the average fluorescence intensity data were fit to a modified Gaussian function using IgorPro software


$$I=base+\text{max}\left(e^{\frac{{-\left(t-t_{max}\right)}^{\text{2}}}{{\text{2}w}^{\text{2}}}}\right)$$ Equation 2

where I is average fluorescence intensity, t is the time, t_max_ is the time with maximal fluorescence intensity, and w is the width of the Gaussian, where w = width of the left tail for t < t_max_, and w = width of the right tail for t > t_max_. In both cases, the fits were weighted by the standard error of the mean for each time point, and the reported error is the error of the fitted parameter.

### Embryonic p-granule imaging and quantitation

WRM104 and WRM105 strains were grown at 20°C for at least one generation before individual worms were dissected and mounted as described above. DIC, mCherry, and GFP images of one, two, and four cell embryos were taken using a Zeiss AxioObserver 7 with a 63X oil immersion objective. To ensure observance of granules at varying z-coordinates, the z-stack feature was used to take 20 images at even slices through each embryo. A maximal z-projection was calculated using Fiji(ImageJ) from the range of slices with visible p-granules, then the total number of p-granules was counted using the multi-point tool. A one-way ANOVA analysis with Bonferroni correction for multiple hypothesis testing was used to compare the p-granule counts between each genotype and embryonic stage using StatPlus, as described above.

### Embryonic germ-cell progenitor imaging and quantitation

Embryos from WRM104 and WRM105 strains were harvested and mounted as described above. DIC, mCherry, and GFP images of embryos were acquired using a Zeiss AxioObserver 7 as described above. Images of the embryos for each genotype were binned by the approximate cell count and/or developmental stage into the following categories: 2–32 cells, 33–200 + cells, or bean through pretzel stages. The number of germ cell progenitors was determined by counting the number of cells expressing mCherry::PGL-1. The data sets for each genotype and each bin were compared using a Pearson’s Chi-Square test with Bonferroni correction for multiple hypothesis testing of post-hoc pairwise comparisons using StatPlus as described above.

### Larvae gonad length imaging and quantitation

WRM104 and WRM105 embryos were synchronized by bleaching as described above. Synchronized L1 worms were plated onto NGM agar plates seeded with *E. coli* OP50. DIC, GFP, and mCherry images were taken for L1 through L4 larval stages using a 40X oil immersion objective on a Zeiss AxioObserver 7 microscope. L1 larvae were imaged between 0–8 hours after plating. Because L1 larvae have yet to begin germ cell proliferation, the quantity of germ cell progenitors was counted rather than the length of the gonad. The subsequent L2 and L3 larval stages were imaged 20–24 hours after plating. Differentiation between each stage was based on the presence or absence of separation of the gonad arms, indicating completion of the L2 to L3 molt. Analysis of gonad length was conducted using the segmented line tool in Fiji (ImageJ) software as described above. To measure the length, a line was drawn from the distal to the proximal end of the gonad using PGL-1 expression as a guide. From the L3 larval stage and onwards, each gonad arm length was measured separately unless a gonad arm was absent (no value recorded). L4 larvae were imaged 40–48 hours after plating and identified based on the observance of the characteristic square vulval lumen. Young adult hermaphrodites were selected at random and imaged 65–70 hours after plating. Young adult gonads were too large to properly fit in frame using the 40X objective, thus the 20X objective was utilized for this developmental stage. If gonads were fragmented, the length of each fragment was summed to avoid erroneous overmeasurement of gaps. We found that taking Z-stack images helped define the distal and proximal ends of each gonad for all stages except L1. Statistical significance between comparison groups was determined using a one-way ANOVA test with Bonferroni correction for multiple hypothesis testing in StatPlus software.

### RNA sequencing

Five medium plates (60mm x 15mm petri dish seeded with 200 µL of OP50 bacteria) of synchronized young adult stage worms were harvested using 1 mL ddH_2_O per plate and combined into a 15 mL conical tube. Tubes were spun in a room temperature centrifuge for 1 minute at 3000 rpm (2100 x g), and pellets were washed with 3 mL ddH_2_O four more times. Pellets from each replicate were aggregated into one 1.7 mL tube each then washed once more to remove any remaining contaminating OP50 (960 x g). Each tube was flash frozen in liquid nitrogen to lyse the worms. Total RNA was extracted using eight pellet volumes of TRIzol, followed by 1.6 volumes of chloroform. The aqueous phase was extracted, and an equal volume of isopropanol was added to precipitate the RNA. Extraction was followed by rRNA depletion using a *C. elegans* optimized protocol [52]. Sample libraries were prepared using the NEBNext Ultra II library prep kit (E7775S) and barcoded using the NEBNext Multiplex Oligos for Illumina (Dual Index Primer Set 1 (E7335S) & Set 2 (E7500S)). Sample concentration was calculated using a Qubit Fluorometer and Fragment analyzer prior to data collection on an Illumina NEXTSeq 1000 (Illumina, San Diego, CA). In total, data from three biological replicates per genotype were collected.

The resulting raw sequencing reads were submitted to the OneStopRNASeq pipeline for quality control and differential gene expression analysis [53]. FastQC v.0.11.5 was used for raw sequencing quality control, and MultiQC v.1.6 was used post-alignment quality control using the default configurations in the OneStopRNASeq pipeline. STAR v2.7.5a was used to align the reads to the reference genome using WBcel235.90 annotations using the following parameters ‘-Q 20 –minOverlap 1 --fracOverlap 0 -p -B -C’ for paired-end strict-mode analysis [54]. Differential expression (DE) analysis was performed with DESeq2 v1.28.1 [37]. Significantly differentially expressed genes were filtered with the criteria FDR < 0.05, absolute log2 fold change (|LFC|) > 0.585, and Base Mean expression level of >100.

## Supporting information

S1 Fig Confirmation of the strain background by whole genome resequencing. **A.** A genome viewer image of the *pos-1* locus with pileup views for GFP-ΔUTR, GFP-WT UTR, and untagged N2-ΔUTR whole genome sequencing data. The brackets show the location of the 3′UTR deletion and the point of insertion of the *gfp::tev::3xflag* tag. The vertical red stripe is a silent mutation in the *pos-1* gene that removes a PAM site. **B.** Venn diagram of the overlap between exonic SNP calls from the whole genome sequencing data. The number for the six unique GFP-ΔUTR SNPs is listed in gray because visual inspection of the sequencing tracks in a genome data browser shows that all six alleles are also present in the GFP-WT UTR strain at an allele frequency >0.5, with the exception of *Y22D7AR.2* which has an allele frequency of <0.5, as described in the text. These represent false negatives in the bioinformatic pipeline that likely correspond to the allele frequency cutoff. **C.** Venn diagram of exonic indel alleles in the whole genome sequencing data. The lone candidate allele in the GFP-ΔUTR strain is listed in gray because it was also found in the untagged N2-ΔUTR strain by visual inspection of the genome sequencing tracks.
(TIFF)

S2 Fig Quantitation of p-granules in young embryos. **A.** DIC, GFP, and mCherry images of mCherry::PGL-1 marked GFP-WT UTR and GFP-∆UTR embryos at the two-cell stage. Scale bars: 10 μm. **B.** Box-and-Whisker plot representing the number of p-granules present within an individual embryo at the specified cell stage. Each dot represents the total number of p-granules counted for an embryo. Orange triangles indicate outliers as in Fig 2C. Red squares indicate far outliers as in Fig 2B.
(TIFF)

S3 Fig Alignment of 3′UTR sequences of *pos-1* homologs in other *Caenorhabditis* species. MAFFT (Multiple Alignment using Fast Fourier Transform) alignment of *pos-1* homologs in *C. brenneri* (*pos-1.1* & *pos-1.2*), *C. briggsae* (*cbr-pos-1* & *CBG23753*), and *C. remanei* (*cre-pos-1*). Sequences were recovered from WormBase and aligned using MAFFT (https://mafft.cbrc.jp/alignment/server/index.html) [55,56]. Conserved regions were rendered using pyBoxshade (https://github.com/mdbaron42/). Manual annotation of GLD-1, FBF-1, and POS-1 binding motifs were masked as colored boxes onto the alignment rendering.
(TIFF)

S1 Movie Time lapse images of GFP-WT UTR and GFP-ΔUTR embryos are presented. Each frame represents five minutes of elapsed time. The scale bar represents 5 microns. The growth temperature was 20ºC. DIC and GFP images for both strains are presented side by side.
(MOV)

S2 Movie The time lapse images shown here are labeled as in S1 Movie. The only difference is that the animals were grown at 25ºC.
(MOV)

S1 Table Primers and Oligonucleotides used in this study. (DOCX)

S1 Data All bioinformatic output from the whole genome sequencing data described in S1 Fig. The data set comprises a zipped directory that includes informatics reports in html format, excel spreadsheets, and text files. (GZ)

S2 Data All bioinformatic output from the One Stop RNA Seq and Wormcat pipeline described in Fig 7. The data set comprises a zipped directory that includes informatics reports in html format, excel spreadsheets, graphics files in PDF and SVG format, and text files. (GZ)

S3 Data An excel spreadsheet that contains all numerical data and statistical analyses for Figs 2 through Figs 6 and S2. (XLSX)
