# Supplementary figures and images for: The pos-1 3′ untranslated region governs germline specification and proliferation to ensure reproductive robustness (part 1 of 2)
Source: PLoS Genet. 2026 Apr 27;22(4):e1012129. doi: 10.1371/journal.pgen.1012129 (PMC13132445; doi:10.1371/journal.pgen.1012129)

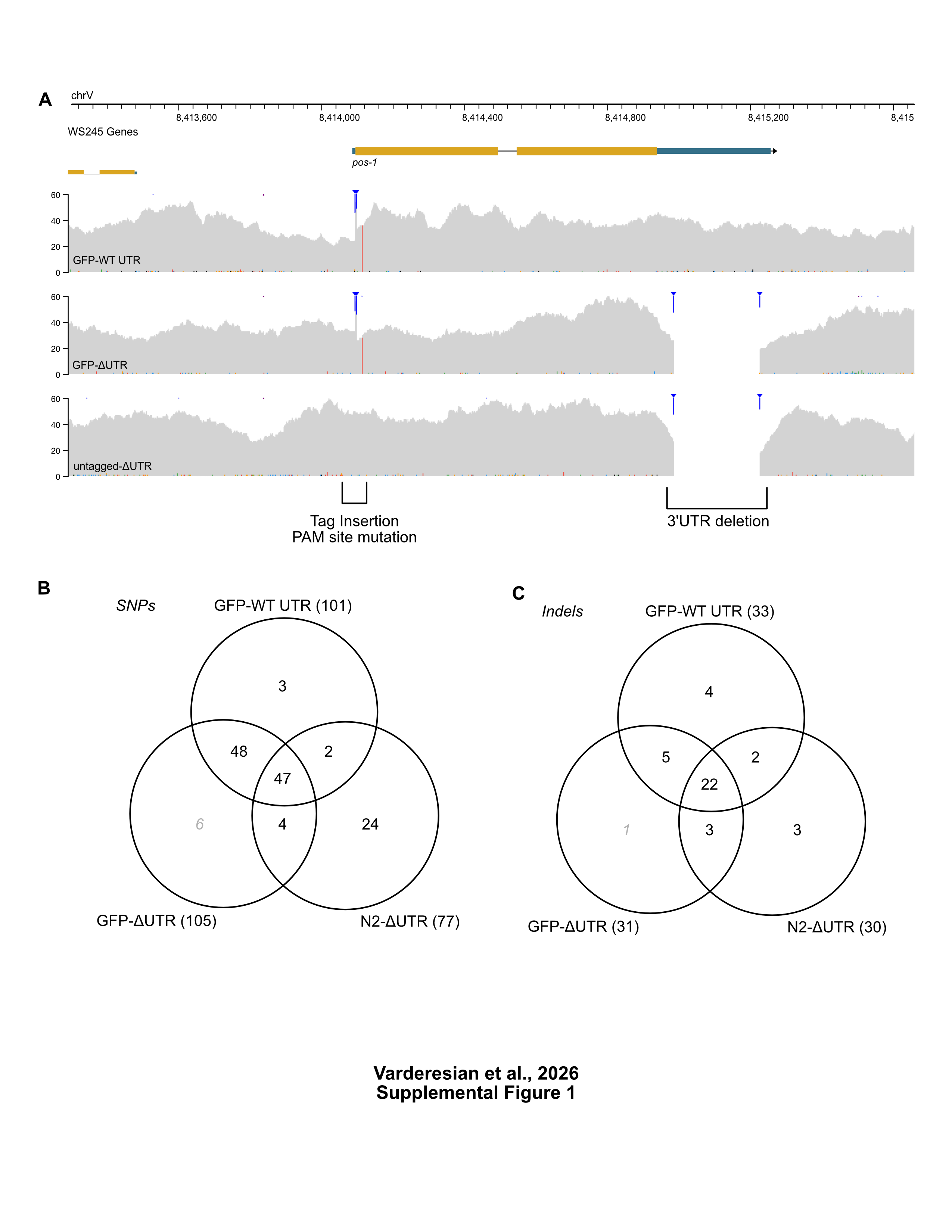

Supplement: S1 Fig — A. A genome viewer image of the pos-1 locus with pileup views for GFP-ΔUTR, GFP-WT UTR, and untagged N2-ΔUTR whole genome sequencing data. The brackets show the location of the 3′UTR deletion and the point of insertion of the gfp::tev::3xflag tag. The vertical red stripe is a silent mutation in the pos-1 gene that removes a PAM site. B. Venn diagram of the overlap between exonic SNP calls from the whole genome sequencing data. The number for the six unique GFP-ΔUTR SNPs is listed in gray because visual inspection of the sequencing tracks in a genome data browser shows that all six alleles are also present in the GFP-WT UTR strain at an allele frequency >0.5, with the exception of Y22D7AR.2 which has an allele frequency of <0.5, as described in the text. These represent false negatives in the bioinformatic pipeline that likely correspond to the allele frequency cutoff. C. Venn diagram of exonic indel alleles in the whole genome sequencing data. The lone candidate allele in the GFP-ΔUTR strain is listed in gray because it was also found in the untagged N2-ΔUTR strain by visual inspection of the genome sequencing tracks. (TIFF) [file pgen.1012129.s001.tiff]

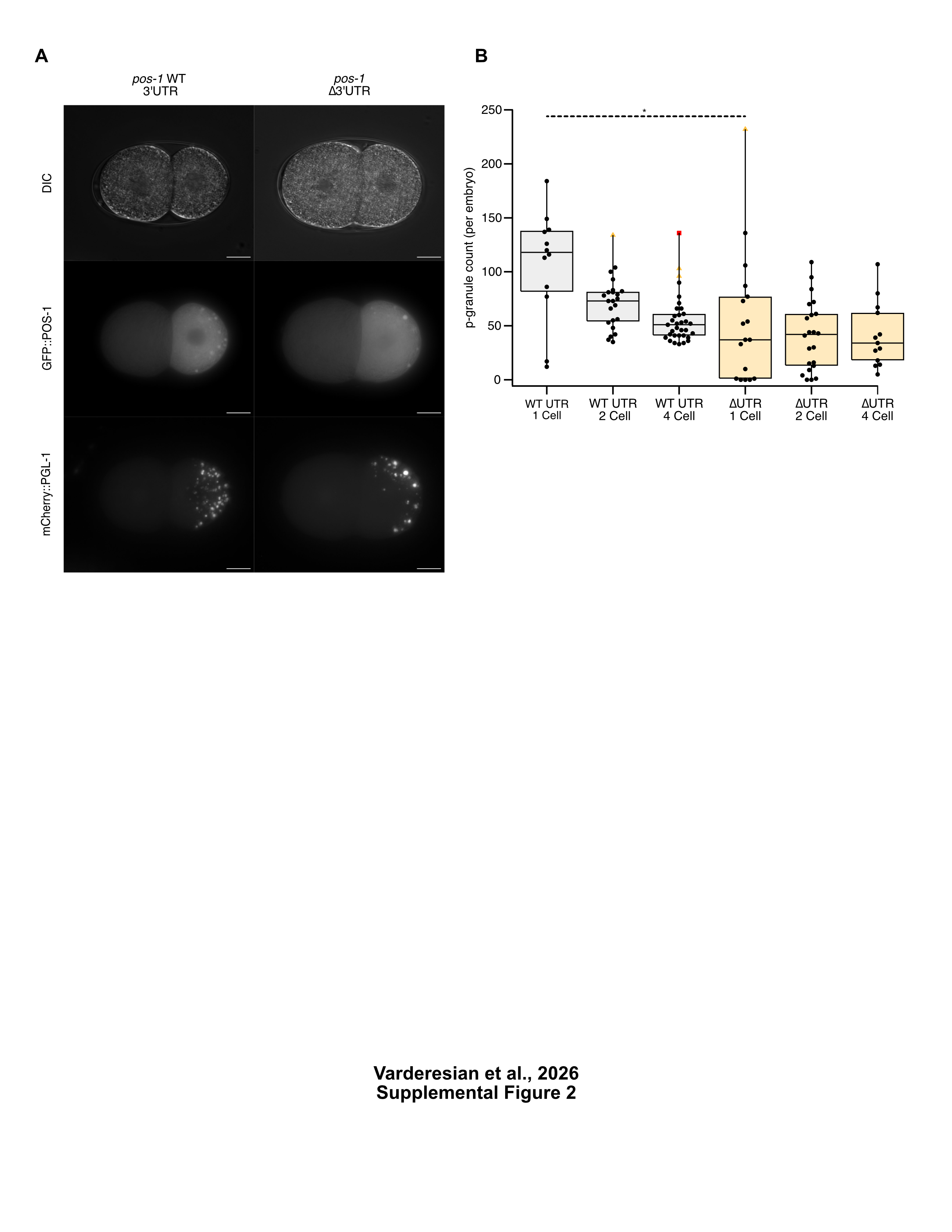

Supplement: S2 Fig — A. DIC, GFP, and mCherry images of mCherry::PGL-1 marked GFP-WT UTR and GFP-∆UTR embryos at the two-cell stage. Scale bars: 10 μm. B. Box-and-Whisker plot representing the number of p-granules present within an individual embryo at the specified cell stage. Each dot represents the total number of p-granules counted for an embryo. Orange triangles indicate outliers as in Fig 2C. Red squares indicate far outliers as in Fig 2B. (TIFF) [file pgen.1012129.s002.tiff]

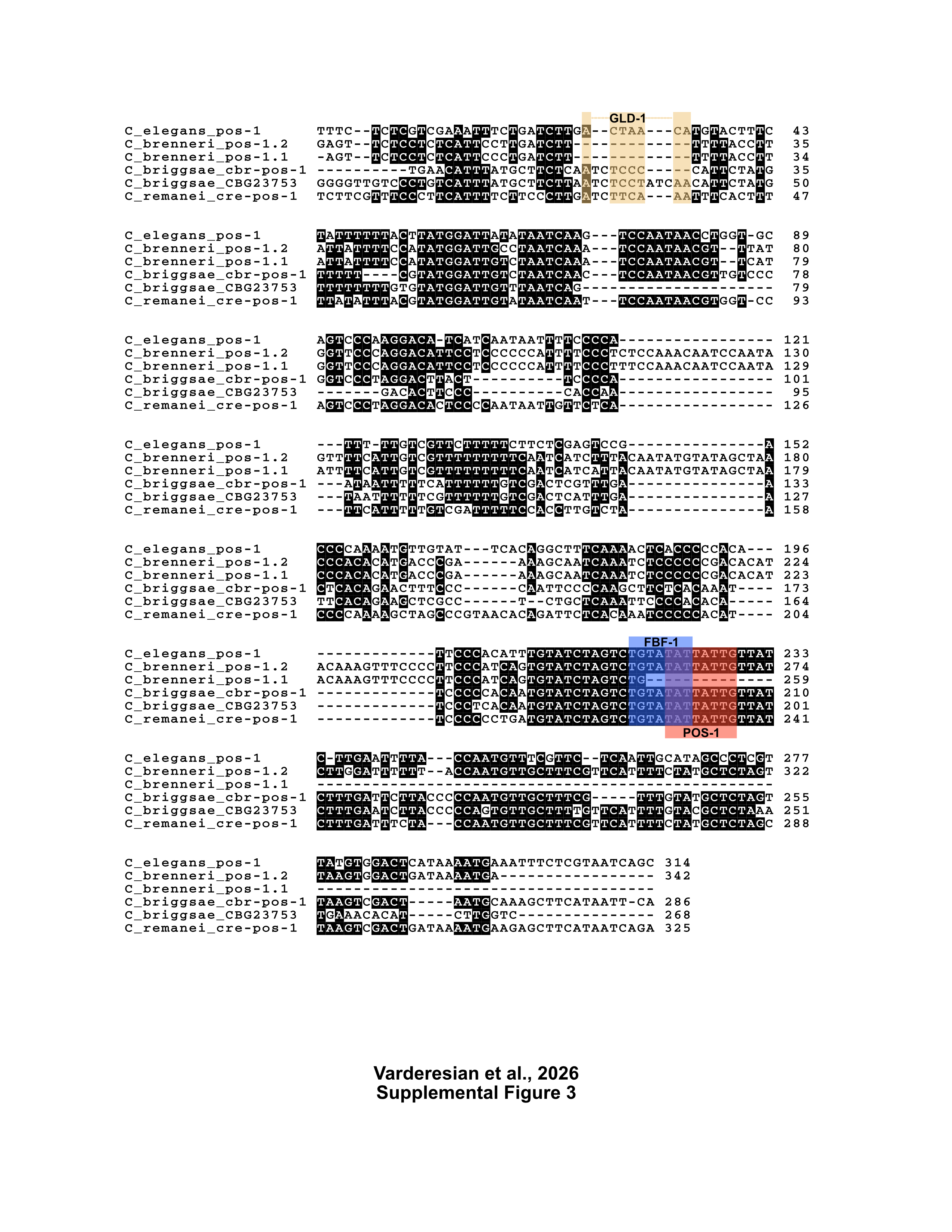

Supplement: S3 Fig — MAFFT (Multiple Alignment using Fast Fourier Transform) alignment of pos-1 homologs in C. brenneri (pos-1.1 & pos-1.2), C. briggsae (cbr-pos-1 & CBG23753), and C. remanei (cre-pos-1). Sequences were recovered from WormBase and aligned using MAFFT (https://mafft.cbrc.jp/alignment/server/index.html) [55,56]. Conserved regions were rendered using pyBoxshade (https://github.com/mdbaron42/). Manual annotation of GLD-1, FBF-1, and POS-1 binding motifs were masked as colored boxes onto the alignment rendering. (TIFF) [file pgen.1012129.s003.tiff]

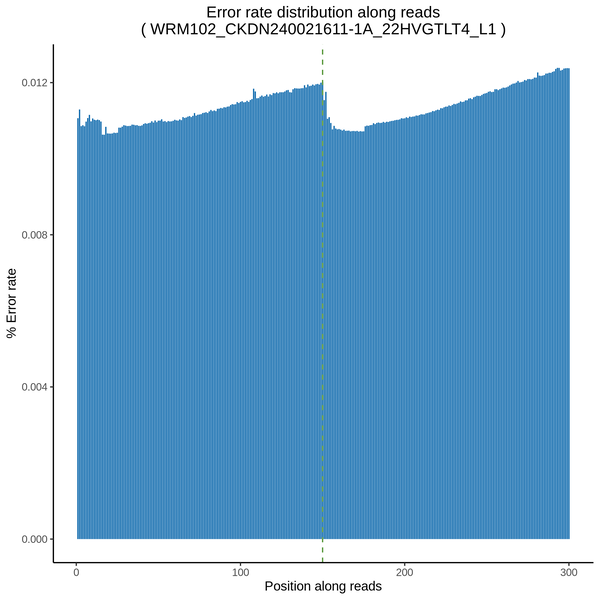

Supplement: S2 Data — (GZ) [file pgen.1012129.s008.gz › SupplementalDataSet1/03.Result_X202SC24112711-Z01-F001_C_elegans/report/src/pictures/Error/WRM102_CKDN240021611-1A_22HVGTLT4_L1.Error.png]

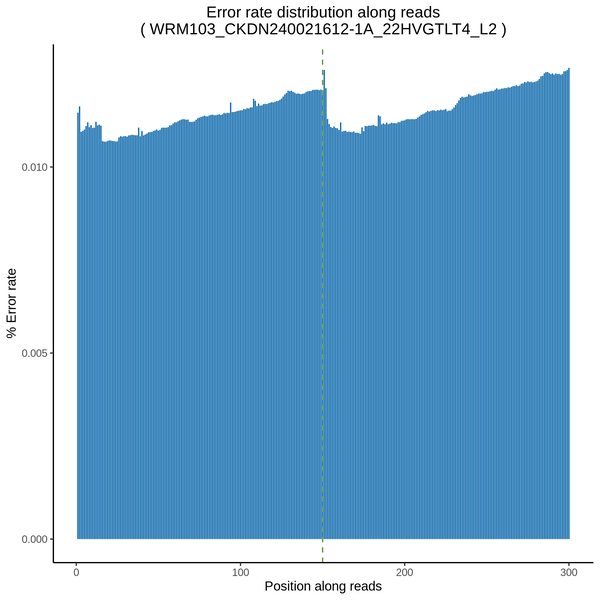

Supplement: S2 Data — (GZ) [file pgen.1012129.s008.gz › SupplementalDataSet1/03.Result_X202SC24112711-Z01-F001_C_elegans/report/src/pictures/Error/WRM103_CKDN240021612-1A_22HVGTLT4_L2.Error.png]

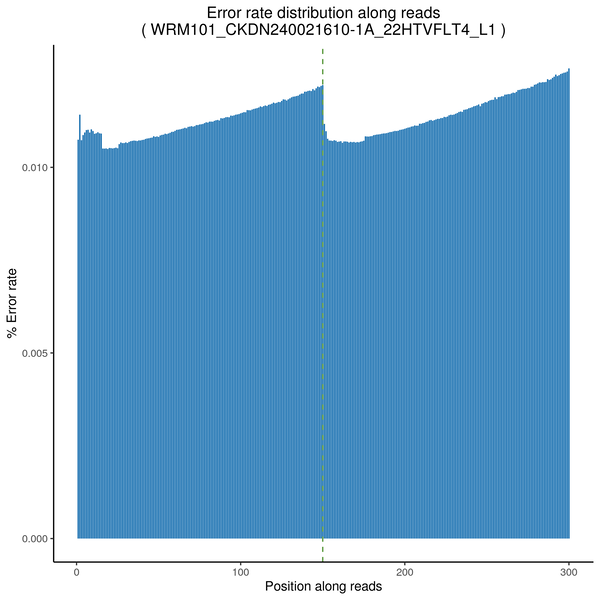

Supplement: S2 Data — (GZ) [file pgen.1012129.s008.gz › SupplementalDataSet1/03.Result_X202SC24112711-Z01-F001_C_elegans/report/src/pictures/Error/WRM101_CKDN240021610-1A_22HTVFLT4_L1.Error.JPEG]

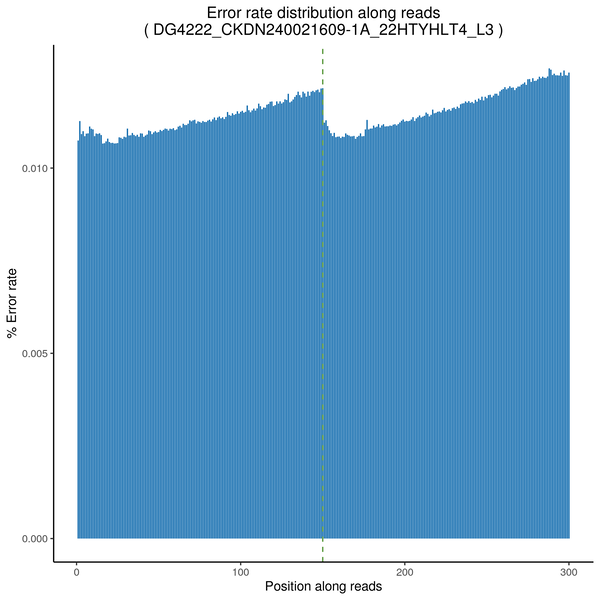

Supplement: S2 Data — (GZ) [file pgen.1012129.s008.gz › SupplementalDataSet1/03.Result_X202SC24112711-Z01-F001_C_elegans/report/src/pictures/Error/DG4222_CKDN240021609-1A_22HTYHLT4_L3.Error.JPEG]

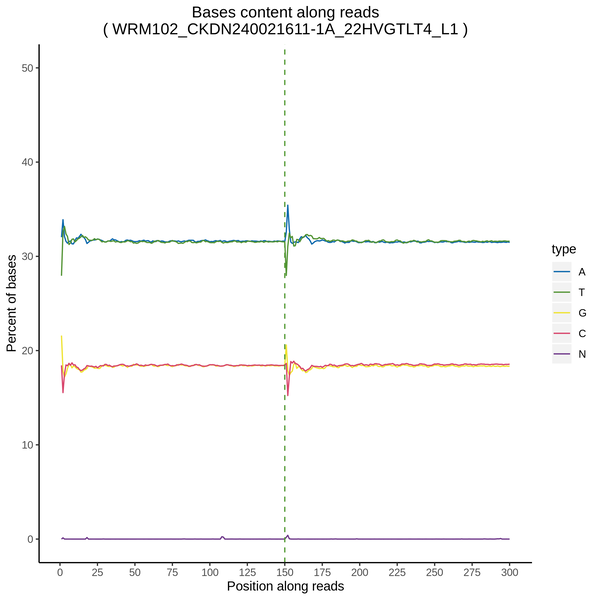

Supplement: S2 Data — (GZ) [file pgen.1012129.s008.gz › SupplementalDataSet1/03.Result_X202SC24112711-Z01-F001_C_elegans/report/src/pictures/GC/WRM102_CKDN240021611-1A_22HVGTLT4_L1.GC.png]

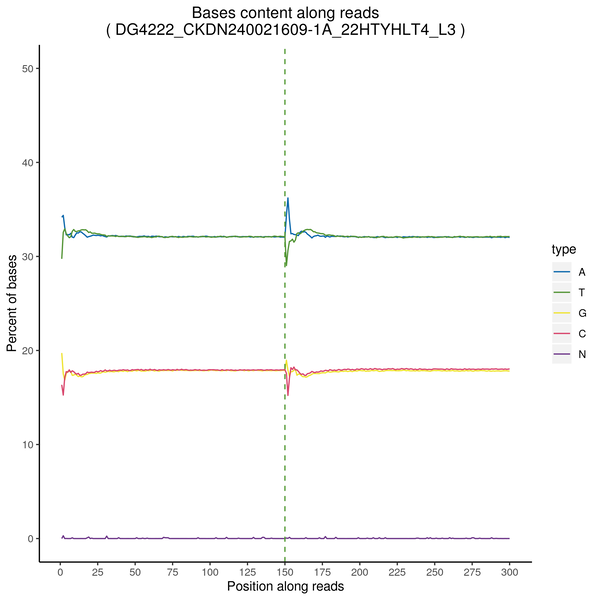

Supplement: S2 Data — (GZ) [file pgen.1012129.s008.gz › SupplementalDataSet1/03.Result_X202SC24112711-Z01-F001_C_elegans/report/src/pictures/GC/DG4222_CKDN240021609-1A_22HTYHLT4_L3.GC.png]

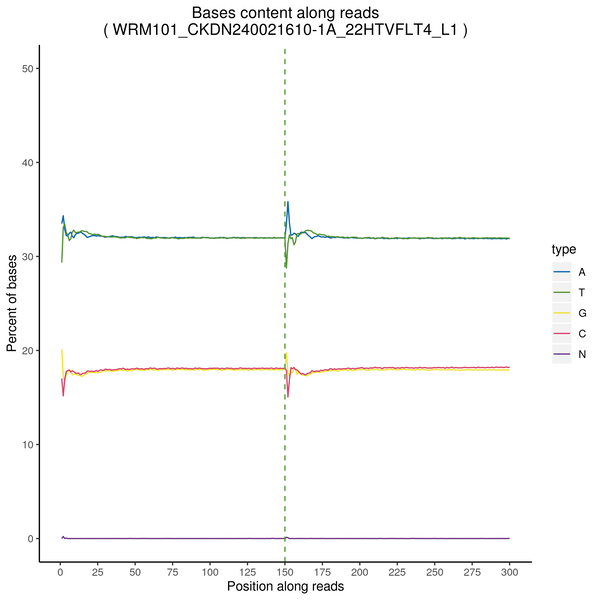

Supplement: S2 Data — (GZ) [file pgen.1012129.s008.gz › SupplementalDataSet1/03.Result_X202SC24112711-Z01-F001_C_elegans/report/src/pictures/GC/WRM101_CKDN240021610-1A_22HTVFLT4_L1.GC.JPEG]

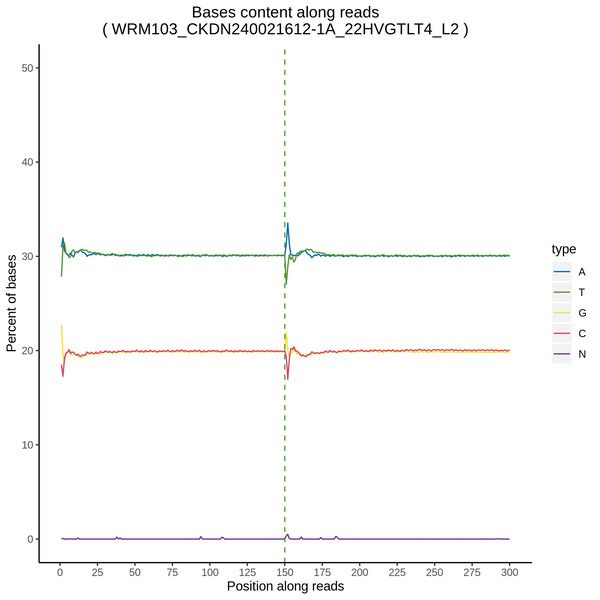

Supplement: S2 Data — (GZ) [file pgen.1012129.s008.gz › SupplementalDataSet1/03.Result_X202SC24112711-Z01-F001_C_elegans/report/src/pictures/GC/WRM103_CKDN240021612-1A_22HVGTLT4_L2.GC.JPEG]

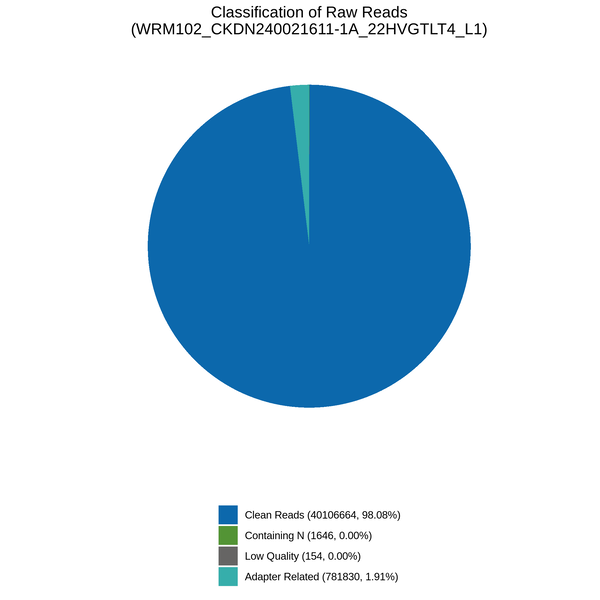

Supplement: S2 Data — (GZ) [file pgen.1012129.s008.gz › SupplementalDataSet1/03.Result_X202SC24112711-Z01-F001_C_elegans/report/src/pictures/Class/WRM102_CKDN240021611-1A_22HVGTLT4_L1.pie3d.png]

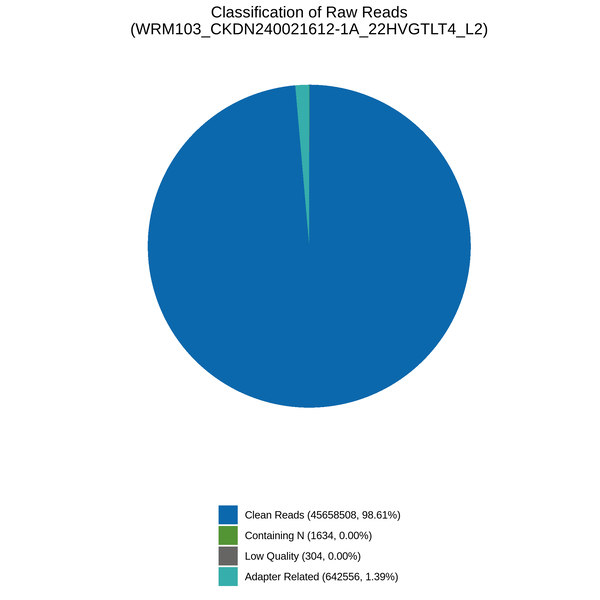

Supplement: S2 Data — (GZ) [file pgen.1012129.s008.gz › SupplementalDataSet1/03.Result_X202SC24112711-Z01-F001_C_elegans/report/src/pictures/Class/WRM103_CKDN240021612-1A_22HVGTLT4_L2.pie3d.png]

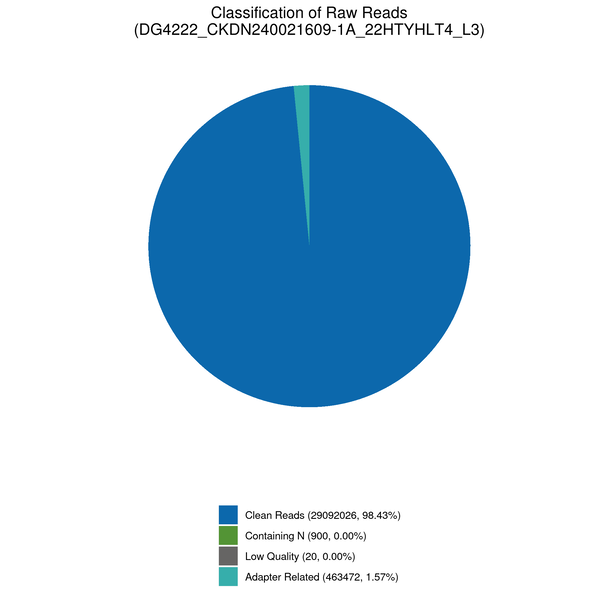

Supplement: S2 Data — (GZ) [file pgen.1012129.s008.gz › SupplementalDataSet1/03.Result_X202SC24112711-Z01-F001_C_elegans/report/src/pictures/Class/DG4222_CKDN240021609-1A_22HTYHLT4_L3.pie3d.png]

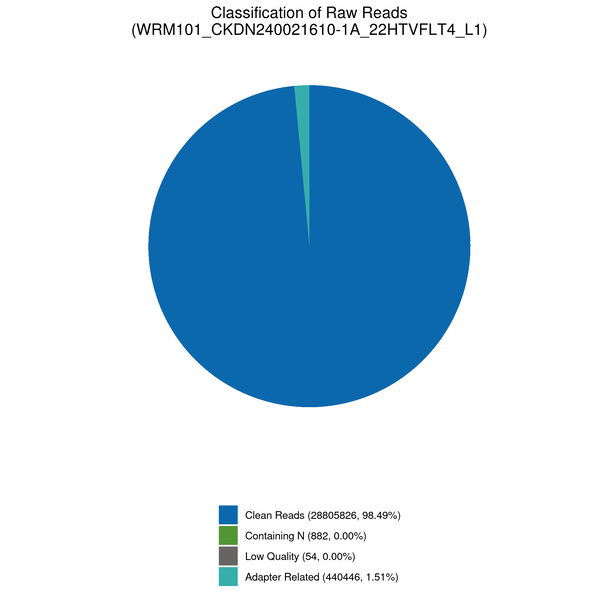

Supplement: S2 Data — (GZ) [file pgen.1012129.s008.gz › SupplementalDataSet1/03.Result_X202SC24112711-Z01-F001_C_elegans/report/src/pictures/Class/WRM101_CKDN240021610-1A_22HTVFLT4_L1.pie3d.png]

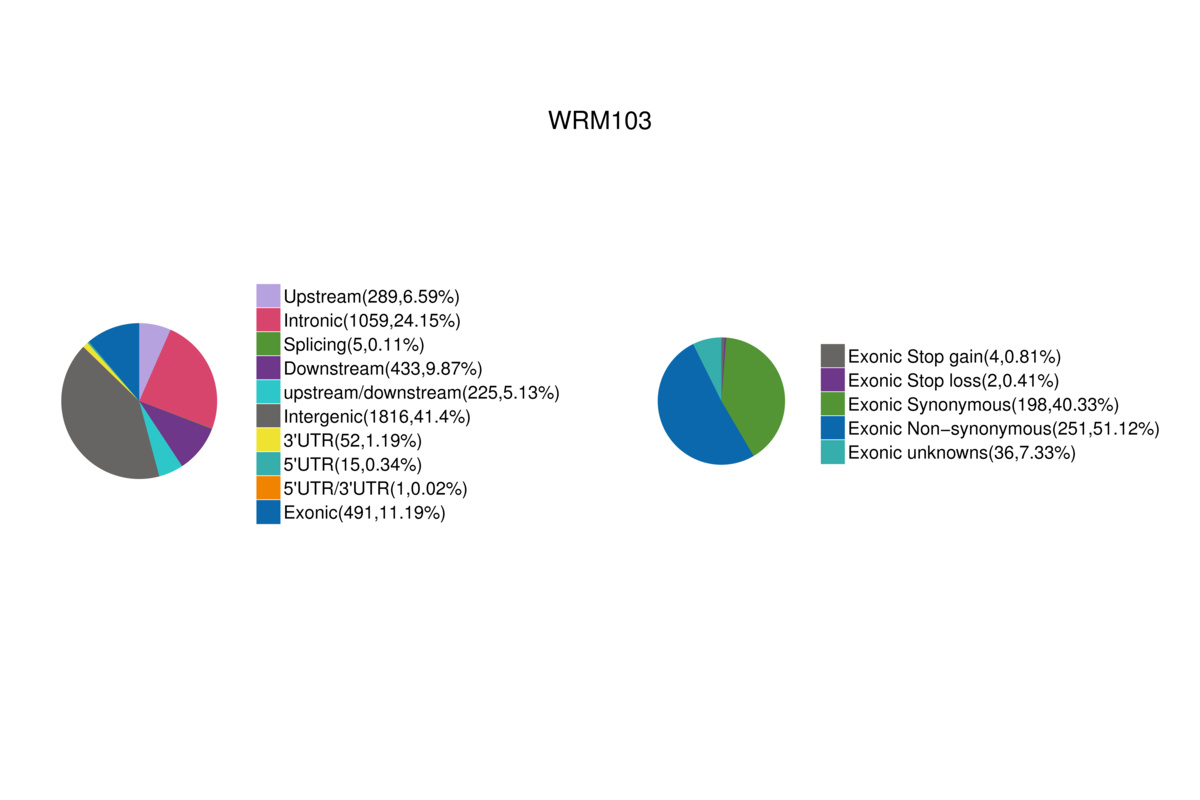

Supplement: S2 Data — (GZ) [file pgen.1012129.s008.gz › SupplementalDataSet1/03.Result_X202SC24112711-Z01-F001_C_elegans/report/src/pictures/SNP/WRM103.SNP.table.png]

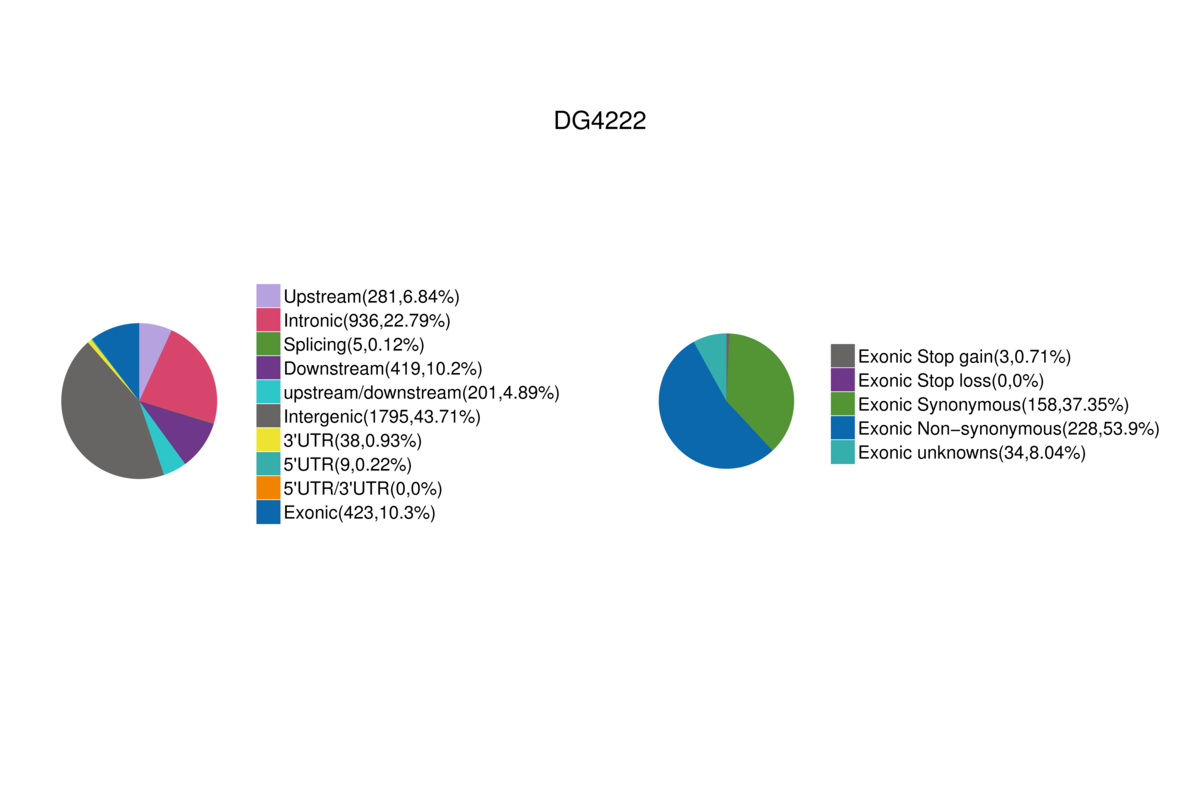

Supplement: S2 Data — (GZ) [file pgen.1012129.s008.gz › SupplementalDataSet1/03.Result_X202SC24112711-Z01-F001_C_elegans/report/src/pictures/SNP/DG4222.SNP.table.png]

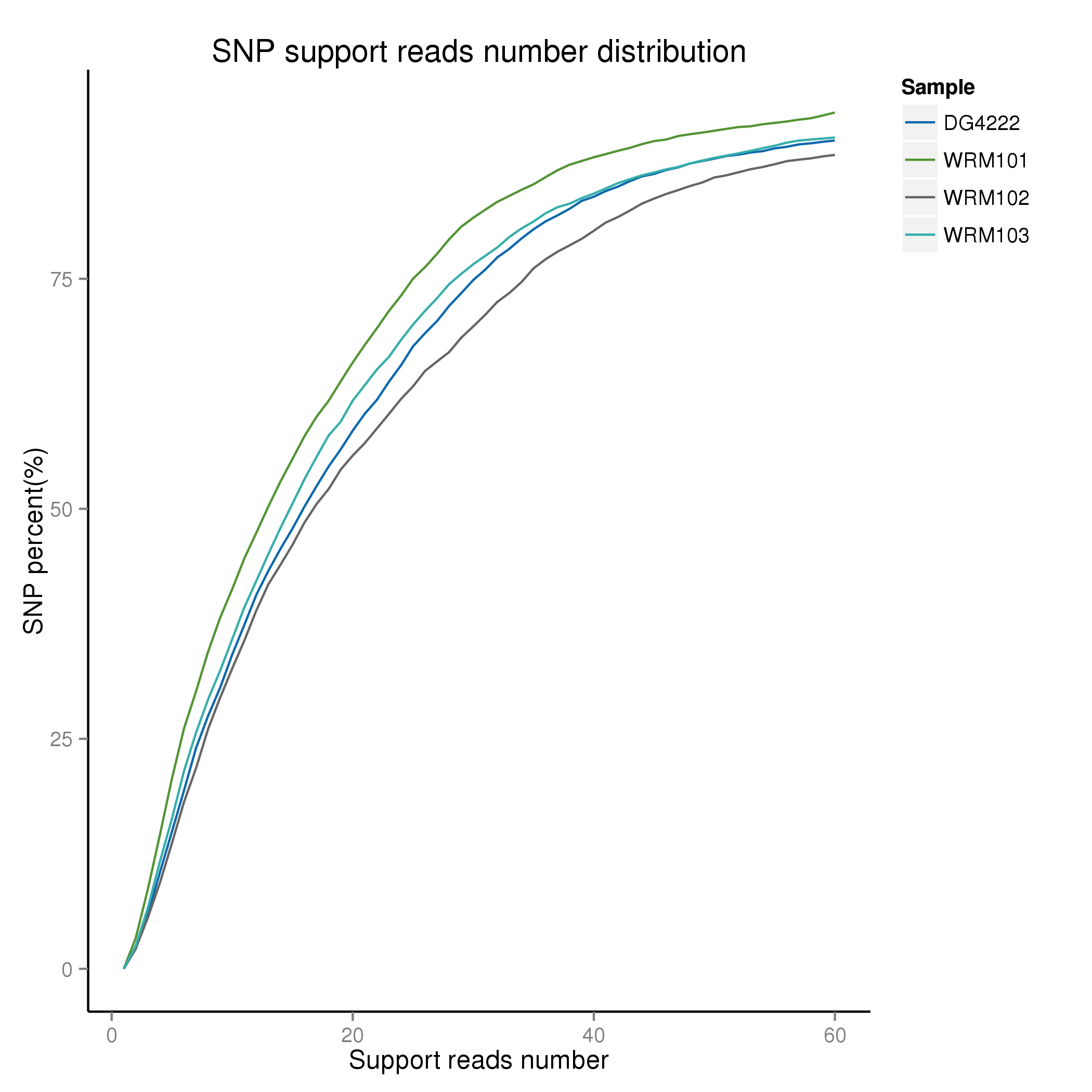

Supplement: S2 Data — (GZ) [file pgen.1012129.s008.gz › SupplementalDataSet1/03.Result_X202SC24112711-Z01-F001_C_elegans/report/src/pictures/SNP/SNP_readsNum_cumulative_distribution.JPEG]

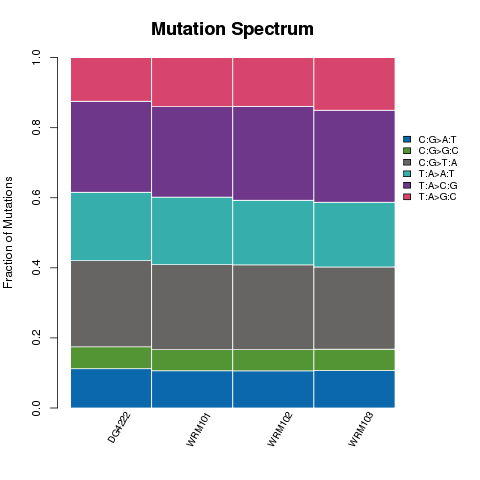

Supplement: S2 Data — (GZ) [file pgen.1012129.s008.gz › SupplementalDataSet1/03.Result_X202SC24112711-Z01-F001_C_elegans/report/src/pictures/SNP/SNP.frequency.xls.barplot.png]

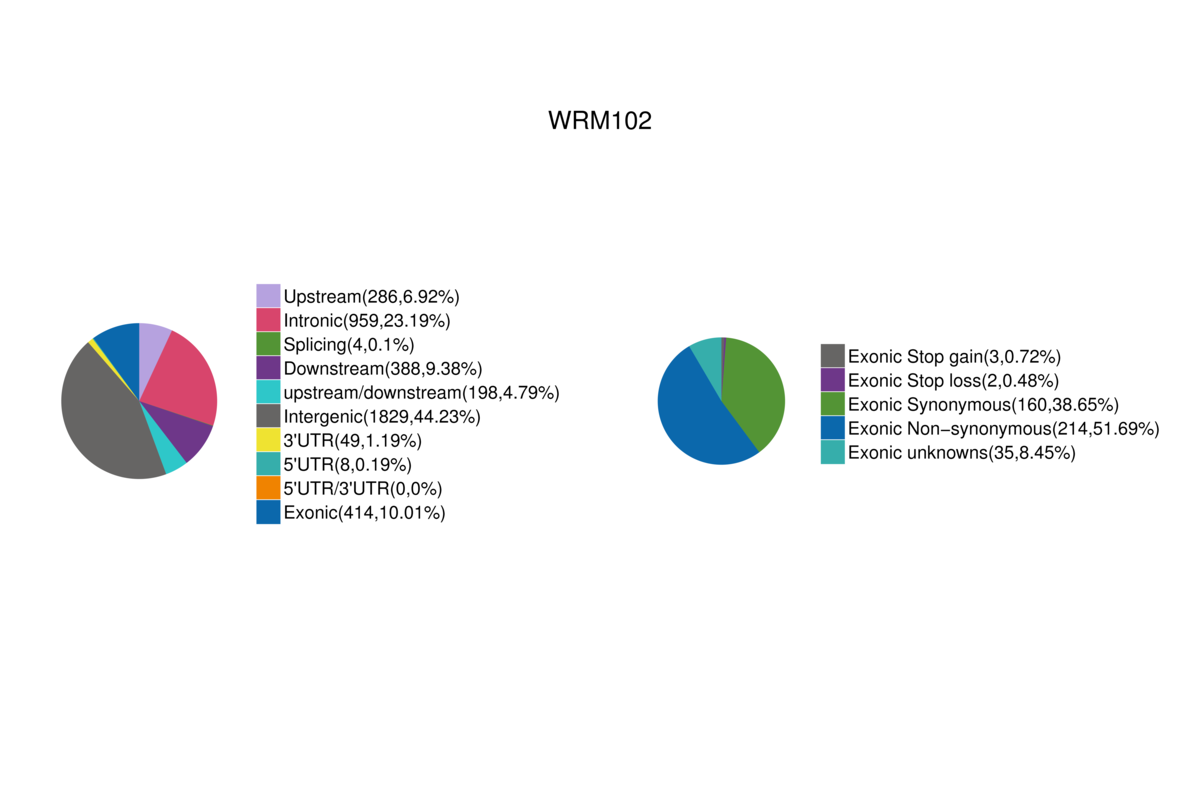

Supplement: S2 Data — (GZ) [file pgen.1012129.s008.gz › SupplementalDataSet1/03.Result_X202SC24112711-Z01-F001_C_elegans/report/src/pictures/SNP/WRM102.SNP.table.png]

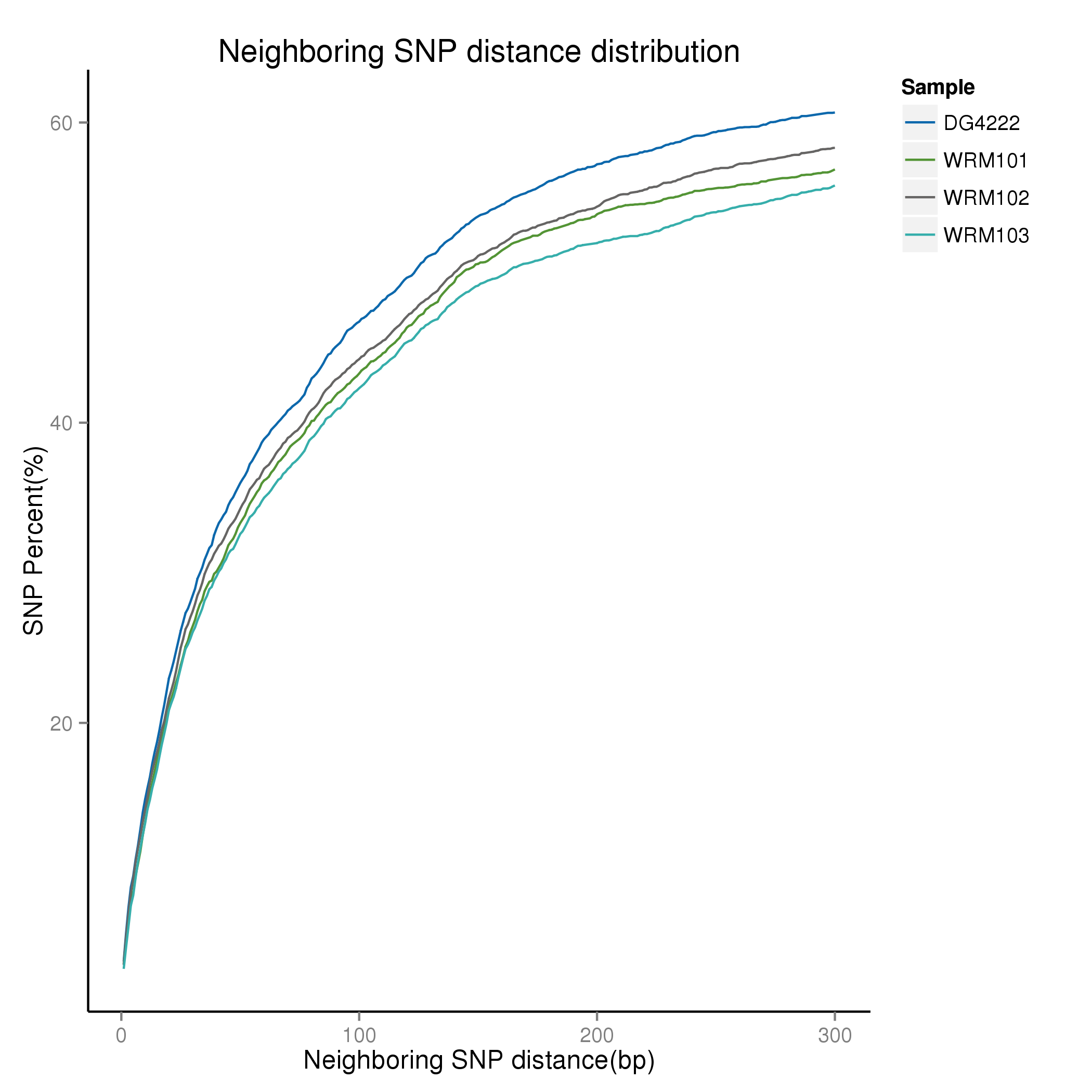

Supplement: S2 Data — (GZ) [file pgen.1012129.s008.gz › SupplementalDataSet1/03.Result_X202SC24112711-Z01-F001_C_elegans/report/src/pictures/SNP/SNP_distance_cumulative_distribution.JPEG]

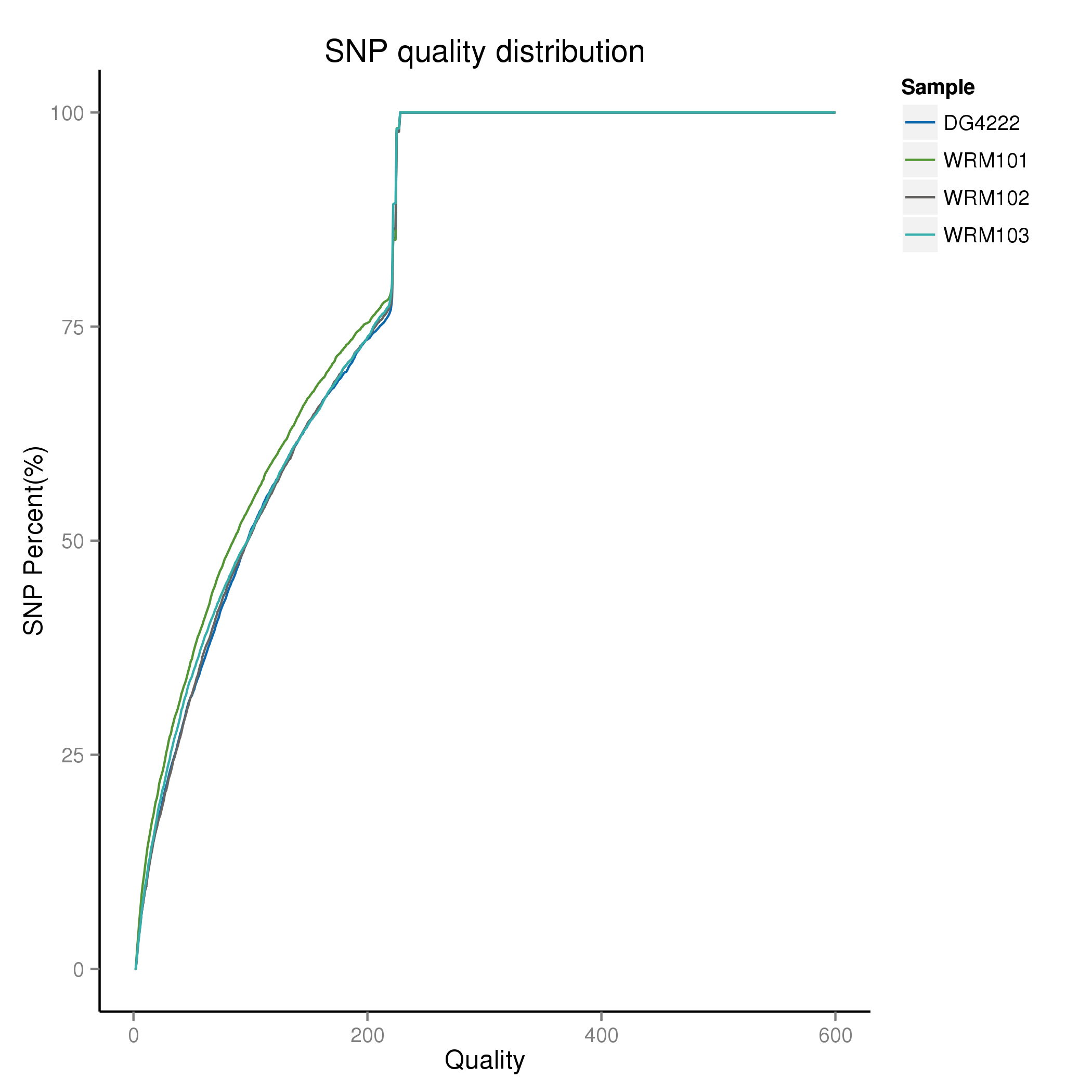

Supplement: S2 Data — (GZ) [file pgen.1012129.s008.gz › SupplementalDataSet1/03.Result_X202SC24112711-Z01-F001_C_elegans/report/src/pictures/SNP/SNP_quality_cumulative_distribution.JPEG]

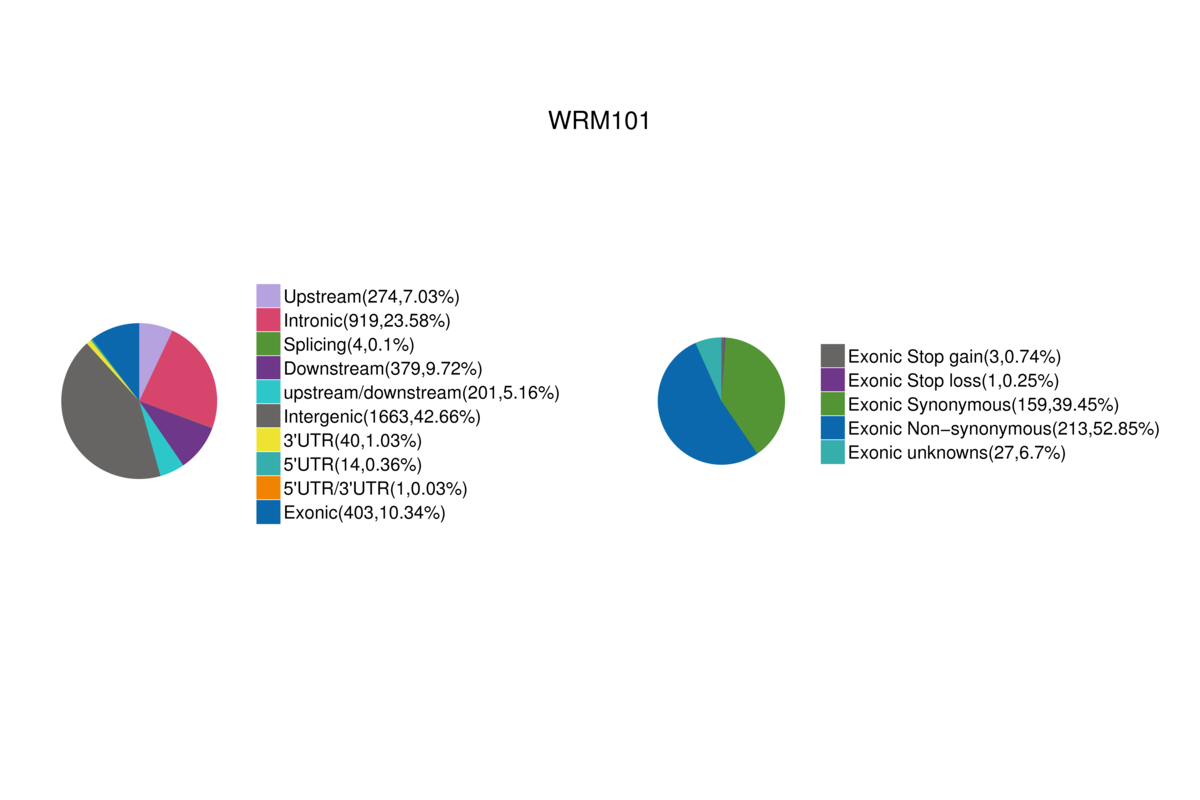

Supplement: S2 Data — (GZ) [file pgen.1012129.s008.gz › SupplementalDataSet1/03.Result_X202SC24112711-Z01-F001_C_elegans/report/src/pictures/SNP/WRM101.SNP.table.png]

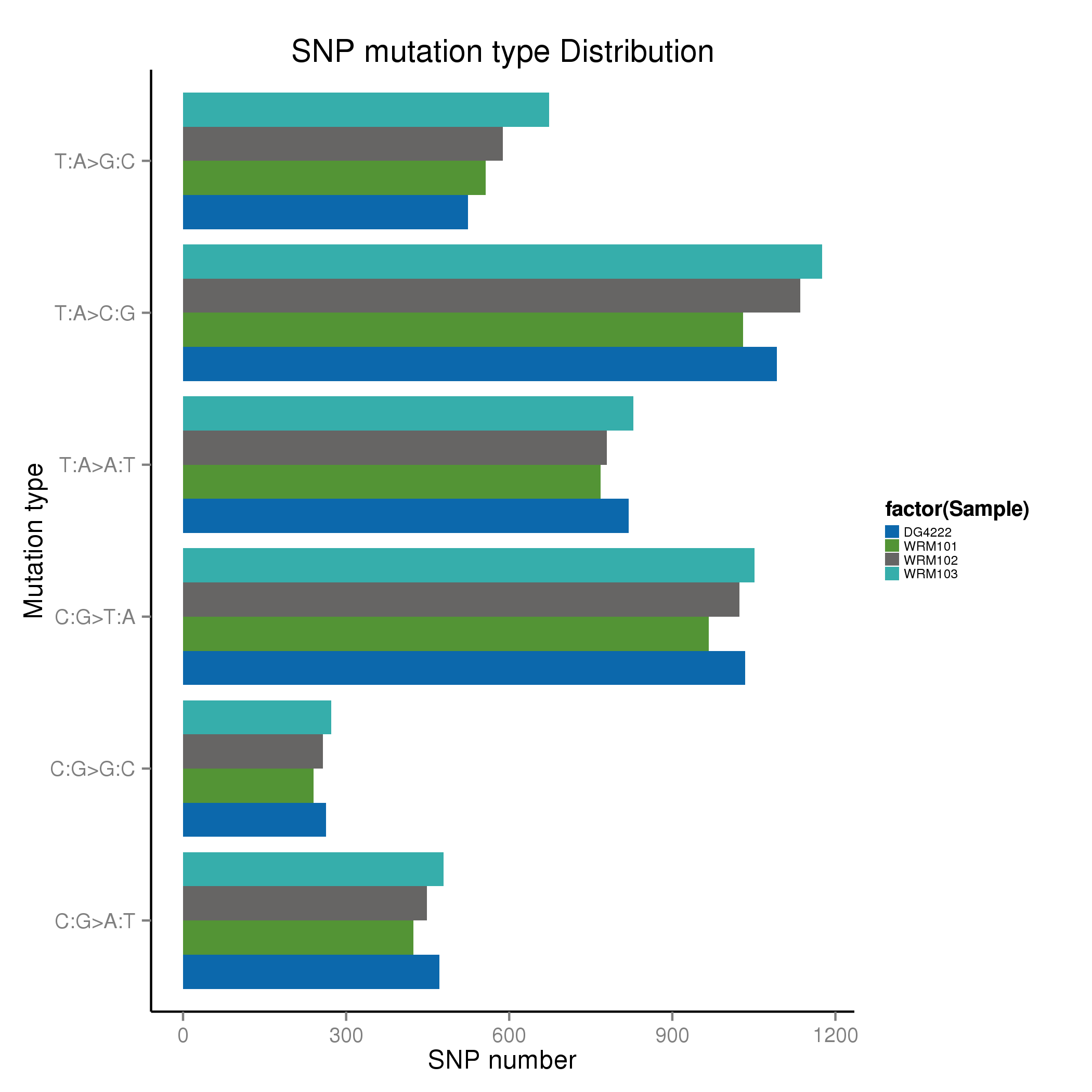

Supplement: S2 Data — (GZ) [file pgen.1012129.s008.gz › SupplementalDataSet1/03.Result_X202SC24112711-Z01-F001_C_elegans/report/src/pictures/SNP/SNP_frequency.png]

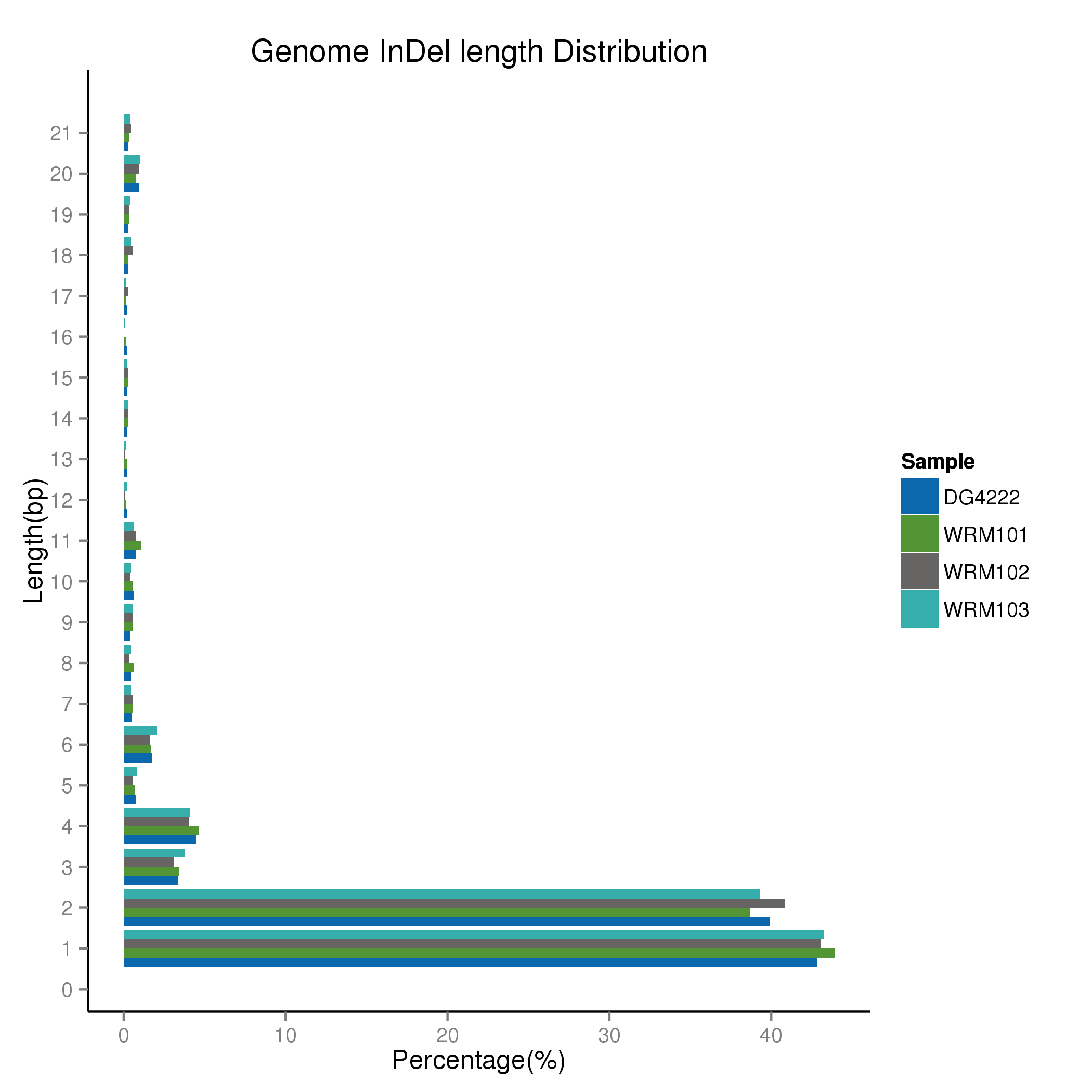

Supplement: S2 Data — (GZ) [file pgen.1012129.s008.gz › SupplementalDataSet1/03.Result_X202SC24112711-Z01-F001_C_elegans/report/src/pictures/InDel/InDel_GENOMEpercentage.png]

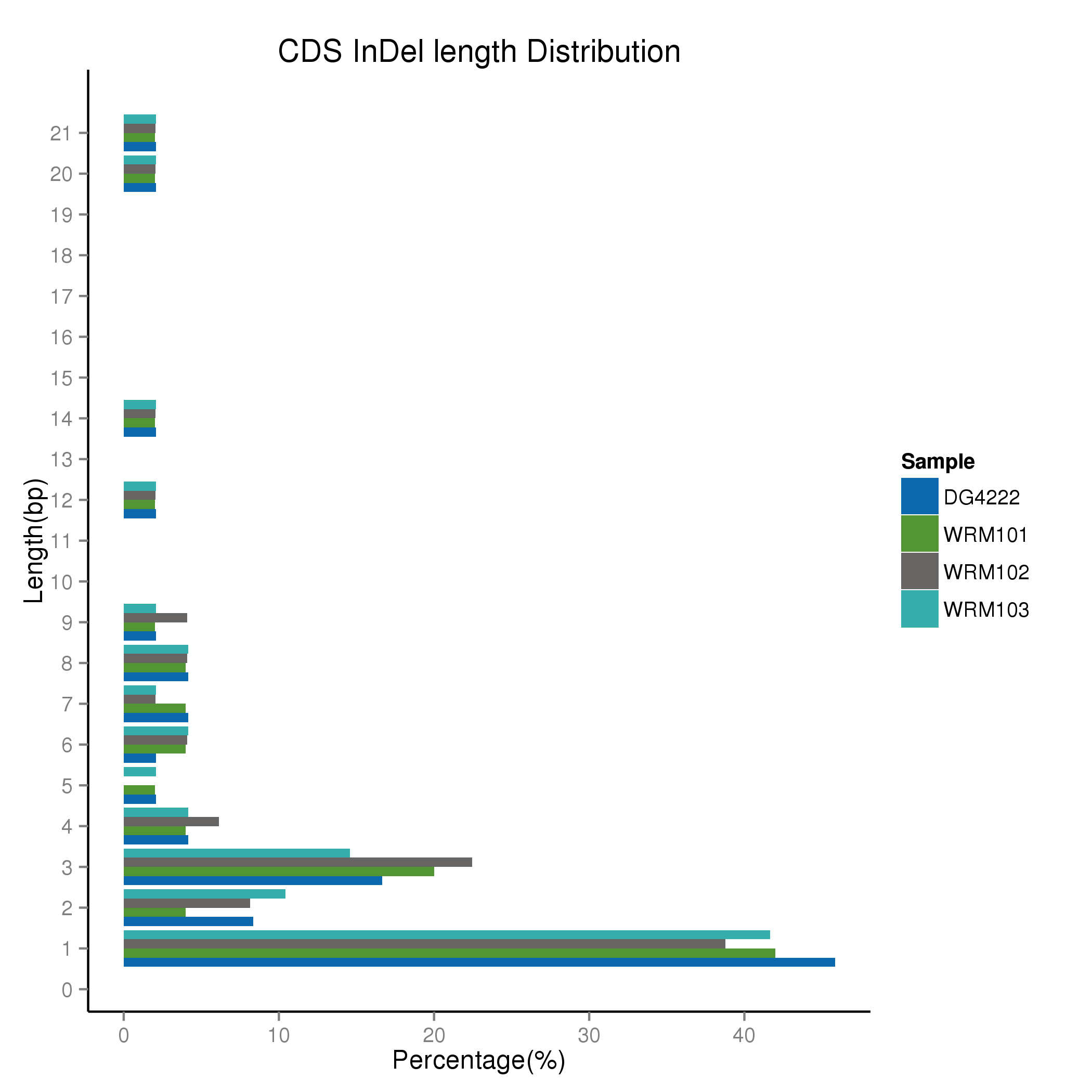

Supplement: S2 Data — (GZ) [file pgen.1012129.s008.gz › SupplementalDataSet1/03.Result_X202SC24112711-Z01-F001_C_elegans/report/src/pictures/InDel/InDel_CDSpercentage.png]

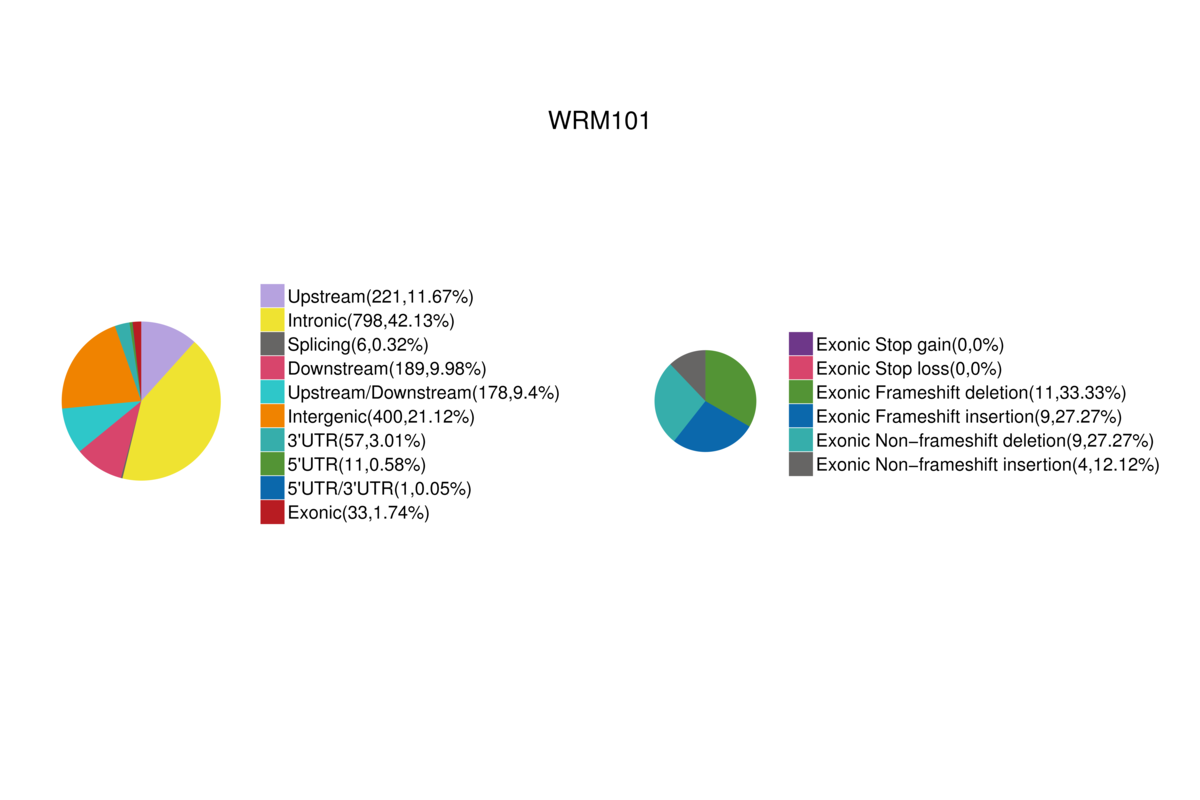

Supplement: S2 Data — (GZ) [file pgen.1012129.s008.gz › SupplementalDataSet1/03.Result_X202SC24112711-Z01-F001_C_elegans/report/src/pictures/InDel/WRM101.InDel.table.png]

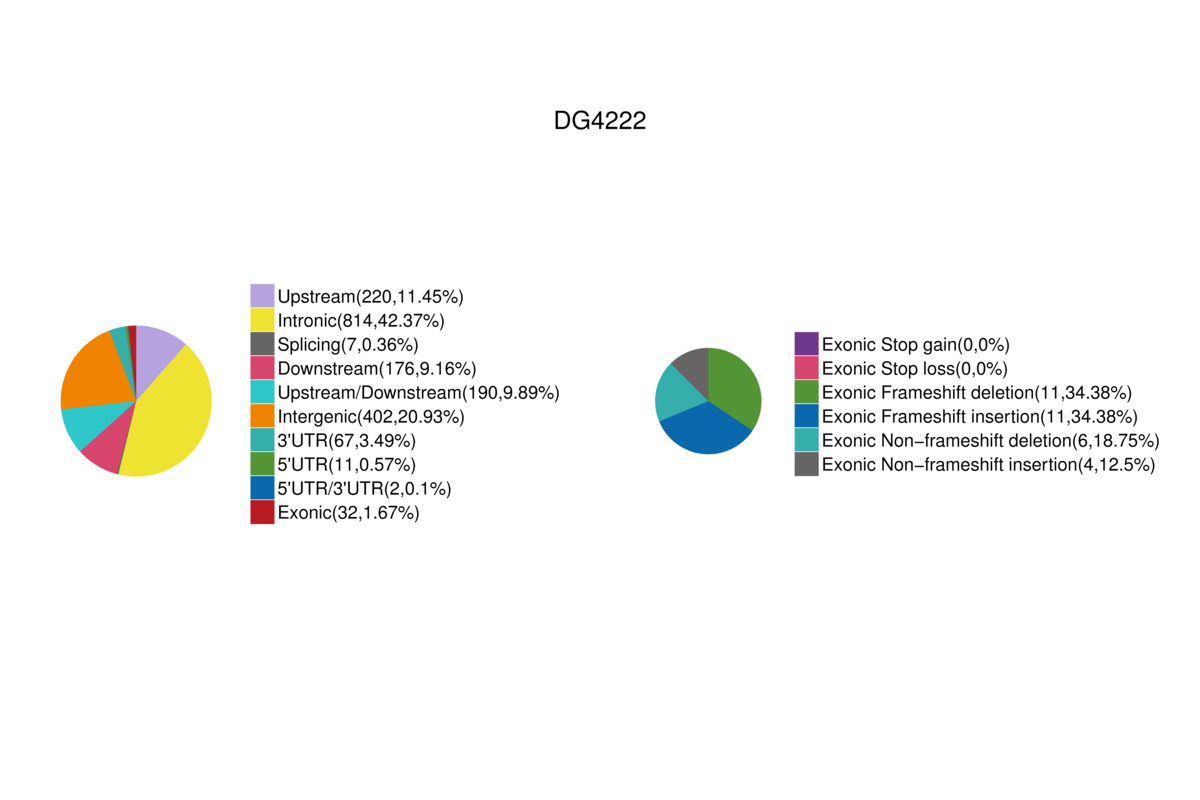

Supplement: S2 Data — (GZ) [file pgen.1012129.s008.gz › SupplementalDataSet1/03.Result_X202SC24112711-Z01-F001_C_elegans/report/src/pictures/InDel/DG4222.InDel.table.png]

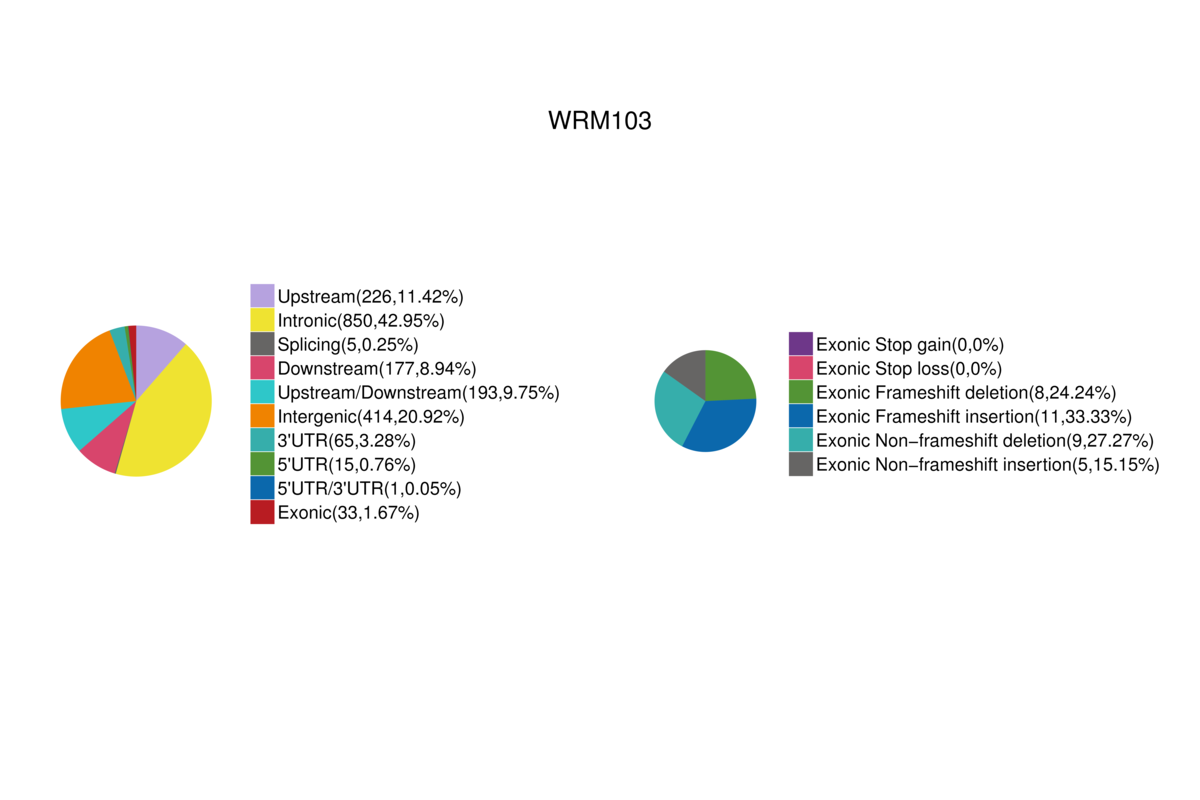

Supplement: S2 Data — (GZ) [file pgen.1012129.s008.gz › SupplementalDataSet1/03.Result_X202SC24112711-Z01-F001_C_elegans/report/src/pictures/InDel/WRM103.InDel.table.png]

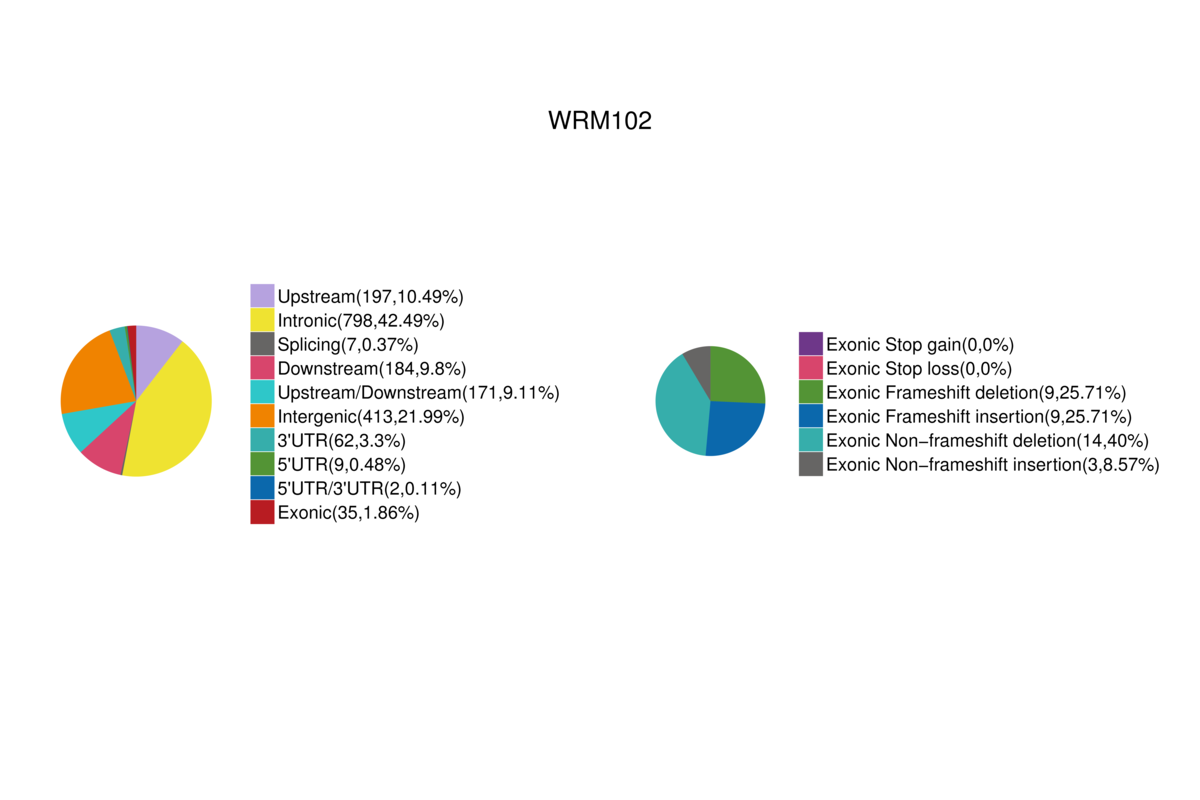

Supplement: S2 Data — (GZ) [file pgen.1012129.s008.gz › SupplementalDataSet1/03.Result_X202SC24112711-Z01-F001_C_elegans/report/src/pictures/InDel/WRM102.InDel.table.png]

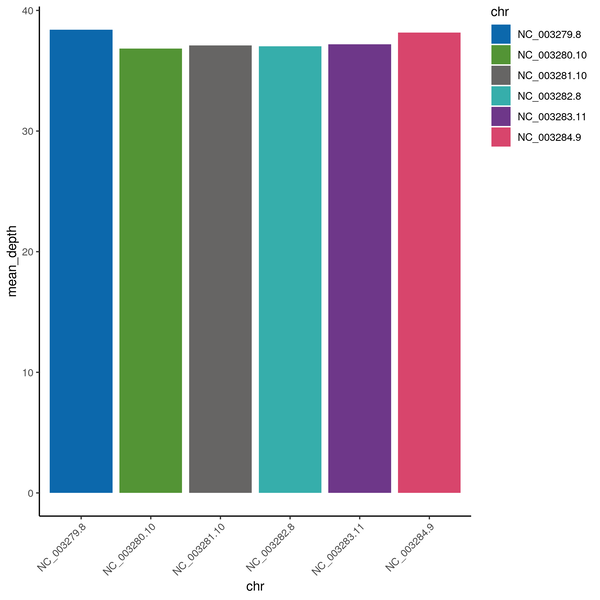

Supplement: S2 Data — (GZ) [file pgen.1012129.s008.gz › SupplementalDataSet1/03.Result_X202SC24112711-Z01-F001_C_elegans/report/src/pictures/Map/DG4222.mapbychrdepth.png]

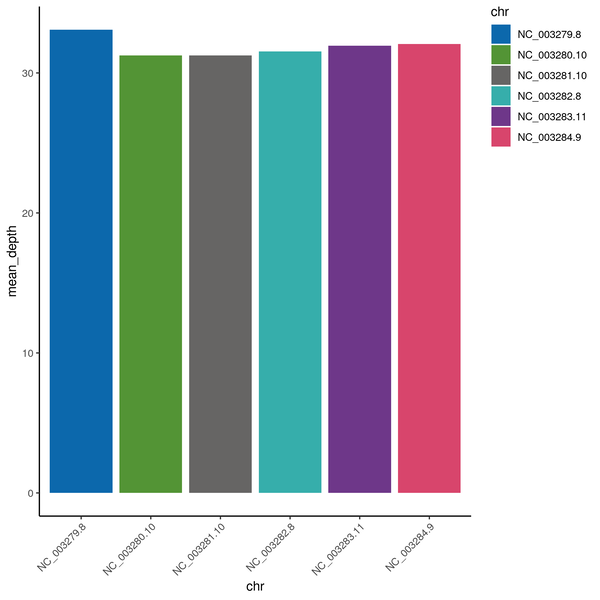

Supplement: S2 Data — (GZ) [file pgen.1012129.s008.gz › SupplementalDataSet1/03.Result_X202SC24112711-Z01-F001_C_elegans/report/src/pictures/Map/WRM101.mapbychrdepth.png]

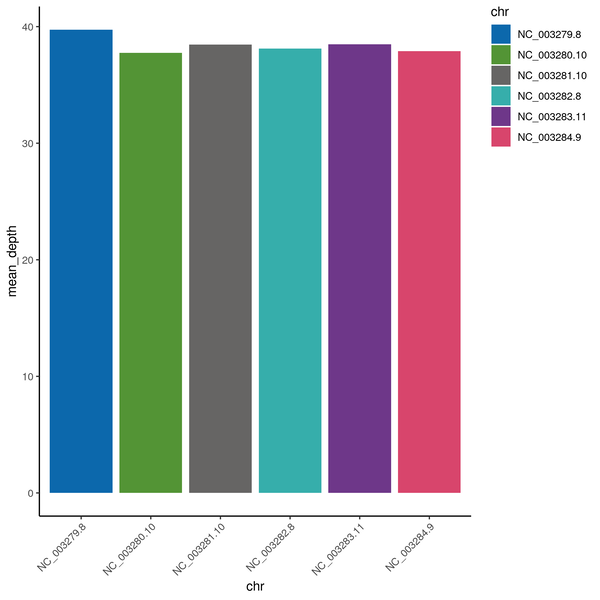

Supplement: S2 Data — (GZ) [file pgen.1012129.s008.gz › SupplementalDataSet1/03.Result_X202SC24112711-Z01-F001_C_elegans/report/src/pictures/Map/WRM103.mapbychrdepth.png]

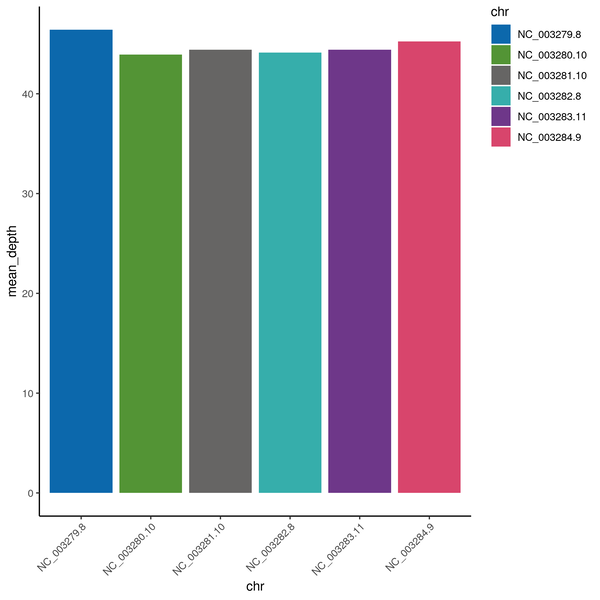

Supplement: S2 Data — (GZ) [file pgen.1012129.s008.gz › SupplementalDataSet1/03.Result_X202SC24112711-Z01-F001_C_elegans/report/src/pictures/Map/WRM102.mapbychrdepth.png]

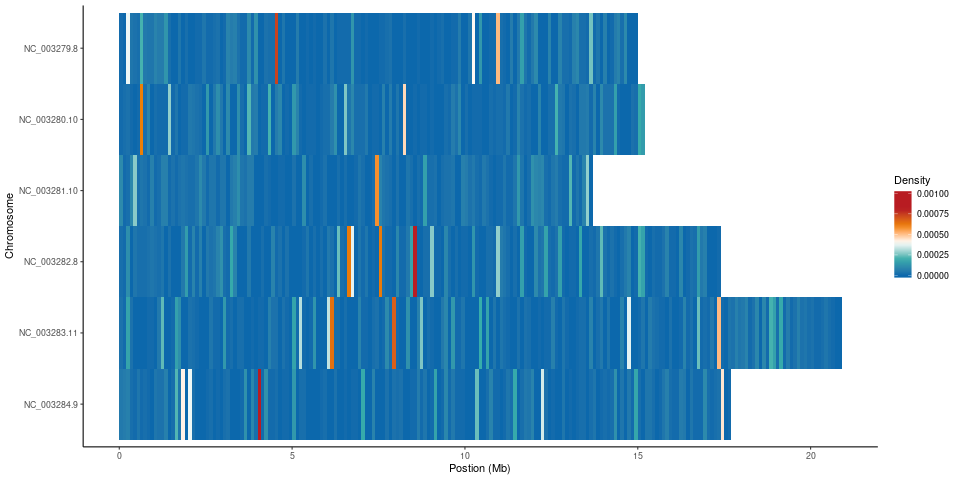

Supplement: S2 Data — (GZ) [file pgen.1012129.s008.gz › SupplementalDataSet1/03.Result_X202SC24112711-Z01-F001_C_elegans/report/src/pictures/Circos/WRM102.snpDensity.png]

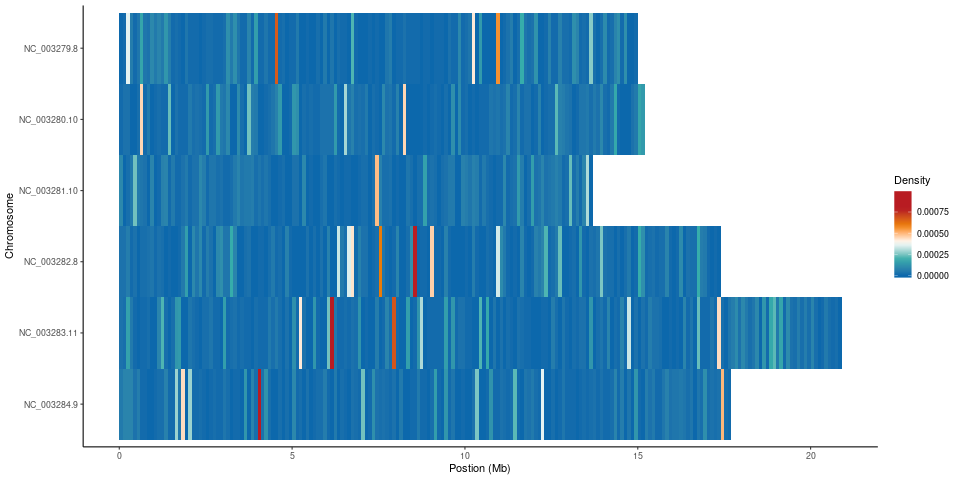

Supplement: S2 Data — (GZ) [file pgen.1012129.s008.gz › SupplementalDataSet1/03.Result_X202SC24112711-Z01-F001_C_elegans/report/src/pictures/Circos/WRM103.snpDensity.png]

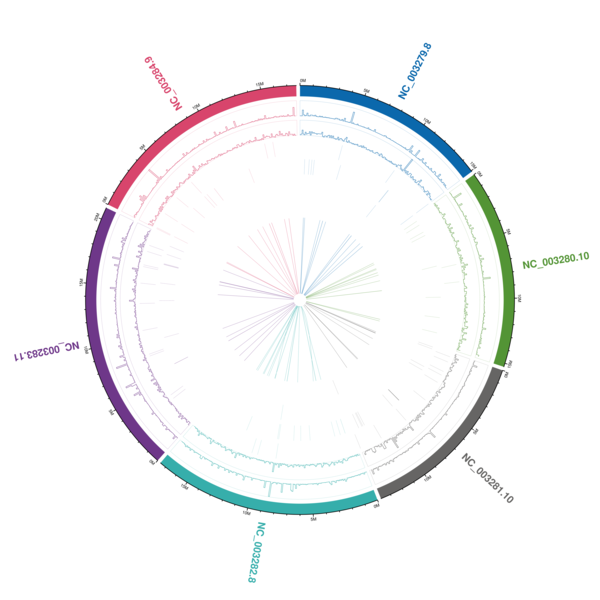

Supplement: S2 Data — (GZ) [file pgen.1012129.s008.gz › SupplementalDataSet1/03.Result_X202SC24112711-Z01-F001_C_elegans/report/src/pictures/Circos/DG4222.JPEG]

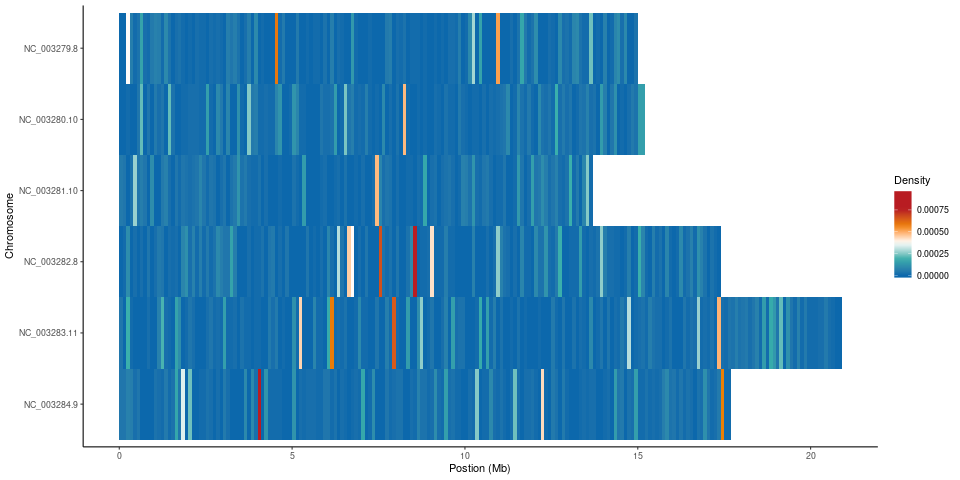

Supplement: S2 Data — (GZ) [file pgen.1012129.s008.gz › SupplementalDataSet1/03.Result_X202SC24112711-Z01-F001_C_elegans/report/src/pictures/Circos/WRM101.snpDensity.png]

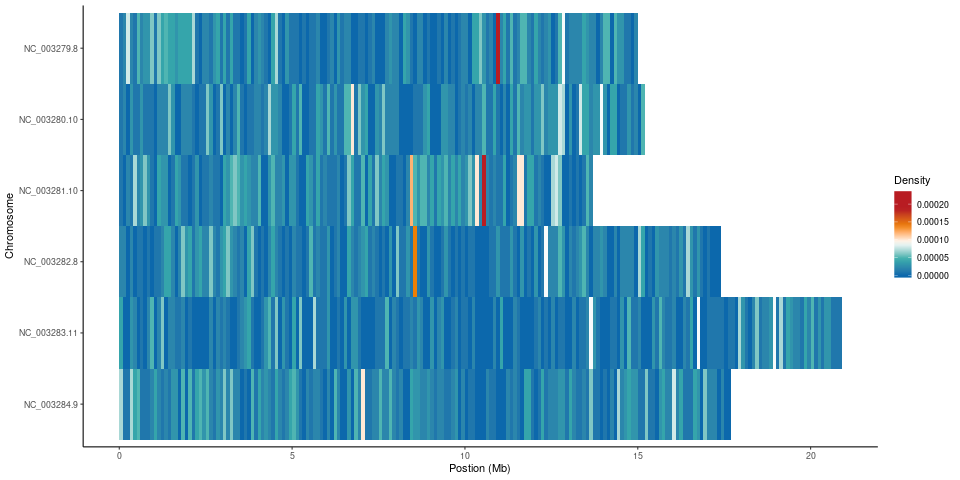

Supplement: S2 Data — (GZ) [file pgen.1012129.s008.gz › SupplementalDataSet1/03.Result_X202SC24112711-Z01-F001_C_elegans/report/src/pictures/Circos/DG4222.indDensity.png]

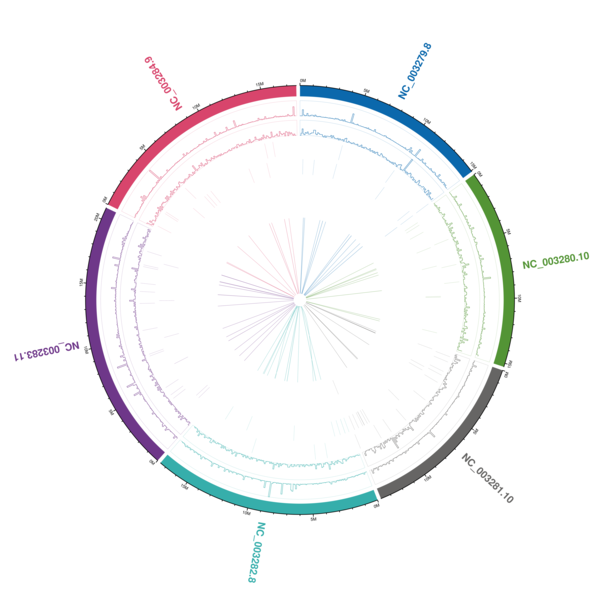

Supplement: S2 Data — (GZ) [file pgen.1012129.s008.gz › SupplementalDataSet1/03.Result_X202SC24112711-Z01-F001_C_elegans/report/src/pictures/Circos/WRM101.JPEG]

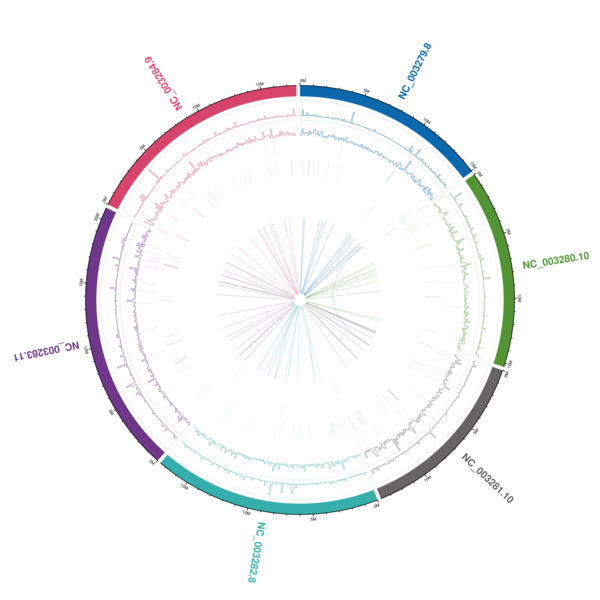

Supplement: S2 Data — (GZ) [file pgen.1012129.s008.gz › SupplementalDataSet1/03.Result_X202SC24112711-Z01-F001_C_elegans/report/src/pictures/Circos/WRM102.JPEG]

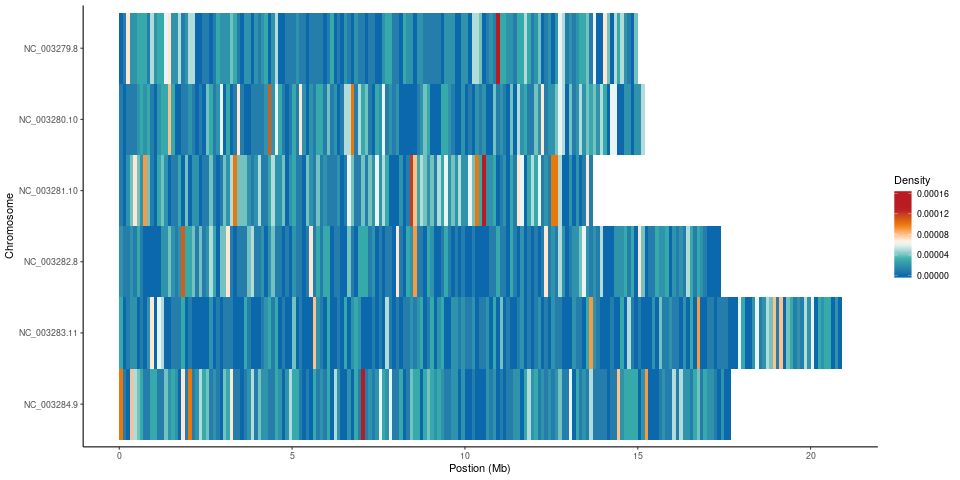

Supplement: S2 Data — (GZ) [file pgen.1012129.s008.gz › SupplementalDataSet1/03.Result_X202SC24112711-Z01-F001_C_elegans/report/src/pictures/Circos/WRM102.indDensity.png]

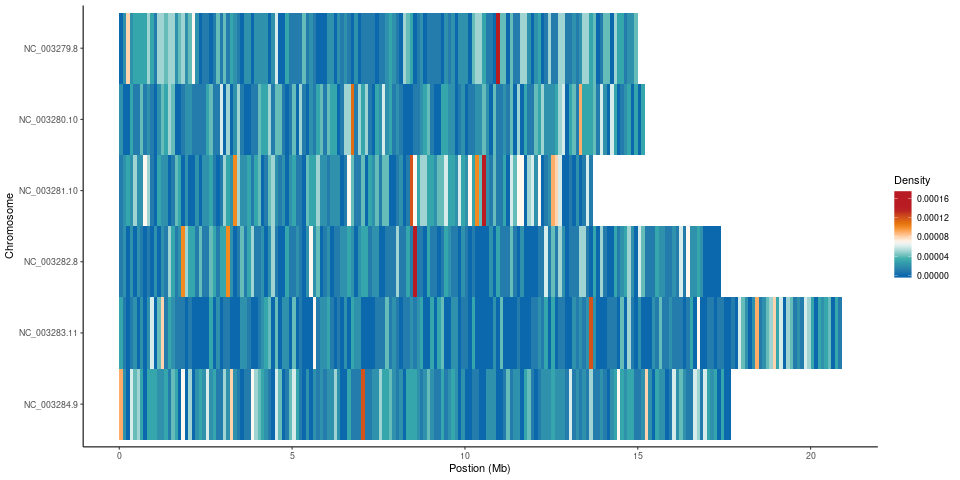

Supplement: S2 Data — (GZ) [file pgen.1012129.s008.gz › SupplementalDataSet1/03.Result_X202SC24112711-Z01-F001_C_elegans/report/src/pictures/Circos/WRM103.indDensity.png]

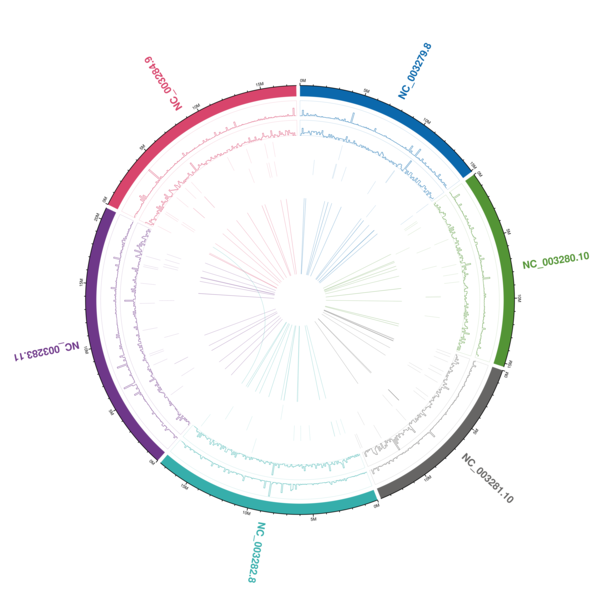

Supplement: S2 Data — (GZ) [file pgen.1012129.s008.gz › SupplementalDataSet1/03.Result_X202SC24112711-Z01-F001_C_elegans/report/src/pictures/Circos/WRM103.JPEG]

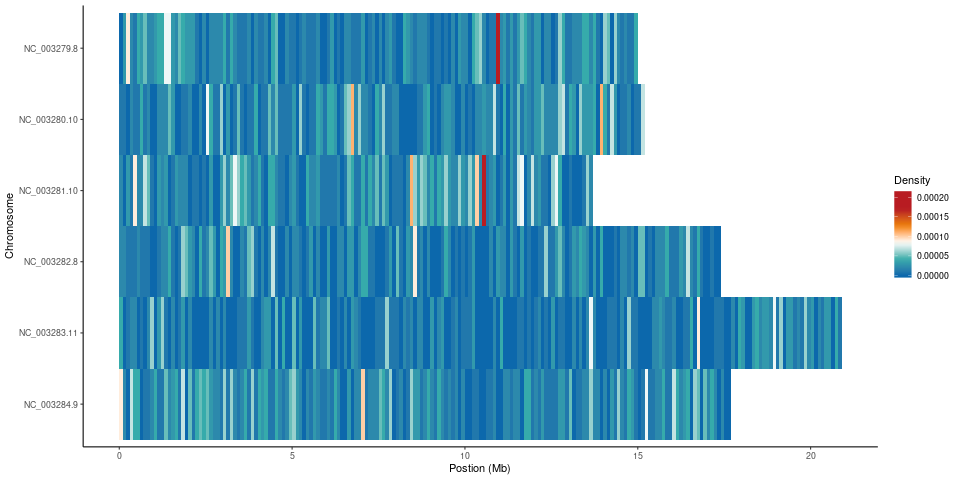

Supplement: S2 Data — (GZ) [file pgen.1012129.s008.gz › SupplementalDataSet1/03.Result_X202SC24112711-Z01-F001_C_elegans/report/src/pictures/Circos/WRM101.indDensity.png]

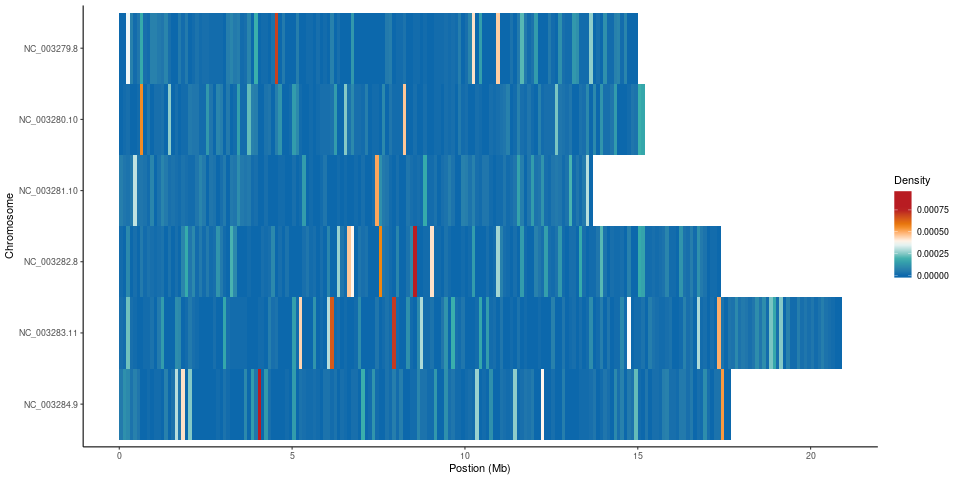

Supplement: S2 Data — (GZ) [file pgen.1012129.s008.gz › SupplementalDataSet1/03.Result_X202SC24112711-Z01-F001_C_elegans/report/src/pictures/Circos/DG4222.snpDensity.png]

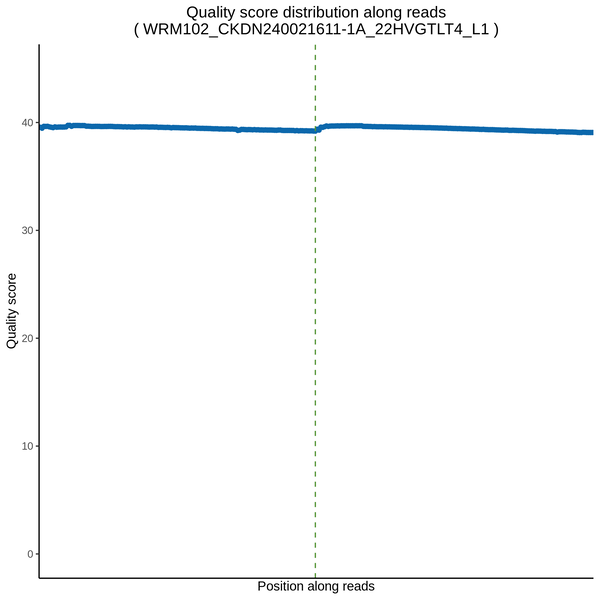

Supplement: S2 Data — (GZ) [file pgen.1012129.s008.gz › SupplementalDataSet1/03.Result_X202SC24112711-Z01-F001_C_elegans/report/src/pictures/Quality/WRM102_CKDN240021611-1A_22HVGTLT4_L1.QM.png]

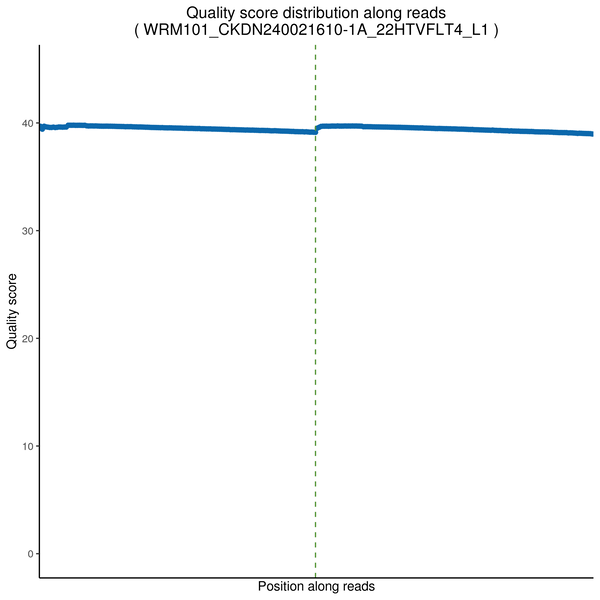

Supplement: S2 Data — (GZ) [file pgen.1012129.s008.gz › SupplementalDataSet1/03.Result_X202SC24112711-Z01-F001_C_elegans/report/src/pictures/Quality/WRM101_CKDN240021610-1A_22HTVFLT4_L1.QM.JPEG]

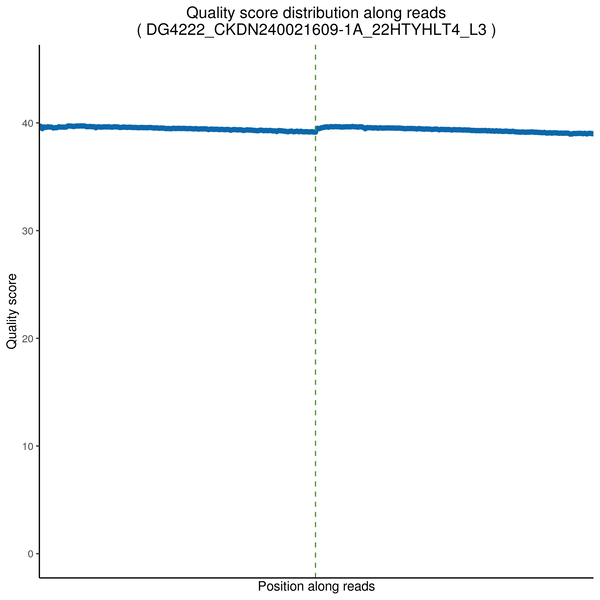

Supplement: S2 Data — (GZ) [file pgen.1012129.s008.gz › SupplementalDataSet1/03.Result_X202SC24112711-Z01-F001_C_elegans/report/src/pictures/Quality/DG4222_CKDN240021609-1A_22HTYHLT4_L3.QM.png]

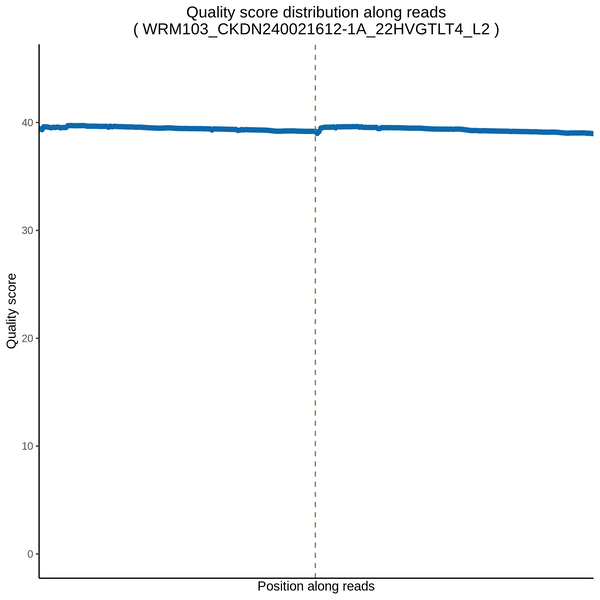

Supplement: S2 Data — (GZ) [file pgen.1012129.s008.gz › SupplementalDataSet1/03.Result_X202SC24112711-Z01-F001_C_elegans/report/src/pictures/Quality/WRM103_CKDN240021612-1A_22HVGTLT4_L2.QM.png]

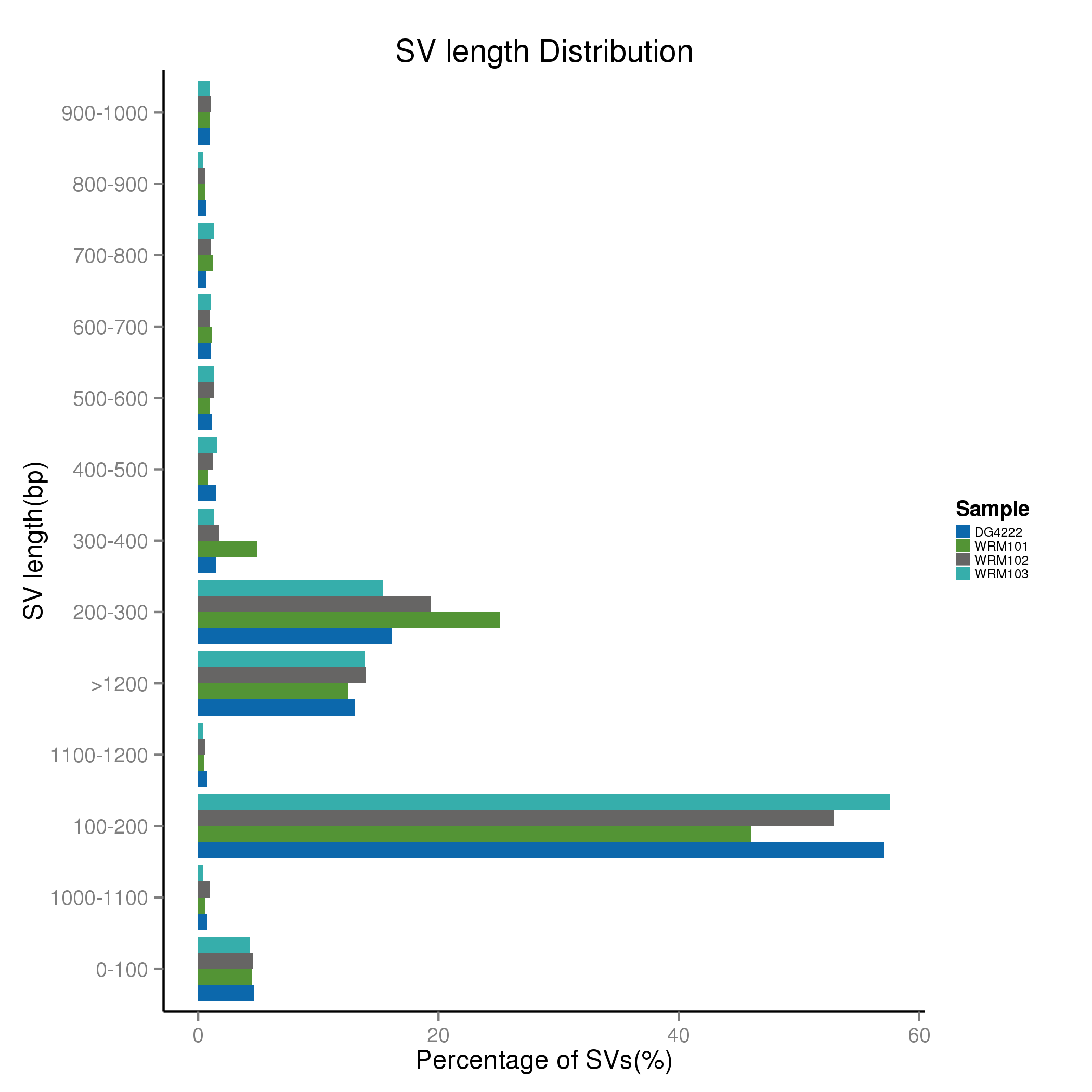

Supplement: S2 Data — (GZ) [file pgen.1012129.s008.gz › SupplementalDataSet1/03.Result_X202SC24112711-Z01-F001_C_elegans/report/src/pictures/SV/SV_length_distribution.png]

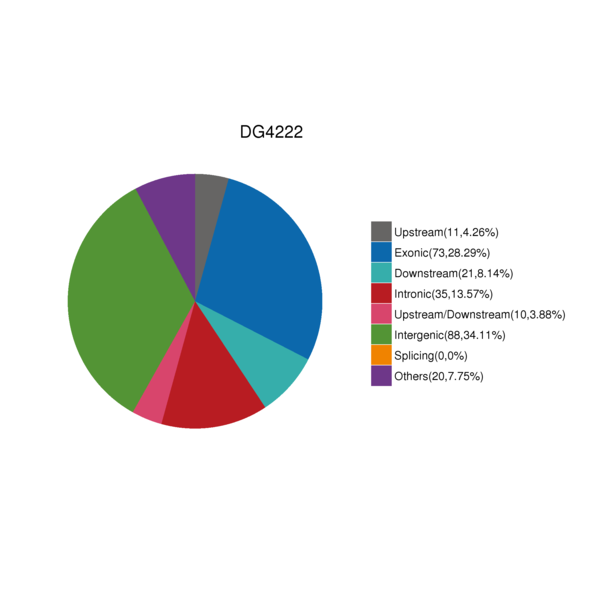

Supplement: S2 Data — (GZ) [file pgen.1012129.s008.gz › SupplementalDataSet1/03.Result_X202SC24112711-Z01-F001_C_elegans/report/src/pictures/SV/DG4222.SV.table.png]

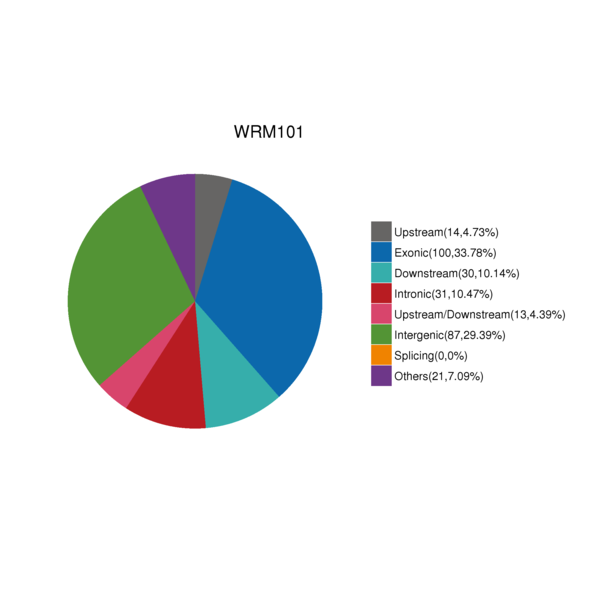

Supplement: S2 Data — (GZ) [file pgen.1012129.s008.gz › SupplementalDataSet1/03.Result_X202SC24112711-Z01-F001_C_elegans/report/src/pictures/SV/WRM101.SV.table.png]

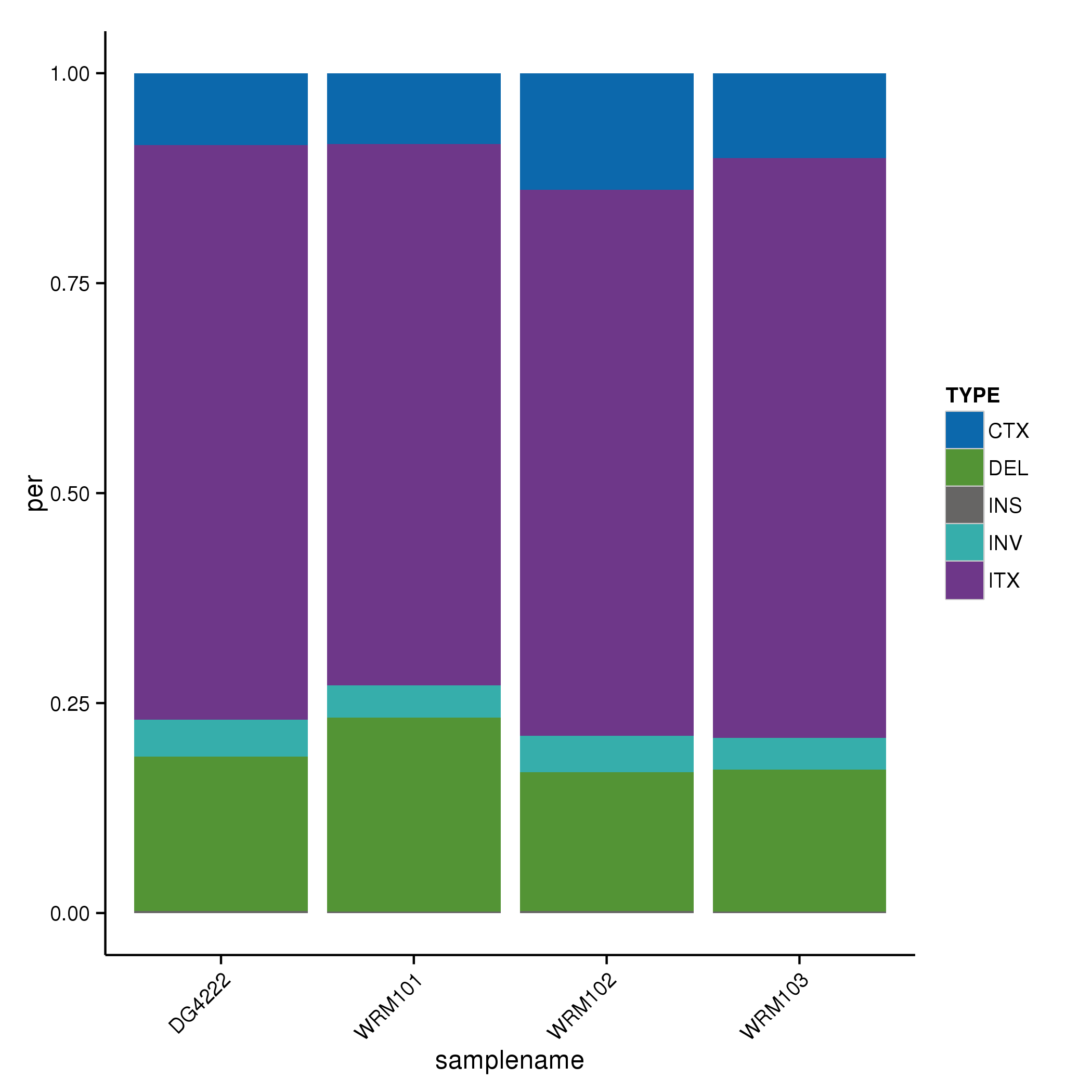

Supplement: S2 Data — (GZ) [file pgen.1012129.s008.gz › SupplementalDataSet1/03.Result_X202SC24112711-Z01-F001_C_elegans/report/src/pictures/SV/SV_ann_Variation_type_statistics_distribution.png]

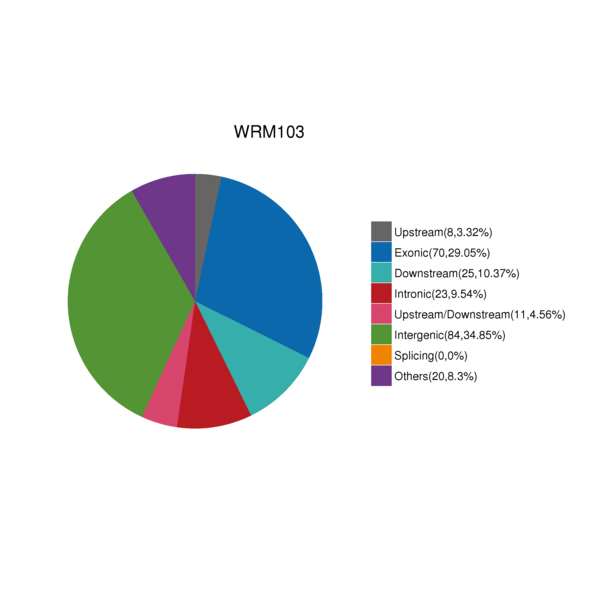

Supplement: S2 Data — (GZ) [file pgen.1012129.s008.gz › SupplementalDataSet1/03.Result_X202SC24112711-Z01-F001_C_elegans/report/src/pictures/SV/WRM103.SV.table.png]

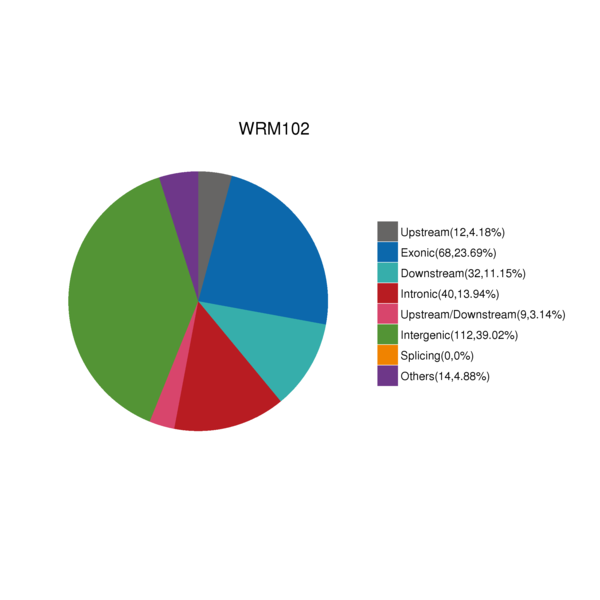

Supplement: S2 Data — (GZ) [file pgen.1012129.s008.gz › SupplementalDataSet1/03.Result_X202SC24112711-Z01-F001_C_elegans/report/src/pictures/SV/WRM102.SV.table.png]

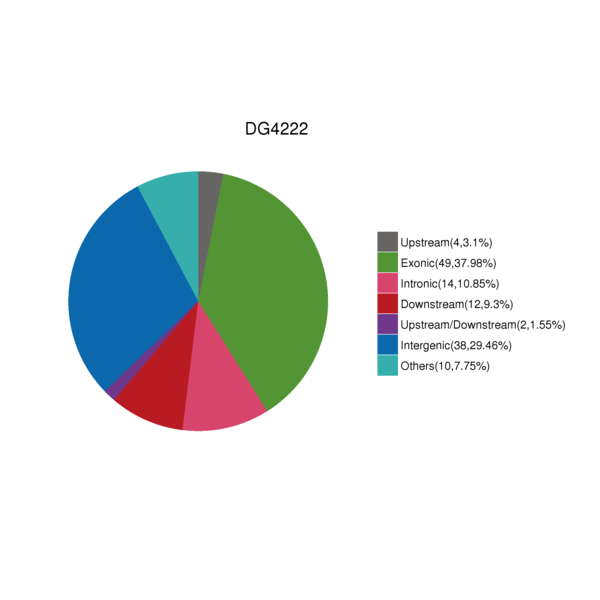

Supplement: S2 Data — (GZ) [file pgen.1012129.s008.gz › SupplementalDataSet1/03.Result_X202SC24112711-Z01-F001_C_elegans/report/src/pictures/CNV/DG4222.CNV.table.png]

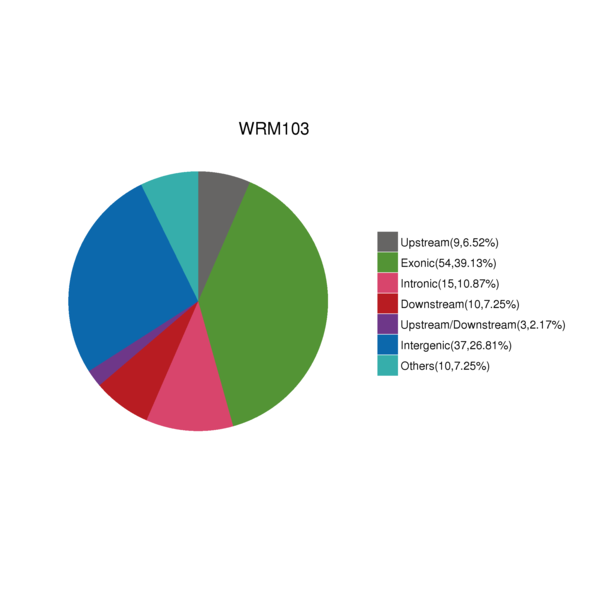

Supplement: S2 Data — (GZ) [file pgen.1012129.s008.gz › SupplementalDataSet1/03.Result_X202SC24112711-Z01-F001_C_elegans/report/src/pictures/CNV/WRM103.CNV.table.png]

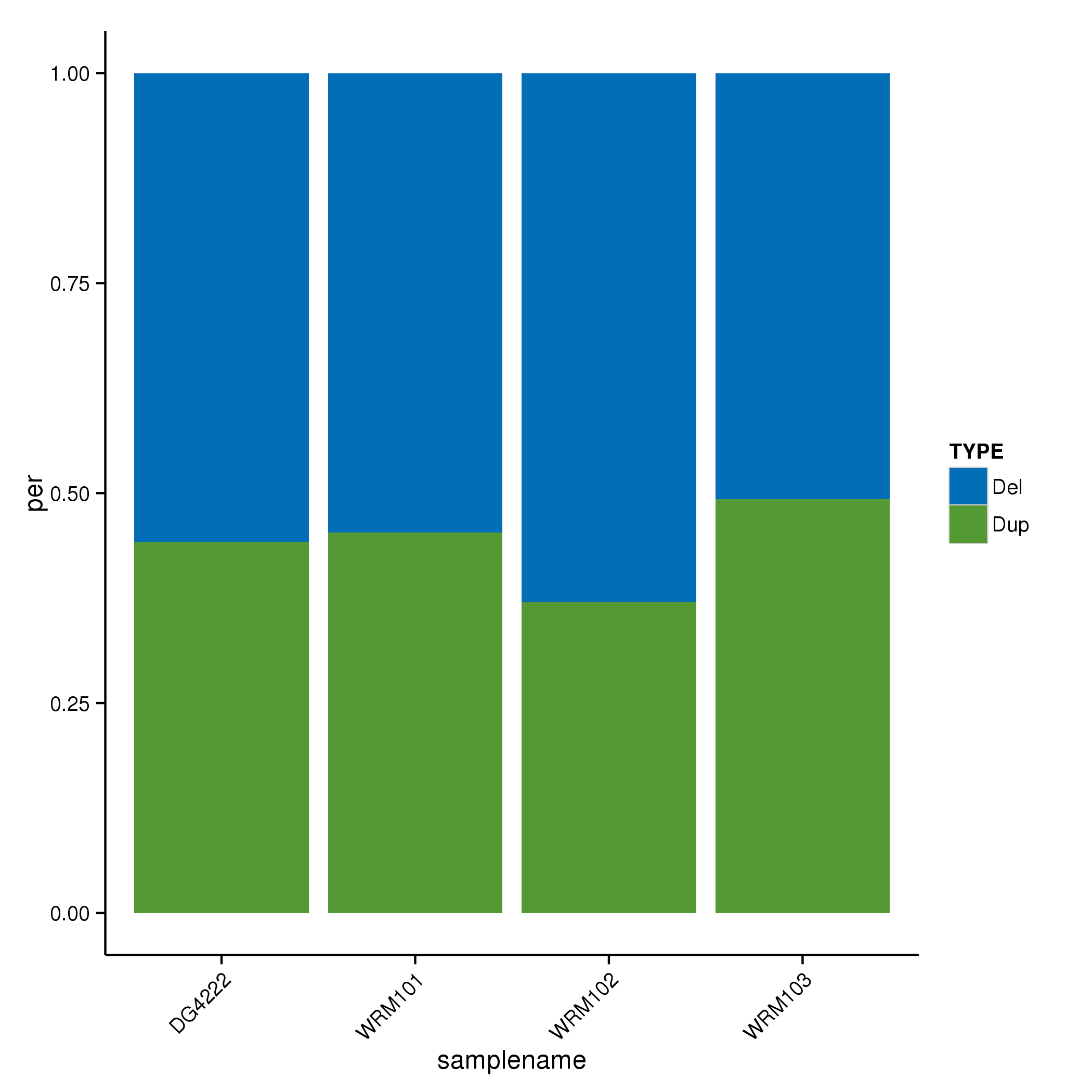

Supplement: S2 Data — (GZ) [file pgen.1012129.s008.gz › SupplementalDataSet1/03.Result_X202SC24112711-Z01-F001_C_elegans/report/src/pictures/CNV/CNV_ann_Variation_type_statistics_distribution.png]

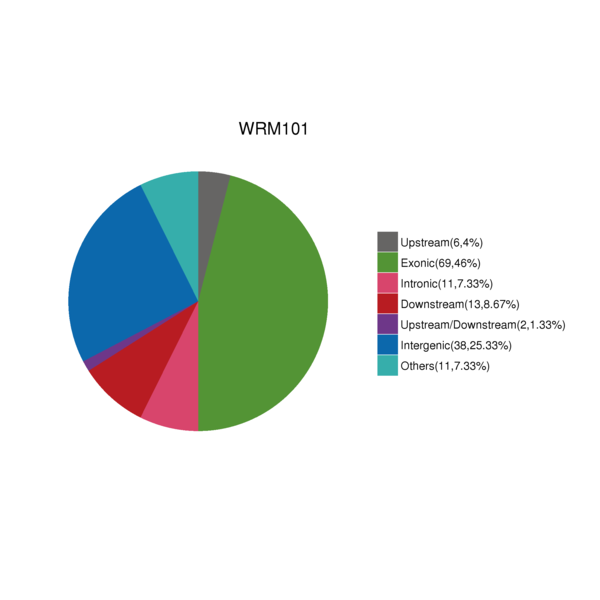

Supplement: S2 Data — (GZ) [file pgen.1012129.s008.gz › SupplementalDataSet1/03.Result_X202SC24112711-Z01-F001_C_elegans/report/src/pictures/CNV/WRM101.CNV.table.png]

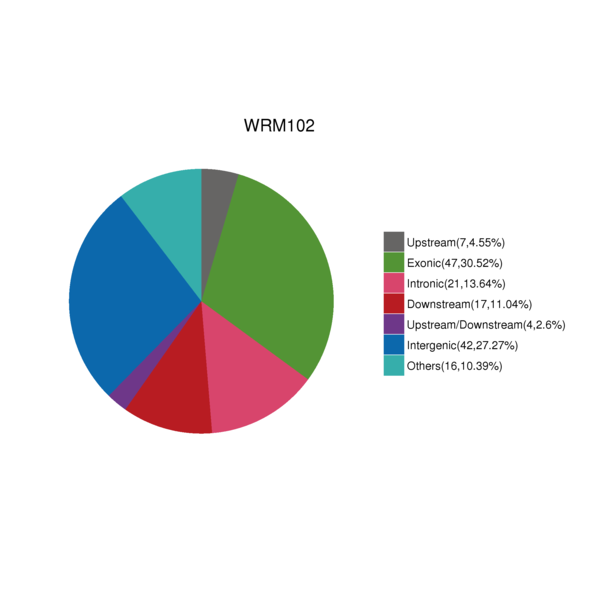

Supplement: S2 Data — (GZ) [file pgen.1012129.s008.gz › SupplementalDataSet1/03.Result_X202SC24112711-Z01-F001_C_elegans/report/src/pictures/CNV/WRM102.CNV.table.png]

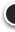

Supplement: S2 Data — (GZ) [file pgen.1012129.s008.gz › SupplementalDataSet1/03.Result_X202SC24112711-Z01-F001_C_elegans/report/src/js/fancybox/fancy_title_left.png]

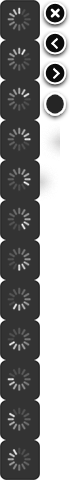

Supplement: S2 Data — (GZ) [file pgen.1012129.s008.gz › SupplementalDataSet1/03.Result_X202SC24112711-Z01-F001_C_elegans/report/src/js/fancybox/fancybox.png]

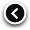

Supplement: S2 Data — (GZ) [file pgen.1012129.s008.gz › SupplementalDataSet1/03.Result_X202SC24112711-Z01-F001_C_elegans/report/src/js/fancybox/fancy_nav_left.png]

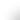

Supplement: S2 Data — (GZ) [file pgen.1012129.s008.gz › SupplementalDataSet1/03.Result_X202SC24112711-Z01-F001_C_elegans/report/src/js/fancybox/fancy_shadow_nw.png]

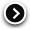

Supplement: S2 Data — (GZ) [file pgen.1012129.s008.gz › SupplementalDataSet1/03.Result_X202SC24112711-Z01-F001_C_elegans/report/src/js/fancybox/fancy_nav_right.png]

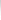

Supplement: S2 Data — (GZ) [file pgen.1012129.s008.gz › SupplementalDataSet1/03.Result_X202SC24112711-Z01-F001_C_elegans/report/src/js/fancybox/fancy_shadow_s.png]

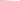

Supplement: S2 Data — (GZ) [file pgen.1012129.s008.gz › SupplementalDataSet1/03.Result_X202SC24112711-Z01-F001_C_elegans/report/src/js/fancybox/fancy_shadow_e.png]

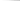

Supplement: S2 Data — (GZ) [file pgen.1012129.s008.gz › SupplementalDataSet1/03.Result_X202SC24112711-Z01-F001_C_elegans/report/src/js/fancybox/fancy_shadow_w.png]

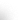

Supplement: S2 Data — (GZ) [file pgen.1012129.s008.gz › SupplementalDataSet1/03.Result_X202SC24112711-Z01-F001_C_elegans/report/src/js/fancybox/fancy_shadow_ne.png]

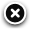

Supplement: S2 Data — (GZ) [file pgen.1012129.s008.gz › SupplementalDataSet1/03.Result_X202SC24112711-Z01-F001_C_elegans/report/src/js/fancybox/fancy_close.png]

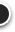

Supplement: S2 Data — (GZ) [file pgen.1012129.s008.gz › SupplementalDataSet1/03.Result_X202SC24112711-Z01-F001_C_elegans/report/src/js/fancybox/fancy_title_right.png]

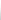

Supplement: S2 Data — (GZ) [file pgen.1012129.s008.gz › SupplementalDataSet1/03.Result_X202SC24112711-Z01-F001_C_elegans/report/src/js/fancybox/fancy_shadow_n.png]

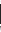

Supplement: S2 Data — (GZ) [file pgen.1012129.s008.gz › SupplementalDataSet1/03.Result_X202SC24112711-Z01-F001_C_elegans/report/src/js/fancybox/fancy_title_main.png]

Supplement: S2 Data — (GZ) [file pgen.1012129.s008.gz › SupplementalDataSet1/03.Result_X202SC24112711-Z01-F001_C_elegans/report/src/js/fancybox/fancy_title_over.png]

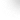

Supplement: S2 Data — (GZ) [file pgen.1012129.s008.gz › SupplementalDataSet1/03.Result_X202SC24112711-Z01-F001_C_elegans/report/src/js/fancybox/fancy_shadow_sw.png]

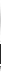

Supplement: S2 Data — (GZ) [file pgen.1012129.s008.gz › SupplementalDataSet1/03.Result_X202SC24112711-Z01-F001_C_elegans/report/src/js/fancybox/fancybox-x.png]

Supplement: S2 Data — (GZ) [file pgen.1012129.s008.gz › SupplementalDataSet1/03.Result_X202SC24112711-Z01-F001_C_elegans/report/src/js/fancybox/blank.gif]

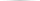

Supplement: S2 Data — (GZ) [file pgen.1012129.s008.gz › SupplementalDataSet1/03.Result_X202SC24112711-Z01-F001_C_elegans/report/src/js/fancybox/fancybox-y.png]

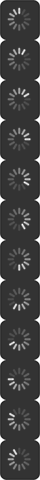

Supplement: S2 Data — (GZ) [file pgen.1012129.s008.gz › SupplementalDataSet1/03.Result_X202SC24112711-Z01-F001_C_elegans/report/src/js/fancybox/fancy_loading.png]

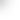

Supplement: S2 Data — (GZ) [file pgen.1012129.s008.gz › SupplementalDataSet1/03.Result_X202SC24112711-Z01-F001_C_elegans/report/src/js/fancybox/fancy_shadow_se.png]

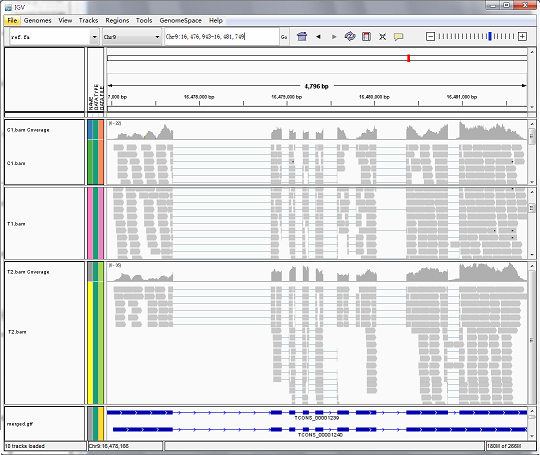

Supplement: S2 Data — (GZ) [file pgen.1012129.s008.gz › SupplementalDataSet1/03.Result_X202SC24112711-Z01-F001_C_elegans/report/src/images/igv.png]

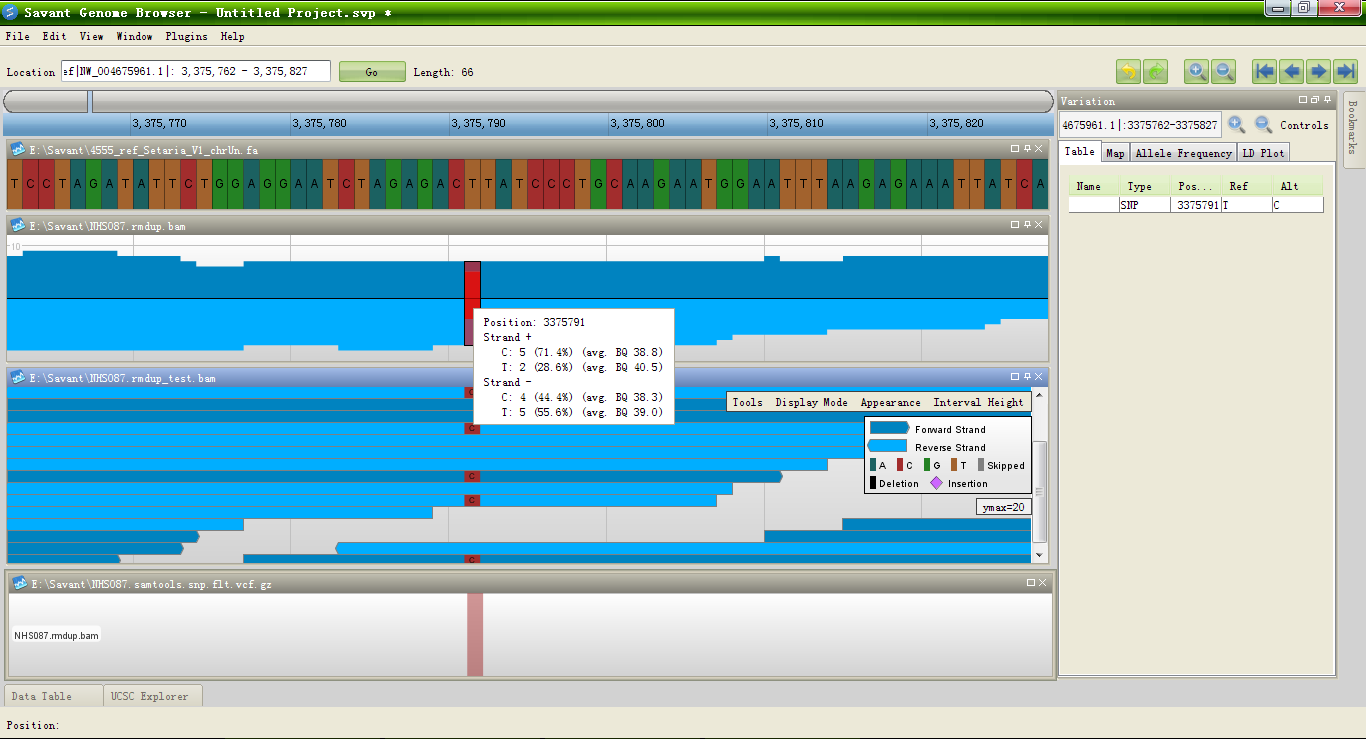

Supplement: S2 Data — (GZ) [file pgen.1012129.s008.gz › SupplementalDataSet1/03.Result_X202SC24112711-Z01-F001_C_elegans/report/src/images/savant.png]

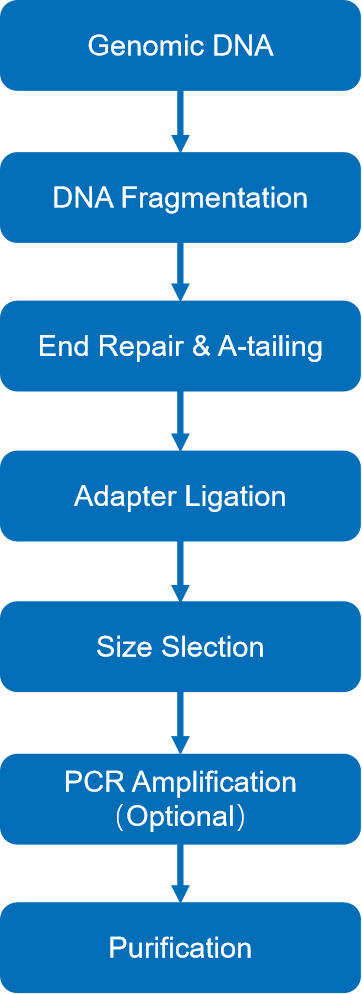

Supplement: S2 Data — (GZ) [file pgen.1012129.s008.gz › SupplementalDataSet1/03.Result_X202SC24112711-Z01-F001_C_elegans/report/src/images/wgs_experiment_pipeline.png]

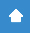

Supplement: S2 Data — (GZ) [file pgen.1012129.s008.gz › SupplementalDataSet1/03.Result_X202SC24112711-Z01-F001_C_elegans/report/src/images/goTop.jpg]

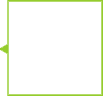

Supplement: S2 Data — (GZ) [file pgen.1012129.s008.gz › SupplementalDataSet1/03.Result_X202SC24112711-Z01-F001_C_elegans/report/src/images/album-slider-arrow_box.png]

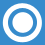

Supplement: S2 Data — (GZ) [file pgen.1012129.s008.gz › SupplementalDataSet1/03.Result_X202SC24112711-Z01-F001_C_elegans/report/src/images/open.gif]

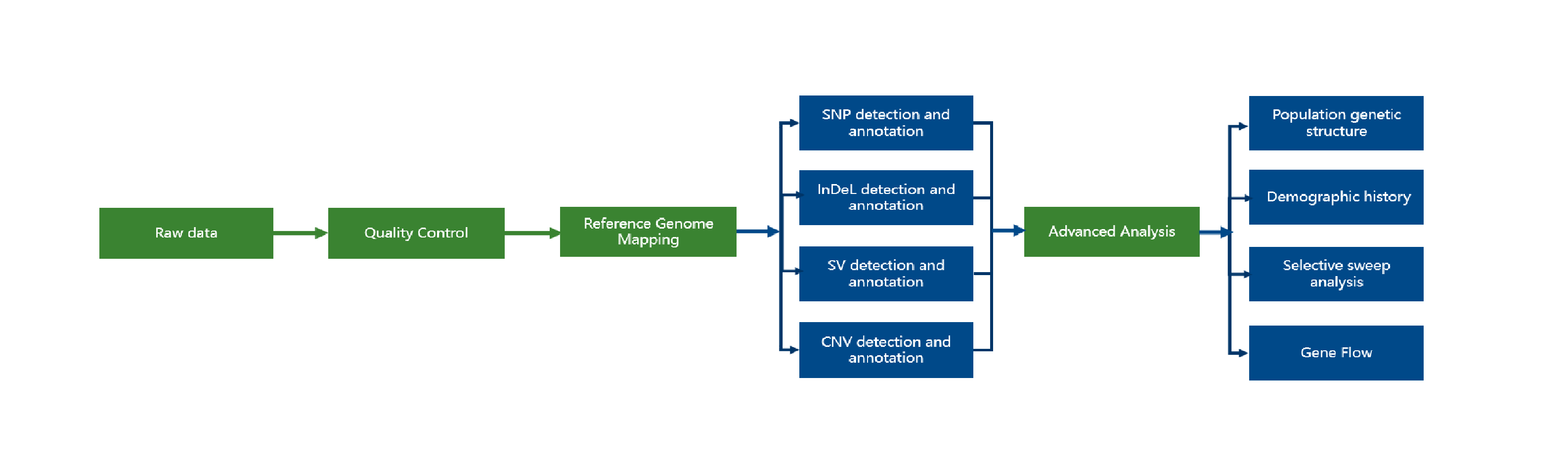

Supplement: S2 Data — (GZ) [file pgen.1012129.s008.gz › SupplementalDataSet1/03.Result_X202SC24112711-Z01-F001_C_elegans/report/src/images/bioinfoWorkflow.adv.png]

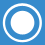

Supplement: S2 Data — (GZ) [file pgen.1012129.s008.gz › SupplementalDataSet1/03.Result_X202SC24112711-Z01-F001_C_elegans/report/src/images/close.gif]

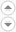

Supplement: S2 Data — (GZ) [file pgen.1012129.s008.gz › SupplementalDataSet1/03.Result_X202SC24112711-Z01-F001_C_elegans/report/src/images/album-slider-button.png]

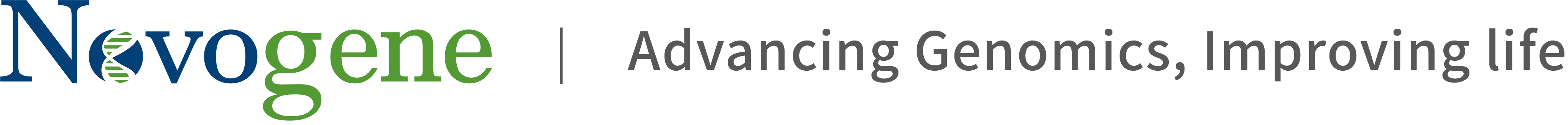

Supplement: S2 Data — (GZ) [file pgen.1012129.s008.gz › SupplementalDataSet1/03.Result_X202SC24112711-Z01-F001_C_elegans/report/src/images/logo1.png]

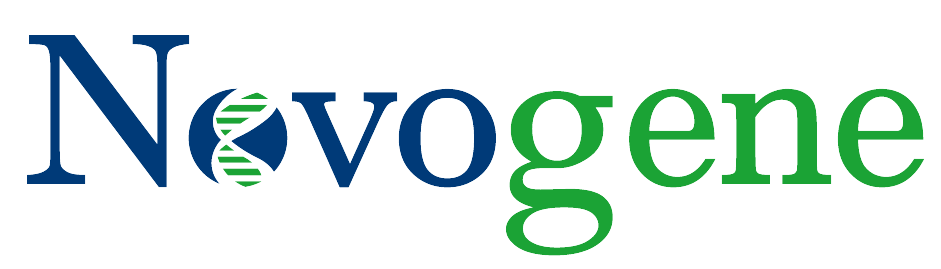

Supplement: S2 Data — (GZ) [file pgen.1012129.s008.gz › SupplementalDataSet1/03.Result_X202SC24112711-Z01-F001_C_elegans/report/src/images/logo.png]

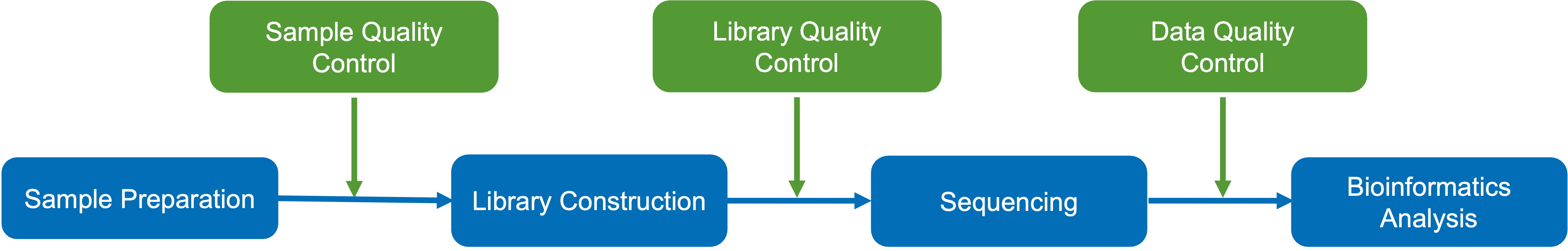

Supplement: S2 Data — (GZ) [file pgen.1012129.s008.gz › SupplementalDataSet1/03.Result_X202SC24112711-Z01-F001_C_elegans/report/src/images/experimentWorkflow.png]

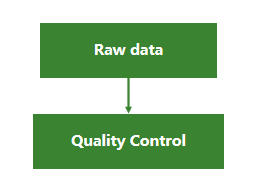

Supplement: S2 Data — (GZ) [file pgen.1012129.s008.gz › SupplementalDataSet1/03.Result_X202SC24112711-Z01-F001_C_elegans/report/src/images/QC.png]

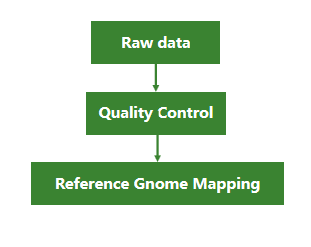

Supplement: S2 Data — (GZ) [file pgen.1012129.s008.gz › SupplementalDataSet1/03.Result_X202SC24112711-Z01-F001_C_elegans/report/src/images/mapping.png]

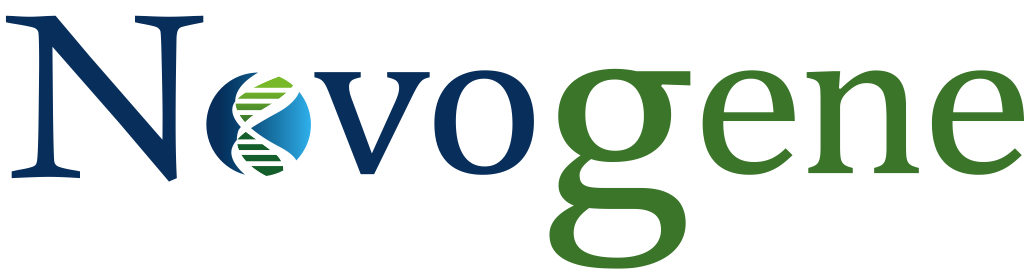

Supplement: S2 Data — (GZ) [file pgen.1012129.s008.gz › SupplementalDataSet1/03.Result_X202SC24112711-Z01-F001_C_elegans/report/src/images/novogeneLogo.png]

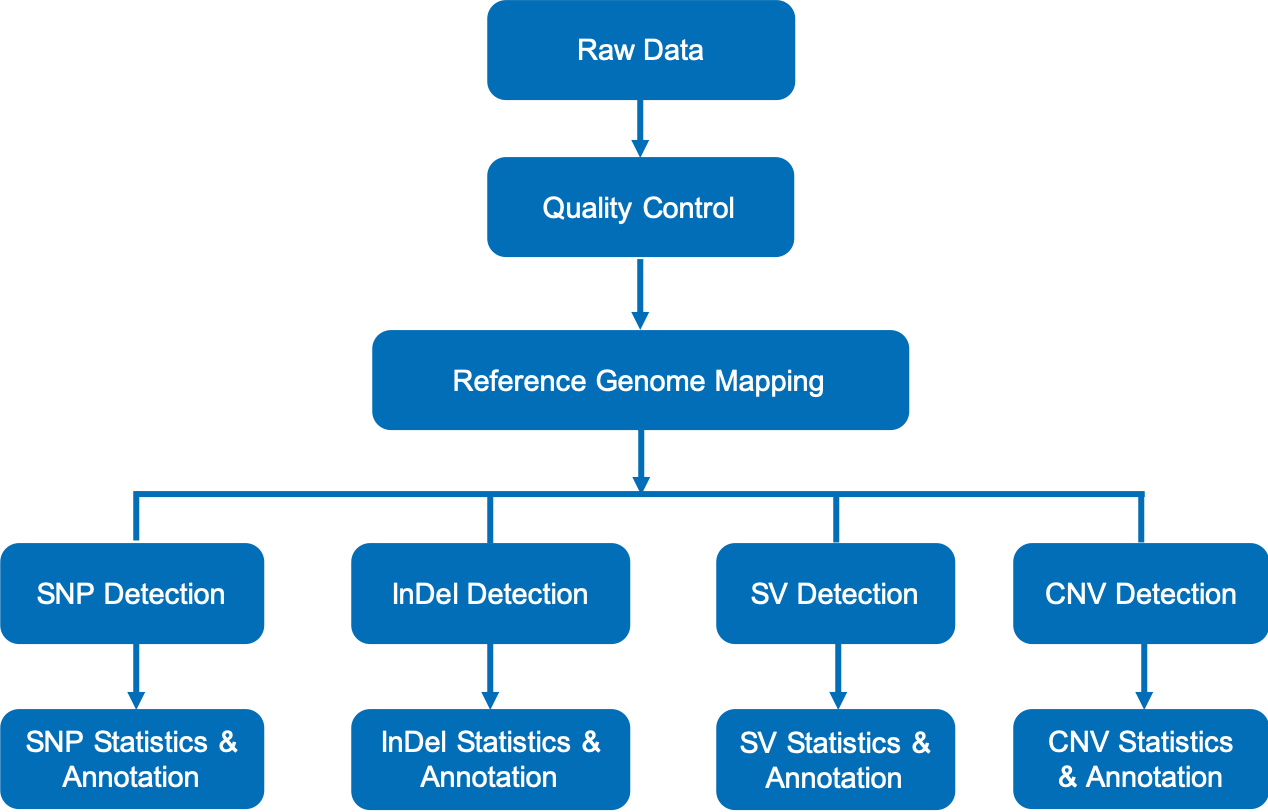

Supplement: S2 Data — (GZ) [file pgen.1012129.s008.gz › SupplementalDataSet1/03.Result_X202SC24112711-Z01-F001_C_elegans/report/src/images/bioinfoWorkflow.png]

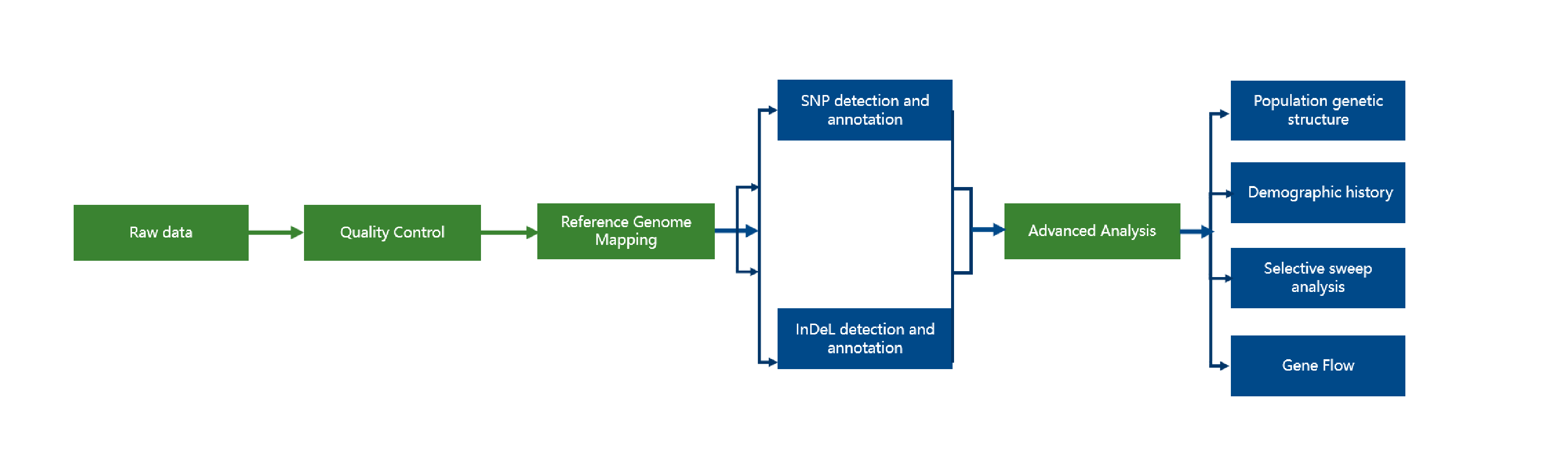

Supplement: S2 Data — (GZ) [file pgen.1012129.s008.gz › SupplementalDataSet1/03.Result_X202SC24112711-Z01-F001_C_elegans/report/src/images/flow_snpindel.adv.png]

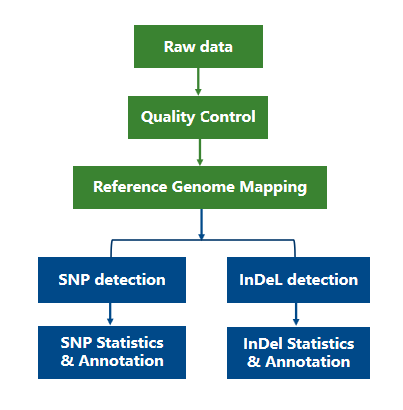

Supplement: S2 Data — (GZ) [file pgen.1012129.s008.gz › SupplementalDataSet1/03.Result_X202SC24112711-Z01-F001_C_elegans/report/src/images/flow_snpindel.png]

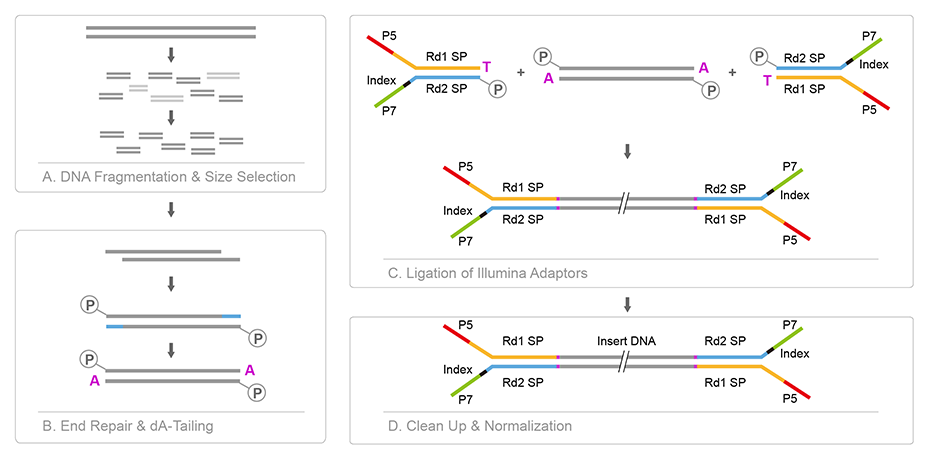

Supplement: S2 Data — (GZ) [file pgen.1012129.s008.gz › SupplementalDataSet1/03.Result_X202SC24112711-Z01-F001_C_elegans/report/src/images/LibPrep_PCR-free.png]
