# Supplementary material for: The pos-1 3′ untranslated region governs germline specification and proliferation to ensure reproductive robustness
Source: PLoS Genet. 2026 Apr 27;22(4):e1012129. doi: 10.1371/journal.pgen.1012129 (PMC13132445; doi:10.1371/journal.pgen.1012129)
Supplement: S1 Table — (DOCX) [file pgen.1012129.s006.docx]

**S1 Table:** Primers and Oligonucleotides used in this study.

| Primer Name | Purpose | Sequence |
| --- | --- | --- |
| DG4222_P1S1F_NEW | Sequencing of *pos-1* locus | 5' GATCATGATTTCGAGCGG 3′ |
| DG4222_P0S1R | Sequencing of *pos-1* locus | 5' GTCTGGGTAACGGGAGAAGC 3′ |
| DG4222_P0S2F_NEW | Sequencing of *pos-1* locus | 5' GCCATGCCAGAGGGATAC 3′ |
| DG4222_P1S2R_NEW | Sequencing of *pos-1* locus | 5' GCTTGTCGGCCATGATGTAG 3′ |
| DG4222_P2S3F | Sequencing of *pos-1* locus | 5' CTACAACTCCCACAACGTC 3′ |
| DG4222_P0S3R | Sequencing of *pos-1* locus | 5' CTCAGGAAATCGTTGTCAGC 3′ |
| DG4222_P0S4F | Sequencing of *pos-1* locus | 5' CGGAATCACCCACGGAATG 3′ |
| DG4222_P2S4R_NEW | Sequencing of *pos-1* locus | 5' GCATCACACAGAGCCGTC 3′ |
| DG4222_P3S5F_02 | Sequencing of *pos-1* locus | 5' CCGATGACCTTCGTGAAG 3′ |
| DG4222_P0S5R_NEW | Sequencing of *pos-1* locus | 5' GTGCGGCAGCATTCCTTG 3′ |
| DG4222_P0S6F_NEW | Sequencing of *pos-1* locus | 5' CACCAGCAAGGAATGCTG 3′ |
| DG4222_P3S6R | Sequencing of *pos-1* locus | 5' GGACTGCACCAGGTTATTG 3′ |
| pos-1_PCR004F | Sequencing of *pos-1* locus & PCR confirmation of *spr28* and *spr29* alleles | 5' CTACGGCTCAATTGGCAC 3′ |
| pos-1_PCR005R | Sequencing of *pos-1* locus & PCR confirmation of *spr28* and *spr29* alleles | 5' TAATACCGCAGAGGCACCG 3′ |
| CD.Cas9.BZLP9054.AB | crRNA used to generate *spr28 & spr29 alleles* | 5' UACUUUCUAUUUUUUACUUA 3′ |
| CD.Cas9.BZLP9054.AM | crRNA used to generate *spr28 & spr29 alleles* | 5' UCGUUAUGUGGACUCAUAAA 3′ |
| pos-1_ssODN_UTR-Delete | ssODN repair template (includes jump board sequence) used to generate strains WRM101 and WRM102 | 5' TCGAAATTTCTGATCTTGACTAACATGTACTTTCTTGTATCAGTTCGATATCTGACGGCTCATAAAATGAAATTTCTCGTAATCAGCATACAA 3′ |
| Linker1_pgl1_HA_F | Generation of pgl-1::mCherry reporter template | 5’ -TTAAATATTTATTTCAGTTTCATCCATTTCACATGTCCGGAGGGAGTGGA - 3’ |
| Linker2_pgl1_HA_R | Generation of pgl-1::mCherry reporter template | 5’ - CCACCGAAATCCACAATTTCTCGCTTGTTAGCCTCAGAACCTCCGCCACC - 3’ |
| CD.HC9.YJVG2684.AB | Guide RNA for the generation of spr20 | 5’ – GUUUCAUCCAUUUCACAUGG – 3’ |
| WRM81/82_Sequence_F1 | Sequencing and PCR confirmation of spr20 | 5’ – GAGTTTATGCGTTTCAAGGTG – 3’ |
| WRM81/82_Sequence_F1 | Sequencing and PCR confirmation of spr20 | 5’ – GAGTTTATGCGTTTCAAGGTG – 3’ |
| WRM81/82_Sequence_F3 | Sequencing and PCR confirmation of spr20 | 5’ – CTATGGGATGGGAAGCTTC – 3’ |
| WRM81/82_Sequence_R1 | Sequencing and PCR confirmation of spr20 | 5’ – GCCGGATGTTTAACATAAGC – 3’ |
| WRM81/82_Sequence_R2 | Sequencing and PCR confirmation of spr20 | 5’ – CCGTCTTCAGGGTACATTC – 3’ |
| WRM81/82_Sequence_R3 | Sequencing and PCR confirmation of spr20 | 5’ – CAATTCATCCATGCCACCT – 3’ |
