# Supplementary material for: The pos-1 3′ untranslated region governs germline specification and proliferation to ensure reproductive robustness
Source: PLoS Genet. 2026 Apr 27;22(4):e1012129. doi: 10.1371/journal.pgen.1012129 (PMC13132445; doi:10.1371/journal.pgen.1012129)
Supplement: S2 Data — (GZ) [file pgen.1012129.s008.gz › SupplementalDataSet1/03.Result_X202SC24112711-Z01-F001_C_elegans/report/src/result_tree.html]

Directory Tree


# Directory Tree

.
  
 |-- 01.OriginalData
  
 |-- 02.QualityControl
  
 | |-- CleanData\_QCsummary  
 | |-- ErrorRate  
 | |-- QualityDistribution  
 | `-- ReadsClassification  
 |-- 03.Mapping
  
 | |-- MapStat  
 | `-- Reference  
 |-- 04.SNP\_VarDetect
  
 | |-- DG4222  
 | |-- WRM101  
 | |-- WRM102  
 | |-- WRM103  
 | `-- picture\_in\_reports  
 |-- 05.InDel\_VarDetect
  
 | |-- DG4222  
 | |-- WRM101  
 | |-- WRM102  
 | |-- WRM103  
 | `-- picture\_in\_reports  
 |-- 06.SV\_VarDetect
  
 | |-- DG4222  
 | |-- WRM101  
 | |-- WRM102  
 | |-- WRM103  
 | `-- picture\_in\_reports  
 |-- 07.CNV\_VarDetect
  
 | |-- DG4222  
 | |-- WRM101  
 | |-- WRM102  
 | |-- WRM103  
 | `-- picture\_in\_reports  
 `-- 08.VarDetect\_Visualization

34 directories

---

tree v1.5.3 (c) 1996 - 2009 by Steve Baker and Thomas Moore   
HTML output hacked and copyleft (c) 1998 by Francesc Rocher   
Charsets / OS/2 support (c) 2001 by Kyosuke Tokoro
