# Supplementary material for: The pos-1 3′ untranslated region governs germline specification and proliferation to ensure reproductive robustness
Source: PLoS Genet. 2026 Apr 27;22(4):e1012129. doi: 10.1371/journal.pgen.1012129 (PMC13132445; doi:10.1371/journal.pgen.1012129)
Supplement: S1 Data — (GZ) [file pgen.1012129.s007.gz › SupplementalDataSet2/OneStopRNASeq/DESeq2.zip!/DESeq2/sample_PCA.labeled.pdf]

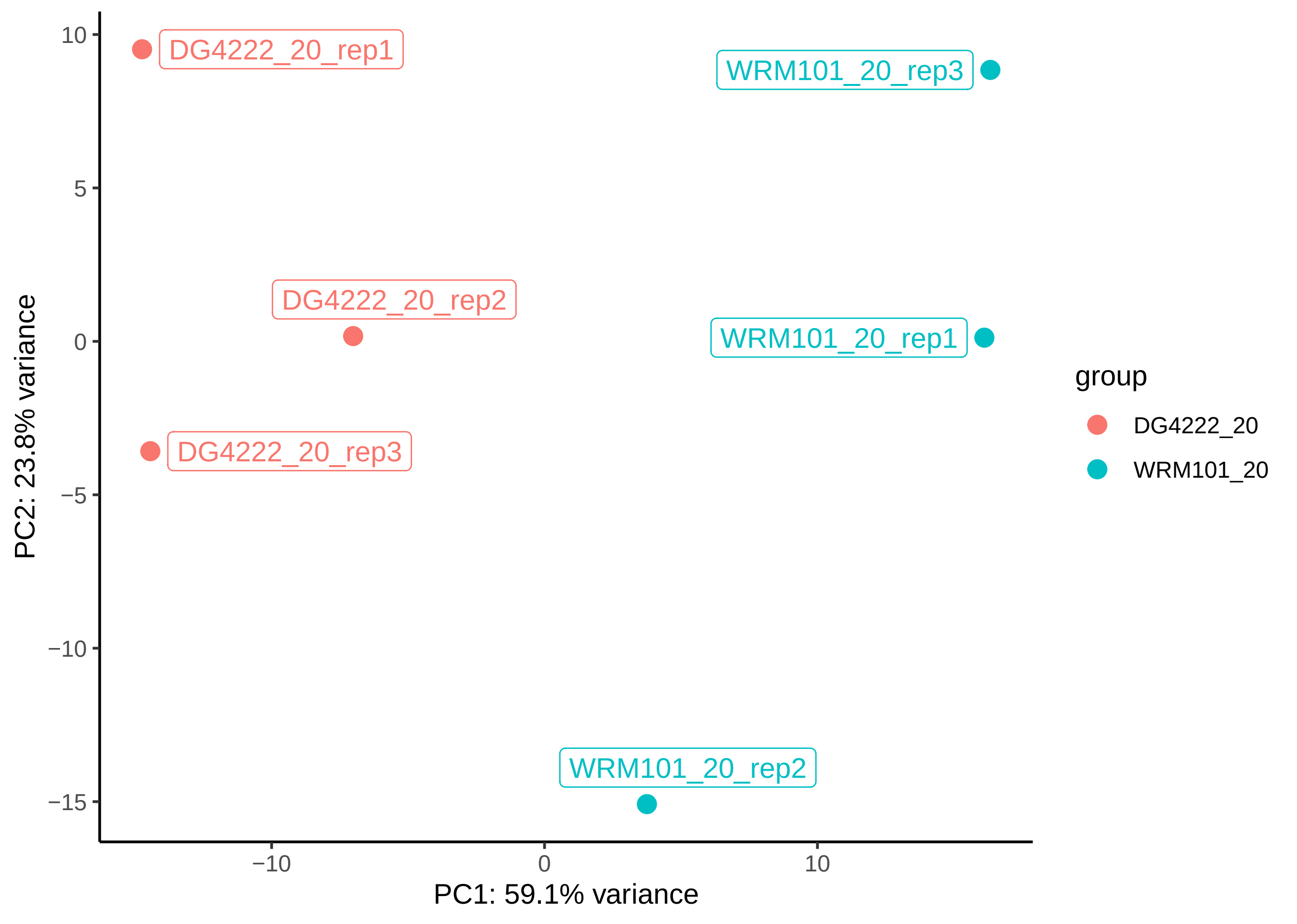

DG4222_20_rep1
WRM101_20_rep3
DG4222_20_rep2
WRM101_20_rep1
DG4222_20_rep3
WRM101_20_rep2
group
DG4222_20
WRM101_20
10
5
0
−5
−10
−15
−10
0
10
PC1: 59.1% variance
PC2: 23.8% variance
