# Supplementary material for: The pos-1 3′ untranslated region governs germline specification and proliferation to ensure reproductive robustness
Source: PLoS Genet. 2026 Apr 27;22(4):e1012129. doi: 10.1371/journal.pgen.1012129 (PMC13132445; doi:10.1371/journal.pgen.1012129)
Supplement: S1 Data — (GZ) [file pgen.1012129.s007.gz › SupplementalDataSet2/OneStopRNASeq/DESeq2.zip!/DESeq2/sample_poisson_distance.pdf]

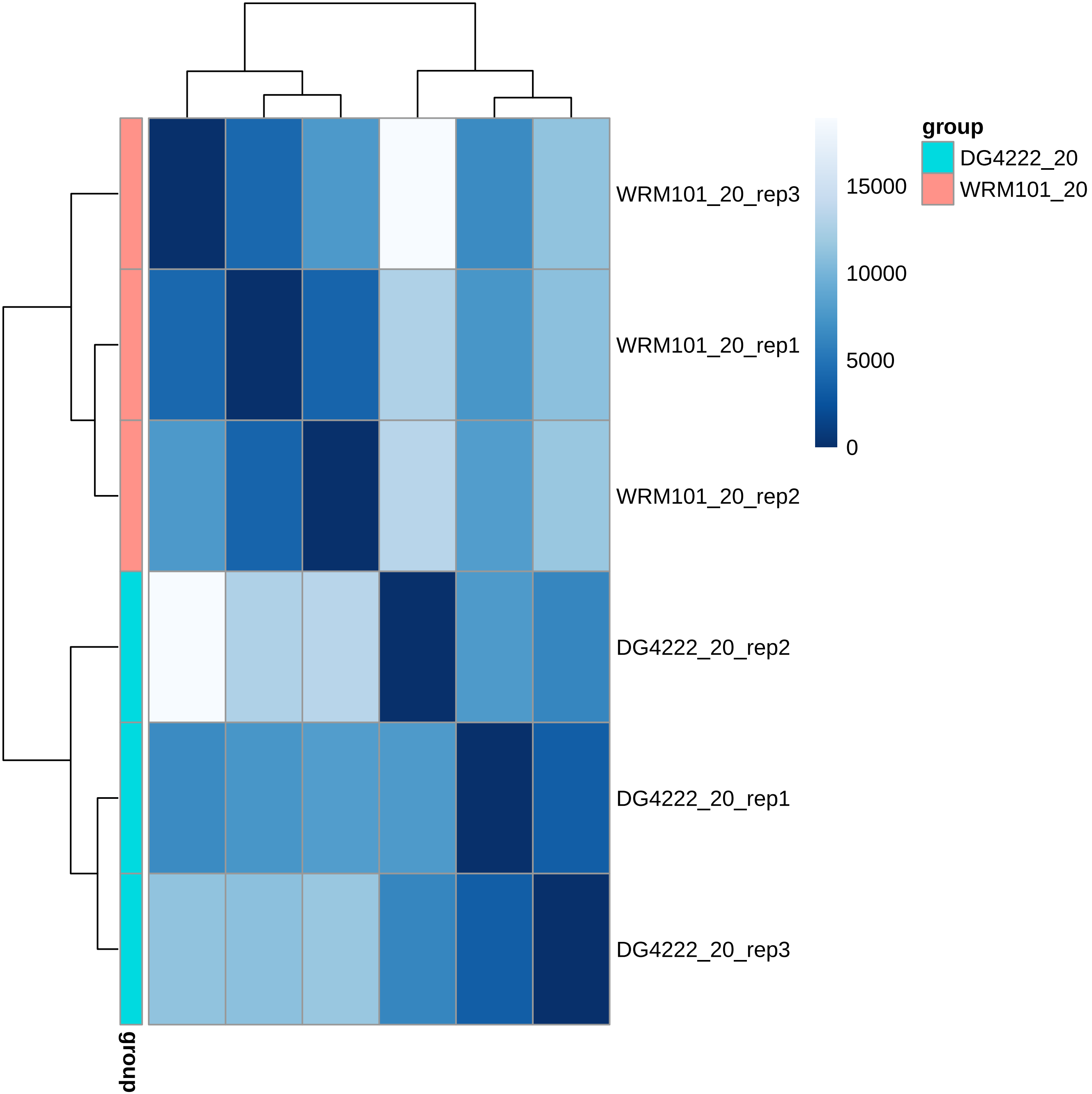

WRM101_20_rep3
WRM101_20_rep1
WRM101_20_rep2
DG4222_20_rep2
DG4222_20_rep1
DG4222_20_rep3
group
15000
10000
5000
0
group
DG4222_20
WRM101_20
