# Supplementary material for: The pos-1 3′ untranslated region governs germline specification and proliferation to ensure reproductive robustness
Source: PLoS Genet. 2026 Apr 27;22(4):e1012129. doi: 10.1371/journal.pgen.1012129 (PMC13132445; doi:10.1371/journal.pgen.1012129)
Supplement: S1 Data — (GZ) [file pgen.1012129.s007.gz › SupplementalDataSet2/OneStopRNASeq/DESeq2.zip!/DESeq2/WRM101_20_vs_DG4222_20.maplot.raw_lfc.pdf]

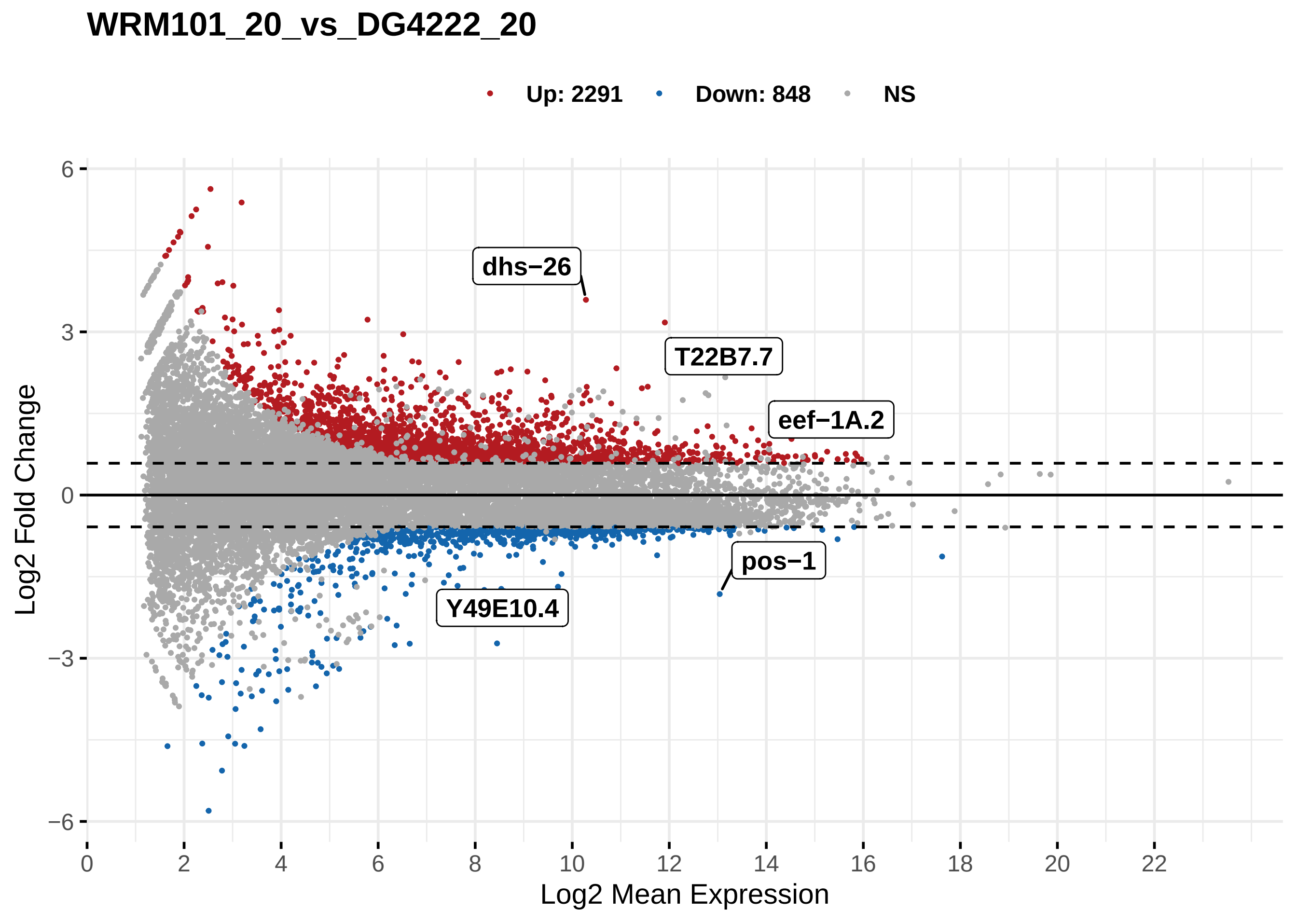
WRM101_20_vs_DG4222_20
Up: 2291
Down: 848
NS
dhs−26
T22B7.7
eef−1A.2
pos−1
Y49E10.4
Log2 Fold Change
Log2 Mean Expression
6
3
0
−3
−6
0
2
4
6
8
10
12
14
16
18
20
22
