# Supplementary material for: The pos-1 3′ untranslated region governs germline specification and proliferation to ensure reproductive robustness
Source: PLoS Genet. 2026 Apr 27;22(4):e1012129. doi: 10.1371/journal.pgen.1012129 (PMC13132445; doi:10.1371/journal.pgen.1012129)
Supplement: S1 Data — (GZ) [file pgen.1012129.s007.gz › SupplementalDataSet2/OneStopRNASeq/DESeq2.zip!/DESeq2/WRM101_20_vs_DG4222_20.LFC_shrunken.pdf]

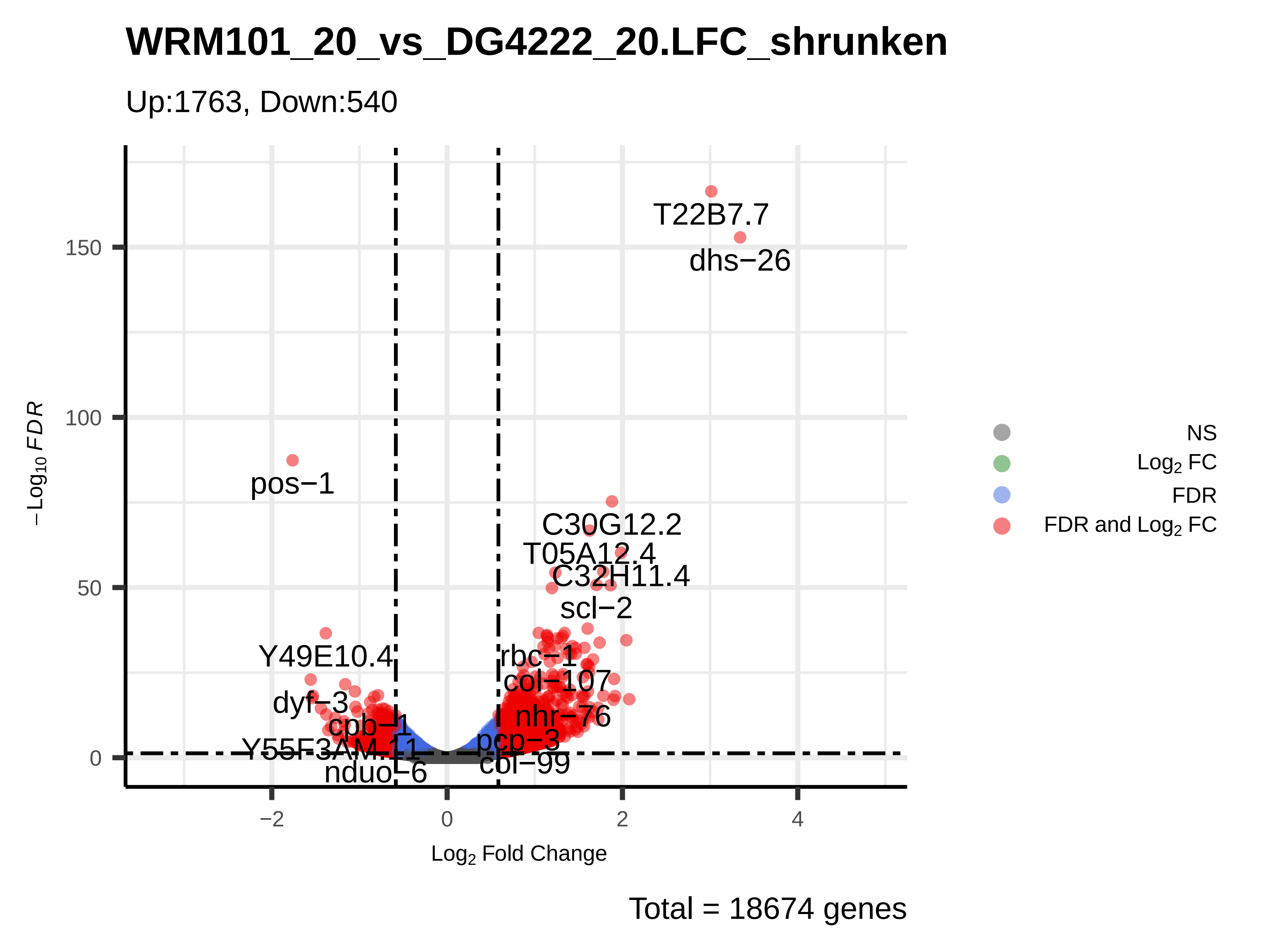

WRM101_20_vs_DG4222_20.LFC_shrunken
Up:1763, Down:540
T22B7.7
dhs−26
pos−1
C30G12.2
T05A12.4
C32H11.4
scl−2
Y49E10.4
rbc−1
col−107
dyf−3
cpb−1
nhr−76
Y55F3AM.11
pcp−3
col−99
nduo−6
150
100
50
0
−2
0
2
4
$-Log_{10}$ FDR
Log_2 Fold Change
NS
Log_2 FC
FDR
FDR and Log_2 FC
Total = 18674 genes
