# Supplementary material for: The pos-1 3′ untranslated region governs germline specification and proliferation to ensure reproductive robustness
Source: PLoS Genet. 2026 Apr 27;22(4):e1012129. doi: 10.1371/journal.pgen.1012129 (PMC13132445; doi:10.1371/journal.pgen.1012129)
Supplement: S1 Data — (GZ) [file pgen.1012129.s007.gz › SupplementalDataSet2/OneStopRNASeq/DESeq2.zip!/DESeq2/DESeq2.html]

DE Analysis and Standard Visualizations


Code 

- Show All Code
- Hide All Code

# DE Analysis and Standard Visualizations

## User ReadME:

- Please search for **“Warning”** in this report for
  possible problems before proceeding
- Normalized expression values, differentially expressed genes, and
  various types of plots such as heatmap and volcano plots are in the
  DESeq2 folder. For example, TPM.xlsx contains normalized expression
  values.
- Please use LFC\_shrunken for Log2FoldChange, LFC\_raw is not
  recommended
- Please use FDR (padj) instead of raw p-values for identifying
  significantly differentially expressed genes.

## Developer Log:

- blackSamples added in params
- manual dispersion estimation when DESeq() fails

## Input parameter extraction

```
max_fdr <- params$max_fdr
min_lfc <- params$min_lfc
indfilter <- params$indfilter
cookscutoff <- params$cookscutoff

countFile <- params$countFile
annoFile <- params$annoFile
metaFile <- params$metaFile
contrastFile <-params$contrastFile

blackSamplesStr <- gsub(" " , '', params$blackSamples)
blackSamples <- unlist(strsplit(blackSamplesStr, ','))

paste("Data file name:", countFile)
```

```
## [1] "Data file name: ../feature_count/counts.strict.txt"
```

```
paste("MetaData file name:", metaFile)
```

```
## [1] "MetaData file name: ../meta/meta.xlsx"
```

```
paste("Contrast file name:", contrastFile)
```

```
## [1] "Contrast file name: ../meta/contrast.de.xlsx"
```

```
paste("Annotation file name:", annoFile)
```

```
## [1] "Annotation file name: https://raw.githubusercontent.com/radio1988/OneStopRNAseq/dev/snakemake/workflow/resources/anno_tables/c_elegans.PRJNA13758.WS286.canonical_geneset.gtf.anno.txt"
```

```
paste("FDR cut-off:", max_fdr)
```

```
## [1] "FDR cut-off: 0.05"
```

```
paste("Log2FC cut-off:", min_lfc)
```

```
## [1] "Log2FC cut-off: 0.585"
```

```
cat('blackSamples to remove:', blackSamples)
```

```
## blackSamples to remove: sampleXXXXX sampleYYY
```

## Functions

```
readTable <- function(fname){
  if (str_ends(fname, '.txt')){
      df <-  read_delim(fname, delim ="\t", comment = '#') 
   } else if (str_ends(fname, '.csv')){
     df <-  read_delim(fname, delim =",", comment = '#') 
   }else if (str_ends(fname, '.xlsx')){
      df <- read.xlsx( fname, na.strings = 'NA', sheet=1)  # na term important
  } else{
      stop("file format not supported")
  }
  return (df)
}

get_density <- function(x, y, ...) {
  dens <- MASS::kde2d(x, y, ...)
  ix <- findInterval(x, dens$x)
  iy <- findInterval(y, dens$y)
  ii <- cbind(ix, iy)
  return(dens$z[ii])
}

volcanoplot <- function(res, anno, name='name', 
                        lfcthresh=2, sigthresh=0.05, 
                        labelsig=FALSE, xlim=100, ylim=1000, textcx=1) {
  # If Annotation file is wrong, No annotation match
  res0 <- res
  res<-merge(data.frame(res0), anno, by.x = 0, by.y=1, all.x=T, sort=F)
  if (sum(anno[, 1] %in% row.names(res0)) < 1) {
    warning(
      c("\nThe annotation file and the count filt does have no match in gene-id:\n", 
        "count table gene-id: ", head(row.names(res0), 1), 
        "\nanno table gene-id: ", anno[1:1, 1], 
        "gene-id rather than gene-name used for Volcano-plot"
      ))
    res$Name <- res[, 1]
  }
  
  # remove NA
  res$padj[is.na(res$padj)] <- 1
  # set lim on x, y
  res$padj[res$padj < 10^(-ylim) & !is.na(res$padj)] <- 10^(-ylim) # y-axis top value 50
  res$log2FoldChange[res$log2FoldChange > xlim] <- xlim
  res$log2FoldChange[res$log2FoldChange < -xlim] <- -xlim
  # show num_pos num_neg
  pos <- subset(res, padj<sigthresh & log2FoldChange>lfcthresh)
  neg <- subset(res, padj<sigthresh & log2FoldChange< -lfcthresh)
  pos.n <- dim(pos)[1]
  neg.n <- dim(neg)[1]
  
  EnhancedVolcano(res,
                       lab = res$Name,
                       #selectLab = as.character(res$Name[which(res$padj<labelcut)]), # mark top genes
                       #selectLab = c("FOS", "LDHA"), # mark selected genes
                       x = 'log2FoldChange',
                       y = 'padj',
                       title = name,
                       subtitle = paste("Up:", pos.n, ", Down:", neg.n, sep = ""),
                       xlab = bquote(~Log[2]~ "Fold Change"),
                       ylab = bquote(~-Log[10]~italic(FDR)),
                       pCutoff = max_fdr,
                       FCcutoff = min_lfc,
                       cutoffLineType = 'twodash',
                       cutoffLineWidth = 0.8,
                       legendLabels = c('NS', expression(Log[2]~FC),
                                        "FDR", expression(FDR~and~Log[2]~FC)),
                       caption = paste0('Total = ', nrow(res), ' genes'),
                       legendPosition = 'right',
                       legendLabSize = 10,
                       axisLabSize = 10,
                       legendIconSize = 3.0)
  ggsave(paste(name, "pdf", sep="."), width=8, height=6)
}

# Default plot
maplot <- function(res, anno, fname){
  maplotdat <- merge(anno, data.frame(res), by.x=1, by.y=0)
  ggmaplot(maplotdat, 
           main = name,
           genenames = as.vector(maplotdat$Name),
           fdr = max_fdr, fc = 2^min_lfc, size = 0.4,
           xlab = "Log2 Mean Expression",
           ylab = "Log2 Fold Change",
           palette = c("#B31B21", "#1465AC", "darkgray"),
           legend = "top", top = 20,
           font.label = c("bold", 11),label.rectangle = TRUE,
           font.legend = "bold",
           font.main = "bold",
           ggtheme = ggplot2::theme_minimal())
  ggsave(fname)
}

zscore <- function(matrix){
  return( t(scale(t(matrix))))
}

rename_num_vector_by_order <- function(l){
  # l have to be a vector of numbers
  # output vector of roman numbers ordered by appearance in the input vector
  # e.g. c(2,3,3,2,1) -> c(I, II, II, I, III)
  # test rename_num_vector_by_order(c(2,3,3,2,1))
  u <- unique(l)
  n=0
  for (i in u){
    n = n+1; 
    l <- replace(l, l==i, as.character(as.roman(n)))
  }
  return(l)
}


Heatmap <- function(df, nclass=2, fname="heatmap", main="title"){
  num_sig <- dim(df)[1]

  if (num_sig > 10000){
    warning("sorry, too many significant genes for heatmap, heatmap skipped")
    warning(num_sig)
    return (0)
  }
  
  if (num_sig < 20){CELL_HEIGHT = 20}else{CELL_HEIGHT = NA} # for plotting
  
  # prep group label
  my_sample_col <- data.frame(group = coldata[,'group'])
  row.names(my_sample_col) <- coldata[,'sample']
  
  if (num_sig < 1){
    print(paste("No sig DEG to create Heatmap for:", main) )
    return (0)
  }
  
  if (num_sig <= nclass){
    print(paste("Num-DEG less than num-class, there is no cut-tree for:", main))
    plot1 <- pheatmap(df,
                      annotation_col = my_sample_col,
                      main = main,
                      cluster_cols = F,
                      cluster_rows = F,
                      border_color = NA,
                      show_rownames = F,
                      breaks = seq(-3, 3, length.out = 100),
                      cellheight = CELL_HEIGHT,
                      filename = paste(fname, "pdf", sep = "."))
    plot2 <- pheatmap(df,
                      annotation_col = my_sample_col,
                      main = main,
                      cluster_cols = T,
                      cluster_rows = F,
                      border_color = NA,
                      show_rownames = F,
                      breaks = seq(-3, 3, length.out = 100),
                      cellheight = CELL_HEIGHT,
                      filename = paste(fname, "v2.pdf", sep = "."))
  }
  
  if (num_sig > nclass){
    p <- pheatmap(df, cluster_cols = F, cutree_rows = nclass) # Pre-plot to get hclust for cut-tree
    # cutree manually, print out gene classification (https://www.biostars.org/p/287512/)
    gene_classes <- sort(cutree(p$tree_row, k=nclass))
    gene_classes <- data.frame(gene_classes) 
    classification <- merge(gene_classes, df, by = 0)
    row.names(classification) <- classification$Row.names
    # Re-order original data (genes) to match ordering in heatmap (top-to-bottom)
    idx <- rownames(df[p$tree_row[["order"]],])
    classification <- classification[idx,] 
    # rename gene classes
    classification$gene_classes <- rename_num_vector_by_order(classification$gene_classes)
    write.xlsx(classification, paste(fname,"gene_class.xlsx", sep = "."), 
               keepNA = T, na.string = 'NA')
    # get class label
    annotation_row = data.frame(class = classification$gene_classes)
    row.names(annotation_row) <- row.names(classification)
    annotation_row$class <- as.character(annotation_row$class)
    # output final plot
    plot1 <- pheatmap(df,
                      annotation_col = my_sample_col,
                      main = main,
                      border_color = NA,
                      cluster_cols = F,
                      cutree_rows = nclass,
                      breaks = seq(-3, 3, length.out = 100),
                      show_rownames = F,
                      annotation_row = annotation_row,
                      cellheight = CELL_HEIGHT,
                      filename = paste(fname, "pdf", sep = "."))
    
    plot2 <- pheatmap(df,  # sample clustering
                      annotation_col = my_sample_col,
                      main = main,
                      border_color = NA,
                      cluster_cols = T,
                      cutree_rows = nclass,
                      breaks = seq(-3, 3, length.out = 100),
                      show_rownames = F,
                      annotation_row = annotation_row,
                      cellheight = CELL_HEIGHT,
                      filename = paste(fname, "v2.pdf", sep = "."))
  }
}


process_deseq_res <- function(res="lfcshrink.res", res2="results.res", name='name', anno='anno.df', norm_exp="tpm.df"){
    ## Summary
    print(name)
    print("\n>>> Summary using FDR cut-off only (LFC not used)")
    summary(res, alpha=max_fdr)
    
    print("\n>>> Summary using both FDR and LFC_shrunken cut-off")
    sig_idx <- res$padj<max_fdr & abs(res$log2FoldChange) > min_lfc
    sig_idx[is.na(sig_idx)] <- FALSE
    res_sig <- res[sig_idx,]
    print(table(sig_idx))
    
    up_idx <- res$padj<max_fdr & res$log2FoldChange > min_lfc
    up_idx[is.na(up_idx)] <- FALSE
    res_sig <- res[up_idx,]
    print(table(up_idx))
    
    down_idx <- res$padj<max_fdr & res$log2FoldChange < -min_lfc
    down_idx[is.na(down_idx)] <- FALSE
    res_sig <- res[down_idx,]
    print(table(down_idx))

    # Prep
    res.df <- as.data.frame(res)
    names(res.df)[2] <- "log2FoldChange_shrunken"
    names(res.df)[3] <- "lfcSE_shrunken"

    res2.df <- as.data.frame(res2)
    names(res2.df)[2] <- "log2FoldChange_raw"
    names(res2.df)[3] <- "lfcSE_raw"
    res2.df <- res2.df[, c(2,3)]

    resdata <- merge(res.df, res2.df, by=0, sort=F, all.x=T)
    resdata <- merge(anno, resdata, by.x=1, by.y=1, sort=F, all.y=T)
    resdata <- merge(resdata, norm_exp, by.x=1, by.y=0, all.x=T, sort=F)
    head(resdata)
    sig_idx <- resdata$padj<max_fdr & abs(resdata$log2FoldChange_shrunken) > min_lfc # important to put this line right before output sig.xlsx
    sig_idx[is.na(sig_idx)] <- FALSE
    resdata.sig <- resdata[sig_idx,]
    head(resdata.sig)

    ## Write results
    if (!TEST){
      write.xlsx(resdata, paste(name, 'deseq2.xlsx', sep = '.'), 
                 keepNA = T, na.string = 'NA')
      write.xlsx(resdata.sig, 
                 paste(name, 'deseq2.sig.FDR', max_fdr, 'LFC', min_lfc, 'xlsx', sep = '.'), 
                 keepNA = T, na.string = 'NA')
    }

    ## For GSEA
    rnk <- subset(resdata, select = c("Name","log2FoldChange_shrunken"))
    colnames(rnk) <- c("# Name","log2FoldChange_shrunken")
    rnk <- rnk[order(rnk$log2FoldChange_shrunken), ]
    rnk[, 1] <- toupper(rnk[, 1])
    write.table(rnk, 
                paste('rnk/', name, '.rnk', sep = ''), 
                row.names = F, quote = F, sep='\t')

    # # Corrlelation of Length and LFC
    # resdata.sig.cor <- cor.test(resdata.sig$Length, 
    #                        resdata.sig$log2FoldChange_shrunken, 
    #                        method = "spearman")
    # title <- paste("Spearman Cor:", format(resdata.sig.cor$estimate, digits=2, nsmall=2),
    #                "p-value:", format(resdata.sig.cor$p.value, digits=3, nsmall=3),
    #                sep = " ")
    # 
    # resdata.sig$density <- get_density(resdata.sig$Length, resdata.sig$log2FoldChange_shrunken, n = 100)
    # # set lim (careful, only after outputting excel)
    # resdata.sig$log2FoldChange_shrunken[resdata.sig$log2FoldChange_shrunken > 10] <- 10
    # resdata.sig$log2FoldChange_shrunken[resdata.sig$log2FoldChange_shrunken < -10] <- -10
    # resdata.sig$Length[resdata.sig$Length > 20000] <- 20000
    # ggplot(resdata.sig) + 
    #     geom_point(aes(Length, log2FoldChange_shrunken, color = density)) +
    #     scale_color_viridis() +
    #     ggtitle(paste(paste("Sig-DEG for", name), title, sep = "\n") ) + 
    #     ylim(-10, 10) +        
    #     xlim(0,20000)
    
    ##  Plots
    ggplot(data.frame(res), aes(x=pvalue))+
      geom_histogram(color="darkblue", fill="lightblue")
    ggsave(paste0(name, '.pvalue.pdf'))
    
    ggplot(data.frame(res), aes(x=padj))+
      geom_histogram(color="darkblue", fill="lightblue")
    ggsave(paste0(name, '.fdr.pdf'))
    
    maplot(res, anno, paste0(name, '.maplot.shrunken_lfc.pdf'))
    maplot(res2, anno, paste0(name, '.maplot.raw_lfc.pdf'))
    
    volcanoplot(res, anno, lfcthresh=min_lfc, sigthresh=max_fdr,
                textcx=.8,  name= paste(name, "LFC_shrunken", sep="."))
    volcanoplot(res2, anno, lfcthresh=min_lfc, sigthresh=max_fdr,
                textcx=.8,name= paste(name, "LFC_raw", sep="."))
    
    n1 <- dim(resdata.sig)[2]
    n2 <- dim(norm_exp)[2]
    zscore.df <- zscore(resdata.sig[, (n1-n2+1):n1])
    rownames(zscore.df) <- resdata.sig[,1]
    colnames(zscore.df) <- gsub (":TPM", "", colnames(zscore.df))
    colnames(zscore.df) <- gsub (":DESeq2NormalizedCount", "", colnames(zscore.df))
    Heatmap(zscore.df, nclass = 2,
            fname = paste(name, "heatmap", sep="."),
            main = paste(name, "LFC >", min_lfc, "FDR <", max_fdr ))
}
```

## Importing data

```
meta.df <- readTable(metaFile)
meta.df <- meta.df[!(meta.df[, 1] %in% blackSamples), ]

contrast.df <- readTable(contrastFile)
print(contrast.df)
```

```
##   contrast1
## 1 WRM101_20
## 2 DG4222_20
```

```
# read
paste('countFile:', countFile)
```

```
## [1] "countFile: ../feature_count/counts.strict.txt"
```

```
df <- readTable(countFile)
```

```
## Rows: 46926 Columns: 12
## ── Column specification ────────────────────────────────────────────────────────
## Delimiter: "\t"
## chr (5): Geneid, Chr, Start, End, Strand
## dbl (7): Length, mapped_reads/DG4222_20_rep1.bam, mapped_reads/DG4222_20_rep...
## 
## ℹ Use `spec()` to retrieve the full column specification for this data.
## ℹ Specify the column types or set `show_col_types = FALSE` to quiet this message.
```

```
names(df)[1] <- "GENE_ID"
df <- df %>%
  mutate(GENE_ID = gsub("\\.[^.]*$", "", GENE_ID))

if (sum(duplicated(df$GENE_ID)) > 0) {
  warning("count table gene_id not unique, has made it unique to run, but duplicated gene_id not valid for further analysis, please manually check duplicated gene_id")
  df$GENE_ID <- make.unique(df$GENE_ID)
}

df = column_to_rownames(df, var =  colnames(df)[1]) 

# auto-detect content format
featureCountsFormat <- F
cleanCountFormat <- F
osrCountFormat <- F
salmonCountFormat <- F

match_salmon_quant_format <- function (df){
  n_abundances <- sum(grepl('abundance', colnames(df)))
  n_counts <- sum(grepl('counts.', colnames(df)))
  n_lengths <- sum(grepl('length.', colnames(df)))
  if (n_abundances + n_counts + n_lengths == ncol(df) & n_abundances == n_counts & n_counts == n_lengths & ncol(df) >= 3){
    return (T)
  }else{
    return (F)
  }
}

if (colnames(df)[1] == "Chr" & colnames(df)[2] == "Start" & colnames(df)[3] == "End"){
  cat("featureCounts format table detected\n")
  featureCountsFormat <- T
  colnames(df) <- gsub("\\.bam$", "", colnames(df))
  colnames(df) <- gsub("sorted_reads.", "", colnames(df))
  colnames(df) <- gsub("mapped_reads.", "", colnames(df))
  df[, 6:ncol(df)] <- sapply(df[, 6:ncol(df)], as.integer)
  cts <- as.matrix(df[, 6:ncol(df)])
} else if (ncol(df) == dim(meta.df)[1]){
  cat("clean format table detected\n")
  cleanCountFormat<- T
  df[, 1:ncol(df)] <- sapply(df[, 1:ncol(df)], as.integer)
  cts <- as.matrix(df[, 1:ncol(df)])
} else if (colnames(df)[1] == "Name" & colnames(df)[2] == "Type" ) {
  cat("OSR COUNT.xlsx format detected\n")
  osrCountFormat <- T
  colnames(df) <- gsub(".COUNT", "", colnames(df))
  df[, 3:ncol(df)] <- sapply(df[, 3:ncol(df)], as.integer)
  cts <- as.matrix(df[, 3:ncol(df)])
} else if (match_salmon_quant_format(df)){
  cat("Salmon Quant CSV format detected\n")
  salmonCountFormat <- T
  df <- df[,grepl('count.', colnames(df))]
  colnames(df) <- gsub("counts\\.", "", colnames(df))
  df[,] <- sapply(df[,], as.integer)
  cts <- as.matrix(df)
}else {
  stop("COUNT file not in featureCounts output format, nor cleanCountFormat format, nor osrCount format. \nMay have different number of samples in meta-data and count-table if cleanCountFormat is provided\n")
}
```

```
## featureCounts format table detected
```

```
# remove blackSamples
df <- df[, !(colnames(df) %in% blackSamples)]
cts <- cts[, !(colnames(cts) %in% blackSamples)]

# check sample names
if (all(meta.df[[1]] %in% colnames(cts)) ){
  cat("all samples in meta.df included in count table")
} else {
  warning("count-table: ", colnames(cts), "\n")
  warning("meta-data: ",meta.df[[1]], "\n")
  stop("some samples in meta not included in count table")
}
```

```
## all samples in meta.df included in count table
```

```
# match order
cts_idx <- match(meta.df[[1]], colnames(cts))
cts <- cts[,cts_idx]

# Report Summary
print(paste("lib-size in millions:"))
```

```
## [1] "lib-size in millions:"
```

```
print(format(colSums(cts/1e6), digits=2))
```

```
## DG4222_20_rep1 DG4222_20_rep2 DG4222_20_rep3 WRM101_20_rep1 WRM101_20_rep2 
##           "27"           "42"           "41"           "35"           "44" 
## WRM101_20_rep3 
##           "48"
```

```
print(paste("Dim of input data:"))
```

```
## [1] "Dim of input data:"
```

```
print(dim(cts))
```

```
## [1] 46926     6
```

## Importing annotation

```
# from GitHub (must be raw, not zipped)
getAnnotation <- function(urlpath) {
  tmp <- tempfile()
  download.file(urlpath, destfile = tmp, method = 'auto')
  return(read.table(tmp, sep="\t", header = TRUE, quote=""))
}

paste("Getting data from", annoFile)
```

```
## [1] "Getting data from https://raw.githubusercontent.com/radio1988/OneStopRNAseq/dev/snakemake/workflow/resources/anno_tables/c_elegans.PRJNA13758.WS286.canonical_geneset.gtf.anno.txt"
```

```
if (grepl('https://', annoFile)){
  print("downloading remote annoFile")
  anno <- getAnnotation(annoFile)
}else{
  print("reading local annoFile")
  anno <- read.table(annoFile, sep = "\t", header = T, quote="")
}
```

```
## [1] "downloading remote annoFile"
```

```
anno <- anno[!duplicated(anno[1]), ] # assume first column is Gene ID
print("Dimention of annotation table: ")
```

```
## [1] "Dimention of annotation table: "
```

```
dim(anno)
```

```
## [1] 46926     6
```

```
head(anno)
```

```
##             Gene   Name           Type   Chr Start End
## 1 WBGene00014450 MTCE.1           tRNA MtDNA     1  55
## 2 WBGene00014451 MTCE.2           tRNA MtDNA    58 111
## 3 WBGene00010957 nduo-6 protein_coding MtDNA   113 549
## 4 WBGene00010958 ndfl-4 protein_coding MtDNA   549 783
## 5 WBGene00014452 MTCE.5           tRNA MtDNA   785 840
## 6 WBGene00014453 MTCE.6           tRNA MtDNA   842 896
```

```
if (sum(anno[, 1] %in% row.names(cts)) < 1){
  warning("!!! Annotation file and count file have no ID in common")
  warning("The results will be unannotated")
  warning("Please Double check Annotation file")
  print("count table ID:")
  print(row.names(cts)[1:2])
  print("anno table ID:")
  print(anno[1:2, 1])
  }
```

## COUNT output (without filtering)

```
count <- data.frame(cts)
colnames(count) <- paste(colnames(count),"COUNT", sep = ":")
count_out <- merge(anno, count, by.x=1, by.y=0, all.y=T, sort=F)
head(count_out)
```

```
##             Gene   Name           Type   Chr Start End DG4222_20_rep1:COUNT
## 1 WBGene00014450 MTCE.1           tRNA MtDNA     1  55                    0
## 2 WBGene00014451 MTCE.2           tRNA MtDNA    58 111                    0
## 3 WBGene00010957 nduo-6 protein_coding MtDNA   113 549                 1055
## 4 WBGene00010958 ndfl-4 protein_coding MtDNA   549 783                  100
## 5 WBGene00014452 MTCE.5           tRNA MtDNA   785 840                    0
## 6 WBGene00014453 MTCE.6           tRNA MtDNA   842 896                    0
##   DG4222_20_rep2:COUNT DG4222_20_rep3:COUNT WRM101_20_rep1:COUNT
## 1                    0                    0                    0
## 2                    0                    0                    0
## 3                 2150                  581                  240
## 4                  224                   80                   37
## 5                    0                    0                    0
## 6                    0                    0                    0
##   WRM101_20_rep2:COUNT WRM101_20_rep3:COUNT
## 1                    0                    0
## 2                    0                    0
## 3                  633                  454
## 4                   99                   71
## 5                    0                    0
## 6                    0                    0
```

```
if (!TEST){
  write.xlsx(count_out, 'COUNT.xlsx', keepNA = T, na.string = 'NA')
  print("saved in COUNT.xlsx")}
```

```
## [1] "saved in COUNT.xlsx"
```

## Filtering

```
min_rowsum <- params$min_rowsum
gene_filter <- rowSums(cts) >= min_rowsum  # default 10

min_colsum <- params$min_colsum
sample_filter <- colSums(cts) >= min_colsum

if (sum(gene_filter) > 2 & sum(sample_filter) >= 4){
  kept_samples <-  names(sample_filter)[sample_filter]
  meta.df <- meta.df[meta.df[[1]] %in% kept_samples,]
  group_count <- plyr::count(meta.df[[2]])
  cts <- cts[gene_filter, sample_filter]
  if (featureCountsFormat) {
    df <- df[gene_filter, c(rep(TRUE,5), sample_filter )]
  }else if (cleanCountFormat) {
    df <- df[gene_filter, sample_filter]
  } else if (osrCountFormat) {
    df <- df[gene_filter, c(rep(TRUE,2), sample_filter )]
  }    
  
  # match order
  meta.df <- meta.df[match(colnames(cts), meta.df[[1]]), ]
  cat(paste("Removed genes with less than ", min_rowsum, "reads/fragments across all samples", "\n"))
  cat(paste("Removed samples with less than ", min_colsum, "reads/fragments across all genes", "\n"))
  cat(paste("Data dim after filtering:"))
  dim(cts)
  print(group_count)
  boxplot(
            log10(cts+1), 
            las=2, 
            main = "Raw Gene Count Distribution After Filtering",
            ylab = 'log10(count+1)'
        )
}else{
  print("WARNING!!!: Library size too small, too few genes/samples would be left after filtering, so skipped filtering.")
  print("PLEASE interpret results with caution!!!")
  file.create('WARNING.LowCountSamples.Caution')
  sample_filter[1:length(sample_filter)] <- rep(TRUE, length(sample_filter))
  gene_filter[1:length(gene_filter)] <- rep(TRUE, length(gene_filter))
  kept_samples <-  names(sample_filter)[sample_filter]
  meta.df <- meta.df[meta.df[[1]] %in% kept_samples,]
  group_count <- plyr::count(meta.df[[2]])
  cts <- cts[gene_filter, sample_filter]
  if (featureCountsFormat) {
    df <- df[gene_filter, c(rep(TRUE,5), sample_filter )]
  }else if (cleanCountFormat) {
    df <- df[gene_filter, sample_filter]
  } else if (osrCountFormat) {
    df <- df[gene_filter, c(rep(TRUE,3), sample_filter )]
  }    
  
  # match order
  meta.df <- meta.df[match(colnames(cts), meta.df[[1]]), ]
  cat(paste("Removed genes with less than ", min_rowsum, "reads/fragments across all samples", "\n"))
  cat(paste("Removed samples with less than ", min_colsum, "reads/fragments across all genes", "\n"))
  cat(paste("Data dim after filtering:"))
  dim(cts)
  print(group_count)
  boxplot(
            log10(cts+1), 
            las=2, 
            main = "Raw Gene Count Distribution After Filtering",
            ylab = 'log10(count+1)'
        )
}
```

```
## Removed genes with less than  10 reads/fragments across all samples 
## Removed samples with less than  20000 reads/fragments across all genes 
## Data dim after filtering:          x freq
## 1 DG4222_20    3
## 2 WRM101_20    3
```

## COUNT calculation again (after filtering)

```
count <- data.frame(cts)
colnames(count) <- paste(colnames(count),"COUNT", sep = ":")
count_out <- merge(anno, count, by.x=1, by.y=0, all.y=T, sort=F)
head(count_out)
```

```
##             Gene    Name           Type   Chr Start  End DG4222_20_rep1:COUNT
## 1 WBGene00010957  nduo-6 protein_coding MtDNA   113  549                 1055
## 2 WBGene00010958  ndfl-4 protein_coding MtDNA   549  783                  100
## 3 WBGene00014454  MTCE.7           rRNA MtDNA   898 1593               474657
## 4 WBGene00010959  nduo-1 protein_coding MtDNA  1763 2635                 5844
## 5 WBGene00010960   atp-6 protein_coding MtDNA  2634 3235                 8625
## 6 WBGene00014459 MTCE.14           tRNA MtDNA  3307 3360                    5
##   DG4222_20_rep2:COUNT DG4222_20_rep3:COUNT WRM101_20_rep1:COUNT
## 1                 2150                  581                  240
## 2                  224                   80                   37
## 3               715936               503516               263387
## 4                12752                 8303                 6583
## 5                20018                12546                 4473
## 6                    8                    2                    2
##   WRM101_20_rep2:COUNT WRM101_20_rep3:COUNT
## 1                  633                  454
## 2                   99                   71
## 3               716067               381289
## 4                12135                 6834
## 5                20018                 6213
## 6                    7                    8
```

## TPM calculation

```
calculateTPM <- function(counts,len) {
  # michael's version
  # https://support.bioconductor.org/p/91218/
  x <- counts/len
  return(t(t(x)*1e6/colSums(x)))
}

if (featureCountsFormat ){
  tpm <- calculateTPM(cts, df$Length)
  tpm <- data.frame(tpm)
  colnames(tpm) <- paste(colnames(tpm),"TPM",  sep = ":")
  tpm_out <- merge(anno, tpm, by.x=1, by.y=0, all.y=T, sort=F)
  write.xlsx(tpm_out, 'TPM.xlsx', keepNA = T, na.string = 'NA')

  print("saved in TPM.xlsx")
} else {
  cat ("TPM not calculated if COUNT-table is not featureCount format\n")
}
```

```
## [1] "saved in TPM.xlsx"
```

## FPKM calculation

```
calculateFPKM <- function(counts,len) {
  x <- counts
  x <- t(t(x)*1e6/colSums(x))
  return (x/len*1e3)
}

if (featureCountsFormat ){
  fpkm <- calculateFPKM(cts, df$Length)
  fpkm <- data.frame(fpkm)
  colnames(fpkm) <- paste(colnames(fpkm), "FPKM", sep = ":")
  fpkm_out <- merge(anno, fpkm, by.x=1, by.y=0, all.y=T, sort=F)
  # head(fpkm_out)
  # tail(fpkm_out)
  if (!TEST){
    write.xlsx(fpkm_out, 'FPKM.xlsx', keepNA = T, na.string = 'NA')
    print("saved in FPKM.xlsx")
    print("Recommend to use TPM, rather than FPKM")}
} else {
  cat ("FPKM not calculated if COUNT-table is not featureCount format\n")
}
```

```
## [1] "saved in FPKM.xlsx"
## [1] "Recommend to use TPM, rather than FPKM"
```

## Design matrix extracted from meta-data

```
meta_idx <- match(colnames(cts), meta.df[[1]])
meta_idx <- meta_idx[!is.na(meta_idx)]
meta.df <- meta.df[meta_idx, ]
cts <- cts[, colnames(cts) %in% meta.df[[1]]]


sample <- factor(meta.df[[1]])
batch <- factor(meta.df[[3]])
group <- factor(meta.df[[2]])

coldata <- data.frame(row.names=colnames(cts), 
                      sample,
                      group,
                      batch
                      )
coldata
```

```
##                        sample     group batch
## DG4222_20_rep1 DG4222_20_rep1 DG4222_20     1
## DG4222_20_rep2 DG4222_20_rep2 DG4222_20     1
## DG4222_20_rep3 DG4222_20_rep3 DG4222_20     1
## WRM101_20_rep1 WRM101_20_rep1 WRM101_20     1
## WRM101_20_rep2 WRM101_20_rep2 WRM101_20     1
## WRM101_20_rep3 WRM101_20_rep3 WRM101_20     1
```

```
save.image()
```

## Model fitting

```
if (length(levels(batch)) > 1){
  dds <- DESeqDataSetFromMatrix(countData = cts, 
                                colData = coldata, 
                                design = ~  0 + group + batch)
}else{
  dds <- DESeqDataSetFromMatrix(countData = cts, 
                                colData = coldata, 
                                design = ~  0 + group)  # converted to alph-order
}
dds
```

```
## class: DESeqDataSet 
## dim: 18674 6 
## metadata(1): version
## assays(1): counts
## rownames(18674): WBGene00010957 WBGene00010958 ... WBGene00201913
##   WBGene00007064
## rowData names(0):
## colnames(6): DG4222_20_rep1 DG4222_20_rep2 ... WRM101_20_rep2
##   WRM101_20_rep3
## colData names(3): sample group batch
```

```
if (is.error(dds <-DESeq(dds))){
  cat("using manual dispersion estimation")
  dds <- estimateSizeFactors(dds)
  dds <- estimateDispersionsGeneEst(dds)
  dispersions(dds) <- mcols(dds)$dispGeneEst
  dds <- nbinomWaldTest(dds)
}
```

```
## estimating size factors
```

```
## estimating dispersions
```

```
## gene-wise dispersion estimates
```

```
## mean-dispersion relationship
```

```
## final dispersion estimates
```

```
## fitting model and testing
```

```
resultsNames(dds)
```

```
## [1] "groupDG4222_20" "groupWRM101_20"
```

```
#saveRDS(dds, file = 'deseq2.dds.rds')
```

## Save DESeq2 normalized counts

```
normalized_counts <- counts(dds, normalized=TRUE)
normalized_counts <- data.frame(normalized_counts)

boxplot(
          log10(normalized_counts + 1), 
          las=2, 
          main = "Normazlied Gene Count Distribution",
          ylab = 'log10(norm+1)'
      )
```

```
colnames(normalized_counts) <- paste(colnames(normalized_counts),"DESeq2NormalizedCount",  sep = ":")
normalized_counts_out <- merge(anno, normalized_counts, by.x=1, by.y=0, all.y=T, sort=F)
if (!TEST){
  write.xlsx(normalized_counts_out, 'DESeq2NormalizedCounts.xlsx', keepNA = T, na.string = 'NA')
  print("saved in DESeq2NormalizedCounts.xlsx")
}
```

```
## [1] "saved in DESeq2NormalizedCounts.xlsx"
```


### Histogram of Log10(Counts)

```
log1p_count <- log10(counts(dds) + 1)
hist(log1p_count, 
     main = 'Histogram of log10(count + 1)', 
     xlab = "log10(count+1)",
     100) # by default, use non-normalized data by counts function
```

### Dispersion plot

```
plotDispEsts(dds, main="Dispersion plot")
```

### Sample PCA plot

```
plotQC_PCA <- function(dds, fname='pca.pdf') {
  vsd <- varianceStabilizingTransformation(dds) # fixed num < num(rowsum>5)
  pcaData <- plotPCA(vsd, intgroup = 'group', returnData=TRUE) # labeling fixed
  percentVar <- round(100 * attr(pcaData, 'percentVar'), 1)
  if (length(levels(batch)) > 1){
    ggplot(pcaData, aes(PC1, PC2, color = group, shape = batch)) +
    geom_point(size = 3) +
    xlab(paste0("PC1: ", percentVar[1], "% variance")) +
    ylab(paste0("PC2: ", percentVar[2], "% variance")) +
    geom_label_repel(aes(label = sample),
                      box.padding = 0.35,
                      point.padding = 1,
                      segment.color = 'grey50',
                      segment.alpha = 0.5,
                      show.legend = FALSE) + # if TRUE, legend display might not be correct
    theme_classic()
    ggsave(fname)

  }else{
    ggplot(pcaData, aes(PC1, PC2, color = group)) +
    geom_point(size = 3) +
    xlab(paste0("PC1: ", percentVar[1], "% variance")) +
    ylab(paste0("PC2: ", percentVar[2], "% variance")) +
    geom_label_repel(aes(label = sample),
                      box.padding = 0.35,
                      point.padding = 1,
                      segment.color = 'grey50',
                      segment.alpha = 0.5,
                      max.overlaps = 20,
                      show.legend = FALSE) + # if TRUE, legend display might not be correct
    theme_classic()
    ggsave(fname)
  }
}

plotQC_PCA_no_label <- function(dds, fname='pca.pdf') {
  vsd <- varianceStabilizingTransformation(dds) # fixed num < num(rowsum>5)
  pcaData <- plotPCA(vsd, intgroup = 'group', returnData=TRUE) # labeling fixed
  percentVar <- round(100 * attr(pcaData, 'percentVar'), 1)
    if (length(levels(batch)) > 1){
        ggplot(pcaData, aes(PC1, PC2, color = group, shape = batch)) +
        geom_point(size = 3) +
        xlab(paste0("PC1: ", percentVar[1], "% variance")) +
        ylab(paste0("PC2: ", percentVar[2], "% variance")) +
        theme_classic()
        ggsave(fname)
    }else{
        ggplot(pcaData, aes(PC1, PC2, color = group)) +
        geom_point(size = 3) +
        xlab(paste0("PC1: ", percentVar[1], "% variance")) +
        ylab(paste0("PC2: ", percentVar[2], "% variance")) +
        theme_classic()
        ggsave(fname)
    }
}

plotQC_PCA_no_label(dds, "sample_PCA.pdf")
```

```
## Saving 7 x 5 in image
```

```
if (max(unlist(lapply(meta.df[[1]], nchar))) <= 36){
  plotQC_PCA(dds, "sample_PCA.labeled.pdf")
}
```

```
## Saving 7 x 5 in image
```

### Sample heatmap (Poisson Distance Based)

```
PoissonDistanceHeatmap <-  function(){
  # dist matrix
  poisd <- PoissonDistance(t(counts(dds)))
  samplePoisDistMatrix <- as.matrix( poisd$dd ) 
  rownames(samplePoisDistMatrix) <- coldata$sample
  colnames(samplePoisDistMatrix) <- NULL

  # sample_col
  my_sample_col <- data.frame(group = coldata[,'group'])
  row.names(my_sample_col) <- coldata[,'sample']
  
  pheatmap(samplePoisDistMatrix,
           annotation_row = my_sample_col,
           
           clustering_distance_rows=poisd$dd,
           clustering_distance_cols=poisd$dd,
           clustering_method='complete', 
           legend = T, 
           col=colorRampPalette( rev(brewer.pal(9, "Blues")) )(255), 
           
           filename = "sample_poisson_distance.pdf")
}
PoissonDistanceHeatmap()
```

## DE analysis

```
parse_name <- function(name){
  name <- gsub(" ", "", name)
  name <- gsub(";$", "", name)
  names <- strsplit(name, ";") [[1]]
  name <- gsub(";", ".", name)
  return (list(name=name, names=names))
}

for (i in 1:dim(contrast.df)[2]){
  # parse 
  name1 <- parse_name(contrast.df[1,i])
  name2 <- parse_name(contrast.df[2, i])
  name <- paste(name1$name, name2$name, sep = "_vs_")
  if (nchar(name) > 100) {name = paste0('contrast', i)}
  print(paste(">>>", i, name))
  
  # check group_count for all groups in each contrast
  groups <- unique(c(name1$names, name2$names))
  
  with_sample <- groups %in% group_count[[1]]  # count >= 1
  if (!all(with_sample)){
    print(paste('some groups in', name, 'has no filtered samples, skipped !!!'))
    print(group_count)
    next
    }

  group_with_reps <-  group_count[[1]][group_count$freq >= 2]
  
  with_reps1 <- any(name1$names %in% group_with_reps)
  with_reps2 <- any(name2$names %in% group_with_reps)
  
  if (!any(with_reps1, with_reps2)) {
    print(paste('no groups in', name, 'has reps, skipped !!!'))
    print(group_count)
    next
  }

  # analysis
  gsub("group", "", resultsNames(dds))
  poss <- match(name1$names, gsub("group", "", resultsNames(dds)))
  negs <- match(name2$names, gsub("group", "", resultsNames(dds)))
  contrast <- rep(0, length(resultsNames(dds)))
  contrast[poss] <- 1/length(poss)
  contrast[negs] <- -1/length(negs)
  print(data.frame(resNames=gsub("group", "", resultsNames(dds)), 
                   contrast=contrast))
  
  res <- lfcShrink(dds, contrast = contrast, type = 'ashr')
  res2 <- results(dds, contrast = contrast, 
                  independentFilter=indfilter, cooksCutoff=cookscutoff)

  process_deseq_res(res = res, res2=res2, name = name, anno = anno, norm_exp = normalized_counts)

}
```

```
## [1] ">>> 1 WRM101_20_vs_DG4222_20"
##    resNames contrast
## 1 DG4222_20       -1
## 2 WRM101_20        1
```

```
## using 'ashr' for LFC shrinkage. If used in published research, please cite:
##     Stephens, M. (2016) False discovery rates: a new deal. Biostatistics, 18:2.
##     https://doi.org/10.1093/biostatistics/kxw041
```

```
## [1] "WRM101_20_vs_DG4222_20"
## [1] "\n>>> Summary using FDR cut-off only (LFC not used)"
## 
## out of 18674 with nonzero total read count
## adjusted p-value < 0.05
## LFC > 0 (up)       : 3414, 18%
## LFC < 0 (down)     : 4390, 24%
## outliers [1]       : 0, 0%
## low counts [2]     : 2173, 12%
## (mean count < 4)
## [1] see 'cooksCutoff' argument of ?results
## [2] see 'independentFiltering' argument of ?results
## 
## [1] "\n>>> Summary using both FDR and LFC_shrunken cut-off"
## sig_idx
## FALSE  TRUE 
## 16371  2303 
## up_idx
## FALSE  TRUE 
## 16911  1763 
## down_idx
## FALSE  TRUE 
## 18134   540
```

```
## Saving 7 x 5 in image
```

```
## `stat_bin()` using `bins = 30`. Pick better value with `binwidth`.
```

```
## Saving 7 x 5 in image
```

```
## `stat_bin()` using `bins = 30`. Pick better value with `binwidth`.
```

```
## Warning: Removed 2173 rows containing non-finite values (stat_bin).
```

```
## Saving 7 x 5 in image
```

```
## Warning: ggrepel: 14 unlabeled data points (too many overlaps). Consider
## increasing max.overlaps
```

```
## Saving 7 x 5 in image
```

```
## Warning: ggrepel: 15 unlabeled data points (too many overlaps). Consider
## increasing max.overlaps
```

## Log

```
save.image()
sessionInfo()
```

```
## R version 4.0.2 (2020-06-22)
## Platform: x86_64-conda_cos6-linux-gnu (64-bit)
## Running under: Red Hat Enterprise Linux 8.10 (Ootpa)
## 
## Matrix products: default
## BLAS/LAPACK: /pi/mccb-umw/shared/OneStopRNAseq/conda/osr_envs/1a2581c88198fb51bd0aae6d10b1748f_/lib/libopenblasp-r0.3.10.so
## 
## locale:
##  [1] LC_CTYPE=en_US.UTF-8       LC_NUMERIC=C              
##  [3] LC_TIME=en_US.UTF-8        LC_COLLATE=en_US.UTF-8    
##  [5] LC_MONETARY=en_US.UTF-8    LC_MESSAGES=en_US.UTF-8   
##  [7] LC_PAPER=en_US.UTF-8       LC_NAME=C                 
##  [9] LC_ADDRESS=C               LC_TELEPHONE=C            
## [11] LC_MEASUREMENT=en_US.UTF-8 LC_IDENTIFICATION=C       
## 
## attached base packages:
## [1] parallel  stats4    stats     graphics  grDevices utils     datasets 
## [8] methods   base     
## 
## other attached packages:
##  [1] berryFunctions_1.22.5       PoiClaClu_1.0.2.1          
##  [3] pheatmap_1.0.12             RColorBrewer_1.1-3         
##  [5] MASS_7.3-58.1               ashr_2.2-54                
##  [7] ggpubr_0.4.0                openxlsx_4.2.5             
##  [9] forcats_0.5.2               stringr_1.4.1              
## [11] dplyr_1.0.10                purrr_0.3.4                
## [13] readr_2.1.2                 tidyr_1.2.1                
## [15] tibble_3.1.8                tidyverse_1.3.2            
## [17] EnhancedVolcano_1.8.0       ggrepel_0.9.1              
## [19] ggplot2_3.3.6               DESeq2_1.30.1              
## [21] SummarizedExperiment_1.20.0 Biobase_2.50.0             
## [23] MatrixGenerics_1.2.1        matrixStats_0.62.0         
## [25] GenomicRanges_1.42.0        GenomeInfoDb_1.26.4        
## [27] IRanges_2.24.1              S4Vectors_0.28.1           
## [29] BiocGenerics_0.36.0         BiocManager_1.30.18        
## 
## loaded via a namespace (and not attached):
##   [1] googledrive_2.0.0      ggbeeswarm_0.6.0       colorspace_2.0-3      
##   [4] ggsignif_0.6.3         ellipsis_0.3.2         XVector_0.30.0        
##   [7] fs_1.5.2               farver_2.1.1           bit64_4.0.5           
##  [10] AnnotationDbi_1.52.0   fansi_1.0.3            lubridate_1.8.0       
##  [13] xml2_1.3.3             splines_4.0.2          extrafont_0.18        
##  [16] cachem_1.0.6           geneplotter_1.68.0     knitr_1.40            
##  [19] jsonlite_1.8.0         broom_1.0.1            Rttf2pt1_1.3.10       
##  [22] annotate_1.68.0        dbplyr_2.2.1           compiler_4.0.2        
##  [25] httr_1.4.4             backports_1.4.1        assertthat_0.2.1      
##  [28] Matrix_1.4-1           fastmap_1.1.0          gargle_1.2.1          
##  [31] cli_3.4.1              htmltools_0.5.3        tools_4.0.2           
##  [34] gtable_0.3.1           glue_1.6.2             GenomeInfoDbData_1.2.4
##  [37] maps_3.4.0             Rcpp_1.0.9             carData_3.0-5         
##  [40] cellranger_1.1.0       jquerylib_0.1.4        vctrs_0.4.1           
##  [43] ggalt_0.4.0            extrafontdb_1.0        xfun_0.33             
##  [46] rvest_1.0.3            irlba_2.3.5            lifecycle_1.0.2       
##  [49] rstatix_0.7.0          XML_3.99-0.6           googlesheets4_1.0.1   
##  [52] zlibbioc_1.36.0        scales_1.2.1           vroom_1.5.7           
##  [55] ragg_0.4.0             hms_1.1.2              proj4_1.0-10.1        
##  [58] yaml_2.3.5             memoise_2.0.1          ggrastr_1.0.1         
##  [61] sass_0.4.2             SQUAREM_2021.1         stringi_1.5.3         
##  [64] RSQLite_2.2.8          highr_0.9              genefilter_1.72.0     
##  [67] zip_2.2.1              BiocParallel_1.24.1    truncnorm_1.0-8       
##  [70] systemfonts_1.0.4      rlang_1.0.6            pkgconfig_2.0.3       
##  [73] bitops_1.0-7           invgamma_1.1           evaluate_0.16         
##  [76] lattice_0.20-45        labeling_0.4.2         bit_4.0.4             
##  [79] tidyselect_1.1.2       plyr_1.8.7             magrittr_2.0.3        
##  [82] R6_2.5.1               generics_0.1.3         DelayedArray_0.16.3   
##  [85] DBI_1.1.3              pillar_1.8.1           haven_2.5.0           
##  [88] withr_2.5.0            mixsqp_0.3-43          abind_1.4-5           
##  [91] survival_3.4-0         RCurl_1.98-1.3         ash_1.0-15            
##  [94] car_3.1-0              crayon_1.5.1           modelr_0.1.9          
##  [97] KernSmooth_2.23-18     utf8_1.2.2             tzdb_0.3.0            
## [100] rmarkdown_2.16         locfit_1.5-9.4         grid_4.0.2            
## [103] readxl_1.4.1           blob_1.2.3             reprex_2.0.2          
## [106] digest_0.6.29          xtable_1.8-4           textshaping_0.1.2     
## [109] munsell_0.5.0          beeswarm_0.4.0         vipor_0.4.5           
## [112] bslib_0.4.0
```
