# Supplementary material for: The pos-1 3′ untranslated region governs germline specification and proliferation to ensure reproductive robustness
Source: PLoS Genet. 2026 Apr 27;22(4):e1012129. doi: 10.1371/journal.pgen.1012129 (PMC13132445; doi:10.1371/journal.pgen.1012129)
Supplement: S1 Data — (GZ) [file pgen.1012129.s007.gz › SupplementalDataSet2/OneStopRNASeq/fastqc.zip!/fastqc/details_raw/DG4222_20_rep1.R2_fastqc.html]

DG4222\_20\_rep1.R2.fastq.gz FastQC Report 

FastQC Report

Wed 12 Feb 2025  
DG4222\_20\_rep1.R2.fastq.gz

## Summary

- Basic Statistics
- Per base sequence quality
- Per tile sequence quality
- Per sequence quality scores
- Per base sequence content
- Per sequence GC content
- Per base N content
- Sequence Length Distribution
- Sequence Duplication Levels
- Overrepresented sequences
- Adapter Content

## Basic Statistics

| Measure | Value |
| --- | --- |
| Filename | DG4222\_20\_rep1.R2.fastq.gz |
| File type | Conventional base calls |
| Encoding | Sanger / Illumina 1.9 |
| Total Sequences | 38467042 |
| Total Bases | 1.9 Gbp |
| Sequences flagged as poor quality | 0 |
| Sequence length | 35-51 |
| %GC | 49 |

## Per base sequence quality

## Per tile sequence quality

## Per sequence quality scores

## Per base sequence content

## Per sequence GC content

## Per base N content

## Sequence Length Distribution

## Sequence Duplication Levels

## Overrepresented sequences

| Sequence | Count | Percentage | Possible Source |
| --- | --- | --- | --- |
| GGGGGGGGGGGGGGGGGGGGGGGGGGGGGGGGGGGGGGGGGGGGGGGGGG | 449663 | 1.16895653167197 | No Hit |
| CCCCACTCTTCTCGAATCAGTTCAGTCCCGGATAGCGACGGGCGGTGTGT | 108717 | 0.28262375880110563 | No Hit |
| GTCGCAACGGCGGAGTATAGGTCTCTCGCTTAAGCGCCATCCATTTTCAG | 100240 | 0.26058671212618845 | No Hit |
| CCGACATTGAACCAGAGTGATCTGGCGTTGGCAAGCGTCGCAGAATTCAC | 97350 | 0.25307378716564694 | No Hit |
| GGGTATTGTAATTATTGCCCTTAAACGAGGAATGCCTAGTAAGTGTGAGT | 82476 | 0.2144069200849912 | No Hit |
| CGAAAATTTCTTGCTTCCCGGTGTCGGGAGGCATCTCTATCTCGTGGCAA | 75933 | 0.19739755398920458 | No Hit |
| CAAACGTGGAGTTAAGGTGCCTAACTTCTCGCTCATGAGACCCCATAAA | 67947 | 0.17663692466917524 | No Hit |
| GTGATTTAGCGCCCCGACATTGAACCAGAGTGATCTGGCGTTGGCAAGCG | 64228 | 0.1669689080850043 | No Hit |
| GGAACAGCGGGTTCAAACATTTGGTTCATAGACTTGATCGACAGATCAAT | 62136 | 0.16153048627965727 | No Hit |
| CTCGCCTTGTCAAGGCGAAAATTTCTTGCTTCCCGGTGTCGGGAGGCATC | 52716 | 0.13704199038751147 | No Hit |
| GAAAATTTCTTGCTTCCCGGTGTCGGGAGGCATCTCTATCTCGTGGCAAC | 49192 | 0.12788090126607604 | No Hit |
| ATAAGCTCTCGTGTTGCCACGAGATAGAGATGCCTCCCGACACCGGGAAG | 45640 | 0.11864702256024781 | No Hit |
| GGAAGCTCCAGCCGAGCCTACGCTCATAGCCTTCAACGCAACCGCCACGA | 43979 | 0.11432904042894694 | No Hit |
| GCGGAGTATAGGTCTCTCGCTTAAGCGCCATCCATTTTCAGGGCTAGTTG | 42216 | 0.1097458962402152 | No Hit |
| CCCACTCTTCTCGAATCAGTTCAGTCCCGGATAGCGACGGGCGGTGTGTA | 41584 | 0.10810293133534936 | No Hit |
| GCGCCATCCATTTTCAGGGCTAGTTGATTCGGCAGGTGAGTTGTTACACA | 40637 | 0.10564108360606463 | No Hit |
| CGGGGCTGCACGCGTGCTACACTGGTGGAGTCAGCGGGTTTTTCCTATGC | 39280 | 0.10211338839102835 | No Hit |
| GTCGTATGCAAGTGATTTAGCGCCCCGACATTGAACCAGAGTGATCTGGC | 38910 | 0.1011515260258379 | No Hit |
| AAGCTCTCGTGTTGCCACGAGATAGAGATGCCTCCCGACACCGGGAAGCA | 38648 | 0.1004704234861625 | No Hit |

## Adapter Content

Produced by FastQC (version 0.12.1)
