# Supplementary material for: The pos-1 3′ untranslated region governs germline specification and proliferation to ensure reproductive robustness
Source: PLoS Genet. 2026 Apr 27;22(4):e1012129. doi: 10.1371/journal.pgen.1012129 (PMC13132445; doi:10.1371/journal.pgen.1012129)
Supplement: S1 Data — (GZ) [file pgen.1012129.s007.gz › SupplementalDataSet2/OneStopRNASeq/fastqc.zip!/fastqc/details_raw/WRM101_20_rep2.R1_fastqc.html]

WRM101\_20\_rep2.R1.fastq.gz FastQC Report 

FastQC Report

Wed 12 Feb 2025  
WRM101\_20\_rep2.R1.fastq.gz

## Summary

- Basic Statistics
- Per base sequence quality
- Per tile sequence quality
- Per sequence quality scores
- Per base sequence content
- Per sequence GC content
- Per base N content
- Sequence Length Distribution
- Sequence Duplication Levels
- Overrepresented sequences
- Adapter Content

## Basic Statistics

| Measure | Value |
| --- | --- |
| Filename | WRM101\_20\_rep2.R1.fastq.gz |
| File type | Conventional base calls |
| Encoding | Sanger / Illumina 1.9 |
| Total Sequences | 55274525 |
| Total Bases | 2.7 Gbp |
| Sequences flagged as poor quality | 0 |
| Sequence length | 35-51 |
| %GC | 48 |

## Per base sequence quality

## Per tile sequence quality

## Per sequence quality scores

## Per base sequence content

## Per sequence GC content

## Per base N content

## Sequence Length Distribution

## Sequence Duplication Levels

## Overrepresented sequences

| Sequence | Count | Percentage | Possible Source |
| --- | --- | --- | --- |
| GATCGGAAGAGCACACGTCTGAACTCCAGTCACGCCAATATCTGGGGGGG | 491651 | 0.8894712347143643 | TruSeq Adapter, Index 6 (97% over 45bp) |
| GATCGGAAGAGCACACGTCTGAACTCCAGTCACGCCAATATCTAGGGGGG | 74101 | 0.13405994895478524 | TruSeq Adapter, Index 6 (97% over 45bp) |
| ATCGGAAGAGCACACGTCTGAACTCCAGTCACGCCAATATCTGGGGGGGG | 68966 | 0.12476995505614928 | TruSeq Adapter, Index 6 (97% over 44bp) |
| GTCGCAACGGCGGAGTATAGGTCTCTCGCTTAAGCGCCATCCATTTTCAG | 59642 | 0.10790142475218013 | No Hit |
| CCCCACTCTTCTCGAATCAGTTCAGTCCCGGATAGCGACGGGCGGTGTGT | 57968 | 0.10487290483274167 | No Hit |
| GCGCCATCCATTTTCAGGGCTAGTTGATTCGGCAGGTGAGTTGTTACACA | 57835 | 0.1046322876587361 | No Hit |
| CCGACATTGAACCAGAGTGATCTGGCGTTGGCAAGCGTCGCAGAATTCAC | 57647 | 0.10429216714209666 | No Hit |

## Adapter Content

Produced by FastQC (version 0.12.1)
