# Supplementary material for: The pos-1 3′ untranslated region governs germline specification and proliferation to ensure reproductive robustness
Source: PLoS Genet. 2026 Apr 27;22(4):e1012129. doi: 10.1371/journal.pgen.1012129 (PMC13132445; doi:10.1371/journal.pgen.1012129)
Supplement: S1 Data — (GZ) [file pgen.1012129.s007.gz › SupplementalDataSet2/OneStopRNASeq/fastqc.zip!/fastqc/details_raw/WRM101_20_rep1.R2_fastqc.html]

WRM101\_20\_rep1.R2.fastq.gz FastQC Report 

FastQC Report

Wed 12 Feb 2025  
WRM101\_20\_rep1.R2.fastq.gz

## Summary

- Basic Statistics
- Per base sequence quality
- Per tile sequence quality
- Per sequence quality scores
- Per base sequence content
- Per sequence GC content
- Per base N content
- Sequence Length Distribution
- Sequence Duplication Levels
- Overrepresented sequences
- Adapter Content

## Basic Statistics

| Measure | Value |
| --- | --- |
| Filename | WRM101\_20\_rep1.R2.fastq.gz |
| File type | Conventional base calls |
| Encoding | Sanger / Illumina 1.9 |
| Total Sequences | 48309572 |
| Total Bases | 2.4 Gbp |
| Sequences flagged as poor quality | 0 |
| Sequence length | 35-51 |
| %GC | 49 |

## Per base sequence quality

## Per tile sequence quality

## Per sequence quality scores

## Per base sequence content

## Per sequence GC content

## Per base N content

## Sequence Length Distribution

## Sequence Duplication Levels

## Overrepresented sequences

| Sequence | Count | Percentage | Possible Source |
| --- | --- | --- | --- |
| GGGGGGGGGGGGGGGGGGGGGGGGGGGGGGGGGGGGGGGGGGGGGGGGGG | 329370 | 0.6817903499538353 | No Hit |
| CTCGCCTTGTCAAGGCGAAAATTTCTTGCTTCCCGGTGTCGGGAGGCATC | 143857 | 0.2977815659389406 | No Hit |
| CGAAAATTTCTTGCTTCCCGGTGTCGGGAGGCATCTCTATCTCGTGGCAA | 130761 | 0.2706730666129685 | No Hit |
| CCCCACTCTTCTCGAATCAGTTCAGTCCCGGATAGCGACGGGCGGTGTGT | 112782 | 0.2334568395679432 | No Hit |
| GCGAAAATTTCTTGCTTCCCGGTGTCGGGAGGCATCTCTATCTCGTGGCA | 110893 | 0.22954664139852038 | No Hit |
| GGCGAAAATTTCTTGCTTCCCGGTGTCGGGAGGCATCTCTATCTCGTGGC | 109702 | 0.22708129146745493 | No Hit |
| GGGTATTGTAATTATTGCCCTTAAACGAGGAATGCCTAGTAAGTGTGAGT | 107348 | 0.22220855113351035 | No Hit |
| ATAAGCTCTCGTGTTGCCACGAGATAGAGATGCCTCCCGACACCGGGAAG | 101433 | 0.2099646008041636 | No Hit |
| GTCGCAACGGCGGAGTATAGGTCTCTCGCTTAAGCGCCATCCATTTTCAG | 96398 | 0.19954223564638496 | No Hit |
| CCGACATTGAACCAGAGTGATCTGGCGTTGGCAAGCGTCGCAGAATTCAC | 88545 | 0.18328665797328944 | No Hit |
| GCGGAGTATAGGTCTCTCGCTTAAGCGCCATCCATTTTCAGGGCTAGTTG | 79862 | 0.16531299428610133 | No Hit |
| CTGGCGTTGGCAAGCGTCGCAGAATTCACTACGACGCCAAGGCCATACAT | 76773 | 0.15891881633726748 | No Hit |
| GTGATTTAGCGCCCCGACATTGAACCAGAGTGATCTGGCGTTGGCAAGCG | 74736 | 0.1547022606617173 | No Hit |
| GCTACTACCATTACGATCTGCACTGACGGAAGCTCCAGCCGAGCCTACGC | 74422 | 0.15405228595277143 | No Hit |
| GCGACATGTGCGTTGTCTAGCGCCAGGTCGTAACGAGTAGGAAGGTCGTG | 71234 | 0.1474531796721362 | No Hit |
| GCATAAGCTCTCGTGTTGCCACGAGATAGAGATGCCTCCCGACACCGGGA | 62016 | 0.12837207499996067 | No Hit |
| GAAAATTTCTTGCTTCCCGGTGTCGGGAGGCATCTCTATCTCGTGGCAAC | 60775 | 0.1258032259114198 | No Hit |
| GCGTTGGCAAGCGTCGCAGAATTCACTACGACGCCAAGGCCATACATAGG | 60493 | 0.12521949066325821 | No Hit |
| GCCATACATAGGGCATAAGCTCTCGTGTTGCCACGAGATAGAGATGCCTC | 56273 | 0.11648416177232951 | No Hit |
| ATTTCAATGAGTTGAATACAAATGATTCTTCGAGTAGCAAGGAGAGGGCA | 55561 | 0.11501033376987897 | No Hit |
| GCGCCATCCATTTTCAGGGCTAGTTGATTCGGCAGGTGAGTTGTTACACA | 50942 | 0.10544908160229612 | No Hit |
| CTACCATTACGATCTGCACTGACGGAAGCTCCAGCCGAGCCTACGCTCAT | 48725 | 0.1008599289598343 | No Hit |
| CGGGAGGCATCTCTATCTCGTGGCAACACGAGAGCTTATGCCCTATGTAT | 48701 | 0.10081024936424608 | No Hit |

## Adapter Content

Produced by FastQC (version 0.12.1)
