# Supplementary material for: The pos-1 3′ untranslated region governs germline specification and proliferation to ensure reproductive robustness
Source: PLoS Genet. 2026 Apr 27;22(4):e1012129. doi: 10.1371/journal.pgen.1012129 (PMC13132445; doi:10.1371/journal.pgen.1012129)
Supplement: S1 Data — (GZ) [file pgen.1012129.s007.gz › SupplementalDataSet2/OneStopRNASeq/fastqc.zip!/fastqc/details_raw/DG4222_20_rep3.R1_fastqc.html]

DG4222\_20\_rep3.R1.fastq.gz FastQC Report 

FastQC Report

Wed 12 Feb 2025  
DG4222\_20\_rep3.R1.fastq.gz

## Summary

- Basic Statistics
- Per base sequence quality
- Per tile sequence quality
- Per sequence quality scores
- Per base sequence content
- Per sequence GC content
- Per base N content
- Sequence Length Distribution
- Sequence Duplication Levels
- Overrepresented sequences
- Adapter Content

## Basic Statistics

| Measure | Value |
| --- | --- |
| Filename | DG4222\_20\_rep3.R1.fastq.gz |
| File type | Conventional base calls |
| Encoding | Sanger / Illumina 1.9 |
| Total Sequences | 54534846 |
| Total Bases | 2.7 Gbp |
| Sequences flagged as poor quality | 0 |
| Sequence length | 35-51 |
| %GC | 48 |

## Per base sequence quality

## Per tile sequence quality

## Per sequence quality scores

## Per base sequence content

## Per sequence GC content

## Per base N content

## Sequence Length Distribution

## Sequence Duplication Levels

## Overrepresented sequences

| Sequence | Count | Percentage | Possible Source |
| --- | --- | --- | --- |
| CCCCACTCTTCTCGAATCAGTTCAGTCCCGGATAGCGACGGGCGGTGTGT | 137951 | 0.2529593647335137 | No Hit |
| GTCGCAACGGCGGAGTATAGGTCTCTCGCTTAAGCGCCATCCATTTTCAG | 95025 | 0.17424638918023166 | No Hit |
| GGAACAGCGGGTTCAAACATTTGGTTCATAGACTTGATCGACAGATCAAT | 90891 | 0.1666659148537799 | No Hit |
| CAAACGTGGAGTTAAGGTGCCTAACTTCTCGCTCATGAGACCCCATAAA | 86747 | 0.1590671036276512 | No Hit |
| CAGAAATCAGTGACAGTCAACGTCCGCAAGGACCATCTGTCACCTCTGTT | 74088 | 0.13585442232659828 | No Hit |
| GGGTATTGTAATTATTGCCCTTAAACGAGGAATGCCTAGTAAGTGTGAGT | 72841 | 0.13356781093688244 | No Hit |
| ATAAGCTCTCGTGTTGCCACGAGATAGAGATGCCTCCCGACACCGGGAAG | 70098 | 0.12853799935549465 | No Hit |
| CGAAAATTTCTTGCTTCCCGGTGTCGGGAGGCATCTCTATCTCGTGGCAA | 69679 | 0.12776968325903038 | No Hit |
| CCGACATTGAACCAGAGTGATCTGGCGTTGGCAAGCGTCGCAGAATTCAC | 64768 | 0.1187644318276795 | No Hit |
| CTCGCCTTAGGACACCTACGTTACGATTTGATAGATGTACCGCCCCAGTC | 64386 | 0.11806396226001994 | No Hit |
| CGAAAAGCTTGCTTTTAGTCCCCGCTTGTCGAAAGGGAATAGGGTTAATA | 62590 | 0.11477065507803946 | No Hit |
| GTGATTTAGCGCCCCGACATTGAACCAGAGTGATCTGGCGTTGGCAAGCG | 58517 | 0.1073020358396171 | No Hit |
| GTGGGAGTCTTCGGACGACAGTGAAATACCACCACTTTCATCGACTCTTT | 58342 | 0.10698114009527046 | No Hit |
| GGAAGCTCCAGCCGAGCCTACGCTCATAGCCTTCAACGCAACCGCCACGA | 57462 | 0.10536749292369874 | No Hit |
| GCGCCATCCATTTTCAGGGCTAGTTGATTCGGCAGGTGAGTTGTTACACA | 55552 | 0.101865145085401 | No Hit |

## Adapter Content

Produced by FastQC (version 0.12.1)
