# Supplementary material for: The pos-1 3′ untranslated region governs germline specification and proliferation to ensure reproductive robustness
Source: PLoS Genet. 2026 Apr 27;22(4):e1012129. doi: 10.1371/journal.pgen.1012129 (PMC13132445; doi:10.1371/journal.pgen.1012129)
Supplement: S1 Data — (GZ) [file pgen.1012129.s007.gz › SupplementalDataSet2/OneStopRNASeq/fastqc.zip!/fastqc/details_raw/DG4222_20_rep3.R2_fastqc.html]

DG4222\_20\_rep3.R2.fastq.gz FastQC Report 

FastQC Report

Wed 12 Feb 2025  
DG4222\_20\_rep3.R2.fastq.gz

## Summary

- Basic Statistics
- Per base sequence quality
- Per tile sequence quality
- Per sequence quality scores
- Per base sequence content
- Per sequence GC content
- Per base N content
- Sequence Length Distribution
- Sequence Duplication Levels
- Overrepresented sequences
- Adapter Content

## Basic Statistics

| Measure | Value |
| --- | --- |
| Filename | DG4222\_20\_rep3.R2.fastq.gz |
| File type | Conventional base calls |
| Encoding | Sanger / Illumina 1.9 |
| Total Sequences | 54534846 |
| Total Bases | 2.7 Gbp |
| Sequences flagged as poor quality | 0 |
| Sequence length | 35-51 |
| %GC | 48 |

## Per base sequence quality

## Per tile sequence quality

## Per sequence quality scores

## Per base sequence content

## Per sequence GC content

## Per base N content

## Sequence Length Distribution

## Sequence Duplication Levels

## Overrepresented sequences

| Sequence | Count | Percentage | Possible Source |
| --- | --- | --- | --- |
| CCCCACTCTTCTCGAATCAGTTCAGTCCCGGATAGCGACGGGCGGTGTGT | 141232 | 0.2589757015175215 | No Hit |
| GGGGGGGGGGGGGGGGGGGGGGGGGGGGGGGGGGGGGGGGGGGGGGGGGG | 111052 | 0.20363493829248183 | No Hit |
| GTCGCAACGGCGGAGTATAGGTCTCTCGCTTAAGCGCCATCCATTTTCAG | 108363 | 0.19870414596934957 | No Hit |
| GGAACAGCGGGTTCAAACATTTGGTTCATAGACTTGATCGACAGATCAAT | 93567 | 0.17157286920733214 | No Hit |
| CAAACGTGGAGTTAAGGTGCCTAACTTCTCGCTCATGAGACCCCATAAA | 90732 | 0.16637435814891638 | No Hit |
| CAGAAATCAGTGACAGTCAACGTCCGCAAGGACCATCTGTCACCTCTGTT | 77457 | 0.14203212382776326 | No Hit |
| ATAAGCTCTCGTGTTGCCACGAGATAGAGATGCCTCCCGACACCGGGAAG | 74838 | 0.13722968980236966 | No Hit |
| GGGTATTGTAATTATTGCCCTTAAACGAGGAATGCCTAGTAAGTGTGAGT | 74612 | 0.13681527586967057 | No Hit |
| CGAAAATTTCTTGCTTCCCGGTGTCGGGAGGCATCTCTATCTCGTGGCAA | 73152 | 0.13413808851683565 | No Hit |
| CCGACATTGAACCAGAGTGATCTGGCGTTGGCAAGCGTCGCAGAATTCAC | 66139 | 0.12127842077338956 | No Hit |
| CTCGCCTTAGGACACCTACGTTACGATTTGATAGATGTACCGCCCCAGTC | 65534 | 0.12016903834293398 | No Hit |
| CGAAAAGCTTGCTTTTAGTCCCCGCTTGTCGAAAGGGAATAGGGTTAATA | 64815 | 0.11885061525616118 | No Hit |
| GGAAGCTCCAGCCGAGCCTACGCTCATAGCCTTCAACGCAACCGCCACGA | 62360 | 0.11434890638546957 | No Hit |
| GTGGGAGTCTTCGGACGACAGTGAAATACCACCACTTTCATCGACTCTTT | 59476 | 0.10906054451863677 | No Hit |
| GTGATTTAGCGCCCCGACATTGAACCAGAGTGATCTGGCGTTGGCAAGCG | 58616 | 0.10748357114641893 | No Hit |
| AAGCTCTCGTGTTGCCACGAGATAGAGATGCCTCCCGACACCGGGAAGCA | 55933 | 0.10256378096309285 | No Hit |
| GCCATTTTTGGTAAGCAGAACTGGCGCTGTGGGATGAACCAAACGTGGAG | 55208 | 0.10123435573651386 | No Hit |
| GCGGGTTCAAACATTTGGTTCATAGACTTGATCGACAGATCAATGGTCTG | 54904 | 0.10067691398633453 | No Hit |

## Adapter Content

Produced by FastQC (version 0.12.1)
