# Supplementary material for: The pos-1 3′ untranslated region governs germline specification and proliferation to ensure reproductive robustness
Source: PLoS Genet. 2026 Apr 27;22(4):e1012129. doi: 10.1371/journal.pgen.1012129 (PMC13132445; doi:10.1371/journal.pgen.1012129)
Supplement: S1 Data — (GZ) [file pgen.1012129.s007.gz › SupplementalDataSet2/OneStopRNASeq/fastqc.zip!/fastqc/details_raw/WRM101_20_rep3.R1_fastqc.html]

WRM101\_20\_rep3.R1.fastq.gz FastQC Report 

FastQC Report

Wed 12 Feb 2025  
WRM101\_20\_rep3.R1.fastq.gz

## Summary

- Basic Statistics
- Per base sequence quality
- Per tile sequence quality
- Per sequence quality scores
- Per base sequence content
- Per sequence GC content
- Per base N content
- Sequence Length Distribution
- Sequence Duplication Levels
- Overrepresented sequences
- Adapter Content

## Basic Statistics

| Measure | Value |
| --- | --- |
| Filename | WRM101\_20\_rep3.R1.fastq.gz |
| File type | Conventional base calls |
| Encoding | Sanger / Illumina 1.9 |
| Total Sequences | 78810492 |
| Total Bases | 3.9 Gbp |
| Sequences flagged as poor quality | 0 |
| Sequence length | 35-51 |
| %GC | 49 |

## Per base sequence quality

## Per tile sequence quality

## Per sequence quality scores

## Per base sequence content

## Per sequence GC content

## Per base N content

## Sequence Length Distribution

## Sequence Duplication Levels

## Overrepresented sequences

| Sequence | Count | Percentage | Possible Source |
| --- | --- | --- | --- |
| CCCCACTCTTCTCGAATCAGTTCAGTCCCGGATAGCGACGGGCGGTGTGT | 383080 | 0.4860774121293393 | No Hit |
| GGGTATTGTAATTATTGCCCTTAAACGAGGAATGCCTAGTAAGTGTGAGT | 306392 | 0.38877057130921094 | No Hit |
| CGAAAATTTCTTGCTTCCCGGTGTCGGGAGGCATCTCTATCTCGTGGCAA | 280708 | 0.35618100188995144 | No Hit |
| CTCGCCTTGTCAAGGCGAAAATTTCTTGCTTCCCGGTGTCGGGAGGCATC | 254148 | 0.3224799053405224 | No Hit |
| GCGACATGTGCGTTGTCTAGCGCCAGGTCGTAACGAGTAGGAAGGTCGTG | 247269 | 0.3137513720888838 | No Hit |
| GCTACTACCATTACGATCTGCACTGACGGAAGCTCCAGCCGAGCCTACGC | 230188 | 0.29207786191716706 | No Hit |
| CCGACATTGAACCAGAGTGATCTGGCGTTGGCAAGCGTCGCAGAATTCAC | 228732 | 0.2902303921665658 | No Hit |
| GATCGGAAGAGCACACGTCTGAACTCCAGTCACACTTGAATCTGGGGGGG | 227220 | 0.28831186588709534 | TruSeq Adapter, Index 8 (97% over 45bp) |
| GTGATTTAGCGCCCCGACATTGAACCAGAGTGATCTGGCGTTGGCAAGCG | 214103 | 0.2716681428660539 | No Hit |
| GCGAAAATTTCTTGCTTCCCGGTGTCGGGAGGCATCTCTATCTCGTGGCA | 204058 | 0.2589223780001272 | No Hit |
| ATAAGCTCTCGTGTTGCCACGAGATAGAGATGCCTCCCGACACCGGGAAG | 200720 | 0.2546869013328834 | No Hit |
| GTCGCAACGGCGGAGTATAGGTCTCTCGCTTAAGCGCCATCCATTTTCAG | 187549 | 0.23797465951614663 | No Hit |
| ATTTCAATGAGTTGAATACAAATGATTCTTCGAGTAGCAAGGAGAGGGCA | 180299 | 0.22877537676074905 | No Hit |
| GGAACAGCGGGTTCAAACATTTGGTTCATAGACTTGATCGACAGATCAAT | 174396 | 0.221285257297975 | No Hit |
| CTACCATTACGATCTGCACTGACGGAAGCTCCAGCCGAGCCTACGCTCAT | 172296 | 0.21862063746537708 | No Hit |
| GGCGAAAATTTCTTGCTTCCCGGTGTCGGGAGGCATCTCTATCTCGTGGC | 166004 | 0.2106369289002789 | No Hit |
| GCGTTGTCTAGCGCCAGGTCGTAACGAGTAGGAAGGTCGTGGCGGTTGCG | 154875 | 0.19651571265409687 | No Hit |
| GCGGAGTATAGGTCTCTCGCTTAAGCGCCATCCATTTTCAGGGCTAGTTG | 148183 | 0.18802445745421814 | No Hit |
| CTATACTCCGCCGTTGCGACATGTGCGTTGTCTAGCGCCAGGTCGTAACG | 146096 | 0.18537633288725058 | No Hit |
| CCCACTCTTCTCGAATCAGTTCAGTCCCGGATAGCGACGGGCGGTGTGTA | 144311 | 0.18311140602954237 | No Hit |
| GTTTAGATGAGCGATCAGGATACTAGAACCGAGGTCCAAGCACACACC | 143464 | 0.18203667603039453 | No Hit |
| CGAAAGTCAGAGGTTCGAAGGCGATTAGATACCGCCCTAGTTCTGACCGT | 142727 | 0.18110152135581134 | No Hit |
| CTGGCGTTGGCAAGCGTCGCAGAATTCACTACGACGCCAAGGCCATACAT | 140460 | 0.17822500080319256 | No Hit |
| GAAAATTTCTTGCTTCCCGGTGTCGGGAGGCATCTCTATCTCGTGGCAAC | 137538 | 0.17451737263612058 | No Hit |
| GTTATTTCAATGAGTTGAATACAAATGATTCTTCGAGTAGCAAGGAGAGG | 136003 | 0.1725696624251502 | No Hit |
| GCAATAACGAGATACACTAGGAGAGCTGGAATTACCGCGGCTGCTGGCAC | 128807 | 0.16343889846544798 | No Hit |
| CCGGATAGCTCCTCGGCAGGGCAAAAACCCTCCGAATCGCGAGATGGCAT | 126043 | 0.15993175121911432 | No Hit |
| GCGCCATCCATTTTCAGGGCTAGTTGATTCGGCAGGTGAGTTGTTACACA | 125894 | 0.15974269009765857 | No Hit |
| GGAATGCCTAGTAAGTGTGAGTCATCAGCTCACGTTGATTACGTCCCTGC | 118262 | 0.15005870030604554 | No Hit |
| GTGGGAGTCTTCGGACGACAGTGAAATACCACCACTTTCATCGACTCTTT | 115413 | 0.14644369939982102 | No Hit |
| CTCCGCTTAATGCTTCGAAAAAAGGCCGTGAAGCCAATTCTCTTTTAACC | 113799 | 0.1443957487284815 | No Hit |
| GCATAAGCTCTCGTGTTGCCACGAGATAGAGATGCCTCCCGACACCGGGA | 111913 | 0.14200266634549114 | No Hit |
| GTAATTATTGCCCTTAAACGAGGAATGCCTAGTAAGTGTGAGTCATCAGC | 108908 | 0.13818972225170223 | No Hit |
| CGGATAGCTCCTCGGCAGGGCAAAAACCCTCCGAATCGCGAGATGGCATC | 106324 | 0.1349109709910198 | No Hit |
| TTCCGGATAGCTCCTCGGCAGGGCAAAAACCCTCCGAATCGCGAGATGGC | 104676 | 0.13281987885572394 | No Hit |
| GTCGTATGCAAGTGATTTAGCGCCCCGACATTGAACCAGAGTGATCTGGC | 103717 | 0.13160303579883753 | No Hit |
| CCGCTTAATGCTTCGAAAAAAGGCCGTGAAGCCAATTCTCTTTTAACCGA | 102305 | 0.12981139617806217 | No Hit |
| GCGTTGGCAAGCGTCGCAGAATTCACTACGACGCCAAGGCCATACATAGG | 102143 | 0.12960583979097606 | No Hit |
| CGGGAGGCATCTCTATCTCGTGGCAACACGAGAGCTTATGCCCTATGTAT | 101755 | 0.12911351955523892 | No Hit |
| GCGGGTTCAAACATTTGGTTCATAGACTTGATCGACAGATCAATGGTCTG | 100713 | 0.12779136057163556 | No Hit |
| TGCTACTACCATTACGATCTGCACTGACGGAAGCTCCAGCCGAGCCTACG | 99398 | 0.1261228010097945 | No Hit |
| CCCGACATTGAACCAGAGTGATCTGGCGTTGGCAAGCGTCGCAGAATTCA | 98456 | 0.12492752868488628 | No Hit |
| GCCATACATAGGGCATAAGCTCTCGTGTTGCCACGAGATAGAGATGCCTC | 98434 | 0.12489961361997334 | No Hit |
| GCGGACTAGTGATTGTGCTTGCTTGCGGACGCTTTCTGGTGTGTGCTTGG | 93596 | 0.11876083707230252 | No Hit |
| AAGCTCTCGTGTTGCCACGAGATAGAGATGCCTCCCGACACCGGGAAGCA | 93506 | 0.1186466390794769 | No Hit |
| AAAATTTCTTGCTTCCCGGTGTCGGGAGGCATCTCTATCTCGTGGCAACA | 92481 | 0.1173460508278517 | No Hit |
| CAAACGTGGAGTTAAGGTGCCTAACTTCTCGCTCATGAGACCCCATAAA | 91741 | 0.11640708955350768 | No Hit |
| CGCAGCAATAACGAGATACACTAGGAGAGCTGGAATTACCGCGGCTGCTG | 88446 | 0.11222617414950284 | No Hit |
| TGAGACCCCATAAAAGGTGTTGGTTGATATTGACAGCAGGACGGTGGCCA | 84811 | 0.10761384410593453 | No Hit |
| CTCGAAGCGACAGTCCCCACTCTTCTCGAATCAGTTCAGTCCCGGATAGC | 84803 | 0.10760369317323892 | No Hit |
| CCACTCTTCTCGAATCAGTTCAGTCCCGGATAGCGACGGGCGGTGTGTAC | 84652 | 0.10741209431860926 | No Hit |
| TCCGGATAGCTCCTCGGCAGGGCAAAAACCCTCCGAATCGCGAGATGGCA | 84050 | 0.1066482366332645 | No Hit |
| CCCGGACATGGAAGAATTTCAACGGTTTACCGATACCTTTCGGCATAGGA | 83775 | 0.10629929832185288 | No Hit |
| CGAAAAGCTTGCTTTTAGTCCCCGCTTGTCGAAAGGGAATAGGGTTAATA | 83464 | 0.105904680813311 | No Hit |
| GGGAGGCATCTCTATCTCGTGGCAACACGAGAGCTTATGCCCTATGTATG | 83201 | 0.10557096890094278 | No Hit |
| GGGACAGTAGGAATCTCGTTAATCCATTCATGCGCGTCAACAATTAAATG | 80234 | 0.10180624173745802 | No Hit |
| GGCATAAGCTCTCGTGTTGCCACGAGATAGAGATGCCTCCCGACACCGGG | 80074 | 0.10160322308354577 | No Hit |
| CCGGGATAGGGTATTGTAATTATTGCCCTTAAACGAGGAATGCCTAGTA | 79871 | 0.10134564316639463 | No Hit |
| CAAGAACGAAAGTCAGAGGTTCGAAGGCGATTAGATACCGCCCTAGTTCT | 79378 | 0.10072009193902762 | No Hit |

## Adapter Content

Produced by FastQC (version 0.12.1)
