# Supplementary material for: The pos-1 3′ untranslated region governs germline specification and proliferation to ensure reproductive robustness
Source: PLoS Genet. 2026 Apr 27;22(4):e1012129. doi: 10.1371/journal.pgen.1012129 (PMC13132445; doi:10.1371/journal.pgen.1012129)
Supplement: S1 Data — (GZ) [file pgen.1012129.s007.gz › SupplementalDataSet2/OneStopRNASeq/fastqc.zip!/fastqc/details_raw/WRM101_20_rep3.R2_fastqc.html]

WRM101\_20\_rep3.R2.fastq.gz FastQC Report 

FastQC Report

Wed 12 Feb 2025  
WRM101\_20\_rep3.R2.fastq.gz

## Summary

- Basic Statistics
- Per base sequence quality
- Per tile sequence quality
- Per sequence quality scores
- Per base sequence content
- Per sequence GC content
- Per base N content
- Sequence Length Distribution
- Sequence Duplication Levels
- Overrepresented sequences
- Adapter Content

## Basic Statistics

| Measure | Value |
| --- | --- |
| Filename | WRM101\_20\_rep3.R2.fastq.gz |
| File type | Conventional base calls |
| Encoding | Sanger / Illumina 1.9 |
| Total Sequences | 78810492 |
| Total Bases | 3.9 Gbp |
| Sequences flagged as poor quality | 0 |
| Sequence length | 35-51 |
| %GC | 50 |

## Per base sequence quality

## Per tile sequence quality

## Per sequence quality scores

## Per base sequence content

## Per sequence GC content

## Per base N content

## Sequence Length Distribution

## Sequence Duplication Levels

## Overrepresented sequences

| Sequence | Count | Percentage | Possible Source |
| --- | --- | --- | --- |
| GGGGGGGGGGGGGGGGGGGGGGGGGGGGGGGGGGGGGGGGGGGGGGGGGG | 516177 | 0.6549597482528088 | No Hit |
| CCCCACTCTTCTCGAATCAGTTCAGTCCCGGATAGCGACGGGCGGTGTGT | 416243 | 0.5281568347524084 | No Hit |
| GGGTATTGTAATTATTGCCCTTAAACGAGGAATGCCTAGTAAGTGTGAGT | 310836 | 0.394409414421623 | No Hit |
| CGAAAATTTCTTGCTTCCCGGTGTCGGGAGGCATCTCTATCTCGTGGCAA | 283117 | 0.35923770149791734 | No Hit |
| GCGACATGTGCGTTGTCTAGCGCCAGGTCGTAACGAGTAGGAAGGTCGTG | 258976 | 0.32860599322232376 | No Hit |
| GTCGCAACGGCGGAGTATAGGTCTCTCGCTTAAGCGCCATCCATTTTCAG | 251734 | 0.3194168613996218 | No Hit |
| CTCGCCTTGTCAAGGCGAAAATTTCTTGCTTCCCGGTGTCGGGAGGCATC | 249695 | 0.3168296424288279 | No Hit |
| GCTACTACCATTACGATCTGCACTGACGGAAGCTCCAGCCGAGCCTACGC | 240077 | 0.3046256835955294 | No Hit |
| CCGACATTGAACCAGAGTGATCTGGCGTTGGCAAGCGTCGCAGAATTCAC | 236195 | 0.2996999435049841 | No Hit |
| GTGATTTAGCGCCCCGACATTGAACCAGAGTGATCTGGCGTTGGCAAGCG | 219682 | 0.27874714955465574 | No Hit |
| ATAAGCTCTCGTGTTGCCACGAGATAGAGATGCCTCCCGACACCGGGAAG | 214310 | 0.27193079824955285 | No Hit |
| GCGAAAATTTCTTGCTTCCCGGTGTCGGGAGGCATCTCTATCTCGTGGCA | 205732 | 0.2610464606666838 | No Hit |
| ATTTCAATGAGTTGAATACAAATGATTCTTCGAGTAGCAAGGAGAGGGCA | 177391 | 0.22508551272589442 | No Hit |
| CTACCATTACGATCTGCACTGACGGAAGCTCCAGCCGAGCCTACGCTCAT | 177329 | 0.22500684299750343 | No Hit |
| GGAACAGCGGGTTCAAACATTTGGTTCATAGACTTGATCGACAGATCAAT | 174622 | 0.22157202114662602 | No Hit |
| GGCGAAAATTTCTTGCTTCCCGGTGTCGGGAGGCATCTCTATCTCGTGGC | 169437 | 0.21499294789328305 | No Hit |
| GCGGAGTATAGGTCTCTCGCTTAAGCGCCATCCATTTTCAGGGCTAGTTG | 169046 | 0.21449682105778503 | No Hit |
| GCGTTGTCTAGCGCCAGGTCGTAACGAGTAGGAAGGTCGTGGCGGTTGCG | 157309 | 0.19960413392673657 | No Hit |
| CTATACTCCGCCGTTGCGACATGTGCGTTGTCTAGCGCCAGGTCGTAACG | 147894 | 0.1876577550105892 | No Hit |
| GTTTAGATGAGCGATCAGGATACTAGAACCGAGGTCCAAGCACACACC | 145156 | 0.18418359829551628 | No Hit |
| GAAAATTTCTTGCTTCCCGGTGTCGGGAGGCATCTCTATCTCGTGGCAAC | 143858 | 0.1825366094656534 | No Hit |
| CTGGCGTTGGCAAGCGTCGCAGAATTCACTACGACGCCAAGGCCATACAT | 142129 | 0.18034273913681442 | No Hit |
| CCCACTCTTCTCGAATCAGTTCAGTCCCGGATAGCGACGGGCGGTGTGTA | 140842 | 0.17870970783940798 | No Hit |
| TTCCGGATAGCTCCTCGGCAGGGCAAAAACCCTCCGAATCGCGAGATGGC | 139981 | 0.17761721370804284 | No Hit |
| GCAATAACGAGATACACTAGGAGAGCTGGAATTACCGCGGCTGCTGGCAC | 139828 | 0.17742307712023928 | No Hit |
| CGAAAGTCAGAGGTTCGAAGGCGATTAGATACCGCCCTAGTTCTGACCGT | 135563 | 0.17201136112689158 | No Hit |
| CCGGATAGCTCCTCGGCAGGGCAAAAACCCTCCGAATCGCGAGATGGCAT | 132587 | 0.16823521416412424 | No Hit |
| GCGCCATCCATTTTCAGGGCTAGTTGATTCGGCAGGTGAGTTGTTACACA | 130963 | 0.16617457482691517 | No Hit |
| GTTATTTCAATGAGTTGAATACAAATGATTCTTCGAGTAGCAAGGAGAGG | 125657 | 0.15944196871655109 | No Hit |
| CTCCGCTTAATGCTTCGAAAAAAGGCCGTGAAGCCAATTCTCTTTTAACC | 122832 | 0.1558574206084134 | No Hit |
| GGAATGCCTAGTAAGTGTGAGTCATCAGCTCACGTTGATTACGTCCCTGC | 119876 | 0.1521066509773851 | No Hit |
| GCATAAGCTCTCGTGTTGCCACGAGATAGAGATGCCTCCCGACACCGGGA | 117689 | 0.1493316397517224 | No Hit |
| GTGGGAGTCTTCGGACGACAGTGAAATACCACCACTTTCATCGACTCTTT | 116840 | 0.14825437201940067 | No Hit |
| GTCGTATGCAAGTGATTTAGCGCCCCGACATTGAACCAGAGTGATCTGGC | 114629 | 0.14544890799565113 | No Hit |
| CGGATAGCTCCTCGGCAGGGCAAAAACCCTCCGAATCGCGAGATGGCATC | 109992 | 0.13956517363195753 | No Hit |
| GTAATTATTGCCCTTAAACGAGGAATGCCTAGTAAGTGTGAGTCATCAGC | 109462 | 0.1388926743408733 | No Hit |
| TCCGGATAGCTCCTCGGCAGGGCAAAAACCCTCCGAATCGCGAGATGGCA | 107283 | 0.1361278140479062 | No Hit |
| TGCTACTACCATTACGATCTGCACTGACGGAAGCTCCAGCCGAGCCTACG | 105918 | 0.13439581115671756 | No Hit |
| CCGCTTAATGCTTCGAAAAAAGGCCGTGAAGCCAATTCTCTTTTAACCGA | 105233 | 0.13352663754465585 | No Hit |
| GCGTTGGCAAGCGTCGCAGAATTCACTACGACGCCAAGGCCATACATAGG | 105154 | 0.13342639708428672 | No Hit |
| GCCATACATAGGGCATAAGCTCTCGTGTTGCCACGAGATAGAGATGCCTC | 104194 | 0.13220828516081337 | No Hit |
| CGGGAGGCATCTCTATCTCGTGGCAACACGAGAGCTTATGCCCTATGTAT | 103984 | 0.13194182317755357 | No Hit |
| AAGCTCTCGTGTTGCCACGAGATAGAGATGCCTCCCGACACCGGGAAGCA | 102781 | 0.13041537667345104 | No Hit |
| GCGGGTTCAAACATTTGGTTCATAGACTTGATCGACAGATCAATGGTCTG | 101586 | 0.12889908110204412 | No Hit |
| CCCGACATTGAACCAGAGTGATCTGGCGTTGGCAAGCGTCGCAGAATTCA | 100862 | 0.12798042169309132 | No Hit |
| AAAATTTCTTGCTTCCCGGTGTCGGGAGGCATCTCTATCTCGTGGCAACA | 98051 | 0.12441363771717096 | No Hit |
| GCGGACTAGTGATTGTGCTTGCTTGCGGACGCTTTCTGGTGTGTGCTTGG | 96214 | 0.12208272979694125 | No Hit |
| CGCAGCAATAACGAGATACACTAGGAGAGCTGGAATTACCGCGGCTGCTG | 95526 | 0.12120974958511871 | No Hit |
| CAAACGTGGAGTTAAGGTGCCTAACTTCTCGCTCATGAGACCCCATAAA | 91794 | 0.1164743394826161 | No Hit |
| GGGAGGCATCTCTATCTCGTGGCAACACGAGAGCTTATGCCCTATGTATG | 87291 | 0.11076063324157398 | No Hit |
| CTCGAAGCGACAGTCCCCACTCTTCTCGAATCAGTTCAGTCCCGGATAGC | 86844 | 0.1101934498772067 | No Hit |
| CCACTCTTCTCGAATCAGTTCAGTCCCGGATAGCGACGGGCGGTGTGTAC | 86740 | 0.11006148775216376 | No Hit |
| CGGCGGAGTATAGGTCTCTCGCTTAAGCGCCATCCATTTTCAGGGCTAGT | 85707 | 0.10875074856784298 | No Hit |
| CCCGGACATGGAAGAATTTCAACGGTTTACCGATACCTTTCGGCATAGGA | 83717 | 0.1062257040598097 | No Hit |
| CCGGGATAGGGTATTGTAATTATTGCCCTTAAACGAGGAATGCCTAGTA | 83507 | 0.10595924207654991 | No Hit |
| GGGACAGTAGGAATCTCGTTAATCCATTCATGCGCGTCAACAATTAAATG | 82888 | 0.105173813659227 | No Hit |
| GGCATAAGCTCTCGTGTTGCCACGAGATAGAGATGCCTCCCGACACCGGG | 82126 | 0.10420693731997004 | No Hit |
| CTGGAATTACCGCGGCTGCTGGCACCAGACTTGCCCTCTCCTTGCTACTC | 81759 | 0.10374126328255888 | No Hit |
| CGAAAAGCTTGCTTTTAGTCCCCGCTTGTCGAAAGGGAATAGGGTTAATA | 81363 | 0.10323879211412612 | No Hit |
| GGAAGCTCCAGCCGAGCCTACGCTCATAGCCTTCAACGCAACCGCCACGA | 81122 | 0.10293299526667084 | No Hit |
| TGAGACCCCATAAAAGGTGTTGGTTGATATTGACAGCAGGACGGTGGCCA | 80523 | 0.10217294418108694 | No Hit |
| AAATTTCTTGCTTCCCGGTGTCGGGAGGCATCTCTATCTCGTGGCAACAC | 80495 | 0.1021374159166523 | No Hit |

## Adapter Content

Produced by FastQC (version 0.12.1)
