# Supplementary material for: The pos-1 3′ untranslated region governs germline specification and proliferation to ensure reproductive robustness
Source: PLoS Genet. 2026 Apr 27;22(4):e1012129. doi: 10.1371/journal.pgen.1012129 (PMC13132445; doi:10.1371/journal.pgen.1012129)
Supplement: S1 Data — (GZ) [file pgen.1012129.s007.gz › SupplementalDataSet2/OneStopRNASeq/fastqc.zip!/fastqc/details_trimmed/DG4222_20_rep1.R2_fastqc.html]

DG4222\_20\_rep1.R2.fastq.gz FastQC Report 

FastQC Report

Wed 12 Feb 2025  
DG4222\_20\_rep1.R2.fastq.gz

## Summary

- Basic Statistics
- Per base sequence quality
- Per tile sequence quality
- Per sequence quality scores
- Per base sequence content
- Per sequence GC content
- Per base N content
- Sequence Length Distribution
- Sequence Duplication Levels
- Overrepresented sequences
- Adapter Content

## Basic Statistics

| Measure | Value |
| --- | --- |
| Filename | DG4222\_20\_rep1.R2.fastq.gz |
| File type | Conventional base calls |
| Encoding | Sanger / Illumina 1.9 |
| Total Sequences | 37186120 |
| Total Bases | 1.8 Gbp |
| Sequences flagged as poor quality | 0 |
| Sequence length | 15-51 |
| %GC | 48 |

## Per base sequence quality

## Per tile sequence quality

## Per sequence quality scores

## Per base sequence content

## Per sequence GC content

## Per base N content

## Sequence Length Distribution

## Sequence Duplication Levels

## Overrepresented sequences

| Sequence | Count | Percentage | Possible Source |
| --- | --- | --- | --- |
| CCCCACTCTTCTCGAATCAGTTCAGTCCCGGATAGCGACGGGCGGTGTGT | 108634 | 0.2921358829584802 | No Hit |
| GTCGCAACGGCGGAGTATAGGTCTCTCGCTTAAGCGCCATCCATTTTCAG | 100154 | 0.26933167536704555 | No Hit |
| GGGGGGGGGGGGGGGGGGGGGGGGGGGGGGGGGGGGGGGGGGGGGGGGGG | 90694 | 0.24389207586056305 | No Hit |
| CCGACATTGAACCAGAGTGATCTGGCGTTGGCAAGCGTCGCAGAATTCAC | 82659 | 0.22228455133259398 | No Hit |
| GGGTATTGTAATTATTGCCCTTAAACGAGGAATGCCTAGTAAGTGTGAGT | 82416 | 0.22163108170467904 | No Hit |
| CGAAAATTTCTTGCTTCCCGGTGTCGGGAGGCATCTCTATCTCGTGGCAA | 75904 | 0.2041191713467283 | No Hit |
| CAAACGTGGAGTTAAGGTGCCTAACTTCTCGCTCATGAGACCCCATAAA | 67798 | 0.18232071536368946 | No Hit |
| GGAACAGCGGGTTCAAACATTTGGTTCATAGACTTGATCGACAGATCAAT | 62085 | 0.16695745616912977 | No Hit |
| GTGATTTAGCGCCCCGACATTGAACCAGAGTGATCTGGCGTTGGCAAGCG | 54690 | 0.1470710039122124 | No Hit |
| GAAAATTTCTTGCTTCCCGGTGTCGGGAGGCATCTCTATCTCGTGGCAAC | 49174 | 0.13223751227608582 | No Hit |
| GGAAGCTCCAGCCGAGCCTACGCTCATAGCCTTCAACGCAACCGCCACGA | 43951 | 0.11819194903905006 | No Hit |
| GCGGAGTATAGGTCTCTCGCTTAAGCGCCATCCATTTTCAGGGCTAGTTG | 42186 | 0.11344555441654036 | No Hit |
| CCCACTCTTCTCGAATCAGTTCAGTCCCGGATAGCGACGGGCGGTGTGTA | 41556 | 0.11175137389972387 | No Hit |
| GCGCCATCCATTTTCAGGGCTAGTTGATTCGGCAGGTGAGTTGTTACACA | 40598 | 0.1091751438439934 | No Hit |
| ATAAGCTCTCGTGTTGCCACGAGATAGAGATGCCTCCCGACACCGGGAAG | 40080 | 0.10778215097461095 | No Hit |
| CGGGGCTGCACGCGTGCTACACTGGTGGAGTCAGCGGGTTTTTCCTATGC | 39255 | 0.10556358125020841 | No Hit |
| CGGCGGAGTATAGGTCTCTCGCTTAAGCGCCATCCATTTTCAGGGCTAGT | 38212 | 0.10275877128347889 | No Hit |
| CCGGGATAGGGTATTGTAATTATTGCCCTTAAACGAGGAATGCCTAGTA | 37761 | 0.10154595316747217 | No Hit |
| AAAATTTCTTGCTTCCCGGTGTCGGGAGGCATCTCTATCTCGTGGCAACA | 37742 | 0.10149485883442531 | No Hit |
| GCCATTTTTGGTAAGCAGAACTGGCGCTGTGGGATGAACCAAACGTGGAG | 37241 | 0.10014758194724269 | No Hit |

## Adapter Content

Produced by FastQC (version 0.12.1)
