# Supplementary material for: The pos-1 3′ untranslated region governs germline specification and proliferation to ensure reproductive robustness
Source: PLoS Genet. 2026 Apr 27;22(4):e1012129. doi: 10.1371/journal.pgen.1012129 (PMC13132445; doi:10.1371/journal.pgen.1012129)
Supplement: S1 Data — (GZ) [file pgen.1012129.s007.gz › SupplementalDataSet2/OneStopRNASeq/fastqc.zip!/fastqc/details_trimmed/WRM101_20_rep1.R1_fastqc.html]

WRM101\_20\_rep1.R1.fastq.gz FastQC Report 

FastQC Report

Wed 12 Feb 2025  
WRM101\_20\_rep1.R1.fastq.gz

## Summary

- Basic Statistics
- Per base sequence quality
- Per tile sequence quality
- Per sequence quality scores
- Per base sequence content
- Per sequence GC content
- Per base N content
- Sequence Length Distribution
- Sequence Duplication Levels
- Overrepresented sequences
- Adapter Content

## Basic Statistics

| Measure | Value |
| --- | --- |
| Filename | WRM101\_20\_rep1.R1.fastq.gz |
| File type | Conventional base calls |
| Encoding | Sanger / Illumina 1.9 |
| Total Sequences | 47018922 |
| Total Bases | 2.3 Gbp |
| Sequences flagged as poor quality | 0 |
| Sequence length | 15-51 |
| %GC | 49 |

## Per base sequence quality

## Per tile sequence quality

## Per sequence quality scores

## Per base sequence content

## Per sequence GC content

## Per base N content

## Sequence Length Distribution

## Sequence Duplication Levels

## Overrepresented sequences

| Sequence | Count | Percentage | Possible Source |
| --- | --- | --- | --- |
| CGAAAATTTCTTGCTTCCCGGTGTCGGGAGGCATCTCTATCTCGTGGCAA | 129827 | 0.2761164962480424 | No Hit |
| GCGAAAATTTCTTGCTTCCCGGTGTCGGGAGGCATCTCTATCTCGTGGCA | 108578 | 0.23092405223582116 | No Hit |
| GGCGAAAATTTCTTGCTTCCCGGTGTCGGGAGGCATCTCTATCTCGTGGC | 107800 | 0.22926939924313877 | No Hit |
| GGGTATTGTAATTATTGCCCTTAAACGAGGAATGCCTAGTAAGTGTGAGT | 105362 | 0.22408425271851193 | No Hit |
| CCCCACTCTTCTCGAATCAGTTCAGTCCCGGATAGCGACGGGCGGTGTGT | 101515 | 0.21590244029839734 | No Hit |
| CCGACATTGAACCAGAGTGATCTGGCGTTGGCAAGCGTCGCAGAATTCAC | 78952 | 0.16791537670727544 | No Hit |
| GCTACTACCATTACGATCTGCACTGACGGAAGCTCCAGCCGAGCCTACGC | 70748 | 0.15046708216747293 | No Hit |
| GCGGAGTATAGGTCTCTCGCTTAAGCGCCATCCATTTTCAGGGCTAGTTG | 69081 | 0.146921701012201 | No Hit |
| GCGACATGTGCGTTGTCTAGCGCCAGGTCGTAACGAGTAGGAAGGTCGTG | 68258 | 0.14517134186955627 | No Hit |
| GTGATTTAGCGCCCCGACATTGAACCAGAGTGATCTGGCGTTGGCAAGCG | 66301 | 0.14100918774786034 | No Hit |
| GTCGCAACGGCGGAGTATAGGTCTCTCGCTTAAGCGCCATCCATTTTCAG | 66286 | 0.14097728569787288 | No Hit |
| ATAAGCTCTCGTGTTGCCACGAGATAGAGATGCCTCCCGACACCGGGAAG | 65745 | 0.1398266850949922 | No Hit |
| CTGGCGTTGGCAAGCGTCGCAGAATTCACTACGACGCCAAGGCCATACAT | 64514 | 0.1372085901926888 | No Hit |
| GAAAATTTCTTGCTTCCCGGTGTCGGGAGGCATCTCTATCTCGTGGCAAC | 58472 | 0.12435844445774405 | No Hit |
| ATTTCAATGAGTTGAATACAAATGATTCTTCGAGTAGCAAGGAGAGGGCA | 55691 | 0.11844380439007086 | No Hit |
| GTTATTTCAATGAGTTGAATACAAATGATTCTTCGAGTAGCAAGGAGAGG | 49043 | 0.10430481583563316 | No Hit |
| GCGCCATCCATTTTCAGGGCTAGTTGATTCGGCAGGTGAGTTGTTACACA | 48994 | 0.10420060247234082 | No Hit |
| GCGTTGGCAAGCGTCGCAGAATTCACTACGACGCCAAGGCCATACATAGG | 48611 | 0.1033860367959946 | No Hit |
| CTACCATTACGATCTGCACTGACGGAAGCTCCAGCCGAGCCTACGCTCAT | 47154 | 0.10028728434054697 | No Hit |

## Adapter Content

Produced by FastQC (version 0.12.1)
