# Supplementary material for: The pos-1 3′ untranslated region governs germline specification and proliferation to ensure reproductive robustness
Source: PLoS Genet. 2026 Apr 27;22(4):e1012129. doi: 10.1371/journal.pgen.1012129 (PMC13132445; doi:10.1371/journal.pgen.1012129)
Supplement: S1 Data — (GZ) [file pgen.1012129.s007.gz › SupplementalDataSet2/OneStopRNASeq/fastqc.zip!/fastqc/details_trimmed/DG4222_20_rep3.R2_fastqc.html]

DG4222\_20\_rep3.R2.fastq.gz FastQC Report 

FastQC Report

Wed 12 Feb 2025  
DG4222\_20\_rep3.R2.fastq.gz

## Summary

- Basic Statistics
- Per base sequence quality
- Per tile sequence quality
- Per sequence quality scores
- Per base sequence content
- Per sequence GC content
- Per base N content
- Sequence Length Distribution
- Sequence Duplication Levels
- Overrepresented sequences
- Adapter Content

## Basic Statistics

| Measure | Value |
| --- | --- |
| Filename | DG4222\_20\_rep3.R2.fastq.gz |
| File type | Conventional base calls |
| Encoding | Sanger / Illumina 1.9 |
| Total Sequences | 54155108 |
| Total Bases | 2.7 Gbp |
| Sequences flagged as poor quality | 0 |
| Sequence length | 15-51 |
| %GC | 48 |

## Per base sequence quality

## Per tile sequence quality

## Per sequence quality scores

## Per base sequence content

## Per sequence GC content

## Per base N content

## Sequence Length Distribution

## Sequence Duplication Levels

## Overrepresented sequences

| Sequence | Count | Percentage | Possible Source |
| --- | --- | --- | --- |
| CCCCACTCTTCTCGAATCAGTTCAGTCCCGGATAGCGACGGGCGGTGTGT | 141205 | 0.26074179373809026 | No Hit |
| GTCGCAACGGCGGAGTATAGGTCTCTCGCTTAAGCGCCATCCATTTTCAG | 108333 | 0.20004207174695324 | No Hit |
| GGAACAGCGGGTTCAAACATTTGGTTCATAGACTTGATCGACAGATCAAT | 93541 | 0.17272793547009452 | No Hit |
| GGGGGGGGGGGGGGGGGGGGGGGGGGGGGGGGGGGGGGGGGGGGGGGGGG | 91226 | 0.16845317712227625 | No Hit |
| CAAACGTGGAGTTAAGGTGCCTAACTTCTCGCTCATGAGACCCCATAAA | 90553 | 0.16721045039740295 | No Hit |
| CAGAAATCAGTGACAGTCAACGTCCGCAAGGACCATCTGTCACCTCTGTT | 77442 | 0.1430003611109039 | No Hit |
| ATAAGCTCTCGTGTTGCCACGAGATAGAGATGCCTCCCGACACCGGGAAG | 74813 | 0.13814578672800357 | No Hit |
| GGGTATTGTAATTATTGCCCTTAAACGAGGAATGCCTAGTAAGTGTGAGT | 74584 | 0.1377229272629278 | No Hit |
| CGAAAATTTCTTGCTTCCCGGTGTCGGGAGGCATCTCTATCTCGTGGCAA | 73146 | 0.13506759140799793 | No Hit |
| CCGACATTGAACCAGAGTGATCTGGCGTTGGCAAGCGTCGCAGAATTCAC | 66131 | 0.12211405801277324 | No Hit |
| CTCGCCTTAGGACACCTACGTTACGATTTGATAGATGTACCGCCCCAGTC | 65382 | 0.1207309936488355 | No Hit |
| CGAAAAGCTTGCTTTTAGTCCCCGCTTGTCGAAAGGGAATAGGGTTAATA | 64803 | 0.1196618424249103 | No Hit |
| GGAAGCTCCAGCCGAGCCTACGCTCATAGCCTTCAACGCAACCGCCACGA | 62347 | 0.11512672082566984 | No Hit |
| GTGGGAGTCTTCGGACGACAGTGAAATACCACCACTTTCATCGACTCTTT | 59364 | 0.1096184684923904 | No Hit |
| GTGATTTAGCGCCCCGACATTGAACCAGAGTGATCTGGCGTTGGCAAGCG | 58605 | 0.1082169386496284 | No Hit |
| AAGCTCTCGTGTTGCCACGAGATAGAGATGCCTCCCGACACCGGGAAGCA | 55909 | 0.10323864555860547 | No Hit |
| GCCATTTTTGGTAAGCAGAACTGGCGCTGTGGGATGAACCAAACGTGGAG | 55097 | 0.10173924867807482 | No Hit |
| GCGGGTTCAAACATTTGGTTCATAGACTTGATCGACAGATCAATGGTCTG | 54889 | 0.10135516671853004 | No Hit |
| CTACCATTACGATCTGCACTGACGGAAGCTCCAGCCGAGCCTACGCTCAT | 54528 | 0.10068856293297393 | No Hit |
| GCGCCATCCATTTTCAGGGCTAGTTGATTCGGCAGGTGAGTTGTTACACA | 54177 | 0.10004042462624209 | No Hit |

## Adapter Content

Produced by FastQC (version 0.12.1)
