# Supplementary material for: The pos-1 3′ untranslated region governs germline specification and proliferation to ensure reproductive robustness
Source: PLoS Genet. 2026 Apr 27;22(4):e1012129. doi: 10.1371/journal.pgen.1012129 (PMC13132445; doi:10.1371/journal.pgen.1012129)
Supplement: S1 Data — (GZ) [file pgen.1012129.s007.gz › SupplementalDataSet2/OneStopRNASeq/fastqc.zip!/fastqc/details_trimmed/WRM101_20_rep3.R2_fastqc.html]

WRM101\_20\_rep3.R2.fastq.gz FastQC Report 

FastQC Report

Wed 12 Feb 2025  
WRM101\_20\_rep3.R2.fastq.gz

## Summary

- Basic Statistics
- Per base sequence quality
- Per tile sequence quality
- Per sequence quality scores
- Per base sequence content
- Per sequence GC content
- Per base N content
- Sequence Length Distribution
- Sequence Duplication Levels
- Overrepresented sequences
- Adapter Content

## Basic Statistics

| Measure | Value |
| --- | --- |
| Filename | WRM101\_20\_rep3.R2.fastq.gz |
| File type | Conventional base calls |
| Encoding | Sanger / Illumina 1.9 |
| Total Sequences | 76450436 |
| Total Bases | 3.8 Gbp |
| Sequences flagged as poor quality | 0 |
| Sequence length | 15-51 |
| %GC | 49 |

## Per base sequence quality

## Per tile sequence quality

## Per sequence quality scores

## Per base sequence content

## Per sequence GC content

## Per base N content

## Sequence Length Distribution

## Sequence Duplication Levels

## Overrepresented sequences

| Sequence | Count | Percentage | Possible Source |
| --- | --- | --- | --- |
| CCCCACTCTTCTCGAATCAGTTCAGTCCCGGATAGCGACGGGCGGTGTGT | 416128 | 0.5443108264287727 | No Hit |
| GGGTATTGTAATTATTGCCCTTAAACGAGGAATGCCTAGTAAGTGTGAGT | 310757 | 0.4064816582602616 | No Hit |
| CGAAAATTTCTTGCTTCCCGGTGTCGGGAGGCATCTCTATCTCGTGGCAA | 283069 | 0.37026472942548033 | No Hit |
| GCGACATGTGCGTTGTCTAGCGCCAGGTCGTAACGAGTAGGAAGGTCGTG | 258886 | 0.3386324703236486 | No Hit |
| GTCGCAACGGCGGAGTATAGGTCTCTCGCTTAAGCGCCATCCATTTTCAG | 251656 | 0.3291753627147398 | No Hit |
| GGGGGGGGGGGGGGGGGGGGGGGGGGGGGGGGGGGGGGGGGGGGGGGGGG | 248066 | 0.32447950983562734 | No Hit |
| GCTACTACCATTACGATCTGCACTGACGGAAGCTCCAGCCGAGCCTACGC | 240027 | 0.3139642002826511 | No Hit |
| CCGACATTGAACCAGAGTGATCTGGCGTTGGCAAGCGTCGCAGAATTCAC | 211660 | 0.27685911431558086 | No Hit |
| GCGAAAATTTCTTGCTTCCCGGTGTCGGGAGGCATCTCTATCTCGTGGCA | 205682 | 0.2690396690477998 | No Hit |
| GTGATTTAGCGCCCCGACATTGAACCAGAGTGATCTGGCGTTGGCAAGCG | 197365 | 0.2581607252050204 | No Hit |
| ATTTCAATGAGTTGAATACAAATGATTCTTCGAGTAGCAAGGAGAGGGCA | 177366 | 0.23200129296842728 | No Hit |
| CTACCATTACGATCTGCACTGACGGAAGCTCCAGCCGAGCCTACGCTCAT | 177305 | 0.23192150271059275 | No Hit |
| GGAACAGCGGGTTCAAACATTTGGTTCATAGACTTGATCGACAGATCAAT | 174560 | 0.22833094110804025 | No Hit |
| GGCGAAAATTTCTTGCTTCCCGGTGTCGGGAGGCATCTCTATCTCGTGGC | 169396 | 0.22157623796939496 | No Hit |
| GCGGAGTATAGGTCTCTCGCTTAAGCGCCATCCATTTTCAGGGCTAGTTG | 168990 | 0.22104517494184076 | No Hit |
| ATAAGCTCTCGTGTTGCCACGAGATAGAGATGCCTCCCGACACCGGGAAG | 160956 | 0.2105364055739329 | No Hit |
| GCGTTGTCTAGCGCCAGGTCGTAACGAGTAGGAAGGTCGTGGCGGTTGCG | 157249 | 0.20568751236421987 | No Hit |
| CTATACTCCGCCGTTGCGACATGTGCGTTGTCTAGCGCCAGGTCGTAACG | 147869 | 0.19341812517589826 | No Hit |
| GTTTAGATGAGCGATCAGGATACTAGAACCGAGGTCCAAGCACACACC | 145119 | 0.18982102338827733 | No Hit |
| GAAAATTTCTTGCTTCCCGGTGTCGGGAGGCATCTCTATCTCGTGGCAAC | 143822 | 0.18812449938153394 | No Hit |
| CCCACTCTTCTCGAATCAGTTCAGTCCCGGATAGCGACGGGCGGTGTGTA | 140808 | 0.1841820758223014 | No Hit |
| TTCCGGATAGCTCCTCGGCAGGGCAAAAACCCTCCGAATCGCGAGATGGC | 139906 | 0.18300222643596173 | No Hit |
| GCAATAACGAGATACACTAGGAGAGCTGGAATTACCGCGGCTGCTGGCAC | 139787 | 0.18284657003133378 | No Hit |
| CGAAAGTCAGAGGTTCGAAGGCGATTAGATACCGCCCTAGTTCTGACCGT | 135388 | 0.17709251520815394 | No Hit |
| CCGGATAGCTCCTCGGCAGGGCAAAAACCCTCCGAATCGCGAGATGGCAT | 132463 | 0.17326650694313894 | No Hit |
| GCGCCATCCATTTTCAGGGCTAGTTGATTCGGCAGGTGAGTTGTTACACA | 130914 | 0.1712403576089481 | No Hit |
| GTTATTTCAATGAGTTGAATACAAATGATTCTTCGAGTAGCAAGGAGAGG | 125631 | 0.16432999806567486 | No Hit |
| CTCCGCTTAATGCTTCGAAAAAAGGCCGTGAAGCCAATTCTCTTTTAACC | 122812 | 0.16064264172410997 | No Hit |
| GGAATGCCTAGTAAGTGTGAGTCATCAGCTCACGTTGATTACGTCCCTGC | 118848 | 0.15545758300188112 | No Hit |
| GTGGGAGTCTTCGGACGACAGTGAAATACCACCACTTTCATCGACTCTTT | 116676 | 0.1526165266081674 | No Hit |
| CTGGCGTTGGCAAGCGTCGCAGAATTCACTACGACGCCAAGGCCATACAT | 116556 | 0.15245956216652579 | No Hit |
| CGGATAGCTCCTCGGCAGGGCAAAAACCCTCCGAATCGCGAGATGGCATC | 109878 | 0.14372449098916845 | No Hit |
| GTAATTATTGCCCTTAAACGAGGAATGCCTAGTAAGTGTGAGTCATCAGC | 109429 | 0.14313718237002598 | No Hit |
| TCCGGATAGCTCCTCGGCAGGGCAAAAACCCTCCGAATCGCGAGATGGCA | 107204 | 0.14022680001458723 | No Hit |
| TGCTACTACCATTACGATCTGCACTGACGGAAGCTCCAGCCGAGCCTACG | 105893 | 0.13851196348965228 | No Hit |
| CCGCTTAATGCTTCGAAAAAAGGCCGTGAAGCCAATTCTCTTTTAACCGA | 105218 | 0.13762903850541808 | No Hit |
| GTCGTATGCAAGTGATTTAGCGCCCCGACATTGAACCAGAGTGATCTGGC | 104801 | 0.13708358707071336 | No Hit |
| CGGGAGGCATCTCTATCTCGTGGCAACACGAGAGCTTATGCCCTATGTAT | 103800 | 0.13577424202001936 | No Hit |
| GCGGGTTCAAACATTTGGTTCATAGACTTGATCGACAGATCAATGGTCTG | 101544 | 0.1328233105171565 | No Hit |
| AAAATTTCTTGCTTCCCGGTGTCGGGAGGCATCTCTATCTCGTGGCAACA | 98043 | 0.12824387293226164 | No Hit |
| GCGGACTAGTGATTGTGCTTGCTTGCGGACGCTTTCTGGTGTGTGCTTGG | 96156 | 0.12577560708744684 | No Hit |
| CGCAGCAATAACGAGATACACTAGGAGAGCTGGAATTACCGCGGCTGCTG | 95491 | 0.12490576247334939 | No Hit |
| CCCGACATTGAACCAGAGTGATCTGGCGTTGGCAAGCGTCGCAGAATTCA | 91996 | 0.12033417311053661 | No Hit |
| CAAACGTGGAGTTAAGGTGCCTAACTTCTCGCTCATGAGACCCCATAAA | 91620 | 0.11984235119339282 | No Hit |
| GGGAGGCATCTCTATCTCGTGGCAACACGAGAGCTTATGCCCTATGTATG | 87138 | 0.11397972929807752 | No Hit |
| CTCGAAGCGACAGTCCCCACTCTTCTCGAATCAGTTCAGTCCCGGATAGC | 86833 | 0.11358077800890502 | No Hit |
| CCACTCTTCTCGAATCAGTTCAGTCCCGGATAGCGACGGGCGGTGTGTAC | 86722 | 0.11343558590038649 | No Hit |
| GCATAAGCTCTCGTGTTGCCACGAGATAGAGATGCCTCCCGACACCGGGA | 86543 | 0.11320144727493772 | No Hit |
| CGGCGGAGTATAGGTCTCTCGCTTAAGCGCCATCCATTTTCAGGGCTAGT | 85684 | 0.11207784348018629 | No Hit |
| GCGTTGGCAAGCGTCGCAGAATTCACTACGACGCCAAGGCCATACATAGG | 84292 | 0.11025705595714326 | No Hit |
| CCCGGACATGGAAGAATTTCAACGGTTTACCGATACCTTTCGGCATAGGA | 83699 | 0.10948139000803082 | No Hit |
| CCGGGATAGGGTATTGTAATTATTGCCCTTAAACGAGGAATGCCTAGTA | 83483 | 0.10919885401307589 | No Hit |
| GGGACAGTAGGAATCTCGTTAATCCATTCATGCGCGTCAACAATTAAATG | 82871 | 0.10839833536070351 | No Hit |
| CTGGAATTACCGCGGCTGCTGGCACCAGACTTGCCCTCTCCTTGCTACTC | 81681 | 0.10684177131442389 | No Hit |
| CGAAAAGCTTGCTTTTAGTCCCCGCTTGTCGAAAGGGAATAGGGTTAATA | 81333 | 0.10638657443366314 | No Hit |
| GGAAGCTCCAGCCGAGCCTACGCTCATAGCCTTCAACGCAACCGCCACGA | 81105 | 0.10608834199454403 | No Hit |
| TGAGACCCCATAAAAGGTGTTGGTTGATATTGACAGCAGGACGGTGGCCA | 80503 | 0.10530090371230845 | No Hit |
| AAATTTCTTGCTTCCCGGTGTCGGGAGGCATCTCTATCTCGTGGCAACAC | 80478 | 0.10526820278696646 | No Hit |
| AAGCTCTCGTGTTGCCACGAGATAGAGATGCCTCCCGACACCGGGAAGCA | 79877 | 0.10448207254174456 | No Hit |
| CTCGCCTTAGGACACCTACGTTACGATTTGATAGATGTACCGCCCCAGTC | 78292 | 0.10240883387506122 | No Hit |
| TTTTGGATGAGTTATTTCAATGAGTTGAATACAAATGATTCTTCGAGTAG | 77499 | 0.10137156052321271 | No Hit |

## Adapter Content

Produced by FastQC (version 0.12.1)
