# Supplementary material for: The pos-1 3′ untranslated region governs germline specification and proliferation to ensure reproductive robustness
Source: PLoS Genet. 2026 Apr 27;22(4):e1012129. doi: 10.1371/journal.pgen.1012129 (PMC13132445; doi:10.1371/journal.pgen.1012129)
Supplement: S1 Data — (GZ) [file pgen.1012129.s007.gz › SupplementalDataSet2/OneStopRNASeq/fastqc.zip!/fastqc/details_trimmed/WRM101_20_rep3.R1_fastqc.html]

WRM101\_20\_rep3.R1.fastq.gz FastQC Report 

FastQC Report

Wed 12 Feb 2025  
WRM101\_20\_rep3.R1.fastq.gz

## Summary

- Basic Statistics
- Per base sequence quality
- Per tile sequence quality
- Per sequence quality scores
- Per base sequence content
- Per sequence GC content
- Per base N content
- Sequence Length Distribution
- Sequence Duplication Levels
- Overrepresented sequences
- Adapter Content

## Basic Statistics

| Measure | Value |
| --- | --- |
| Filename | WRM101\_20\_rep3.R1.fastq.gz |
| File type | Conventional base calls |
| Encoding | Sanger / Illumina 1.9 |
| Total Sequences | 76450436 |
| Total Bases | 3.8 Gbp |
| Sequences flagged as poor quality | 0 |
| Sequence length | 15-51 |
| %GC | 49 |

## Per base sequence quality

## Per tile sequence quality

## Per sequence quality scores

## Per base sequence content

## Per sequence GC content

## Per base N content

## Sequence Length Distribution

## Sequence Duplication Levels

## Overrepresented sequences

| Sequence | Count | Percentage | Possible Source |
| --- | --- | --- | --- |
| CCCCACTCTTCTCGAATCAGTTCAGTCCCGGATAGCGACGGGCGGTGTGT | 381214 | 0.49864202213313735 | No Hit |
| GGGTATTGTAATTATTGCCCTTAAACGAGGAATGCCTAGTAAGTGTGAGT | 305370 | 0.39943526286756564 | No Hit |
| CGAAAATTTCTTGCTTCCCGGTGTCGGGAGGCATCTCTATCTCGTGGCAA | 279762 | 0.3659390510212394 | No Hit |
| GCGACATGTGCGTTGTCTAGCGCCAGGTCGTAACGAGTAGGAAGGTCGTG | 246572 | 0.3225253025371889 | No Hit |
| GCTACTACCATTACGATCTGCACTGACGGAAGCTCCAGCCGAGCCTACGC | 229182 | 0.2997785388692878 | No Hit |
| CCGACATTGAACCAGAGTGATCTGGCGTTGGCAAGCGTCGCAGAATTCAC | 203920 | 0.26673490782969506 | No Hit |
| GCGAAAATTTCTTGCTTCCCGGTGTCGGGAGGCATCTCTATCTCGTGGCA | 203414 | 0.2660730411007728 | No Hit |
| GTGATTTAGCGCCCCGACATTGAACCAGAGTGATCTGGCGTTGGCAAGCG | 192061 | 0.25122289688445987 | No Hit |
| GTCGCAACGGCGGAGTATAGGTCTCTCGCTTAAGCGCCATCCATTTTCAG | 186526 | 0.2439829120137392 | No Hit |
| ATTTCAATGAGTTGAATACAAATGATTCTTCGAGTAGCAAGGAGAGGGCA | 179550 | 0.23485804580630512 | No Hit |
| GGAACAGCGGGTTCAAACATTTGGTTCATAGACTTGATCGACAGATCAAT | 173689 | 0.2271916408691247 | No Hit |
| CTACCATTACGATCTGCACTGACGGAAGCTCCAGCCGAGCCTACGCTCAT | 171566 | 0.22441467828908132 | No Hit |
| GGCGAAAATTTCTTGCTTCCCGGTGTCGGGAGGCATCTCTATCTCGTGGC | 165452 | 0.21641733998743973 | No Hit |
| GCGTTGTCTAGCGCCAGGTCGTAACGAGTAGGAAGGTCGTGGCGGTTGCG | 154445 | 0.20201977657786022 | No Hit |
| ATAAGCTCTCGTGTTGCCACGAGATAGAGATGCCTCCCGACACCGGGAAG | 150950 | 0.1974481872150474 | No Hit |
| GCGGAGTATAGGTCTCTCGCTTAAGCGCCATCCATTTTCAGGGCTAGTTG | 147467 | 0.19289229429639876 | No Hit |
| CTATACTCCGCCGTTGCGACATGTGCGTTGTCTAGCGCCAGGTCGTAACG | 145624 | 0.19048158208018592 | No Hit |
| CCCACTCTTCTCGAATCAGTTCAGTCCCGGATAGCGACGGGCGGTGTGTA | 143642 | 0.18788905271907147 | No Hit |
| GTTTAGATGAGCGATCAGGATACTAGAACCGAGGTCCAAGCACACACC | 142713 | 0.18667388633336243 | No Hit |
| CGAAAGTCAGAGGTTCGAAGGCGATTAGATACCGCCCTAGTTCTGACCGT | 141976 | 0.18570986305428003 | No Hit |
| GAAAATTTCTTGCTTCCCGGTGTCGGGAGGCATCTCTATCTCGTGGCAAC | 137147 | 0.17939335231521766 | No Hit |
| GTTATTTCAATGAGTTGAATACAAATGATTCTTCGAGTAGCAAGGAGAGG | 135448 | 0.17717099742897477 | No Hit |
| GCAATAACGAGATACACTAGGAGAGCTGGAATTACCGCGGCTGCTGGCAC | 128276 | 0.16778975596685936 | No Hit |
| CCGGATAGCTCCTCGGCAGGGCAAAAACCCTCCGAATCGCGAGATGGCAT | 125092 | 0.16362496611530117 | No Hit |
| GCGCCATCCATTTTCAGGGCTAGTTGATTCGGCAGGTGAGTTGTTACACA | 125065 | 0.1635896491159318 | No Hit |
| GGAATGCCTAGTAAGTGTGAGTCATCAGCTCACGTTGATTACGTCCCTGC | 116935 | 0.15295530819471062 | No Hit |
| CTGGCGTTGGCAAGCGTCGCAGAATTCACTACGACGCCAAGGCCATACAT | 115162 | 0.15063615856945536 | No Hit |
| GTGGGAGTCTTCGGACGACAGTGAAATACCACCACTTTCATCGACTCTTT | 114788 | 0.15014695272633893 | No Hit |
| CTCCGCTTAATGCTTCGAAAAAAGGCCGTGAAGCCAATTCTCTTTTAACC | 113222 | 0.14809856676291552 | No Hit |
| GTAATTATTGCCCTTAAACGAGGAATGCCTAGTAAGTGTGAGTCATCAGC | 108540 | 0.14197433746486415 | No Hit |
| CGGATAGCTCCTCGGCAGGGCAAAAACCCTCCGAATCGCGAGATGGCATC | 105453 | 0.13793642720363294 | No Hit |
| TTCCGGATAGCTCCTCGGCAGGGCAAAAACCCTCCGAATCGCGAGATGGC | 104108 | 0.1361771174202329 | No Hit |
| CCGCTTAATGCTTCGAAAAAAGGCCGTGAAGCCAATTCTCTTTTAACCGA | 101759 | 0.13310453847509776 | No Hit |
| CGGGAGGCATCTCTATCTCGTGGCAACACGAGAGCTTATGCCCTATGTAT | 101294 | 0.13249630126373643 | No Hit |
| GCGGGTTCAAACATTTGGTTCATAGACTTGATCGACAGATCAATGGTCTG | 100293 | 0.1311869562130424 | No Hit |
| TGCTACTACCATTACGATCTGCACTGACGGAAGCTCCAGCCGAGCCTACG | 98964 | 0.12944857502186122 | No Hit |
| GTCGTATGCAAGTGATTTAGCGCCCCGACATTGAACCAGAGTGATCTGGC | 94698 | 0.1238684891215009 | No Hit |
| GCGGACTAGTGATTGTGCTTGCTTGCGGACGCTTTCTGGTGTGTGCTTGG | 93215 | 0.12192867023021293 | No Hit |
| AAAATTTCTTGCTTCCCGGTGTCGGGAGGCATCTCTATCTCGTGGCAACA | 92201 | 0.12060232069834108 | No Hit |
| CAAACGTGGAGTTAAGGTGCCTAACTTCTCGCTCATGAGACCCCATAAA | 91221 | 0.11932044442493435 | No Hit |
| CCCGACATTGAACCAGAGTGATCTGGCGTTGGCAAGCGTCGCAGAATTCA | 89318 | 0.11683124998790066 | No Hit |
| CGCAGCAATAACGAGATACACTAGGAGAGCTGGAATTACCGCGGCTGCTG | 88026 | 0.11514126616622565 | No Hit |
| TGAGACCCCATAAAAGGTGTTGGTTGATATTGACAGCAGGACGGTGGCCA | 84561 | 0.11060891791382328 | No Hit |
| CTCGAAGCGACAGTCCCCACTCTTCTCGAATCAGTTCAGTCCCGGATAGC | 84399 | 0.11039701591760706 | No Hit |
| CCACTCTTCTCGAATCAGTTCAGTCCCGGATAGCGACGGGCGGTGTGTAC | 84251 | 0.11020342643958238 | No Hit |
| TCCGGATAGCTCCTCGGCAGGGCAAAAACCCTCCGAATCGCGAGATGGCA | 83476 | 0.1091896977539801 | No Hit |
| CCCGGACATGGAAGAATTTCAACGGTTTACCGATACCTTTCGGCATAGGA | 83334 | 0.10900395649803751 | No Hit |
| CGAAAAGCTTGCTTTTAGTCCCCGCTTGTCGAAAGGGAATAGGGTTAATA | 83079 | 0.10867040705954902 | No Hit |
| GGGAGGCATCTCTATCTCGTGGCAACACGAGAGCTTATGCCCTATGTATG | 82846 | 0.1083656344353615 | No Hit |
| GCATAAGCTCTCGTGTTGCCACGAGATAGAGATGCCTCCCGACACCGGGA | 82690 | 0.10816158066122737 | No Hit |
| GCGTTGGCAAGCGTCGCAGAATTCACTACGACGCCAAGGCCATACATAGG | 81266 | 0.10629893595374657 | No Hit |
| GGGACAGTAGGAATCTCGTTAATCCATTCATGCGCGTCAACAATTAAATG | 80035 | 0.10468874238990607 | No Hit |
| CCGGGATAGGGTATTGTAATTATTGCCCTTAAACGAGGAATGCCTAGTA | 79568 | 0.10407788910451735 | No Hit |
| CAAGAACGAAAGTCAGAGGTTCGAAGGCGATTAGATACCGCCCTAGTTCT | 79009 | 0.10334669641387004 | No Hit |
| CTGGAATTACCGCGGCTGCTGGCACCAGACTTGCCCTCTCCTTGCTACTC | 78236 | 0.10233558380229513 | No Hit |
| TTTTGGATGAGTTATTTCAATGAGTTGAATACAAATGATTCTTCGAGTAG | 76823 | 0.1004873275019648 | No Hit |

## Adapter Content

Produced by FastQC (version 0.12.1)
