# Supplementary material for: The pos-1 3′ untranslated region governs germline specification and proliferation to ensure reproductive robustness
Source: PLoS Genet. 2026 Apr 27;22(4):e1012129. doi: 10.1371/journal.pgen.1012129 (PMC13132445; doi:10.1371/journal.pgen.1012129)
Supplement: S1 Data — (GZ) [file pgen.1012129.s007.gz › SupplementalDataSet2/OneStopRNASeq/bam_qc.zip!/bam_qc/samtools_stats_multiqc_report.html]

MultiQC Report


# Toggle navigation v1.22.2

Loading report..

- General Stats
- Samtools
  - Percent mapped
  - Alignment stats
- Software Versions

Toolbox

### MultiQC Toolbox

#### Apply Highlight Samples

+

Regex mode off
help
 Clear

#### Apply Rename Samples

+

Click here for bulk input.

Paste two columns of a tab-delimited table here (eg. from Excel).

First column should be the old name, second column the new name.

Add

Regex mode off
help
 Clear

#### Apply Show / Hide Samples

Hide matching samples

Show only matching samples

+

Regex mode off
help
 Clear

#### Export Plots

- Images
- Data

px

px

Aspect ratio

PNG
SVG

Plot scaling

X

Download the raw data used to create the plots in this report below:

Format:

Tab-separated
Comma-separated
JSON

Note that additional data was saved in `bam_qc/samtools_stats_multiqc_report_data` when this report was generated.

---

##### Choose Plots

 All
 None


---


   Download Plot Images

If you use plots from MultiQC in a publication or presentation, please cite:

> **MultiQC: Summarize analysis results for multiple tools and samples in a single report**  
> *Philip Ewels, Måns Magnusson, Sverker Lundin and Max Käller*  
> Bioinformatics (2016)  
> doi: 10.1093/bioinformatics/btw354  
> PMID: 27312411

#### Save Settings

You can save the toolbox settings for this report to the browser.

 Save


---

#### Load Settings

Choose a saved report profile from the dropdown box below:

[ select ]

Load
 Delete
 Set default
 Clear default

#### Tool Citations

Please remember to cite the tools that you use in your analysis.

To help with this, you can download publication details of the tools mentioned in this report:

List of DOIs

BibTeX file

#### About MultiQC

This report was generated using MultiQC, version 1.22.2

You can see a YouTube video describing how to use MultiQC reports here:
https://youtu.be/qPbIlO\_KWN0

For more information about MultiQC, including other videos and
extensive documentation, please visit http://multiqc.info

You can report bugs, suggest improvements and find the source code for MultiQC on GitHub:
https://github.com/MultiQC/MultiQC

MultiQC is published in Bioinformatics:

> **MultiQC: Summarize analysis results for multiple tools and samples in a single report**  
> *Philip Ewels, Måns Magnusson, Sverker Lundin and Max Käller*  
> Bioinformatics (2016)  
> doi: 10.1093/bioinformatics/btw354  
> PMID: 27312411

---

MultiQC is developed by:

A modular tool to aggregate results from bioinformatics analyses across many samples into a single report.

#### JavaScript Disabled

MultiQC reports use JavaScript for plots and toolbox functions. It looks like
you have JavaScript disabled in your web browser. Please note that many of the report
functions will not work as intended.

Loading report..

Report
generated on 2025-02-12, 11:06 EST
based on data in:

- `/pi/hira.goel-umw/Kai/OneStopRNAseq/users/639/021225_Haik_pos1_UTR_20degrees_03.18.53-02.12.2025/analysis_1/bam_qc/stats/DG4222_20_rep1.stats.txt`
- `/pi/hira.goel-umw/Kai/OneStopRNAseq/users/639/021225_Haik_pos1_UTR_20degrees_03.18.53-02.12.2025/analysis_1/bam_qc/stats/DG4222_20_rep2.stats.txt`
- `/pi/hira.goel-umw/Kai/OneStopRNAseq/users/639/021225_Haik_pos1_UTR_20degrees_03.18.53-02.12.2025/analysis_1/bam_qc/stats/DG4222_20_rep3.stats.txt`
- `/pi/hira.goel-umw/Kai/OneStopRNAseq/users/639/021225_Haik_pos1_UTR_20degrees_03.18.53-02.12.2025/analysis_1/bam_qc/stats/WRM101_20_rep1.stats.txt`
- `/pi/hira.goel-umw/Kai/OneStopRNAseq/users/639/021225_Haik_pos1_UTR_20degrees_03.18.53-02.12.2025/analysis_1/bam_qc/stats/WRM101_20_rep2.stats.txt`
- `/pi/hira.goel-umw/Kai/OneStopRNAseq/users/639/021225_Haik_pos1_UTR_20degrees_03.18.53-02.12.2025/analysis_1/bam_qc/stats/WRM101_20_rep3.stats.txt`

---

×
don't show again

**Welcome!** Not sure where to start?  
Watch a tutorial video
  *(6:06)*

## General Statistics

Configure columns
 Table
Export Plot

Created with MultiQC

Copy table

 Configure columns

 Sort by highlight

 Scatter plot

 Violin plot
Export as CSV
Showing ^6^/_6_ rows and ^6^/_7_ columns.

| Sample Name | Error rate | Non-primary | Reads mapped | % Mapped | % Proper pairs | % MapQ 0 reads | Total seqs |
| --- | --- | --- | --- | --- | --- | --- | --- |
| DG4222\_20\_rep1 | 0.00% | 43.3M | 73.3M | 100.0% | 100.0% | 0.7% | 73.3M |
| DG4222\_20\_rep2 | 0.00% | 40.5M | 97.7M | 100.0% | 100.0% | 1.0% | 97.7M |
| DG4222\_20\_rep3 | 0.00% | 55.6M | 107.4M | 100.0% | 100.0% | 0.4% | 107.4M |
| WRM101\_20\_rep1 | 0.00% | 71.9M | 93.0M | 100.0% | 100.0% | 1.4% | 93.0M |
| WRM101\_20\_rep2 | 0.00% | 49.7M | 106.1M | 100.0% | 100.0% | 0.8% | 106.1M |
| WRM101\_20\_rep3 | 0.00% | 147.0M | 151.3M | 100.0% | 100.0% | 1.1% | 151.3M |

×

#### General Statistics: Columns

Uncheck the tick box to hide columns. Click and drag the handle on the left to change order. Table ID: `table-general_stats_table`

Show All
Show None

| Sort | Visible | Group | Column | Description | ID | Scale |
| --- | --- | --- | --- | --- | --- | --- |
| \|\| |  | Samtools: stats | Error rate | Error rate: mismatches (NM) / bases mapped (CIGAR) | `error_rate` |  |
| \|\| |  | Samtools: stats | Non-primary | Non-primary alignments (millions) | `non-primary_alignments` | read\_count |
| \|\| |  | Samtools: stats | Reads mapped | Reads mapped in the bam file (millions) | `reads_mapped` | read\_count |
| \|\| |  | Samtools: stats | % Mapped | % Mapped reads | `reads_mapped_percent` |  |
| \|\| |  | Samtools: stats | % Proper pairs | % Properly paired reads | `reads_properly_paired_percent` |  |
| \|\| |  | Samtools: stats | % MapQ 0 reads | % of reads that are ambiguously placed (MapQ=0) | `reads_MQ0_percent` |  |
| \|\| |  | Samtools: stats | Total seqs | Total sequences in the bam file (millions) | `raw_total_sequences` | read\_count |

Close

## Samtools

*Version:* `1.9`

samtools is a suite of programs for interacting with high-throughput sequencing data.*DOI: 10.1093/bioinformatics/btp352.*

### Percent mapped Help

Alignment metrics from `samtools stats`; mapped vs. unmapped reads vs. reads mapped with MQ0.

For a set of samples that have come from the same multiplexed library,
similar numbers of reads for each sample are expected. Large differences in numbers might
indicate issues during the library preparation process. Whilst large differences in read
numbers may be controlled for in downstream processings (e.g. read count normalisation),
you may wish to consider whether the read depths achieved have fallen below recommended
levels depending on the applications.

Low alignment rates could indicate contamination of samples (e.g. adapter sequences),
low sequencing quality or other artefacts. These can be further investigated in the
sequence level QC (e.g. from FastQC).

Reads mapped with MQ0 often indicate that the reads are ambiguously mapped to multiple
locations in the reference sequence. This can be due to repetitive regions in the genome,
the presence of alternative contigs in the reference, or due to reads that are too short
to be uniquely mapped. These reads are often filtered out in downstream analyses.

Percentages
Export Plot

Created with MultiQC

---

### Alignment stats

This module parses the output from `samtools stats`. All numbers in millions.

Configure columns
 Table
Export Plot

Created with MultiQC

Copy table

 Configure columns

 Sort by highlight

 Scatter plot

 Violin plot
Export as CSV
Showing ^6^/_6_ rows and ^13^/_13_ columns.

| Sample Name | Total sequences | Mapped & paired | Properly paired | Duplicated | QC Failed | Reads MQ0 | Mapped bases (CIGAR) | Bases Trimmed | Duplicated bases | Diff chromosomes | Other orientation | Inward pairs | Outward pairs |
| --- | --- | --- | --- | --- | --- | --- | --- | --- | --- | --- | --- | --- | --- |
| DG4222\_20\_rep1 | 73.3M | 73.3M | 73.3M | 0.0M | 0.0M | 0.5M | 3677.1Mb | 0.0Mb | 0.0Mb | 0.0M | 0.0M | 36.6M | 0.1M |
| DG4222\_20\_rep2 | 97.7M | 97.7M | 97.7M | 0.0M | 0.0M | 1.0M | 4902.0Mb | 0.0Mb | 0.0Mb | 0.0M | 0.0M | 48.8M | 0.1M |
| DG4222\_20\_rep3 | 107.4M | 107.4M | 107.4M | 0.0M | 0.0M | 0.5M | 5395.1Mb | 0.0Mb | 0.0Mb | 0.0M | 0.0M | 53.6M | 0.1M |
| WRM101\_20\_rep1 | 93.0M | 93.0M | 93.0M | 0.0M | 0.0M | 1.3M | 4668.1Mb | 0.0Mb | 0.0Mb | 0.0M | 0.0M | 46.4M | 0.1M |
| WRM101\_20\_rep2 | 106.1M | 106.1M | 106.1M | 0.0M | 0.0M | 0.9M | 5323.6Mb | 0.0Mb | 0.0Mb | 0.0M | 0.0M | 53.0M | 0.1M |
| WRM101\_20\_rep3 | 151.3M | 151.3M | 151.3M | 0.0M | 0.0M | 1.6M | 7590.9Mb | 0.0Mb | 0.0Mb | 0.0M | 0.0M | 75.5M | 0.1M |

×

#### Samtools: stats: Alignment Stats: Columns

Uncheck the tick box to hide columns. Click and drag the handle on the left to change order. Table ID: `table-samtools-stats-dp`

Show All
Show None

| Sort | Visible | Group | Column | Description | ID | Scale |
| --- | --- | --- | --- | --- | --- | --- |
| \|\| |  |  | Total sequences | Total sequences | `raw_total_sequences` | read\_count |
| \|\| |  |  | Mapped & paired | Paired-end technology bit set + both mates mapped | `reads_mapped_and_paired` | read\_count |
| \|\| |  |  | Properly paired | Proper-pair bit set | `reads_properly_paired` | read\_count |
| \|\| |  |  | Duplicated | PCR or optical duplicate bit set | `reads_duplicated` | read\_count |
| \|\| |  |  | QC Failed | QC Failed | `reads_QC_failed` | read\_count |
| \|\| |  |  | Reads MQ0 | Reads mapped and MQ=0 | `reads_MQ0` | read\_count |
| \|\| |  |  | Mapped bases (CIGAR) | Mapped bases (CIGAR) | `bases_mapped_(cigar)` | base\_count |
| \|\| |  |  | Bases Trimmed | Bases Trimmed | `bases_trimmed` | base\_count |
| \|\| |  |  | Duplicated bases | Duplicated bases | `bases_duplicated` | base\_count |
| \|\| |  |  | Diff chromosomes | Pairs on different chromosomes | `pairs_on_different_chromosomes` | read\_count |
| \|\| |  |  | Other orientation | Pairs with other orientation | `pairs_with_other_orientation` | read\_count |
| \|\| |  |  | Inward pairs | Inward oriented pairs | `inward_oriented_pairs` | read\_count |
| \|\| |  |  | Outward pairs | Outward oriented pairs | `outward_oriented_pairs` | read\_count |

Close

---

## Software Versions

Software Versions lists versions of software tools extracted from file contents.

Copy table

| Software | Version |
| --- | --- |
| Samtools | `1.9` |

**MultiQC v1.22.2** 
- Written by Phil Ewels,
available on GitHub.

This report uses Plotly,
jQuery,
jQuery UI,
Bootstrap,
FileSaver.js and
clipboard.js.

×

### Plot Table Data

Select Column

Select Column

Please select two table columns.

Close

×

### Regex Help

Toolbox search strings can behave as regular expressions (regexes). Click a button below to see an example of it in action. Try modifying them yourself in the text box.

`^` (start of string)
`$` (end of string)
`[]` (character choice)
`\d` (shorthand for `[0-9]`)
`\w` (shorthand for `[0-9a-zA-Z_]`)
`.` (any character)
`\.` (literal full stop)
`()` `|` (group / separator)
`*` (prev char 0 or more)
`+` (prev char 1 or more)
`?` (prev char 0 or 1)
`{}` (char num times)
`{,}` (count range)

```
samp_1
samp_1_edited
samp_2
samp_2_edited
samp_3
samp_3_edited
prepended_samp_1
tmp_samp_1_edited
tmpp_samp_1_edited
tmppp_samp_1_edited
#samp_1_edited.tmp
samp_11
samp_11111
```

See regex101.com for a more heavy duty testing suite.

Close
