# Supplementary material for: The pos-1 3′ untranslated region governs germline specification and proliferation to ensure reproductive robustness
Source: PLoS Genet. 2026 Apr 27;22(4):e1012129. doi: 10.1371/journal.pgen.1012129 (PMC13132445; doi:10.1371/journal.pgen.1012129)
Supplement: S1 Data — (GZ) [file pgen.1012129.s007.gz › SupplementalDataSet2/OneStopRNASeq/bam_qc.zip!/bam_qc/QoRTs_MultiPlot/plot-basic.pdf]

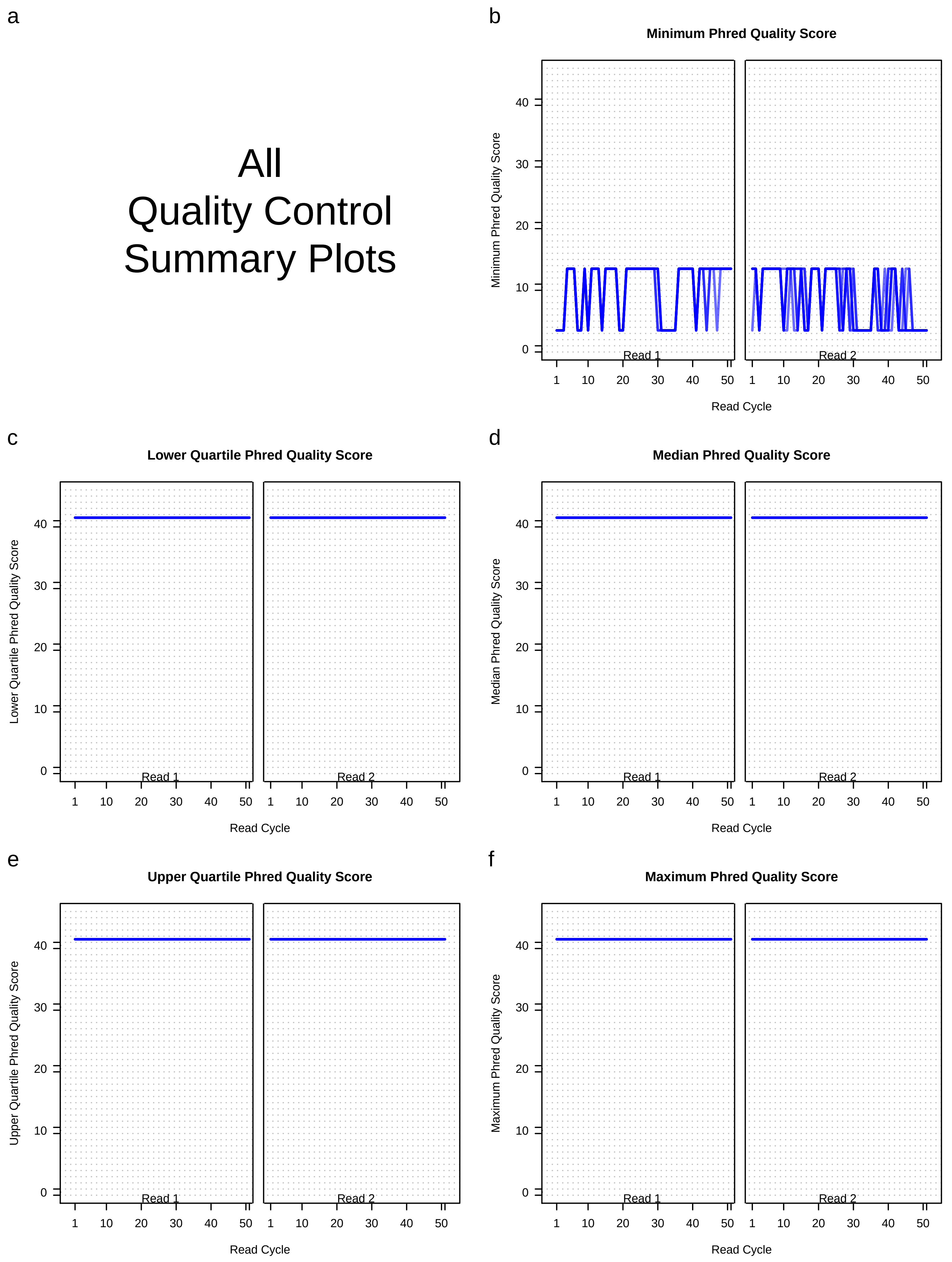
a
All
Quality Control
Summary Plots
b
Minimum Phred Quality Score
Minimum Phred Quality Score
Read 1
Read 2
Read Cycle
c
Lower Quartile Phred Quality Score
Lower Quartile Phred Quality Score
Read 1
Read 2
Read Cycle
d
Median Phred Quality Score
Median Phred Quality Score
Read 1
Read 2
Read Cycle
e
Upper Quartile Phred Quality Score
Upper Quartile Phred Quality Score
Read 1
Read 2
Read Cycle
f
Maximum Phred Quality Score
Maximum Phred Quality Score
Read 1
Read 2
Read Cycle

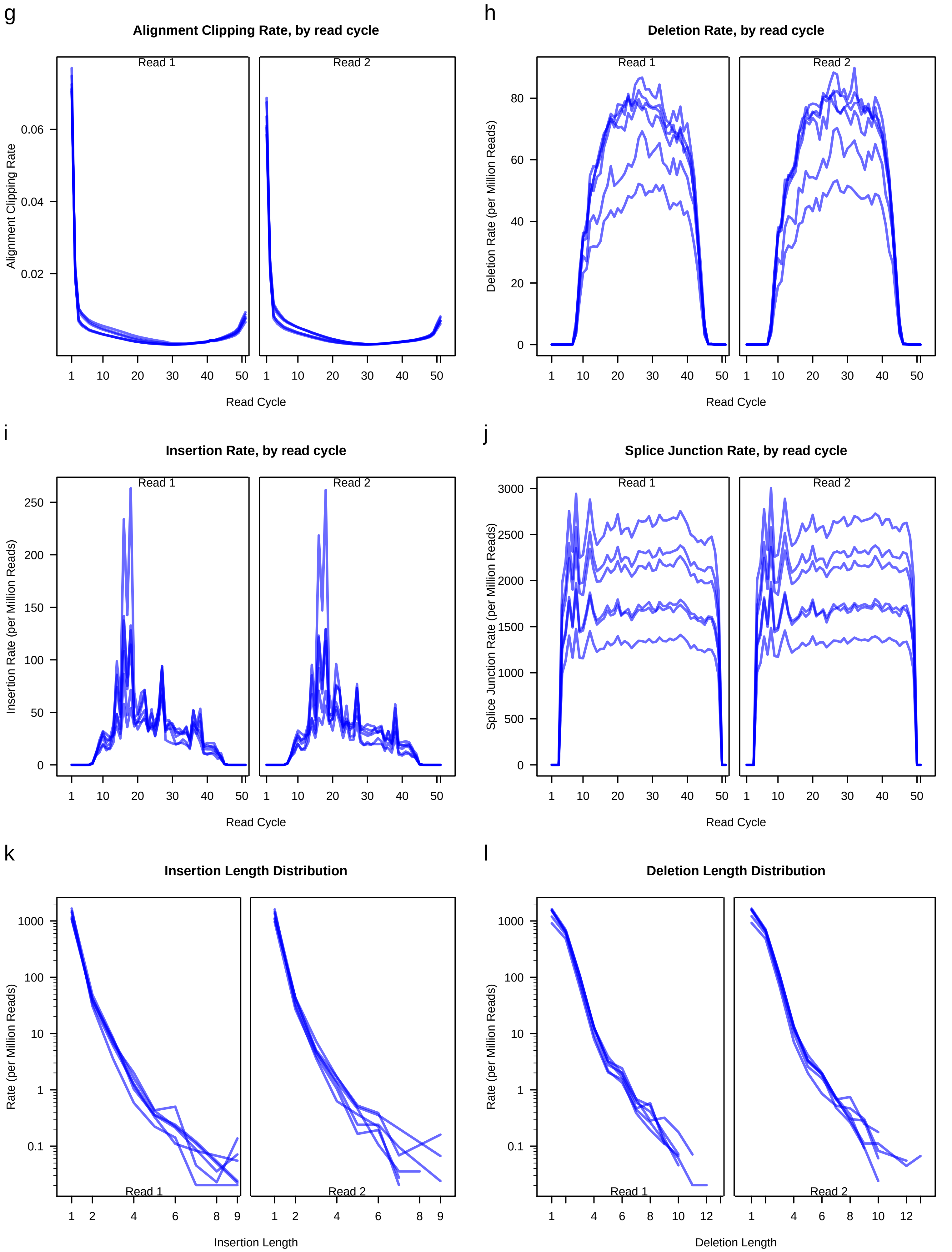
g
Alignment Clipping Rate, by read cycle
Read 1
Read 2
Alignment Clipping Rate
0.02
0.04
0.06
1 10 20 30 40 50
Read Cycle
h
Deletion Rate, by read cycle
Read 1
Read 2
Deletion Rate (per Million Reads)
0 20 40 60 80
1 10 20 30 40 50
Read Cycle
i
Insertion Rate, by read cycle
Read 1
Read 2
Insertion Rate (per Million Reads)
0 50 100 150 200 250
1 10 20 30 40 50
Read Cycle
j
Splice Junction Rate, by read cycle
Read 1
Read 2
Splice Junction Rate (per Million Reads)
0 500 1000 1500 2000 2500 3000
1 10 20 30 40 50
Read Cycle
k
Insertion Length Distribution
Read 1
Read 2
Rate (per Million Reads)
0.1 1 10 100 1000
1 2 4 6 8 9
Insertion Length
l
Deletion Length Distribution
Read 1
Read 2
Rate (per Million Reads)
0.1 1 10 100 1000
1 4 6 8 10 12
Deletion Length

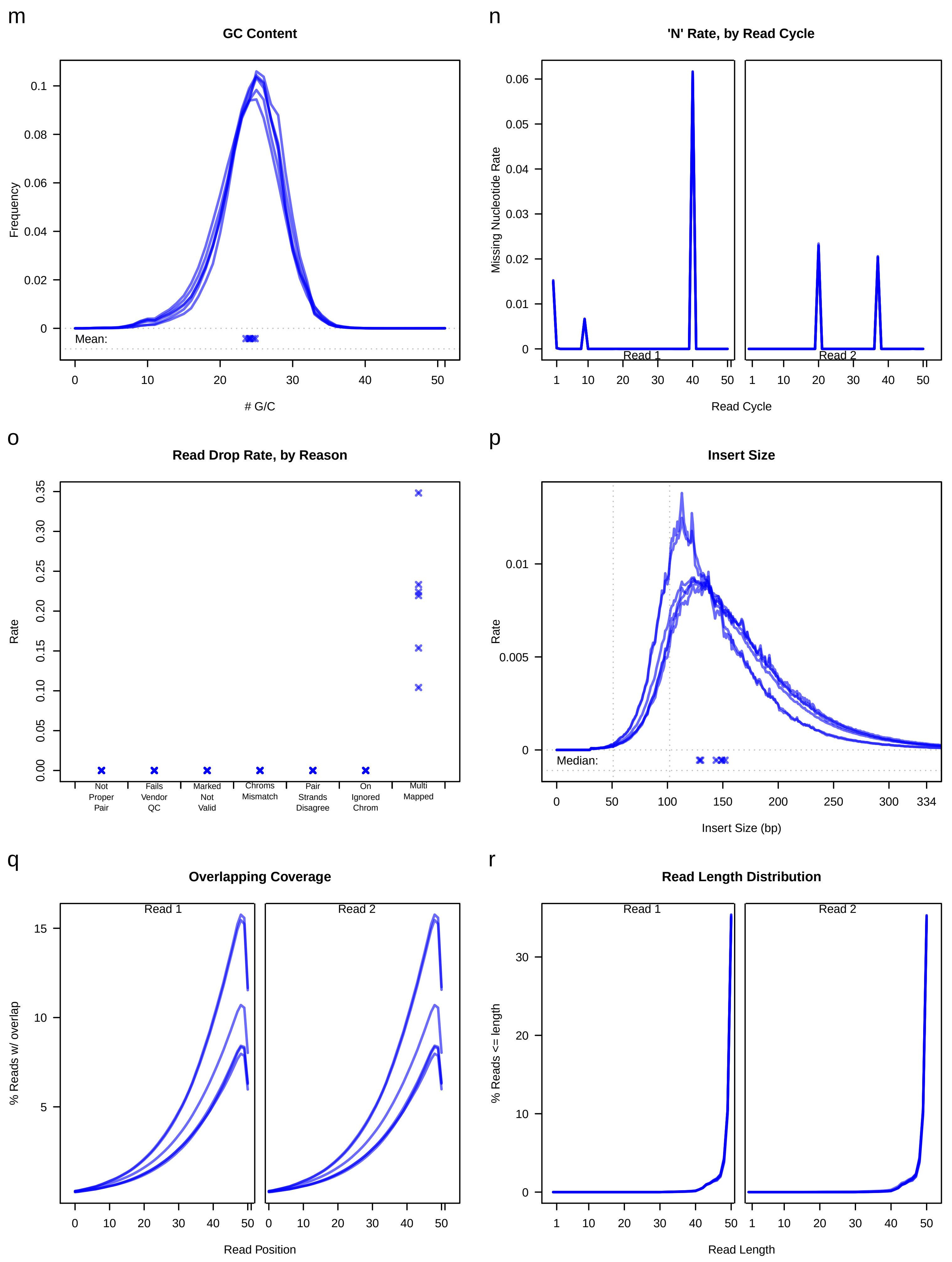

m
GC Content
Frequency
0.1
0.08
0.06
0.04
0.02
0
Mean:
0
10
20
30
40
50
G/C
n
'N' Rate, by Read Cycle
Missing Nucleotide Rate
0.06
0.05
0.04
0.03
0.02
0.01
0
Read 1
Read 2
1
10
20
30
40
50
Read Cycle
o
Read Drop Rate, by Reason
Rate
0.35
0.30
0.25
0.20
0.15
0.10
0.05
0.00
Not Proper Pair
Fails Vendor QC
Marked Not Valid
Chroms Mismatch
Pair Strands Disagree
On Ignored Chrom
Multi Mapped
p
Insert Size
Rate
0.01
0.005
0
Median:
0
50
100
150
200
250
300
334
Insert Size (bp)
q
Overlapping Coverage
% Reads w/ overlap
Read 1
Read 2
15
10
5
0
10
20
30
40
50
Read Position
r
Read Length Distribution
% Reads <= length
Read 1
Read 2
30
20
10
0
1
10
20
30
40
50
Read Length

s

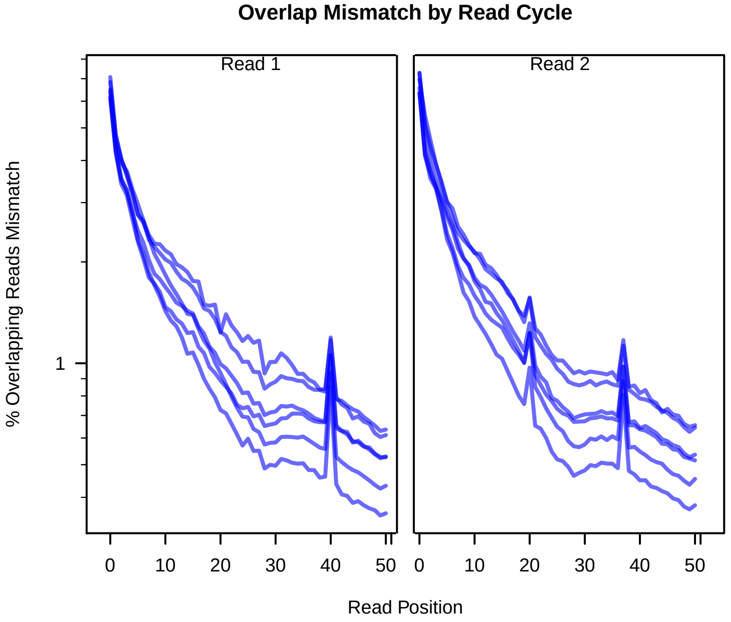
Overlap Mismatch by Read Cycle
Read 1
Read 2
% Overlapping Reads Mismatch
1
0
10
20
30
40
50
Read Position

t

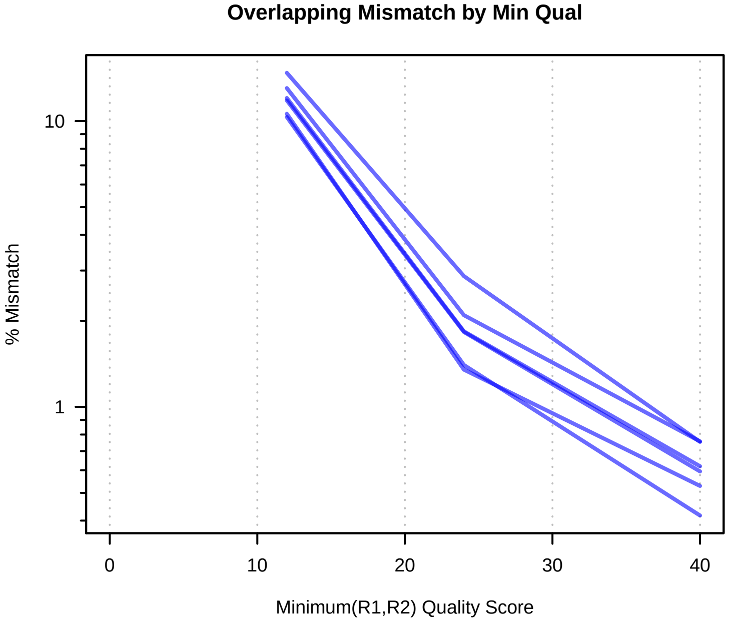
Overlapping Mismatch by Min Qual
% Mismatch
10
1
0
10
20
30
40
Minimum(R1,R2) Quality Score

u

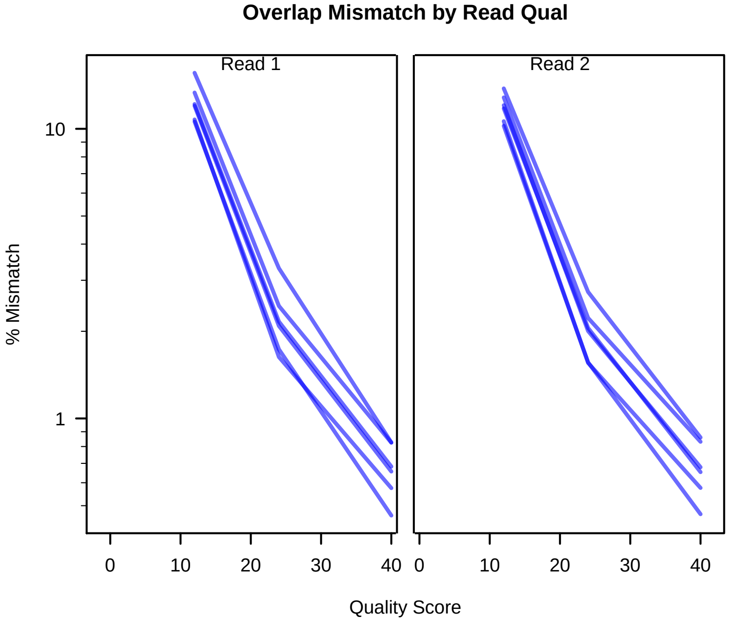
Overlap Mismatch by Read Qual
Read 1
Read 2
% Mismatch
10
1
0
10
20
30
40
Quality Score

v

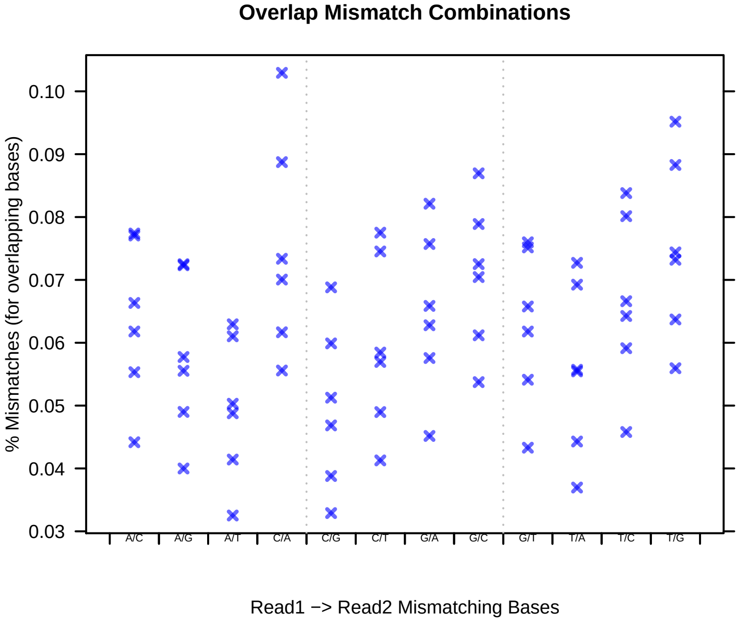
Overlap Mismatch Combinations
% Mismatches (for overlapping bases)
0.10
0.09
0.08
0.07
0.06
0.05
0.04
0.03
A/C
A/G
A/T
C/A
C/G
C/T
G/A
G/C
G/T
T/A
T/C
T/G
Read1 −> Read2 Mismatching Bases

w

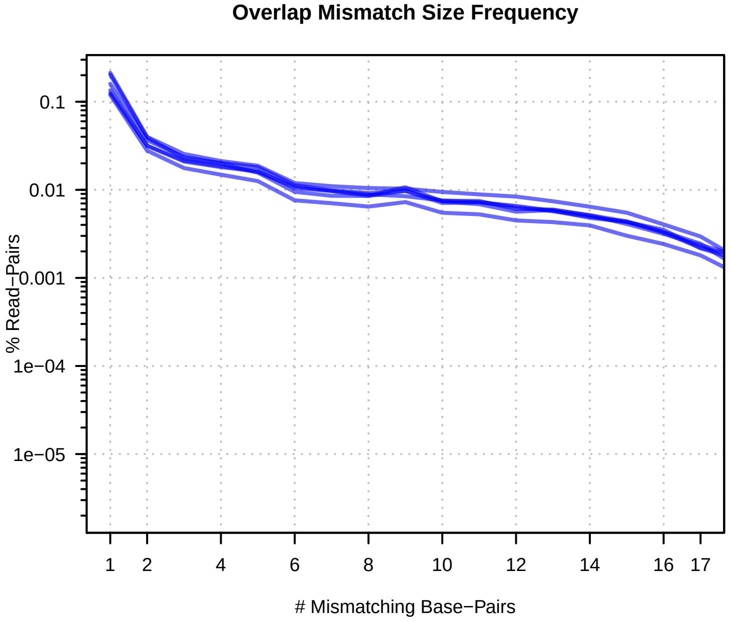
Overlap Mismatch Size Frequency
% Read−Pairs
0.1
0.01
0.001
1e−04
1e−05
1
2
4
6
8
10
12
14
16
17
Mismatching Base−Pairs

x

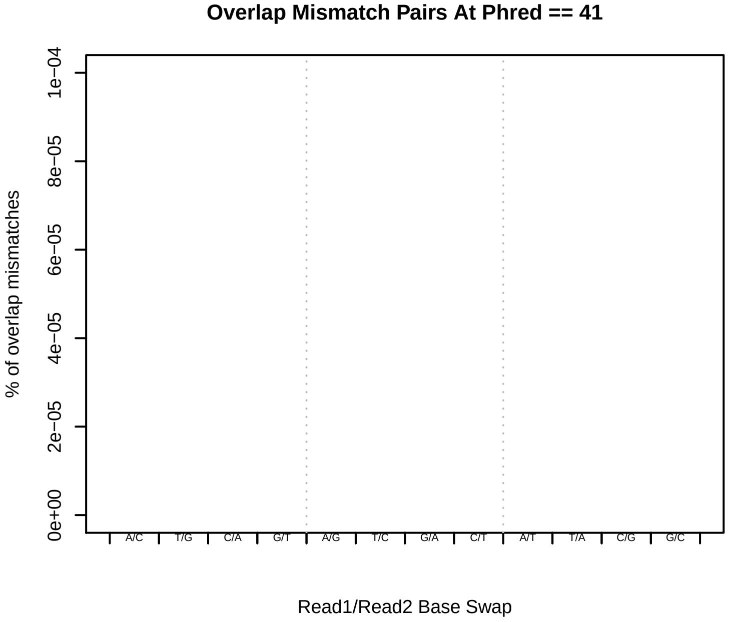
Overlap Mismatch Pairs At Phred == 41
% of overlap mismatches
1e−04
8e−05
6e−05
4e−05
2e−05
0e+00
A/C
T/G
C/A
G/T
A/G
T/C
G/A
C/T
A/T
T/A
C/G
G/C
Read1/Read2 Base Swap

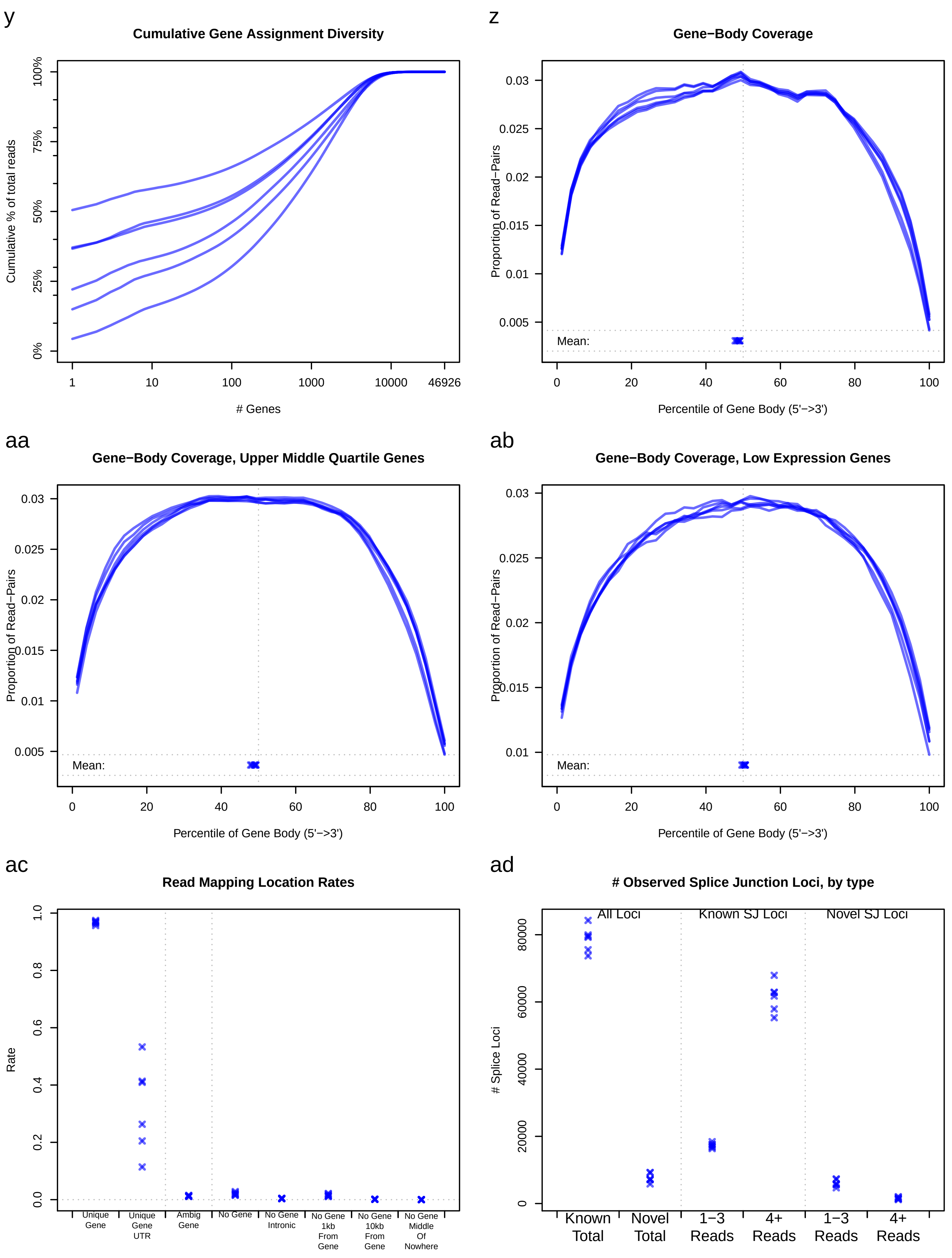
y
Cumulative Gene Assignment Diversity
Cumulative % of total reads
0%
25%
50%
75%
100%
1
10
100
1000
10000
46926
Genes
z
Gene−Body Coverage
Proportion of Read−Pairs
0.005
0.01
0.015
0.02
0.025
0.03
Mean:
0
20
40
60
80
100
Percentile of Gene Body (5'−>3')
aa
Gene−Body Coverage, Upper Middle Quartile Genes
Proportion of Read−Pairs
0.005
0.01
0.015
0.02
0.025
0.03
Mean:
0
20
40
60
80
100
Percentile of Gene Body (5'−>3')
ab
Gene−Body Coverage, Low Expression Genes
Proportion of Read−Pairs
0.01
0.015
0.02
0.025
0.03
Mean:
0
20
40
60
80
100
Percentile of Gene Body (5'−>3')
ac
Read Mapping Location Rates
Rate
0.0
0.2
0.4
0.6
0.8
1.0
Unique Gene
Unique Gene UTR
Ambig Gene
No Gene
No Gene Intronic
No Gene 1kb From Gene
No Gene 10kb From Gene
No Gene Middle Of Nowhere
ad
Observed Splice Junction Loci, by type
Splice Loci
0
20000
40000
60000
80000
All Loci
Known SJ Loci
Novel SJ Loci
Known Total
Novel Total
1−3 Reads
4+ Reads
1−3 Reads
4+ Reads

ae

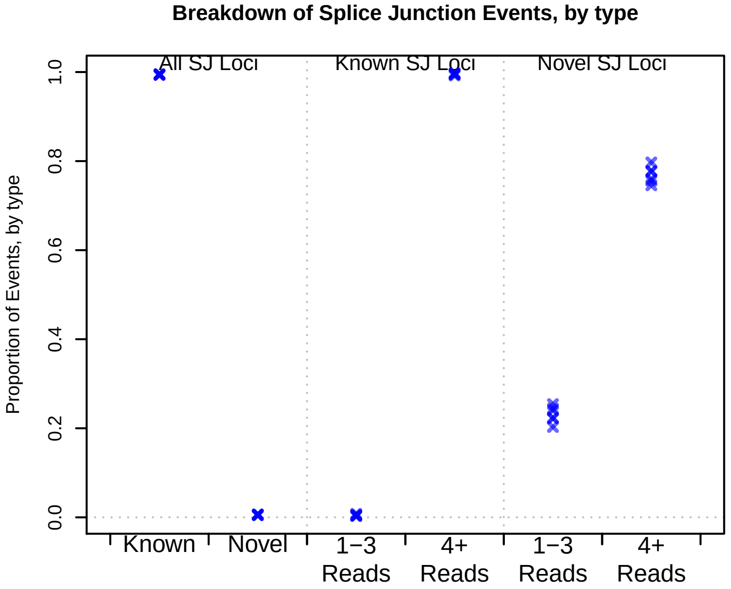

af

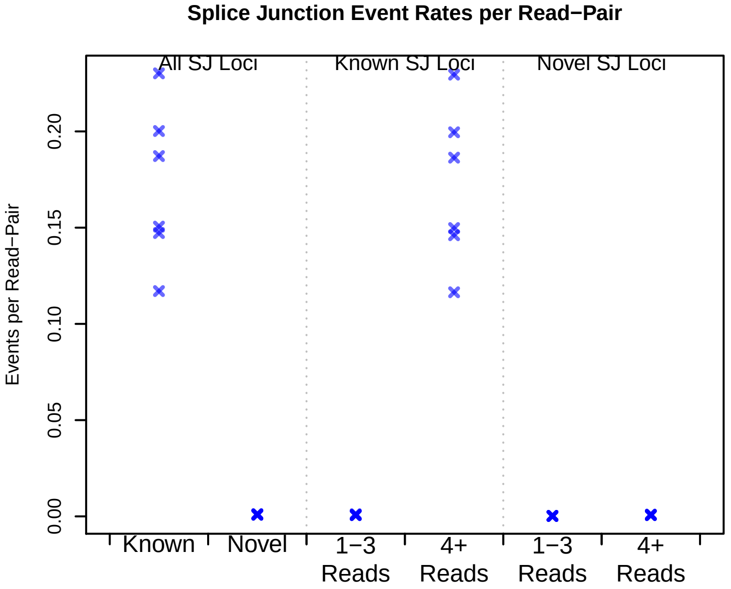

ag

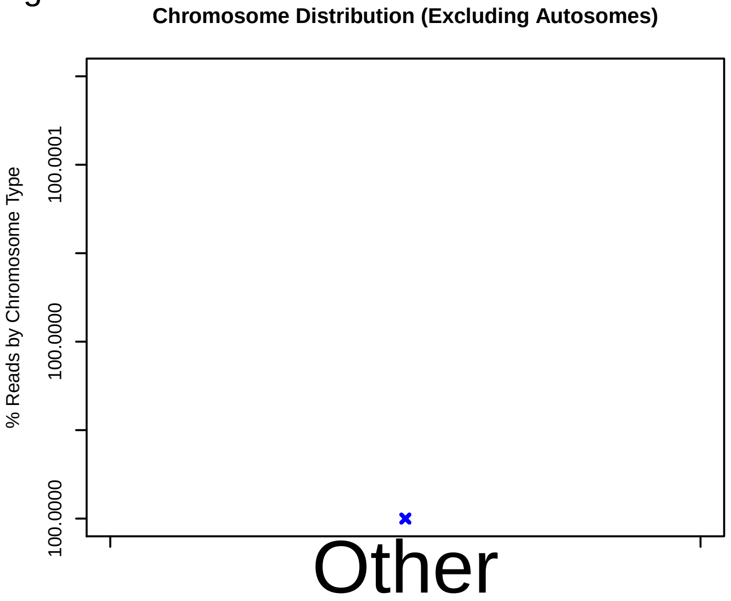

ah

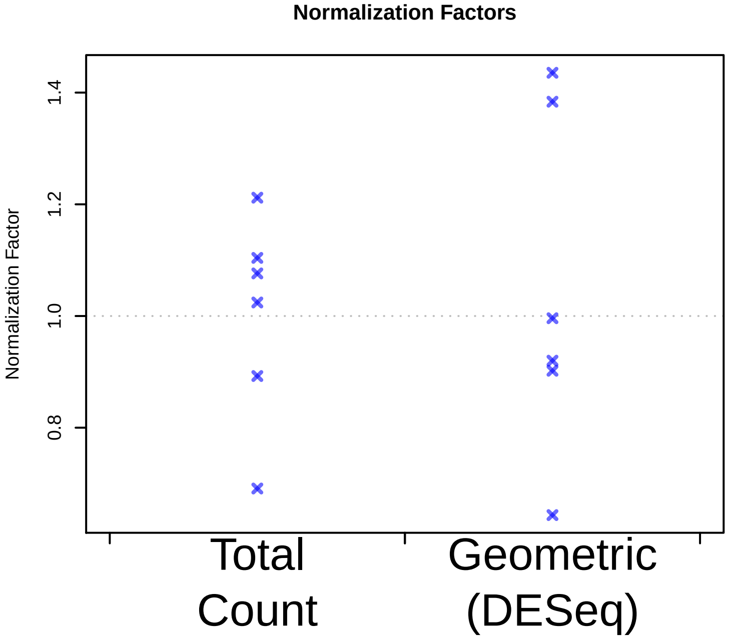

ai

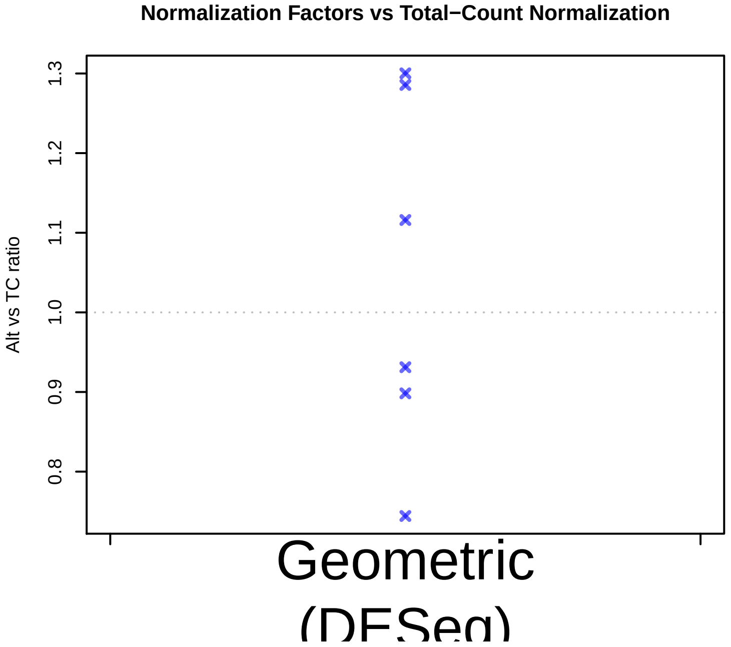

aj

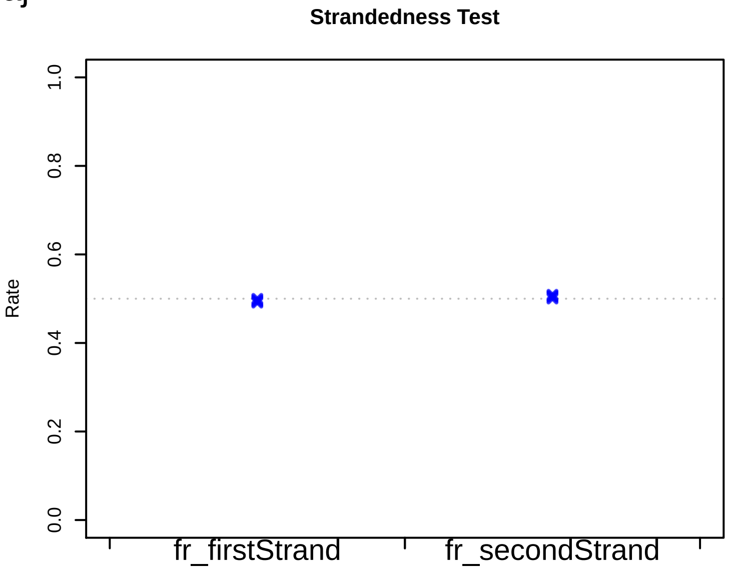

ak

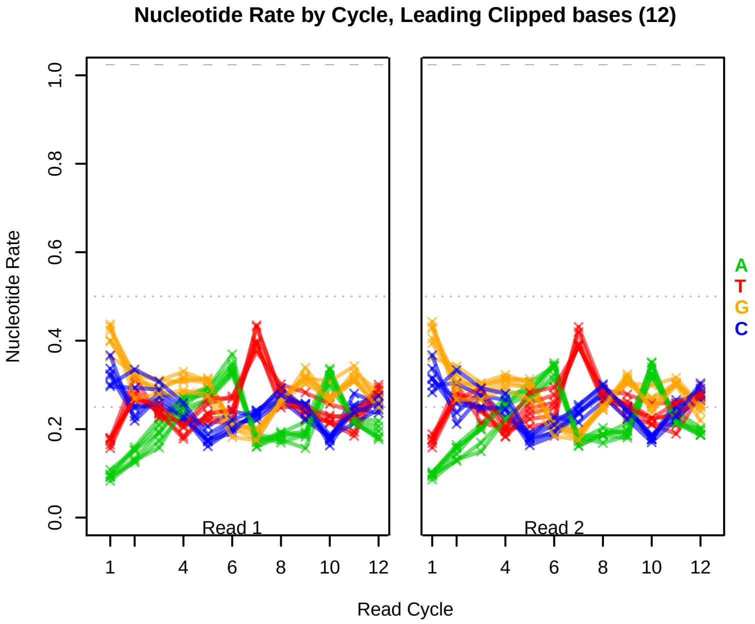

Nucleotide Rate by Cycle, Leading Clipped bases (12)
Nucleotide Rate
0.0
0.2
0.4
0.6
0.8
1.0
Read 1
Read 2
1
4
6
8
10
12
Read Cycle
A
T
G
C

al

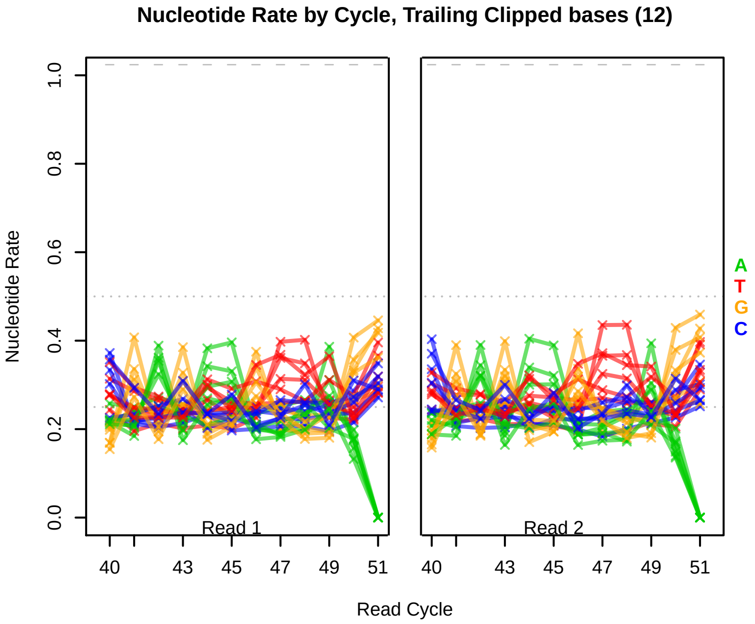

Nucleotide Rate by Cycle, Trailing Clipped bases (12)
Nucleotide Rate
0.0
0.2
0.4
0.6
0.8
1.0
Read 1
Read 2
40
43
45
47
49
51
Read Cycle
A
T
G
C

am

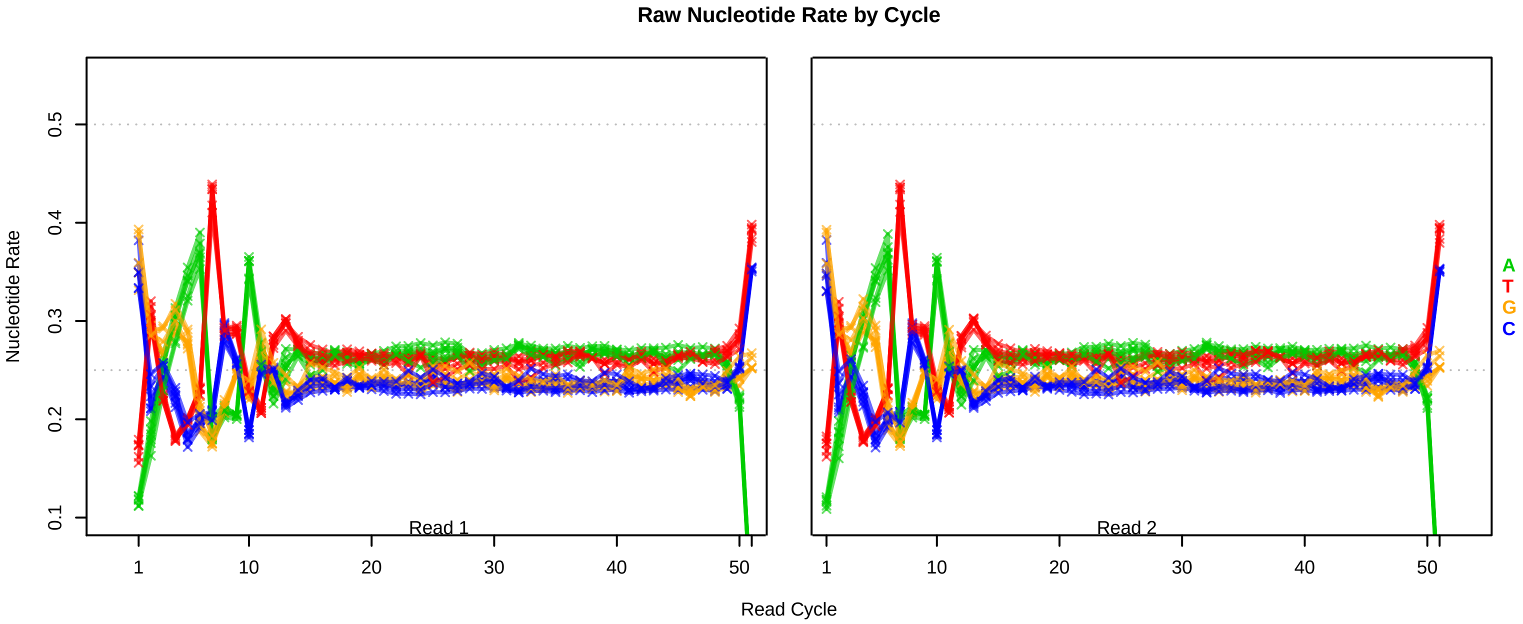

Raw Nucleotide Rate by Cycle
Nucleotide Rate
0.1
0.2
0.3
0.4
0.5
Read 1
Read 2
1
10
20
30
40
50
Read Cycle
A
T
G
C

an

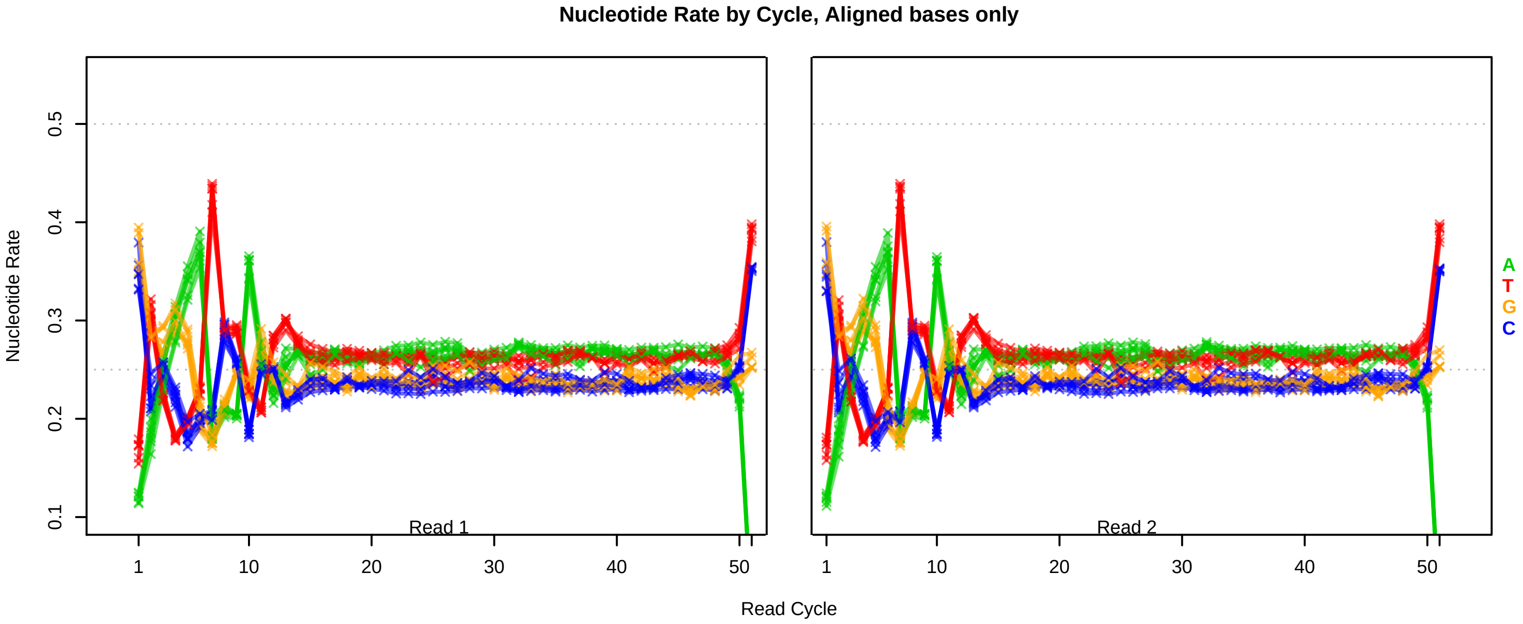

Nucleotide Rate by Cycle, Aligned bases only
Nucleotide Rate
0.1
0.2
0.3
0.4
0.5
Read 1
Read 2
1
10
20
30
40
50
Read Cycle
A
T
G
C
