# Supplementary material for: The pos-1 3′ untranslated region governs germline specification and proliferation to ensure reproductive robustness
Source: PLoS Genet. 2026 Apr 27;22(4):e1012129. doi: 10.1371/journal.pgen.1012129 (PMC13132445; doi:10.1371/journal.pgen.1012129)
Supplement: S1 Data — (GZ) [file pgen.1012129.s007.gz › SupplementalDataSet2/OneStopRNASeq/bam_qc.zip!/bam_qc/QoRTs_MultiPlot/plot-colorByGroup.pdf]

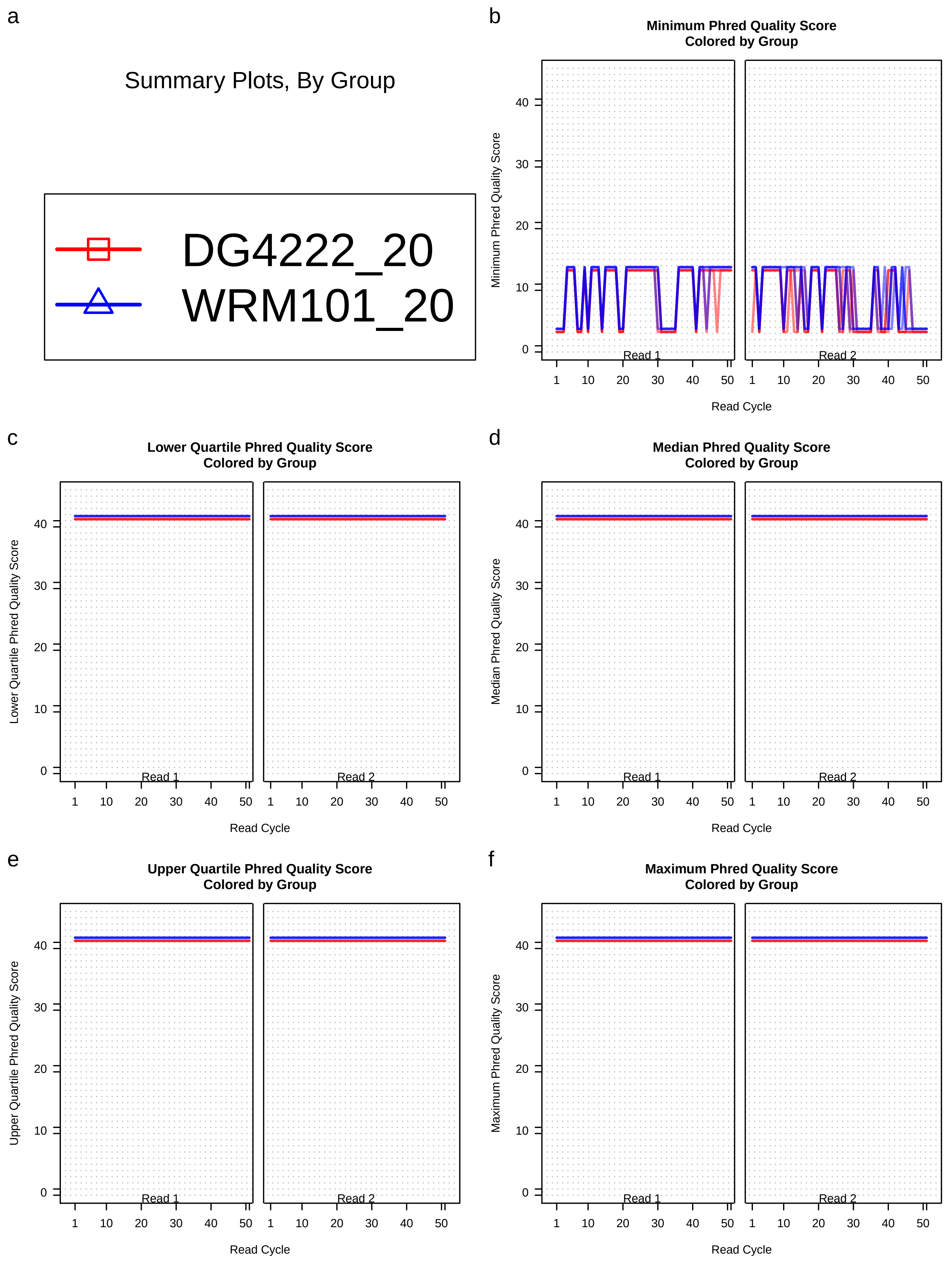
a
Summary Plots, By Group
DG4222_20
WRM101_20
b
Minimum Phred Quality Score
Colored by Group
Minimum Phred Quality Score
Read 1
Read 2
Read Cycle
c
Lower Quartile Phred Quality Score
Colored by Group
Lower Quartile Phred Quality Score
Read 1
Read 2
Read Cycle
d
Median Phred Quality Score
Colored by Group
Median Phred Quality Score
Read 1
Read 2
Read Cycle
e
Upper Quartile Phred Quality Score
Colored by Group
Upper Quartile Phred Quality Score
Read 1
Read 2
Read Cycle
f
Maximum Phred Quality Score
Colored by Group
Maximum Phred Quality Score
Read 1
Read 2
Read Cycle

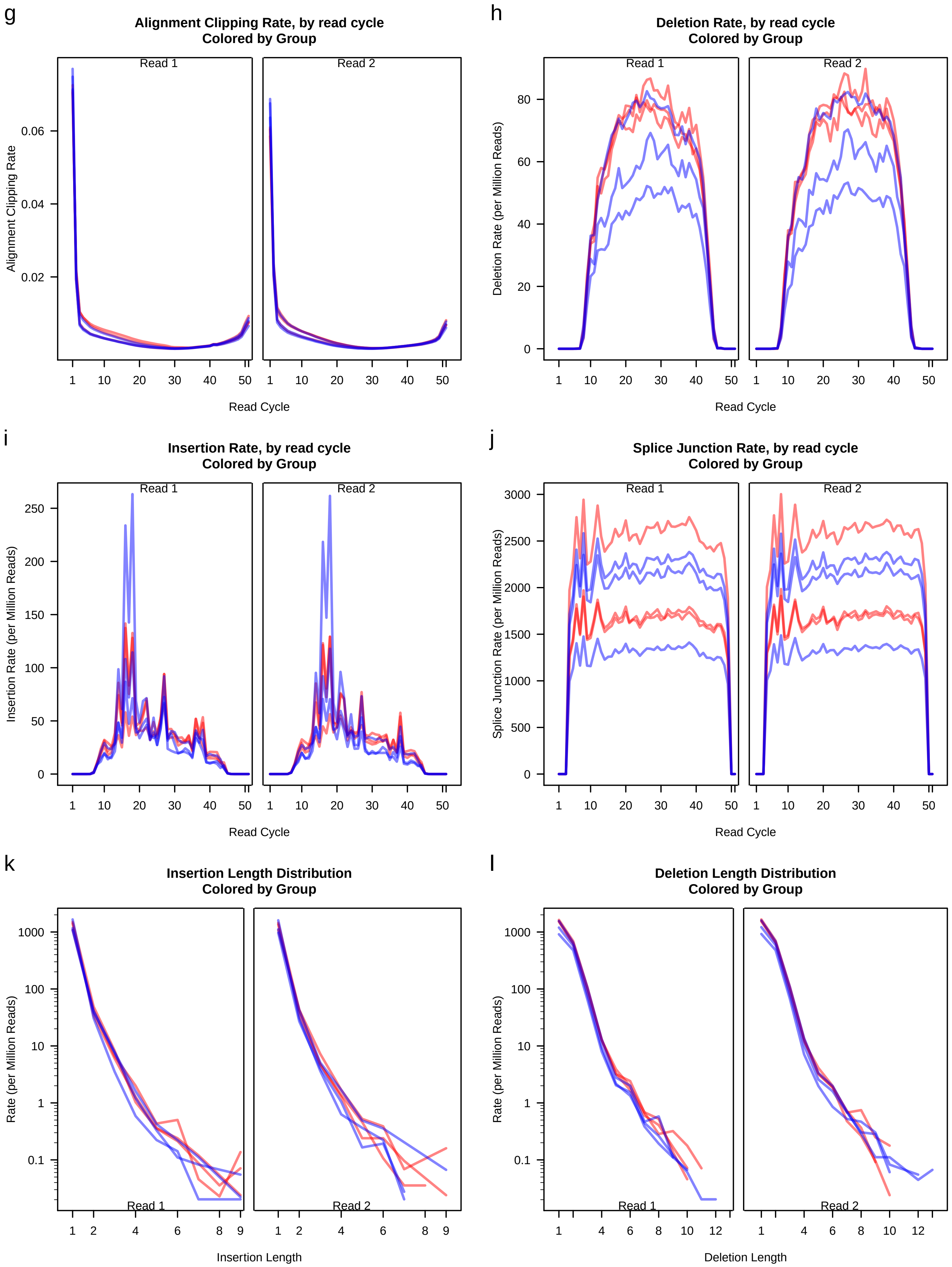
g
Alignment Clipping Rate, by read cycle
Colored by Group
Read 1
Read 2
Alignment Clipping Rate
0.02
0.04
0.06
Read Cycle
h
Deletion Rate, by read cycle
Colored by Group
Read 1
Read 2
Deletion Rate (per Million Reads)
0
20
40
60
80
Read Cycle
i
Insertion Rate, by read cycle
Colored by Group
Read 1
Read 2
Insertion Rate (per Million Reads)
0
50
100
150
200
250
Read Cycle
j
Splice Junction Rate, by read cycle
Colored by Group
Read 1
Read 2
Splice Junction Rate (per Million Reads)
0
500
1000
1500
2000
2500
3000
Read Cycle
1
10
20
30
40
50
k
Insertion Length Distribution
Colored by Group
Read 1
Read 2
Rate (per Million Reads)
0.1
1
10
100
1000
Insertion Length
1
2
4
6
8
9
l
Deletion Length Distribution
Colored by Group
Read 1
Read 2
Rate (per Million Reads)
Deletion Length
1
4
6
8
10
12

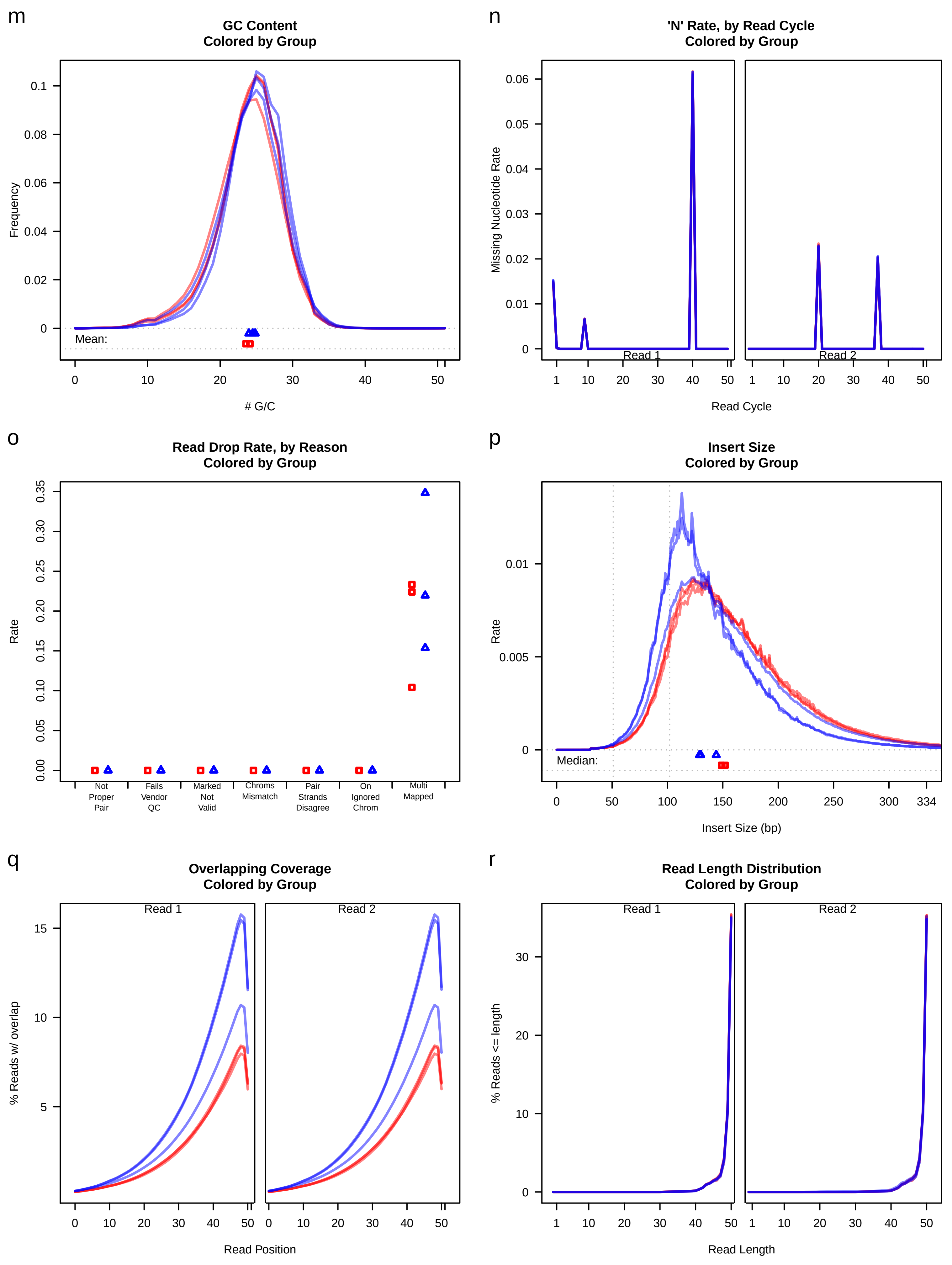
m
GC Content
Colored by Group
Frequency
G/C
Mean:
n
'N' Rate, by Read Cycle
Colored by Group
Missing Nucleotide Rate
Read 1
Read 2
Read Cycle
o
Read Drop Rate, by Reason
Colored by Group
Rate
Not Proper Pair
Fails Vendor QC
Marked Not Valid
Chroms Mismatch
Pair Strands Disagree
On Ignored Chrom
Multi Mapped
p
Insert Size
Colored by Group
Rate
Median:
Insert Size (bp)
q
Overlapping Coverage
Colored by Group
% Reads w/ overlap
Read 1
Read 2
Read Position
r
Read Length Distribution
Colored by Group
% Reads <= length
Read 1
Read 2
Read Length

s

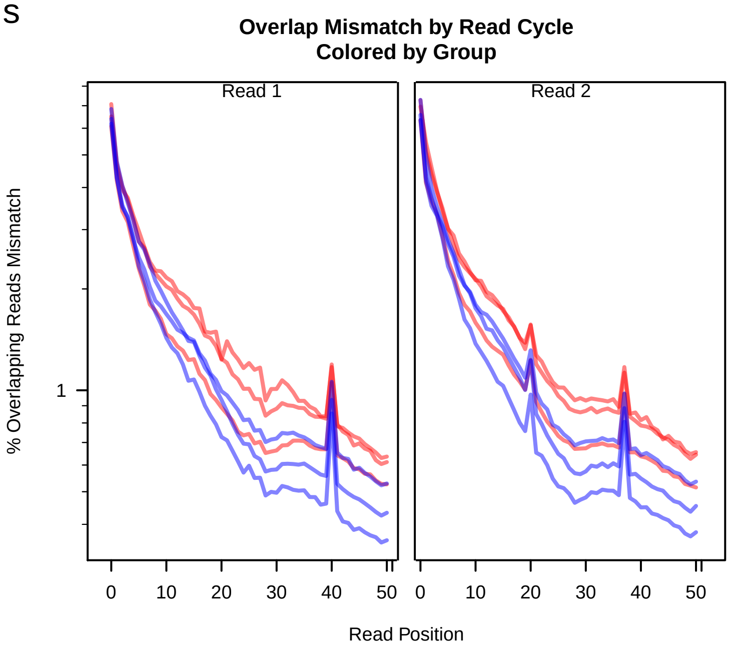

Overlap Mismatch by Read Cycle
Colored by Group
Read 1
Read 2
% Overlapping Reads Mismatch
1
0
10
20
30
40
50
Read Position

t

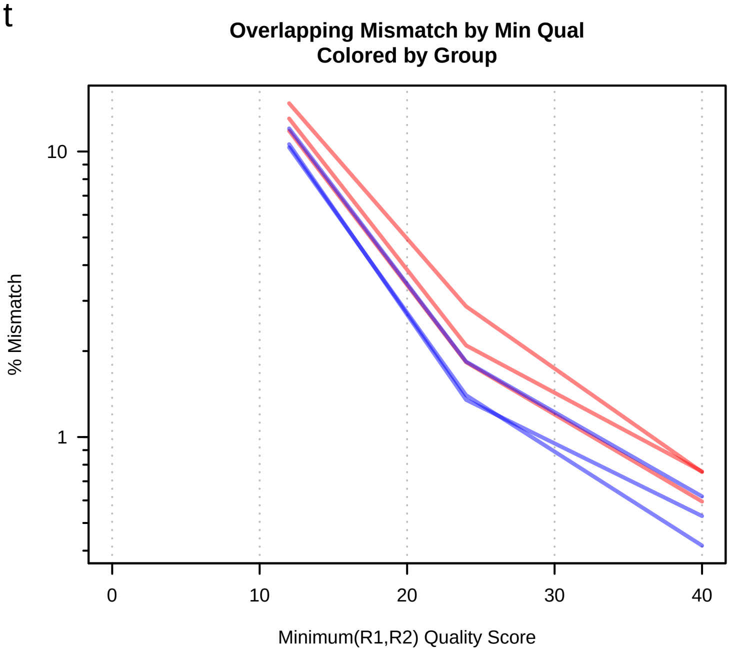

Overlapping Mismatch by Min Qual
Colored by Group
% Mismatch
10
1
0
10
20
30
40
Minimum(R1,R2) Quality Score

u

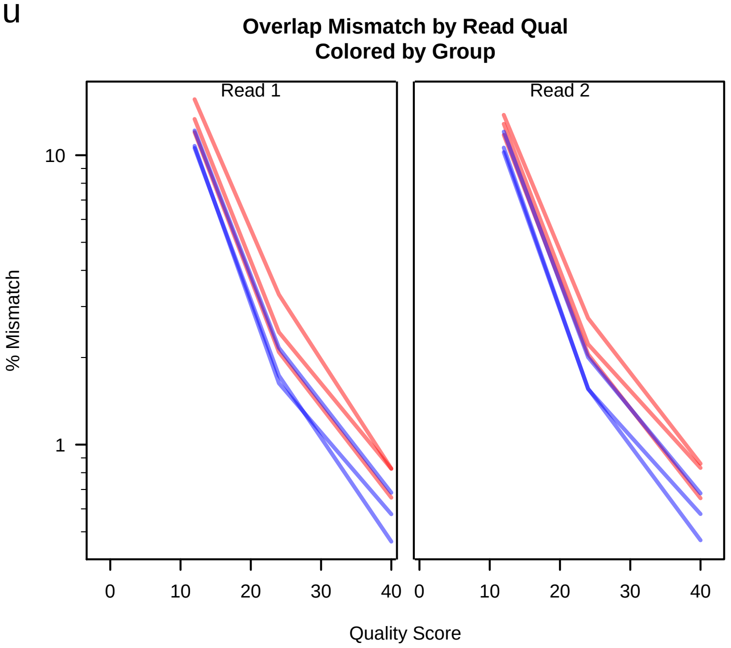

Overlap Mismatch by Read Qual
Colored by Group
Read 1
Read 2
% Mismatch
10
1
0
10
20
30
40
Quality Score

v

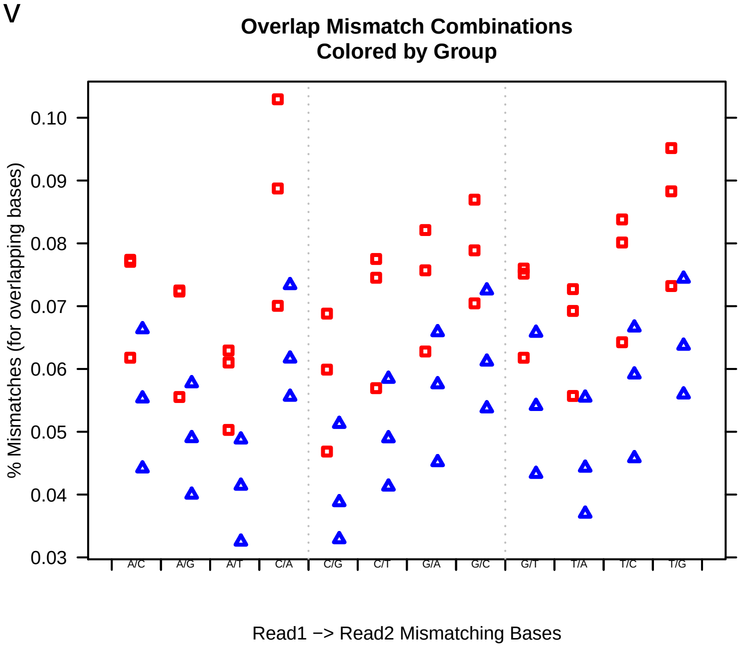

Overlap Mismatch Combinations
Colored by Group
% Mismatches (for overlapping bases)
0.10
0.09
0.08
0.07
0.06
0.05
0.04
0.03
A/C
A/G
A/T
C/A
C/G
C/T
G/A
G/C
G/T
T/A
T/C
T/G
Read1 -> Read2 Mismatching Bases

w

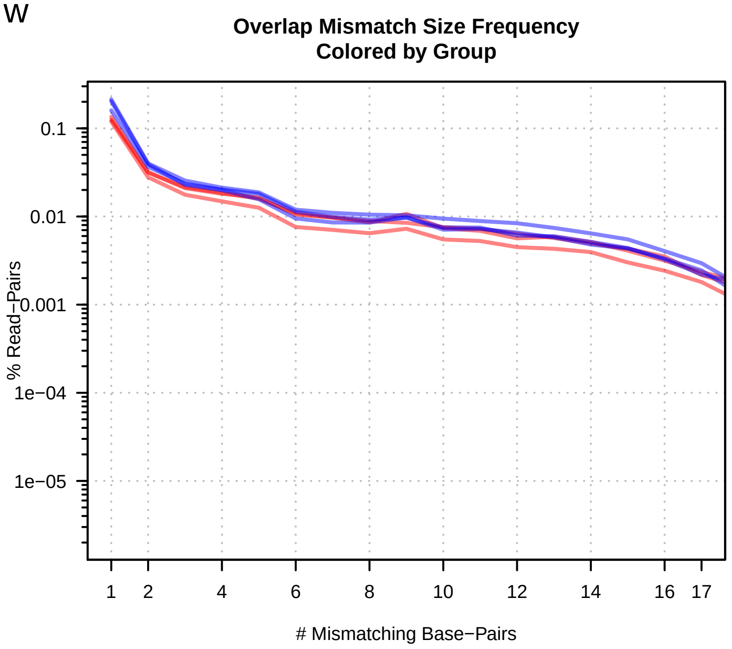

Overlap Mismatch Size Frequency
Colored by Group
% Read−Pairs
0.1
0.01
0.001
1e−04
1e−05
1
2
4
6
8
10
12
14
16
17
Mismatching Base−Pairs

x

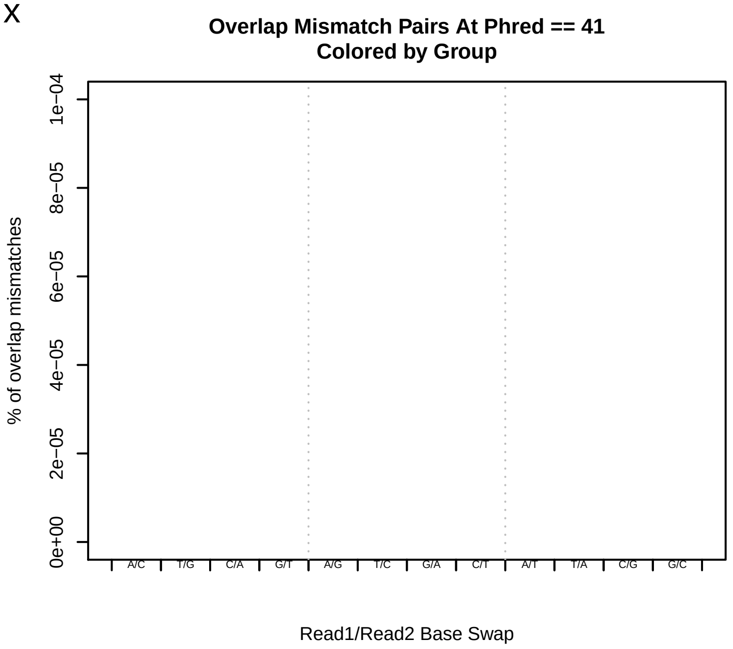

Overlap Mismatch Pairs At Phred == 41
Colored by Group
% of overlap mismatches
1e−04
8e−05
6e−05
4e−05
2e−05
0e+00
A/C
T/G
C/A
G/T
A/G
T/C
G/A
C/T
A/T
T/A
C/G
G/C
Read1/Read2 Base Swap

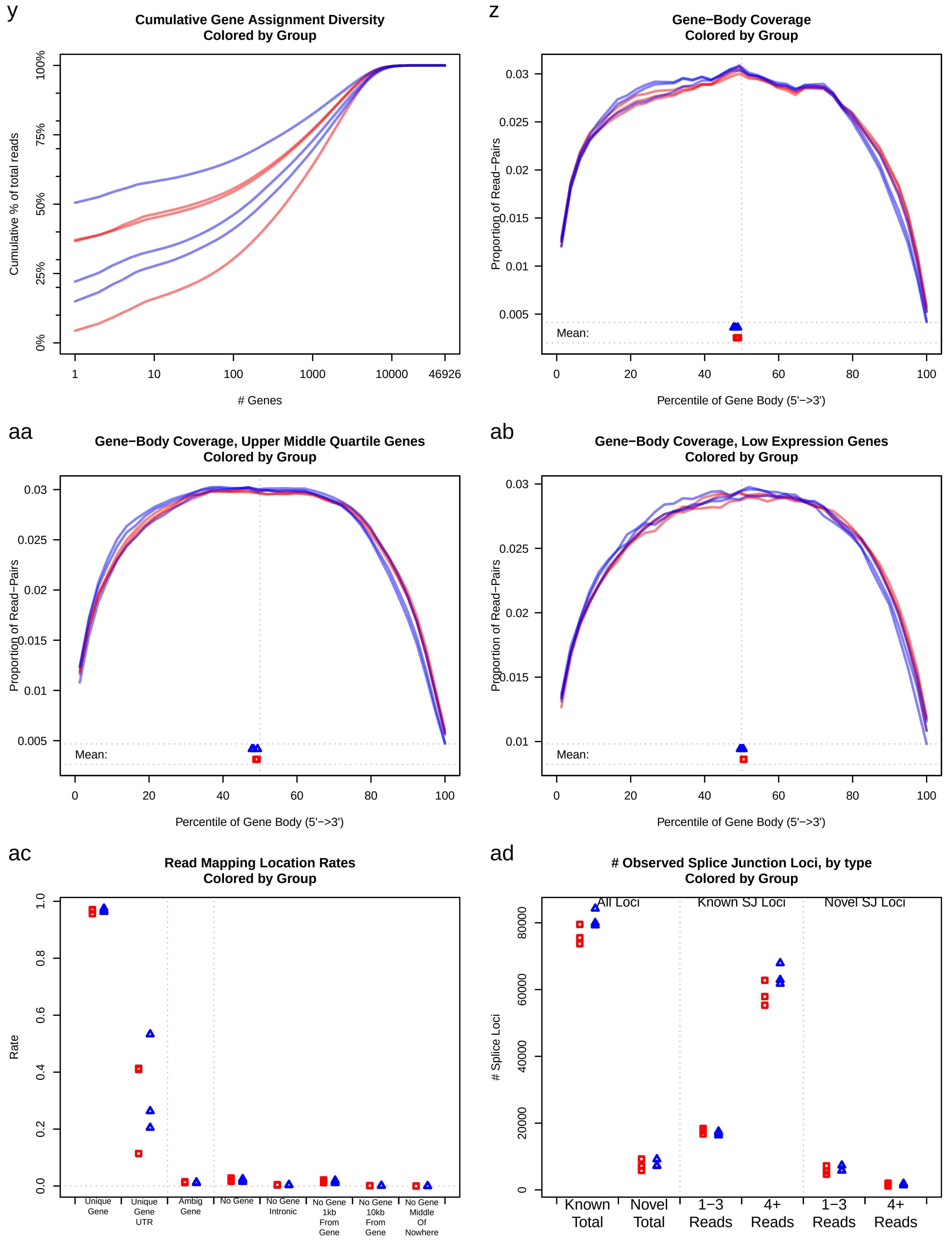
y
Cumulative Gene Assignment Diversity
Colored by Group
Cumulative % of total reads
0%
25%
50%
75%
100%
1
10
100
1000
10000
46926
Genes
z
Gene−Body Coverage
Colored by Group
Proportion of Read−Pairs
0.005
0.01
0.015
0.02
0.025
0.03
Mean:
0
20
40
60
80
100
Percentile of Gene Body (5'−>3')
aa
Gene−Body Coverage, Upper Middle Quartile Genes
Colored by Group
Proportion of Read−Pairs
0.005
0.01
0.015
0.02
0.025
0.03
Mean:
0
20
40
60
80
100
Percentile of Gene Body (5'−>3')
ab
Gene−Body Coverage, Low Expression Genes
Colored by Group
Proportion of Read−Pairs
0.01
0.015
0.02
0.025
0.03
Mean:
0
20
40
60
80
100
Percentile of Gene Body (5'−>3')
ac
Read Mapping Location Rates
Colored by Group
Rate
0.0
0.2
0.4
0.6
0.8
1.0
Unique Gene
Unique Gene UTR
Ambig Gene
No Gene
No Gene Intronic
No Gene 1kb From Gene
No Gene 10kb From Gene
No Gene Middle Of Nowhere
ad
Observed Splice Junction Loci, by type
Colored by Group
Splice Loci
0
20000
40000
60000
80000
All Loci
Known SJ Loci
Novel SJ Loci
Known Total
Novel Total
1−3 Reads
4+ Reads
1−3 Reads
4+ Reads

ae

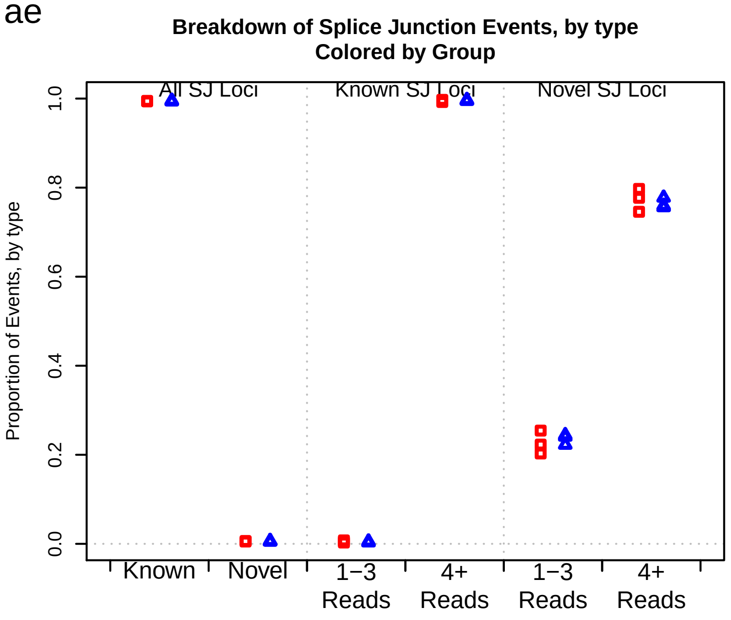

af

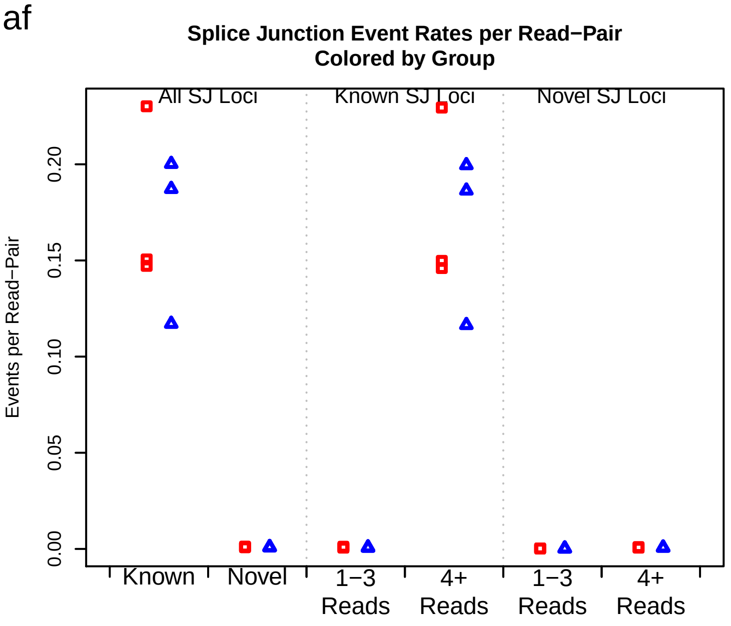

ag

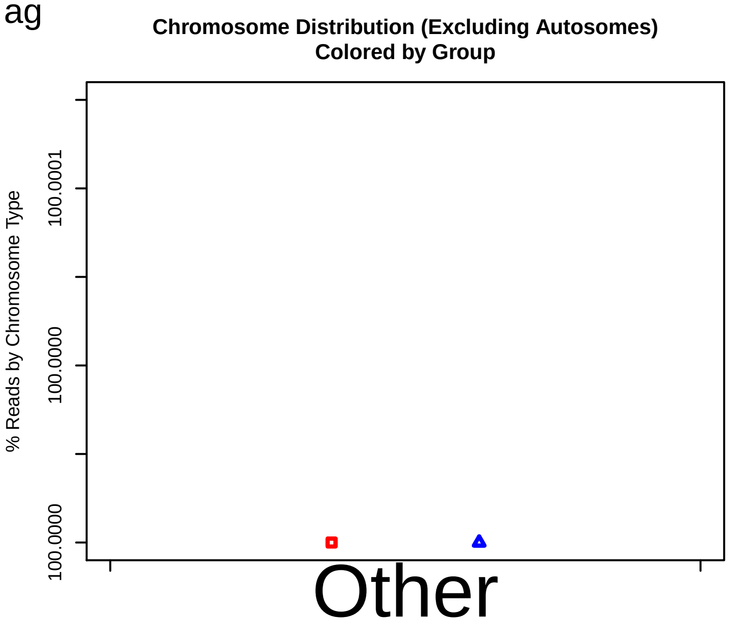

ah

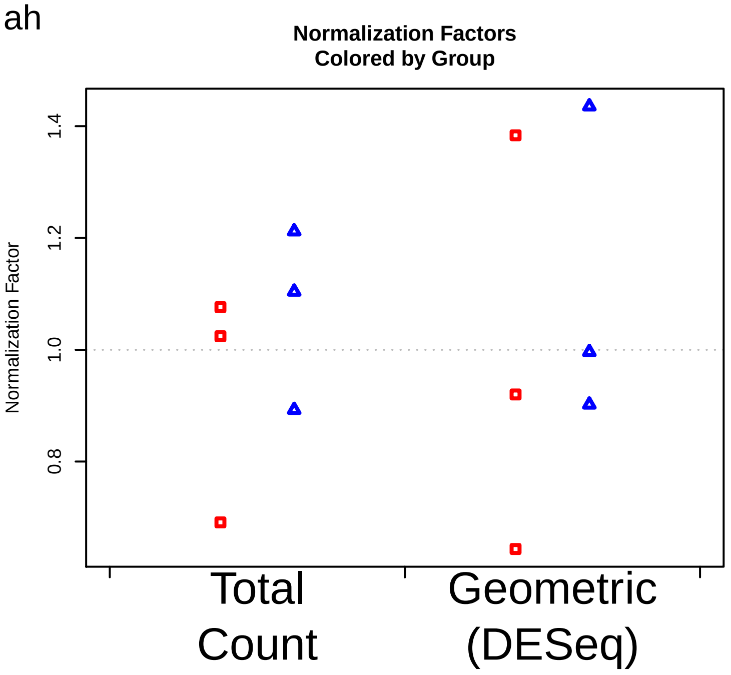

ai

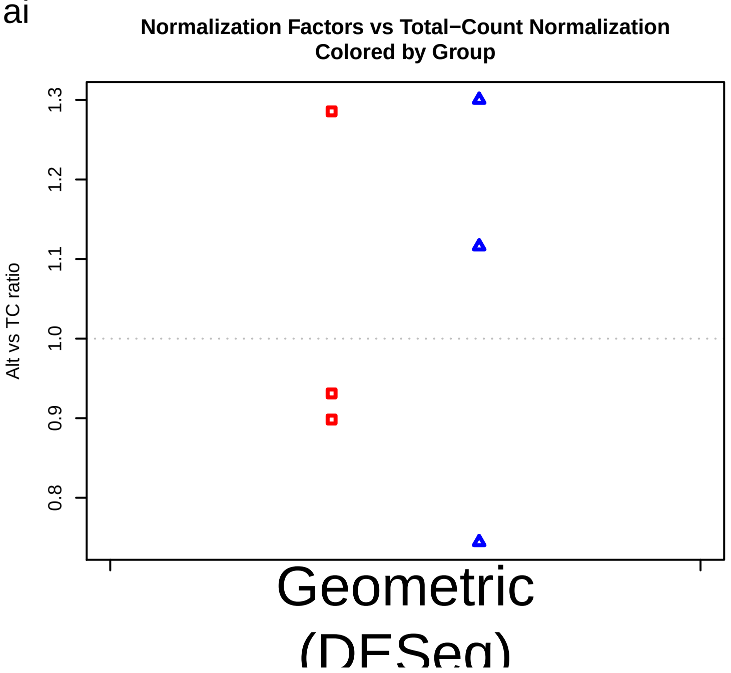

aj

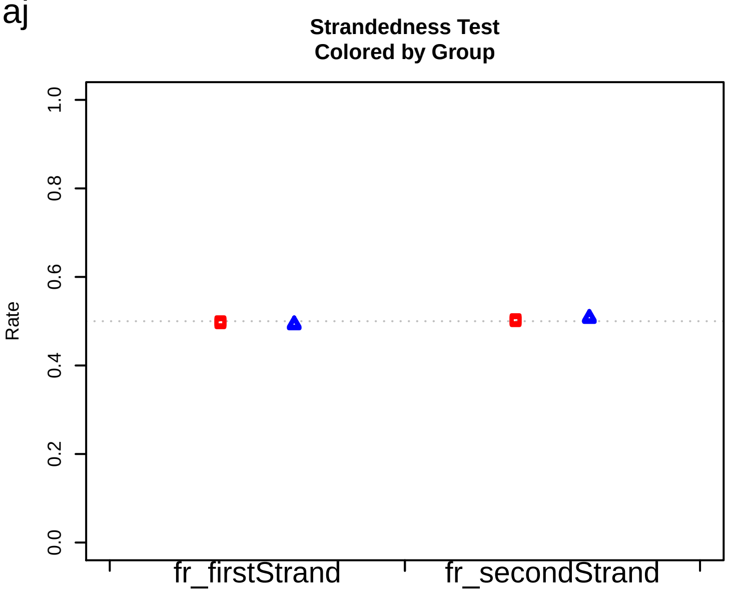

ak

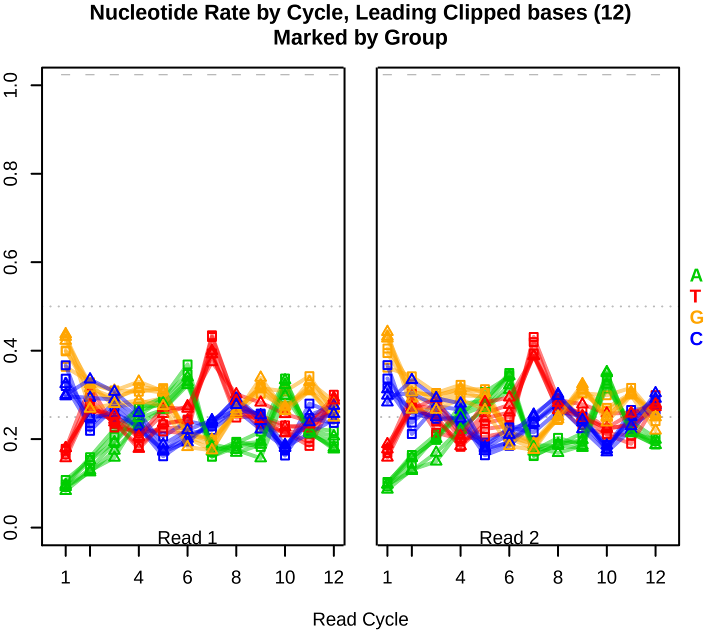
Nucleotide Rate by Cycle, Leading Clipped bases (12)
Marked by Group
Nucleotide Rate
0.0
0.2
0.4
0.6
0.8
1.0
Read 1
Read 2
1
4
6
8
10
12
Read Cycle
A
T
G
C

al

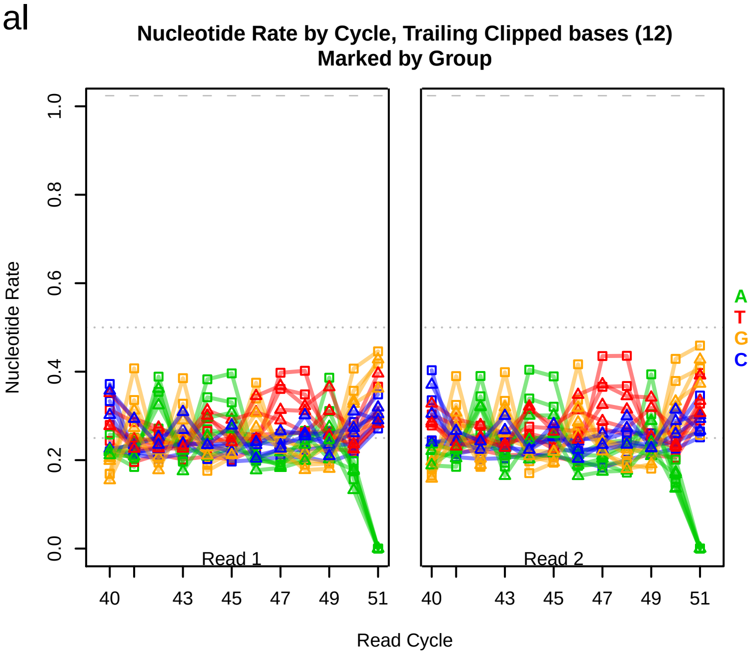
Nucleotide Rate by Cycle, Trailing Clipped bases (12)
Marked by Group
Nucleotide Rate
0.0
0.2
0.4
0.6
0.8
1.0
Read 1
Read 2
40
43
45
47
49
51
Read Cycle
A
T
G
C

am

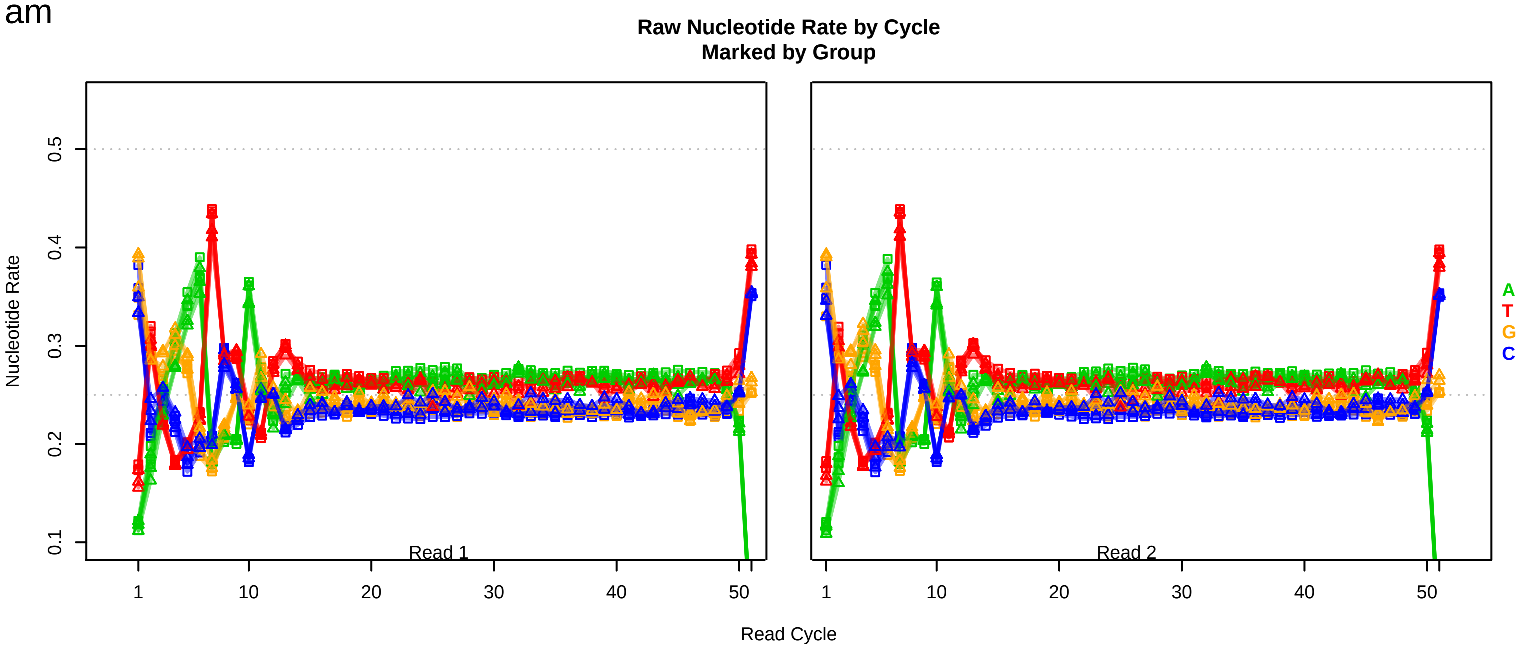
Raw Nucleotide Rate by Cycle
Marked by Group
Nucleotide Rate
0.1
0.2
0.3
0.4
0.5
Read 1
Read 2
1
10
20
30
40
50
Read Cycle
A
T
G
C

an

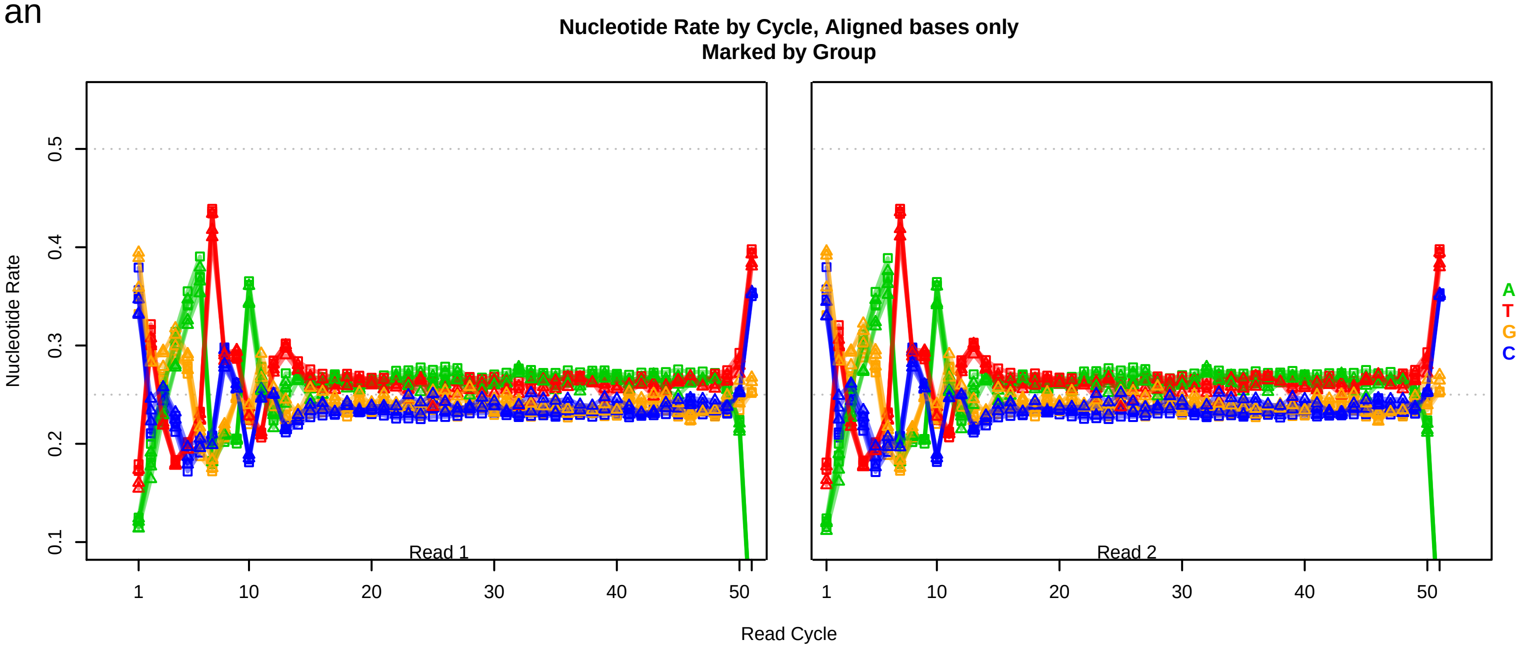
Nucleotide Rate by Cycle, Aligned bases only
Marked by Group
Nucleotide Rate
0.1
0.2
0.3
0.4
0.5
Read 1
Read 2
1
10
20
30
40
50
Read Cycle
A
T
G
C
