# Supplementary material for: The pos-1 3′ untranslated region governs germline specification and proliferation to ensure reproductive robustness
Source: PLoS Genet. 2026 Apr 27;22(4):e1012129. doi: 10.1371/journal.pgen.1012129 (PMC13132445; doi:10.1371/journal.pgen.1012129)

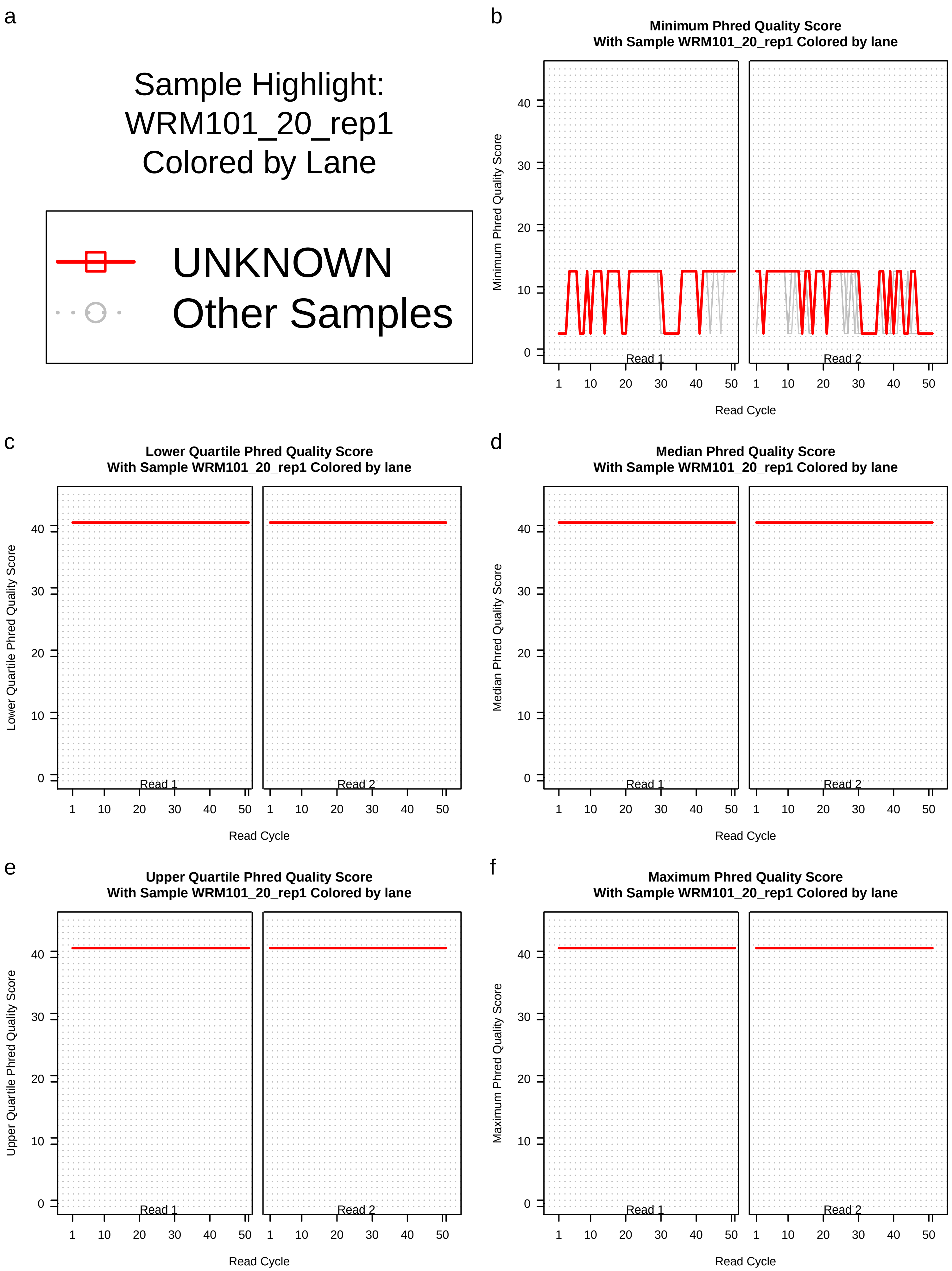
a
Sample Highlight:
WRM101_20_rep1
Colored by Lane
UNKNOWN
Other Samples
b
Minimum Phred Quality Score
With Sample WRM101_20_rep1 Colored by lane
Minimum Phred Quality Score
Read 1
Read 2
Read Cycle
c
Lower Quartile Phred Quality Score
With Sample WRM101_20_rep1 Colored by lane
Lower Quartile Phred Quality Score
Read 1
Read 2
Read Cycle
d
Median Phred Quality Score
With Sample WRM101_20_rep1 Colored by lane
Median Phred Quality Score
Read 1
Read 2
Read Cycle
e
Upper Quartile Phred Quality Score
With Sample WRM101_20_rep1 Colored by lane
Upper Quartile Phred Quality Score
Read 1
Read 2
Read Cycle
f
Maximum Phred Quality Score
With Sample WRM101_20_rep1 Colored by lane
Maximum Phred Quality Score
Read 1
Read 2
Read Cycle

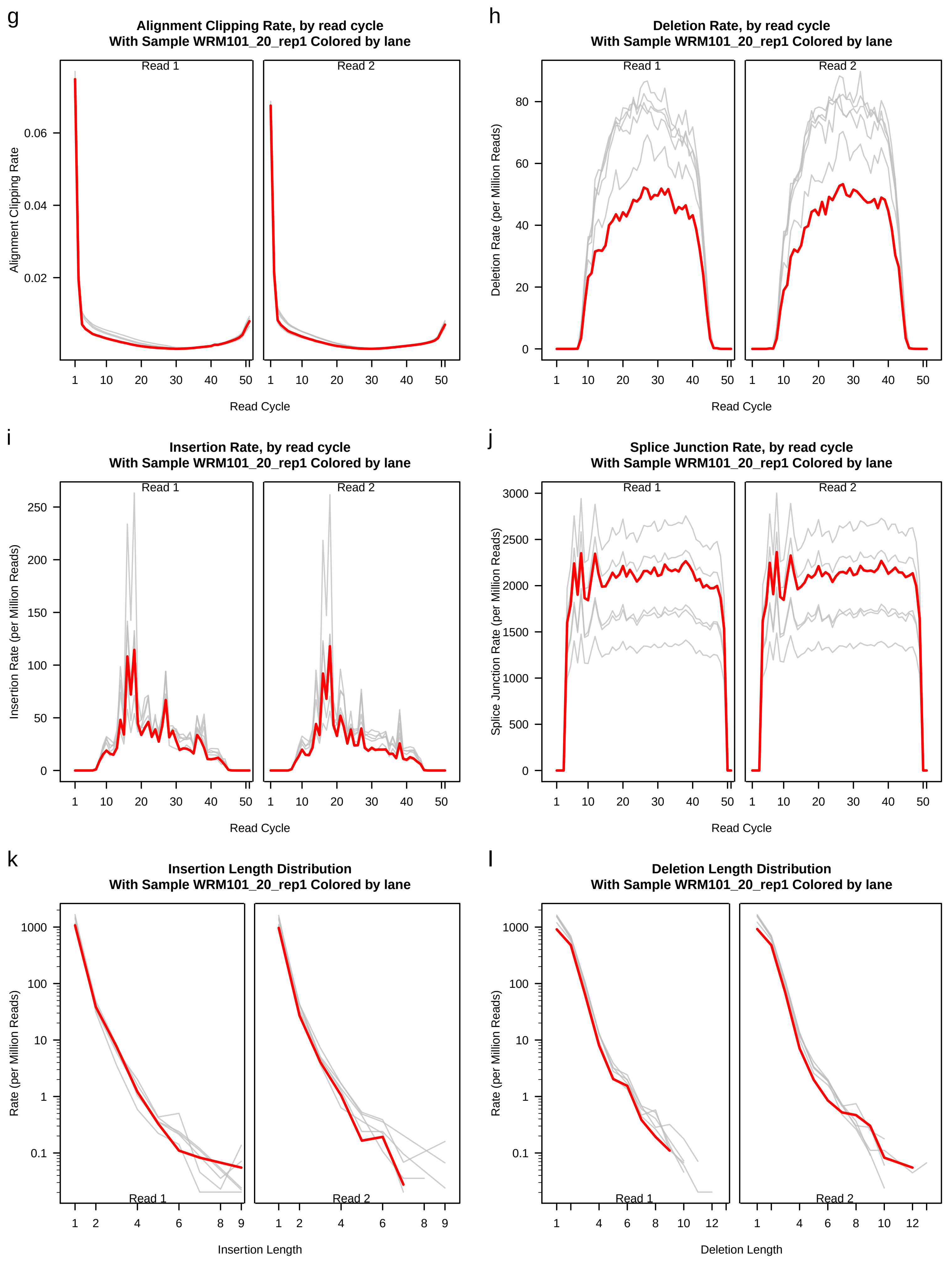
g
Alignment Clipping Rate, by read cycle
With Sample WRM101_20_rep1 Colored by lane
Read 1
Read 2
Alignment Clipping Rate
0.02
0.04
0.06
1 10 20 30 40 50
Read Cycle
h
Deletion Rate, by read cycle
With Sample WRM101_20_rep1 Colored by lane
Read 1
Read 2
Deletion Rate (per Million Reads)
0
20
40
60
80
1 10 20 30 40 50
Read Cycle
i
Insertion Rate, by read cycle
With Sample WRM101_20_rep1 Colored by lane
Read 1
Read 2
Insertion Rate (per Million Reads)
0
50
100
150
200
250
1 10 20 30 40 50
Read Cycle
j
Splice Junction Rate, by read cycle
With Sample WRM101_20_rep1 Colored by lane
Read 1
Read 2
Splice Junction Rate (per Million Reads)
0
500
1000
1500
2000
2500
3000
1 10 20 30 40 50
Read Cycle
k
Insertion Length Distribution
With Sample WRM101_20_rep1 Colored by lane
Read 1
Read 2
Rate (per Million Reads)
0.1
1
10
100
1000
1 2 4 6 8 9
Insertion Length
l
Deletion Length Distribution
With Sample WRM101_20_rep1 Colored by lane
Read 1
Read 2
Rate (per Million Reads)
0.1
1
10
100
1000
1 4 6 8 10 12
Deletion Length

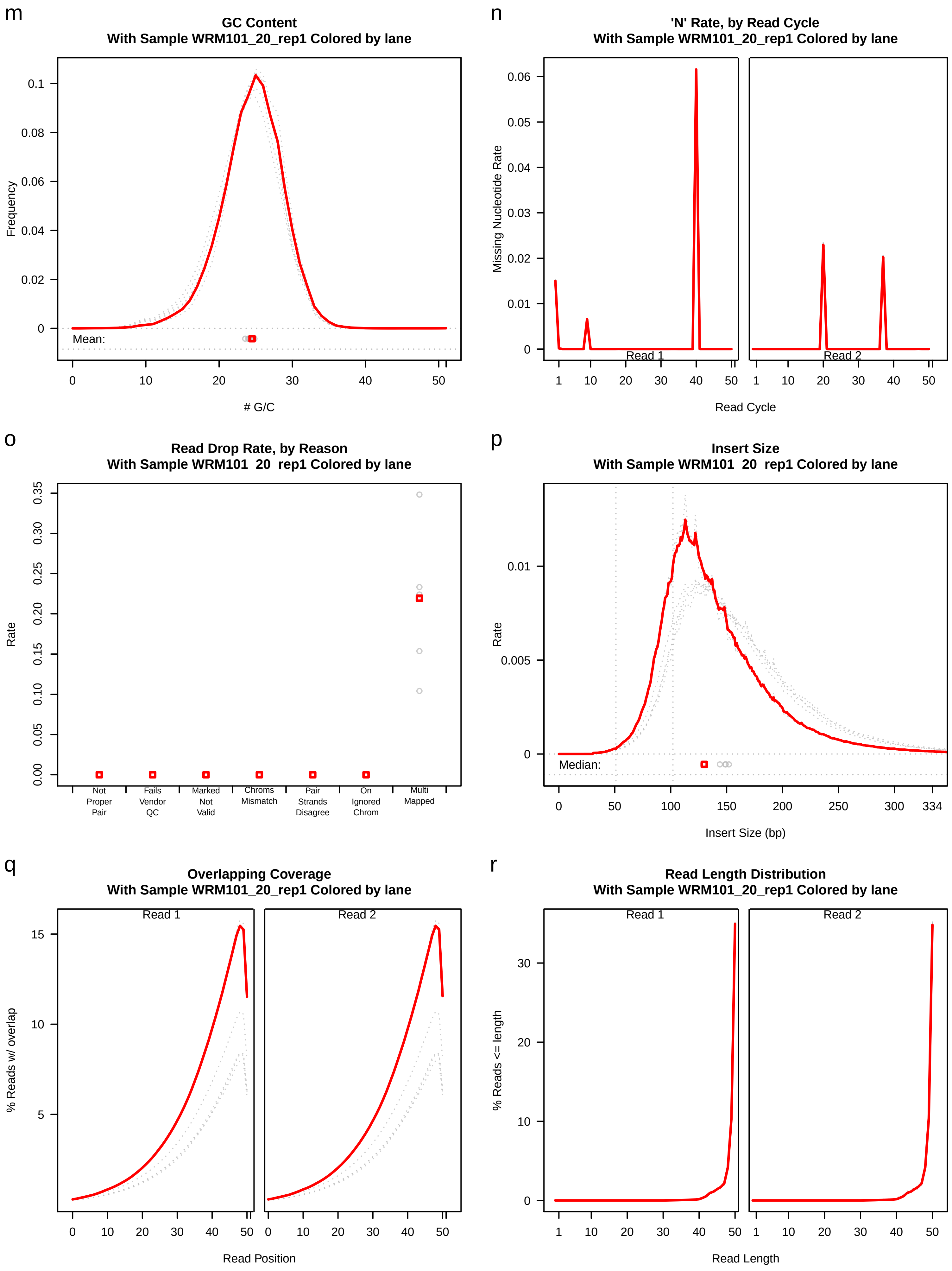
m
GC Content
With Sample WRM101_20_rep1 Colored by lane
Frequency
0.1
0.08
0.06
0.04
0.02
0
Mean:
0 10 20 30 40 50
G/C
n
'N' Rate, by Read Cycle
With Sample WRM101_20_rep1 Colored by lane
Missing Nucleotide Rate
0.06
0.05
0.04
0.03
0.02
0.01
0
Read 1
Read 2
1 10 20 30 40 50 1 10 20 30 40 50
Read Cycle
o
Read Drop Rate, by Reason
With Sample WRM101_20_rep1 Colored by lane
Rate
0.00 0.05 0.10 0.15 0.20 0.25 0.30 0.35
Not Proper Pair
Fails Vendor QC
Marked Not Valid
Chroms Mismatch
Pair Strands Disagree
On Ignored Chrom
Multi Mapped
p
Insert Size
With Sample WRM101_20_rep1 Colored by lane
Rate
0.01
0.005
0
Median:
0 50 100 150 200 250 300 334
Insert Size (bp)
q
Overlapping Coverage
With Sample WRM101_20_rep1 Colored by lane
% Reads w/ overlap
15
10
5
Read 1
Read 2
0 10 20 30 40 50 0 10 20 30 40 50
Read Position
r
Read Length Distribution
With Sample WRM101_20_rep1 Colored by lane
% Reads <= length
30
20
10
0
Read 1
Read 2
1 10 20 30 40 50 1 10 20 30 40 50
Read Length

s

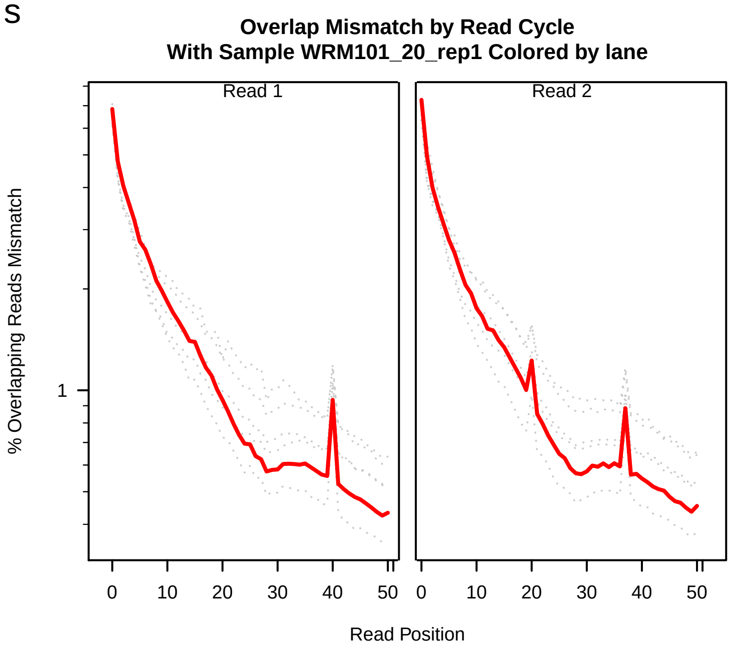
Overlap Mismatch by Read Cycle
With Sample WRM101_20_rep1 Colored by lane
Read 1
Read 2
% Overlapping Reads Mismatch
1
0
10
20
30
40
50
Read Position

t

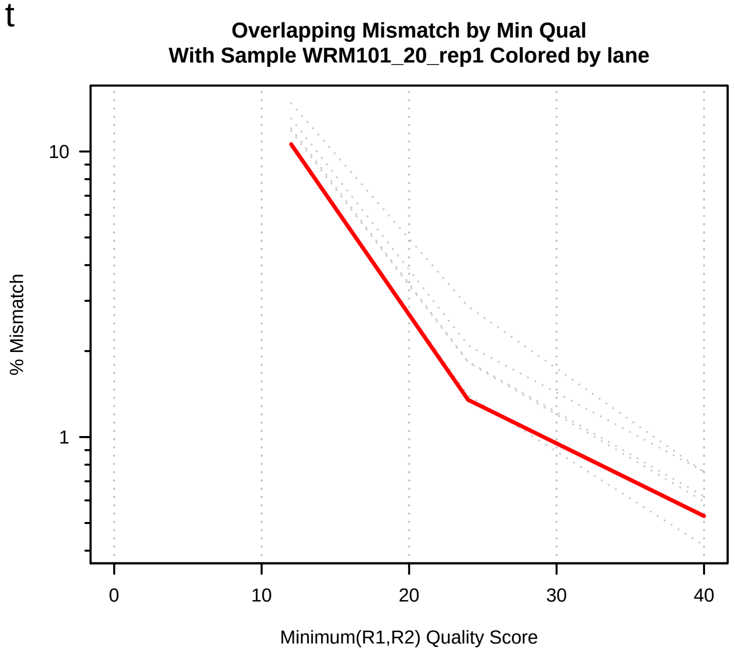
Overlapping Mismatch by Min Qual
With Sample WRM101_20_rep1 Colored by lane
% Mismatch
10
1
0
10
20
30
40
Minimum(R1,R2) Quality Score

u

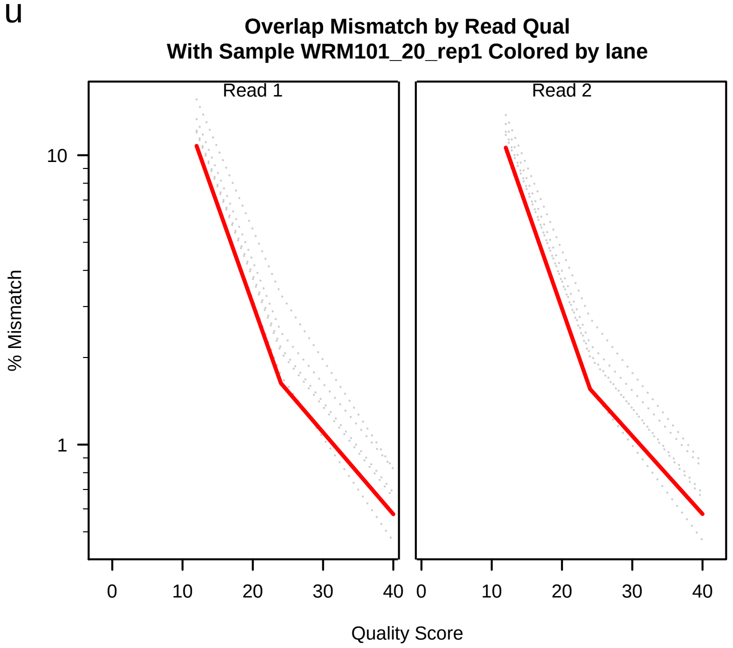
Overlap Mismatch by Read Qual
With Sample WRM101_20_rep1 Colored by lane
Read 1
Read 2
% Mismatch
10
1
0
10
20
30
40
Quality Score

v

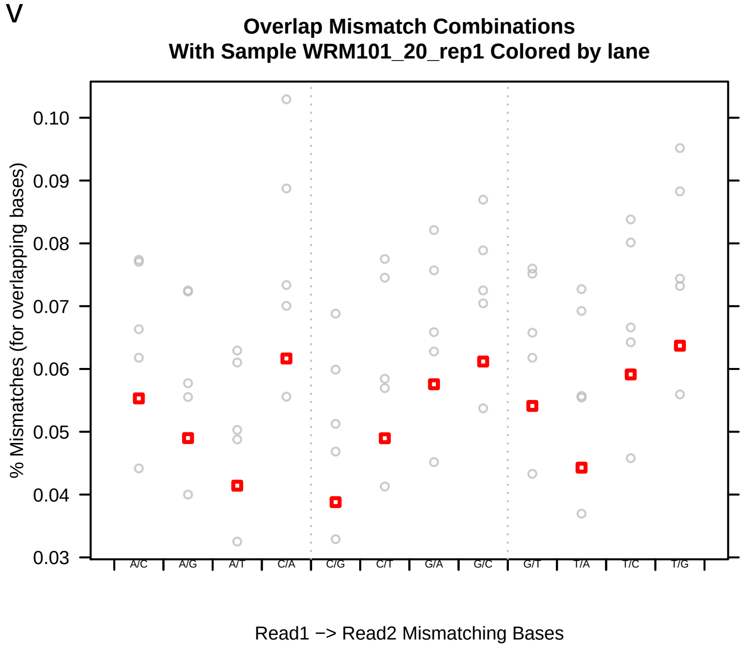
Overlap Mismatch Combinations
With Sample WRM101_20_rep1 Colored by lane
% Mismatches (for overlapping bases)
0.10
0.09
0.08
0.07
0.06
0.05
0.04
0.03
A/C
A/G
A/T
C/A
C/G
C/T
G/A
G/C
G/T
T/A
T/C
T/G
Read1 −> Read2 Mismatching Bases

w

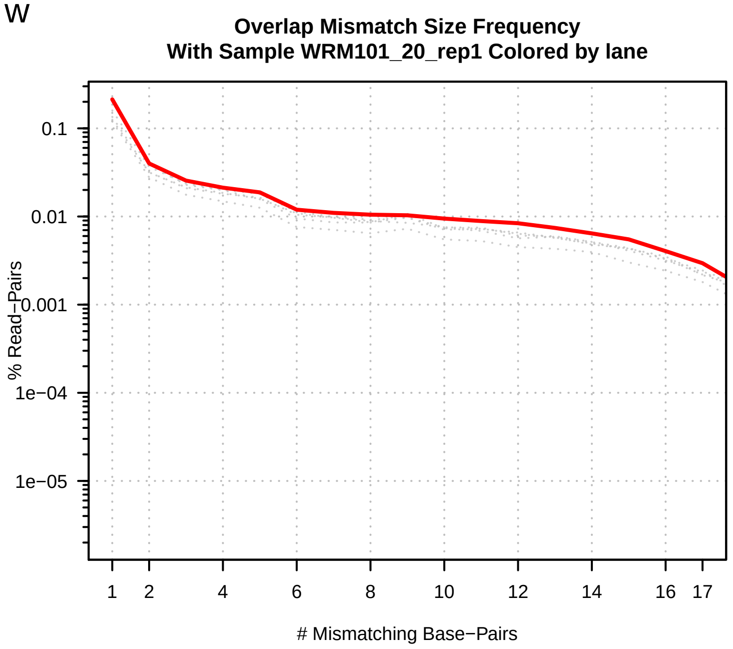
Overlap Mismatch Size Frequency
With Sample WRM101_20_rep1 Colored by lane
% Read−Pairs
0.1
0.01
0.001
1e−04
1e−05
1
2
4
6
8
10
12
14
16
17
Mismatching Base−Pairs

x

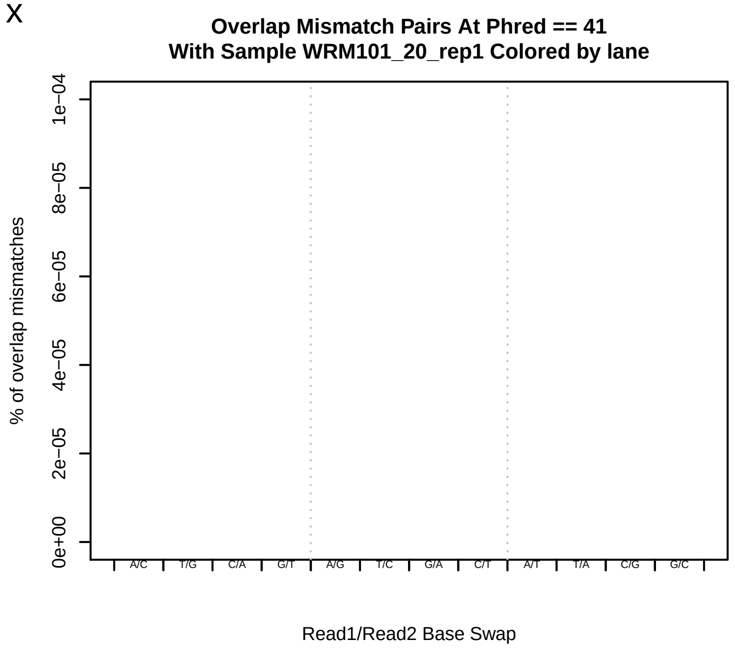
Overlap Mismatch Pairs At Phred == 41
With Sample WRM101_20_rep1 Colored by lane
% of overlap mismatches
1e−04
8e−05
6e−05
4e−05
2e−05
0e+00
A/C
T/G
C/A
G/T
A/G
T/C
G/A
C/T
A/T
T/A
C/G
G/C
Read1/Read2 Base Swap

y

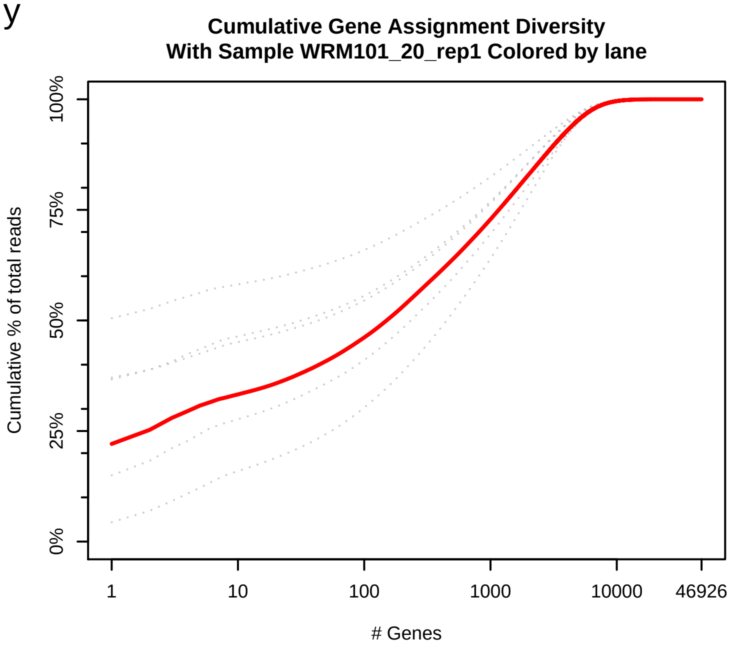
Cumulative Gene Assignment Diversity
With Sample WRM101_20_rep1 Colored by lane
Cumulative % of total reads
0%
25%
50%
75%
100%
1
10
100
1000
10000
46926
Genes

z

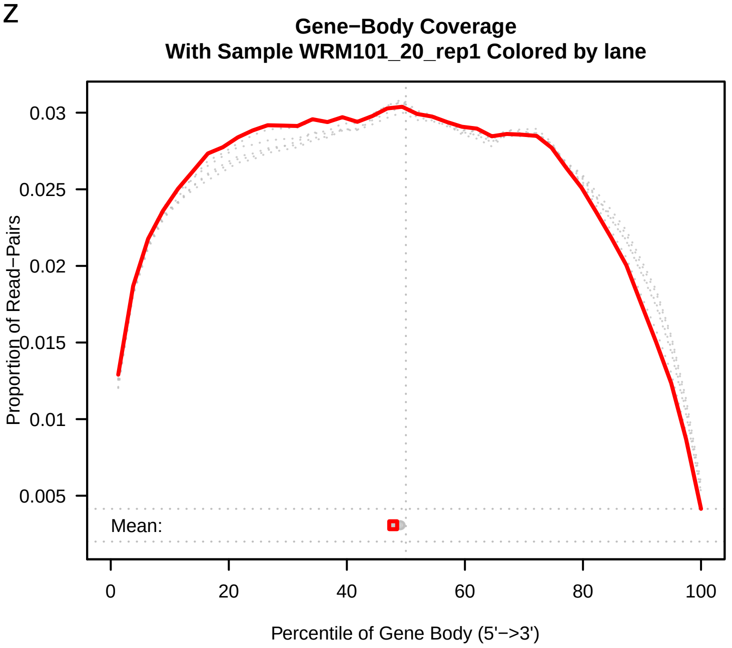
Gene−Body Coverage
With Sample WRM101_20_rep1 Colored by lane
Proportion of Read−Pairs
0.005
0.01
0.015
0.02
0.025
0.03
Mean:
0
20
40
60
80
100
Percentile of Gene Body (5'−>3')

aa

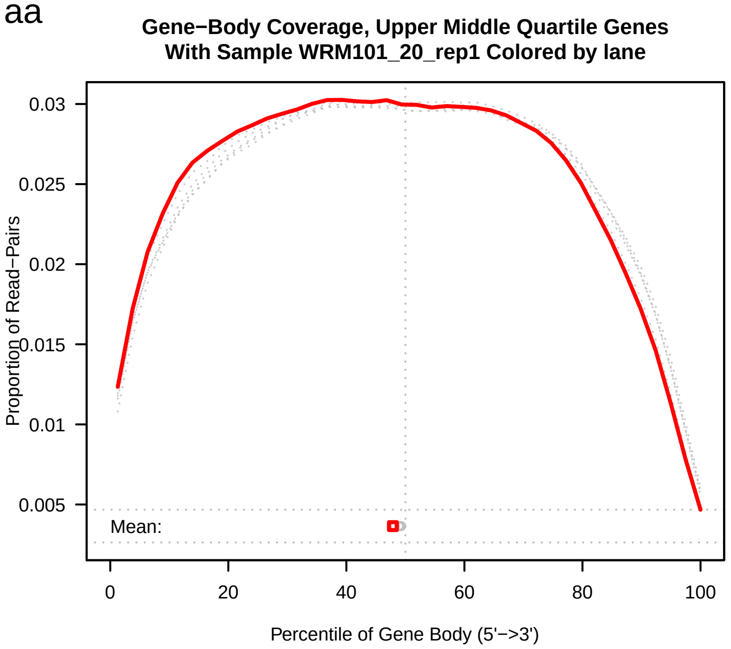
Gene−Body Coverage, Upper Middle Quartile Genes
With Sample WRM101_20_rep1 Colored by lane
Proportion of Read−Pairs
0.005
0.01
0.015
0.02
0.025
0.03
Mean:
0
20
40
60
80
100
Percentile of Gene Body (5'−>3')

ab

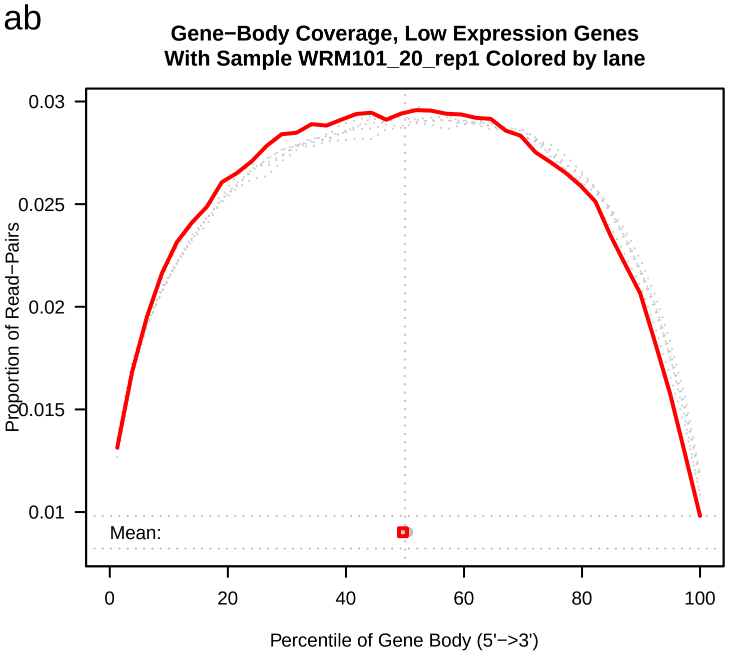
Gene−Body Coverage, Low Expression Genes
With Sample WRM101_20_rep1 Colored by lane
Proportion of Read−Pairs
0.01
0.015
0.02
0.025
0.03
Mean:
0
20
40
60
80
100
Percentile of Gene Body (5'−>3')

ac

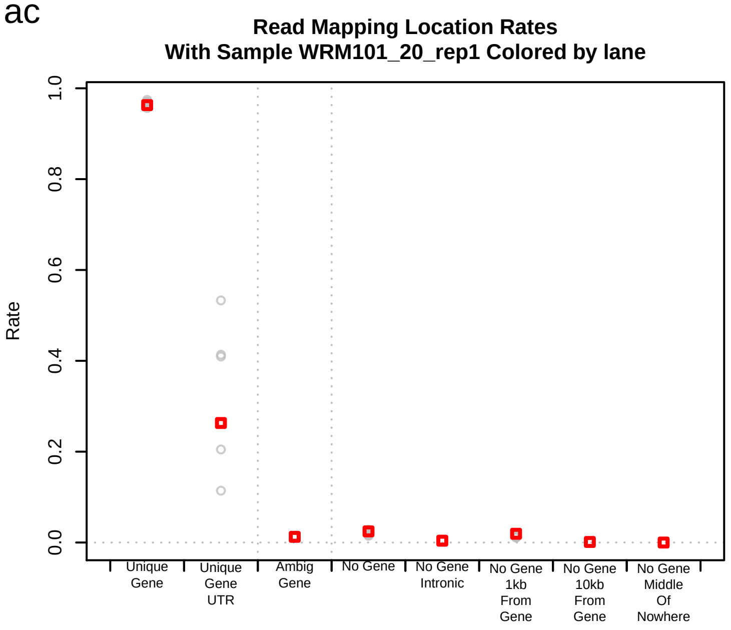
Read Mapping Location Rates
With Sample WRM101_20_rep1 Colored by lane
Rate
0.0
0.2
0.4
0.6
0.8
1.0
Unique Gene
Unique Gene UTR
Ambig Gene
No Gene
No Gene Intronic
No Gene 1kb From Gene
No Gene 10kb From Gene
No Gene Middle Of Nowhere

ad

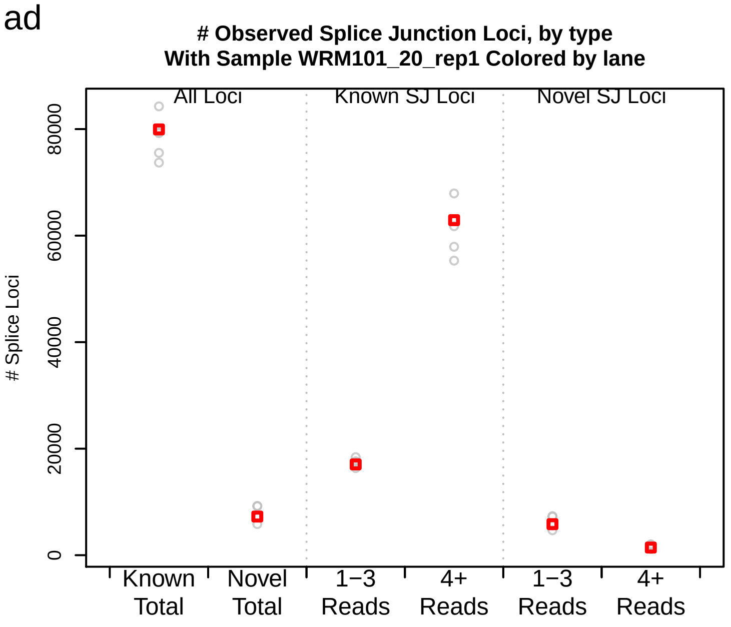
Observed Splice Junction Loci, by type
With Sample WRM101_20_rep1 Colored by lane
Splice Loci
0
20000
40000
60000
80000
All Loci
Known SJ Loci
Novel SJ Loci
Known Total
Novel Total
1−3 Reads
4+ Reads
1−3 Reads
4+ Reads

ae

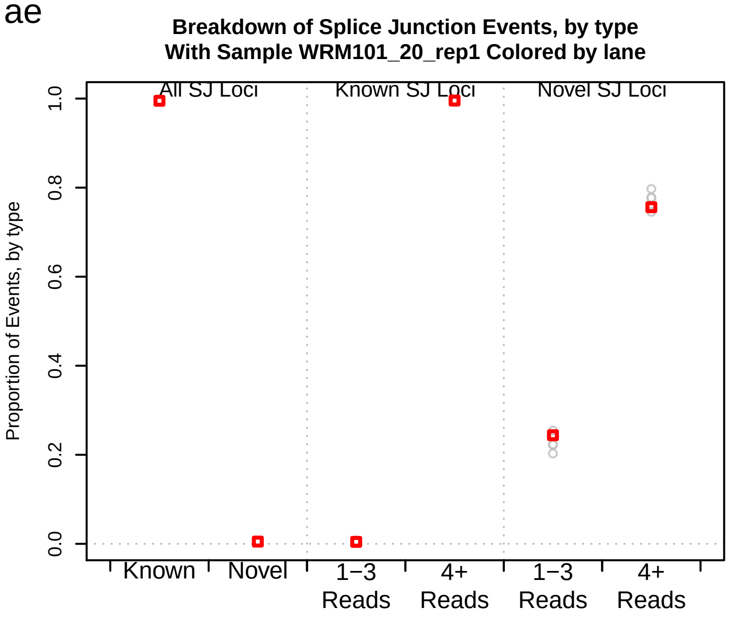

af

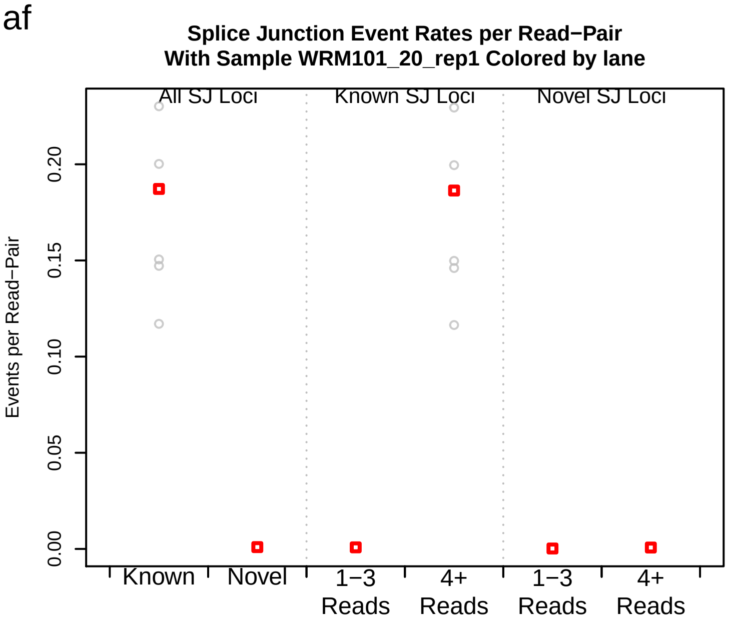

ag

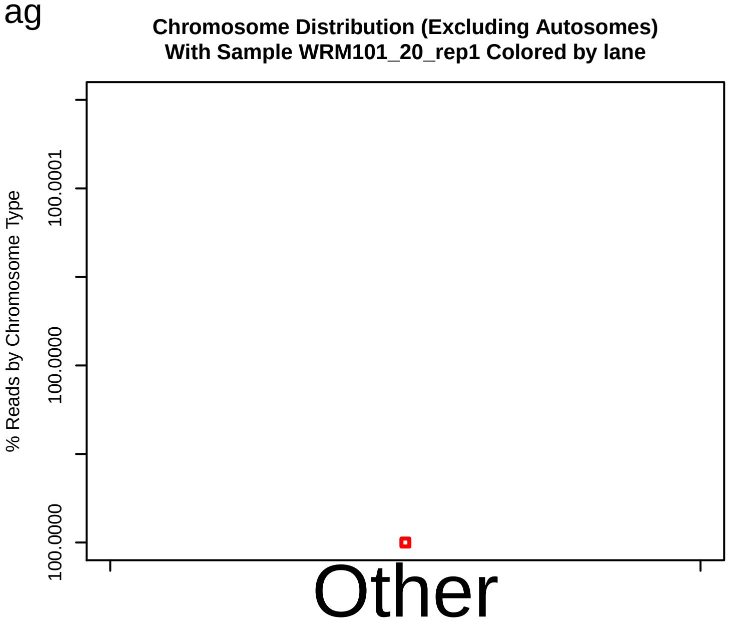

ah

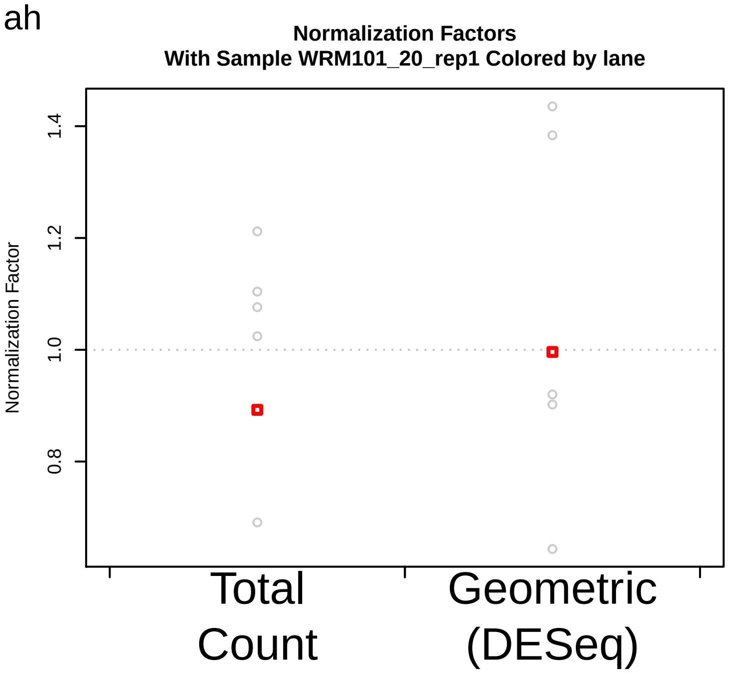

ai

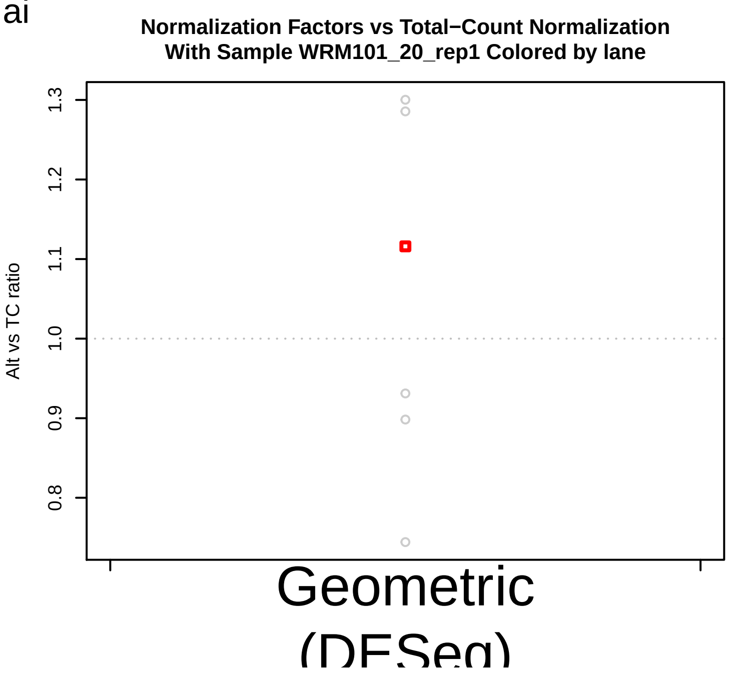

aj

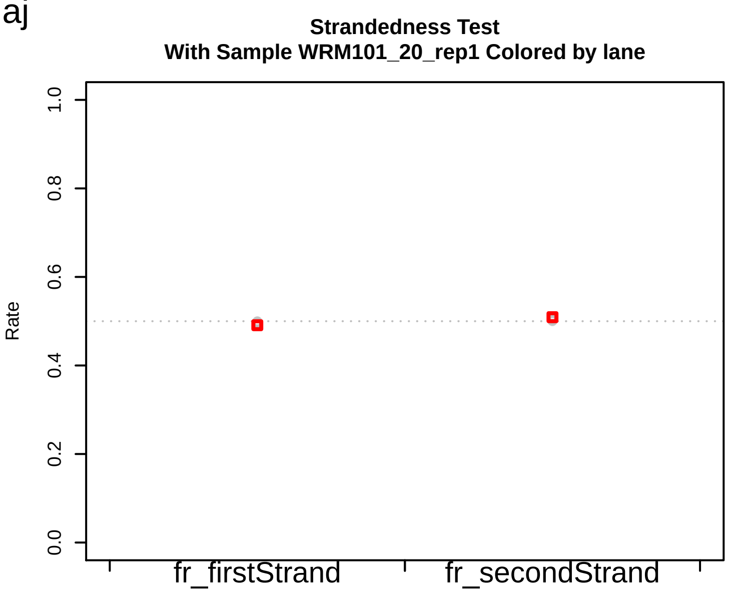

ak

Nucleotide Rate by Cycle, Leading Clipped bases (12)
With Sample WRM101_20_rep1 in Bold

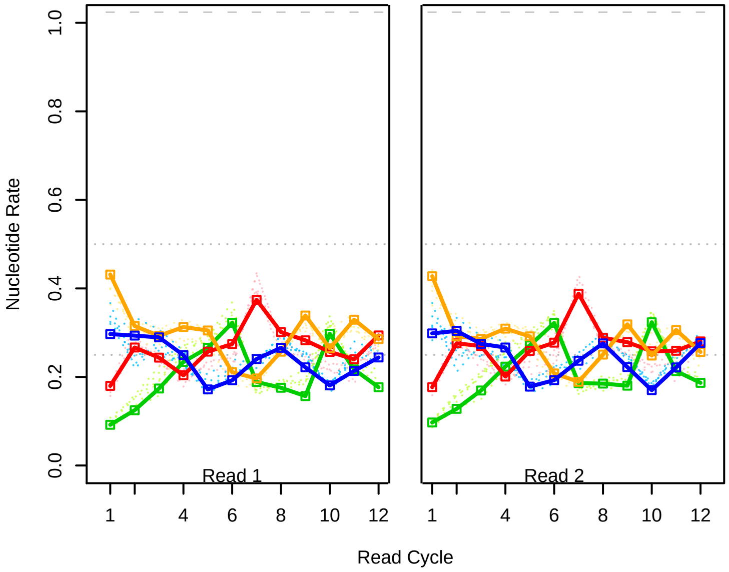

al

Nucleotide Rate by Cycle, Trailing Clipped bases (12)
With Sample WRM101_20_rep1 in Bold

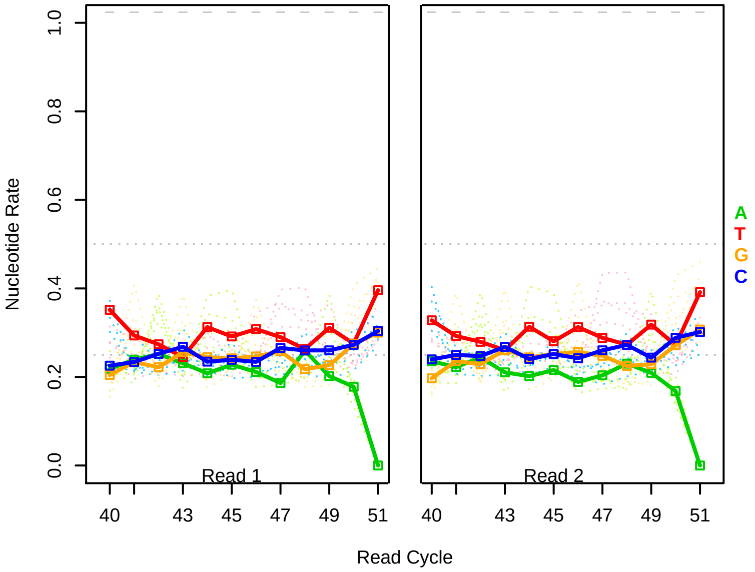

am

Raw Nucleotide Rate by Cycle
With Sample WRM101_20_rep1 in Bold

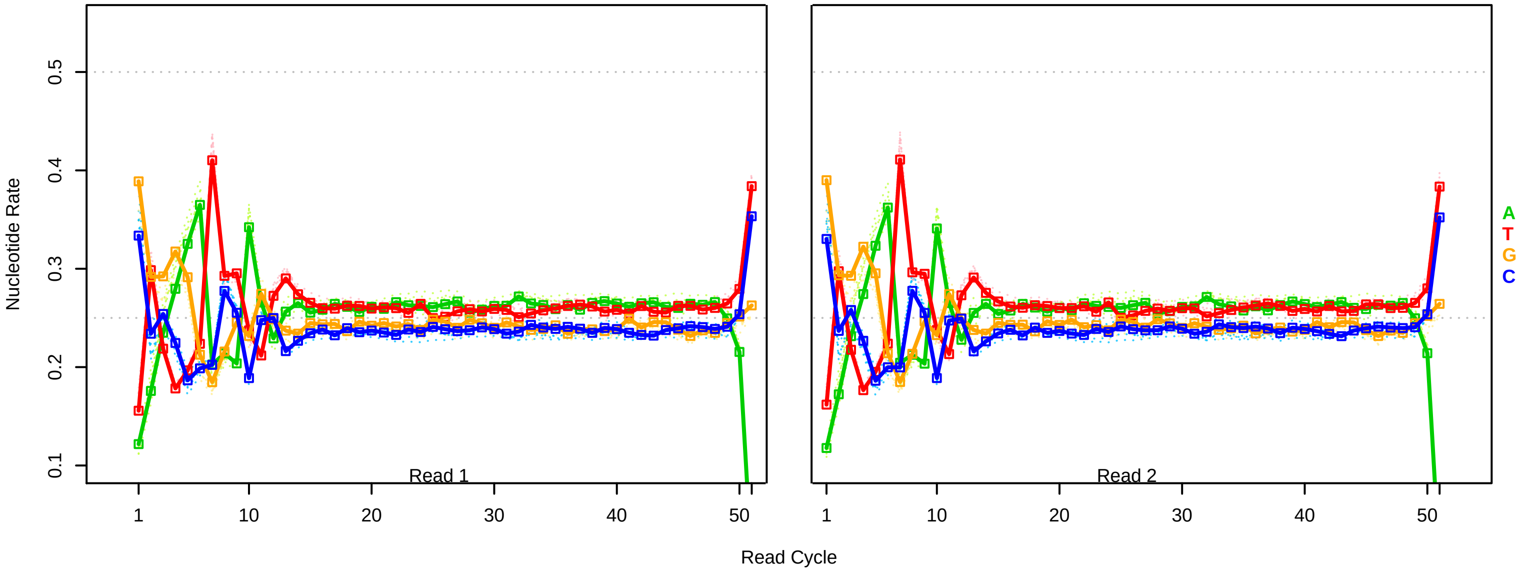

an

Nucleotide Rate by Cycle, Aligned bases only
With Sample WRM101_20_rep1 in Bold

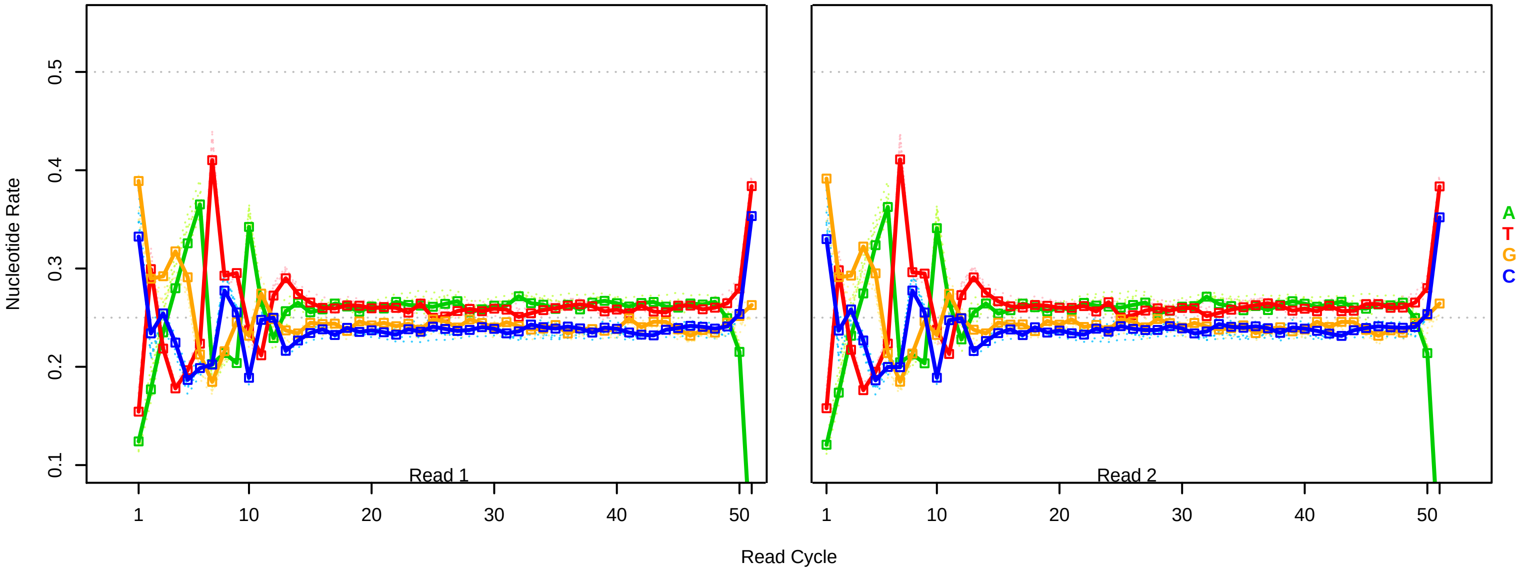

Supplement: S1 Data — (GZ) [file pgen.1012129.s007.gz › SupplementalDataSet2/OneStopRNASeq/bam_qc.zip!/bam_qc/QoRTs_MultiPlot/details/plot-sampleHL-coloredByLane-WRM101_20_rep1.pdf]
