# Supplementary material for: The pos-1 3′ untranslated region governs germline specification and proliferation to ensure reproductive robustness
Source: PLoS Genet. 2026 Apr 27;22(4):e1012129. doi: 10.1371/journal.pgen.1012129 (PMC13132445; doi:10.1371/journal.pgen.1012129)

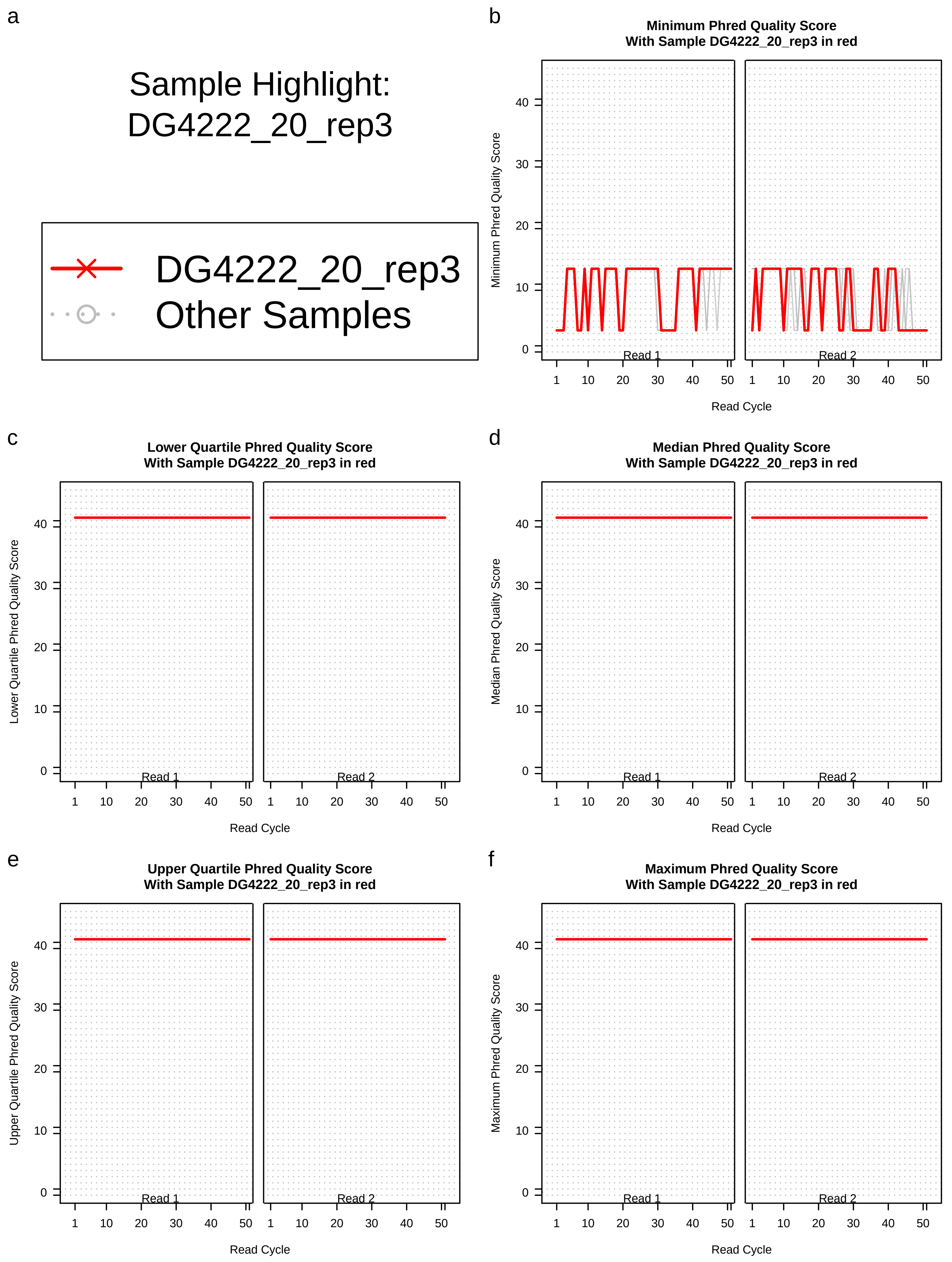
a
Sample Highlight:
DG4222_20_rep3
DG4222_20_rep3
Other Samples
b
Minimum Phred Quality Score
With Sample DG4222_20_rep3 in red
Minimum Phred Quality Score
Read 1
Read 2
Read Cycle
c
Lower Quartile Phred Quality Score
With Sample DG4222_20_rep3 in red
Lower Quartile Phred Quality Score
Read 1
Read 2
Read Cycle
d
Median Phred Quality Score
With Sample DG4222_20_rep3 in red
Median Phred Quality Score
Read 1
Read 2
Read Cycle
e
Upper Quartile Phred Quality Score
With Sample DG4222_20_rep3 in red
Upper Quartile Phred Quality Score
Read 1
Read 2
Read Cycle
f
Maximum Phred Quality Score
With Sample DG4222_20_rep3 in red
Maximum Phred Quality Score
Read 1
Read 2
Read Cycle
0
10
20
30
40
1
50

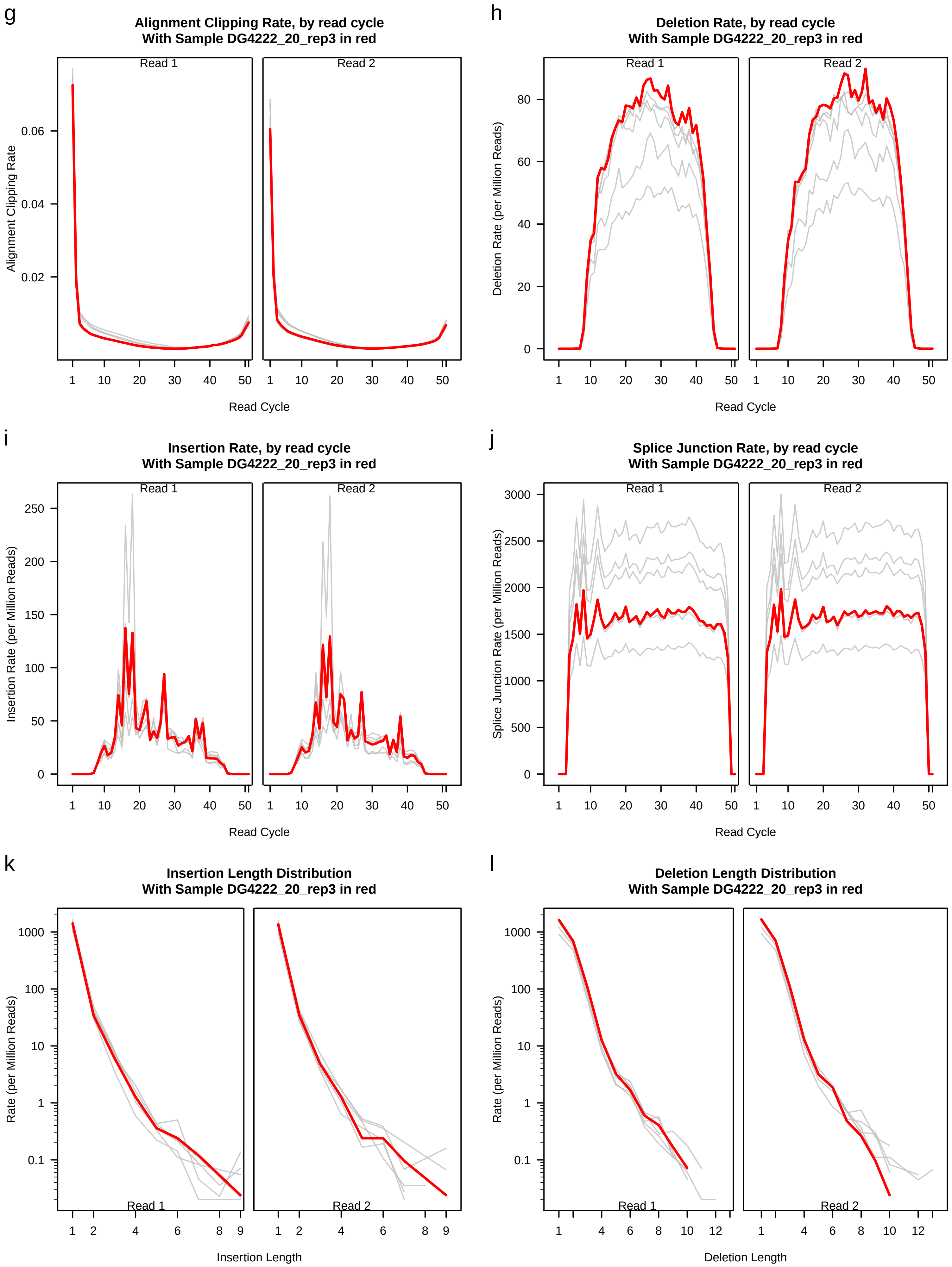
g
Alignment Clipping Rate, by read cycle
With Sample DG4222_20_rep3 in red
Read 1
Read 2
Alignment Clipping Rate
0.02
0.04
0.06
1 10 20 30 40 50
Read Cycle
h
Deletion Rate, by read cycle
With Sample DG4222_20_rep3 in red
Read 1
Read 2
Deletion Rate (per Million Reads)
0 20 40 60 80
Read Cycle
i
Insertion Rate, by read cycle
With Sample DG4222_20_rep3 in red
Read 1
Read 2
Insertion Rate (per Million Reads)
0 50 100 150 200 250
Read Cycle
j
Splice Junction Rate, by read cycle
With Sample DG4222_20_rep3 in red
Read 1
Read 2
Splice Junction Rate (per Million Reads)
0 500 1000 1500 2000 2500 3000
Read Cycle
k
Insertion Length Distribution
With Sample DG4222_20_rep3 in red
Read 1
Read 2
Rate (per Million Reads)
0.1 1 10 100 1000
1 2 4 6 8 9
Insertion Length
l
Deletion Length Distribution
With Sample DG4222_20_rep3 in red
Read 1
Read 2
Rate (per Million Reads)
1 4 6 8 10 12
Deletion Length

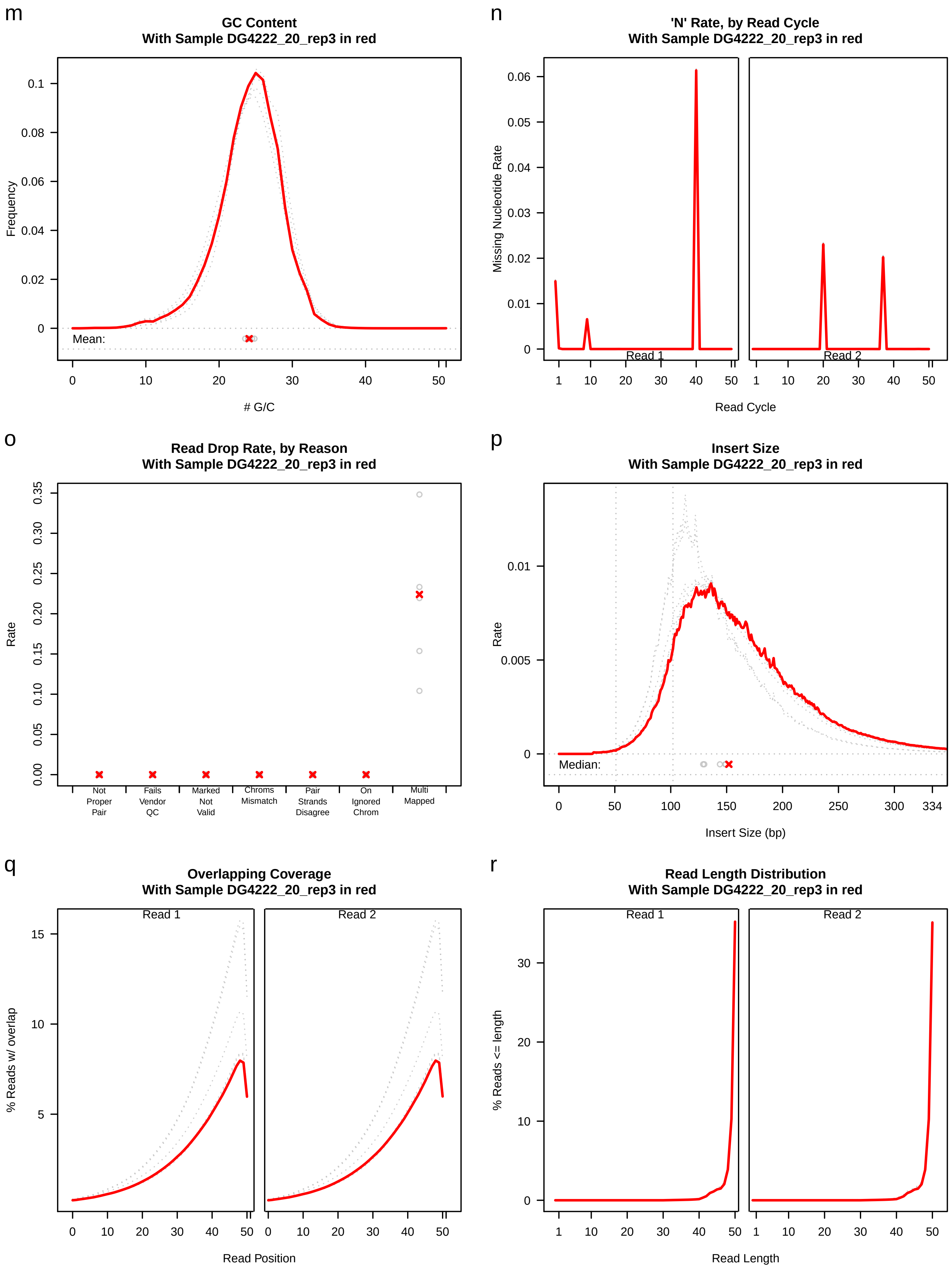

m
GC Content
With Sample DG4222_20_rep3 in red
Frequency
0.1
0.08
0.06
0.04
0.02
0
Mean:
0
10
20
30
40
50
G/C
n
'N' Rate, by Read Cycle
With Sample DG4222_20_rep3 in red
Missing Nucleotide Rate
0.06
0.05
0.04
0.03
0.02
0.01
0
Read 1
Read 2
1
10
20
30
40
50
Read Cycle
o
Read Drop Rate, by Reason
With Sample DG4222_20_rep3 in red
Rate
0.35
0.30
0.25
0.20
0.15
0.10
0.05
0.00
Not Proper Pair
Fails Vendor QC
Marked Not Valid
Chroms Mismatch
Pair Strands Disagree
On Ignored Chrom
Multi Mapped
p
Insert Size
With Sample DG4222_20_rep3 in red
Rate
0.01
0.005
0
Median:
0
50
100
150
200
250
300
334
Insert Size (bp)
q
Overlapping Coverage
With Sample DG4222_20_rep3 in red
% Reads w/ overlap
15
10
5
Read 1
Read 2
0
10
20
30
40
50
Read Position
r
Read Length Distribution
With Sample DG4222_20_rep3 in red
% Reads <= length
30
20
10
0
Read 1
Read 2
1
10
20
30
40
50
Read Length

s

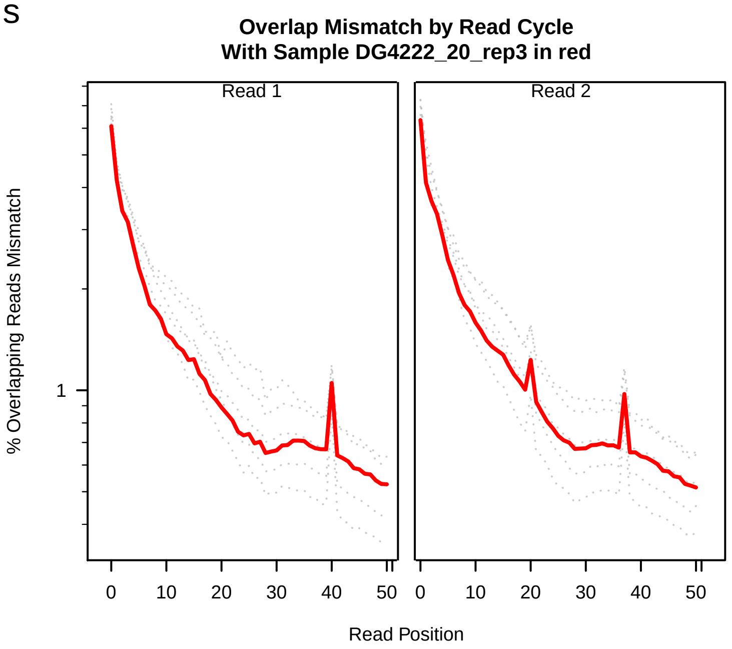

Overlap Mismatch by Read Cycle
With Sample DG4222_20_rep3 in red
Read 1
Read 2
% Overlapping Reads Mismatch
1
0 10 20 30 40 50
Read Position

t

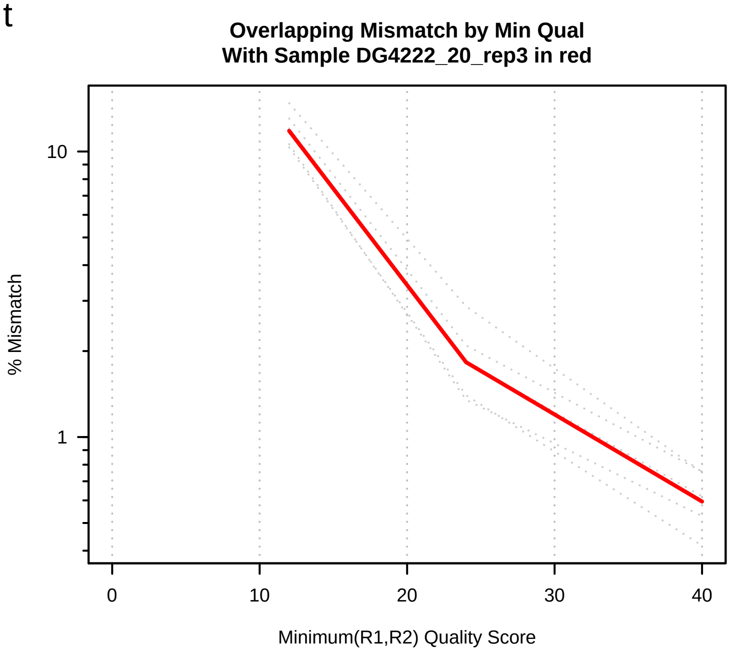

Overlapping Mismatch by Min Qual
With Sample DG4222_20_rep3 in red
% Mismatch
10
1
0 10 20 30 40
Minimum(R1,R2) Quality Score

u

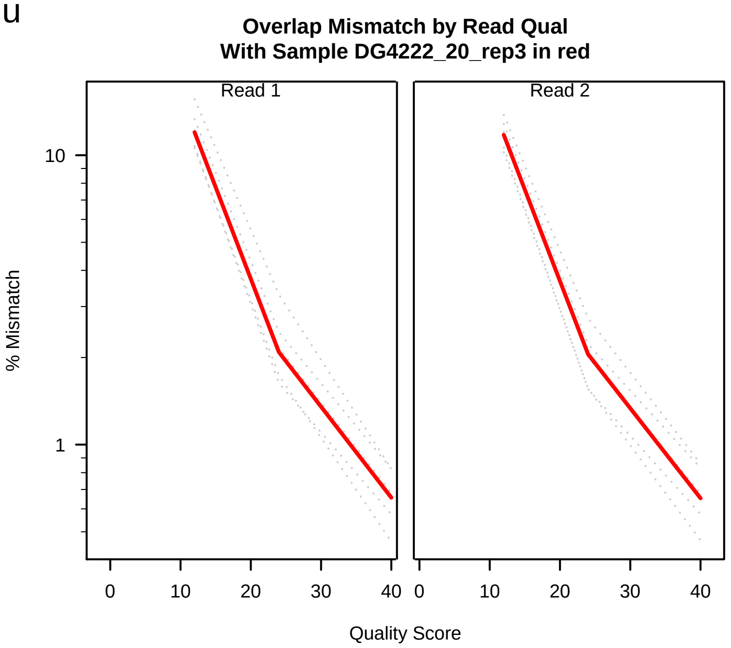

Overlap Mismatch by Read Qual
With Sample DG4222_20_rep3 in red
Read 1
Read 2
% Mismatch
10
1
0 10 20 30 40
Quality Score

v

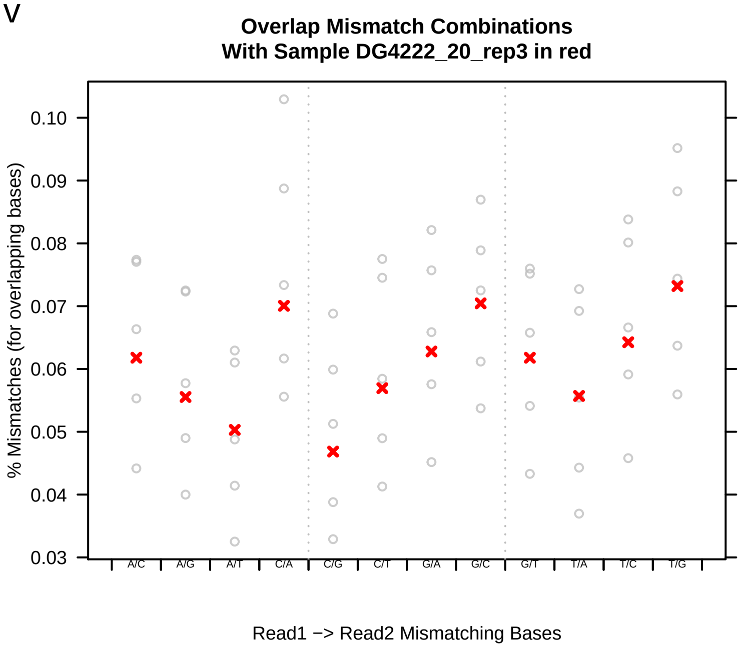

Overlap Mismatch Combinations
With Sample DG4222_20_rep3 in red
% Mismatches (for overlapping bases)
0.10
0.09
0.08
0.07
0.06
0.05
0.04
0.03
A/C A/G A/T C/A C/G C/T G/A G/C G/T T/A T/C T/G
Read1 -> Read2 Mismatching Bases

w

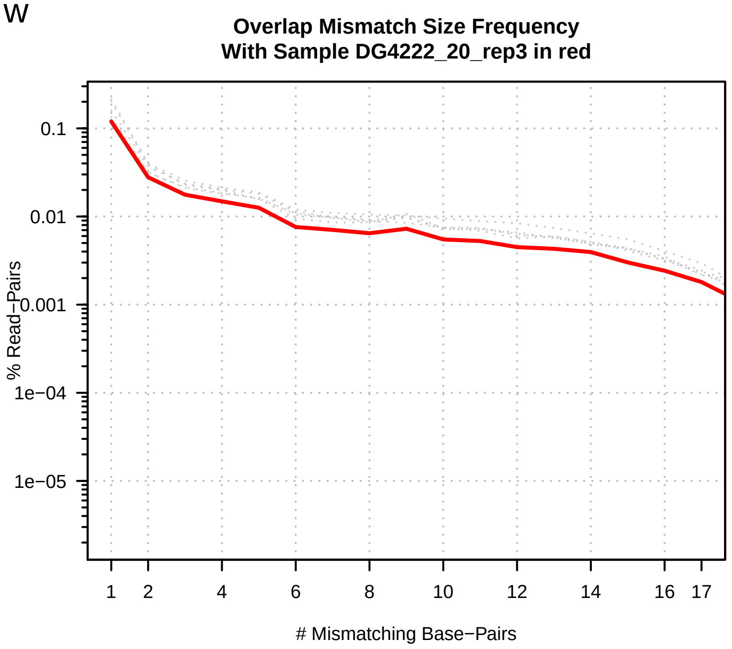

Overlap Mismatch Size Frequency
With Sample DG4222_20_rep3 in red
% Read−Pairs
0.1
0.01
0.001
1e−04
1e−05
1 2 4 6 8 10 12 14 16 17
Mismatching Base−Pairs

x

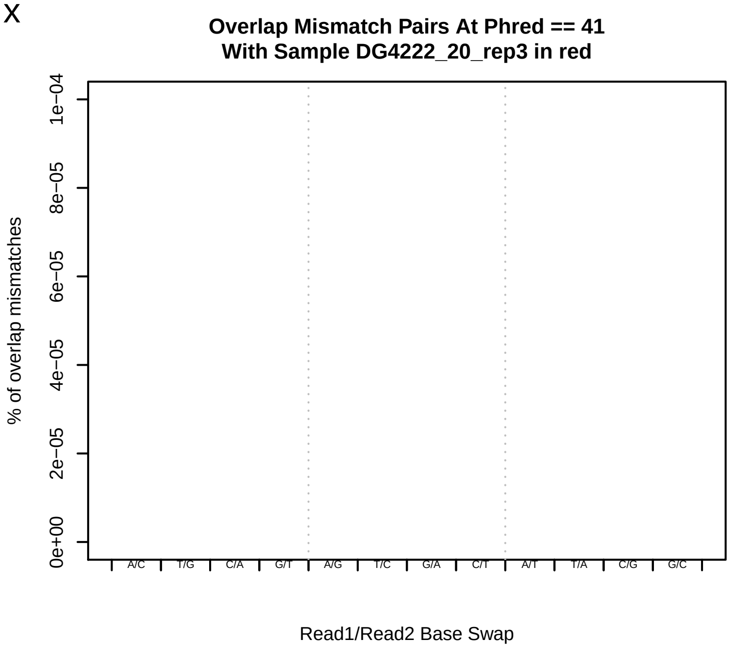

Overlap Mismatch Pairs At Phred == 41
With Sample DG4222_20_rep3 in red
% of overlap mismatches
1e−04
8e−05
6e−05
4e−05
2e−05
0e+00
A/C T/G C/A G/T A/G T/C G/A C/T A/T T/A C/G G/C
Read1/Read2 Base Swap

y

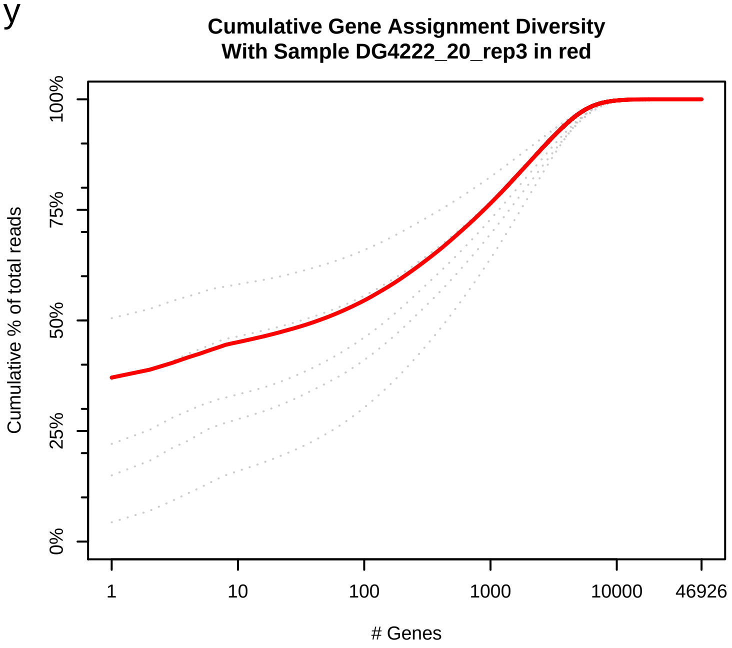
Cumulative Gene Assignment Diversity
With Sample DG4222_20_rep3 in red
Cumulative % of total reads
0%
25%
50%
75%
100%
1
10
100
1000
10000
46926
Genes

z

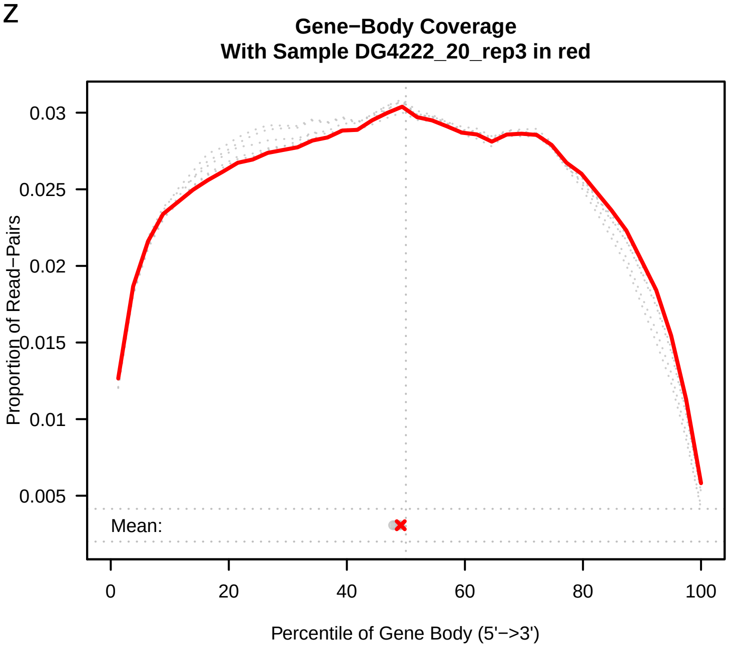
Gene−Body Coverage
With Sample DG4222_20_rep3 in red
Proportion of Read−Pairs
0.005
0.01
0.015
0.02
0.025
0.03
Mean:
0
20
40
60
80
100
Percentile of Gene Body (5'−>3')

aa

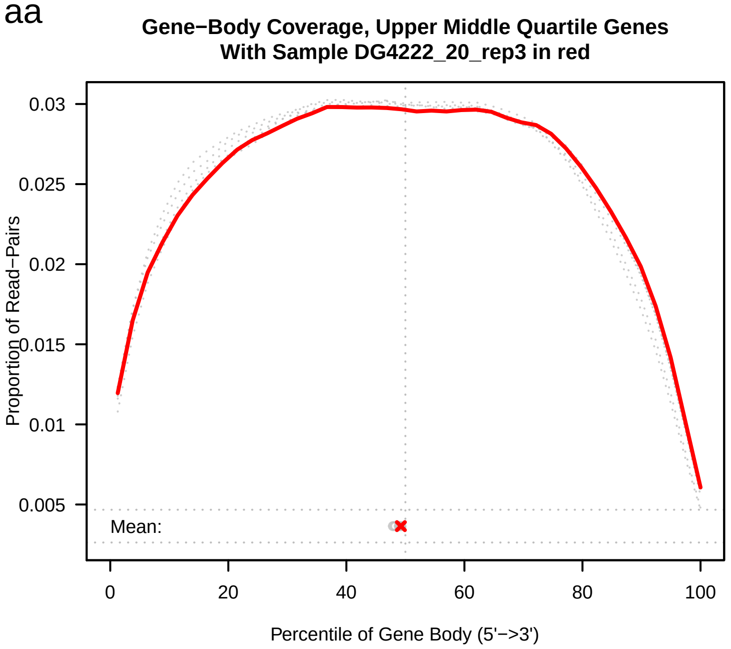
Gene−Body Coverage, Upper Middle Quartile Genes
With Sample DG4222_20_rep3 in red
Proportion of Read−Pairs
0.005
0.01
0.015
0.02
0.025
0.03
Mean:
0
20
40
60
80
100
Percentile of Gene Body (5'−>3')

ab

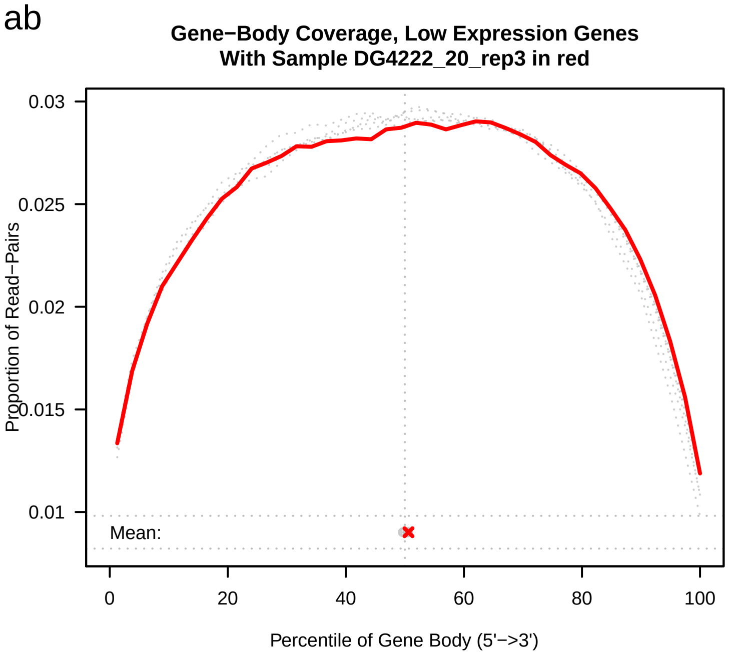
Gene−Body Coverage, Low Expression Genes
With Sample DG4222_20_rep3 in red
Proportion of Read−Pairs
0.01
0.015
0.02
0.025
0.03
Mean:
0
20
40
60
80
100
Percentile of Gene Body (5'−>3')

ac

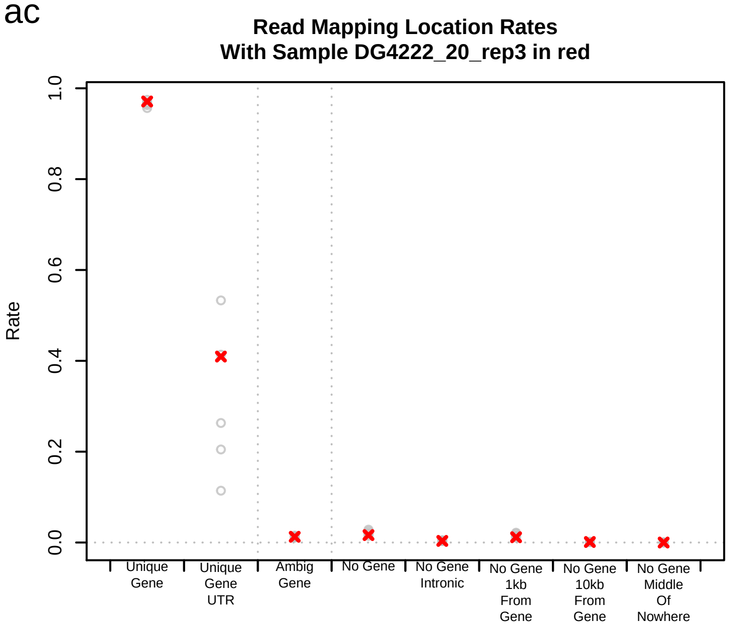
Read Mapping Location Rates
With Sample DG4222_20_rep3 in red
Rate
0.0
0.2
0.4
0.6
0.8
1.0
Unique Gene
Unique Gene UTR
Ambig Gene
No Gene
No Gene Intronic
No Gene 1kb From Gene
No Gene 10kb From Gene
No Gene Middle Of Nowhere

ad

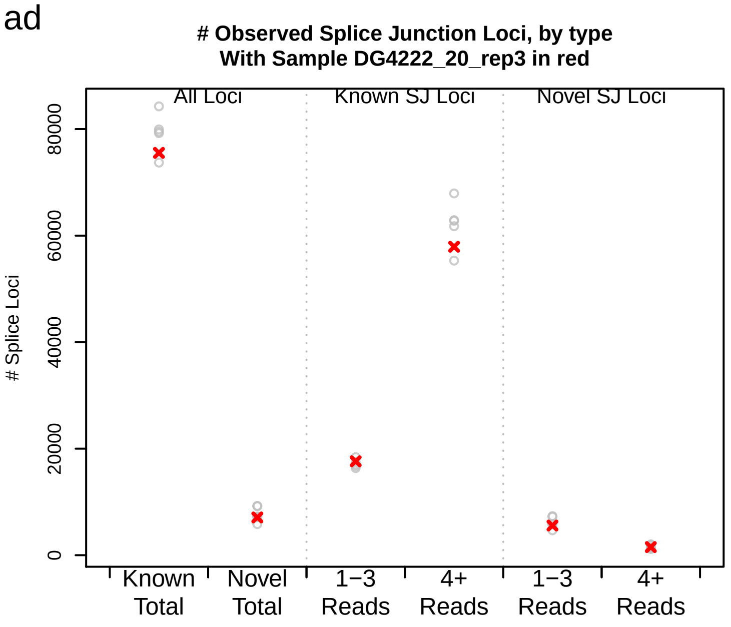
Observed Splice Junction Loci, by type
With Sample DG4222_20_rep3 in red
Splice Loci
0
20000
40000
60000
80000
All Loci
Known SJ Loci
Novel SJ Loci
Known Total
Novel Total
1−3 Reads
4+ Reads
1−3 Reads
4+ Reads

ae

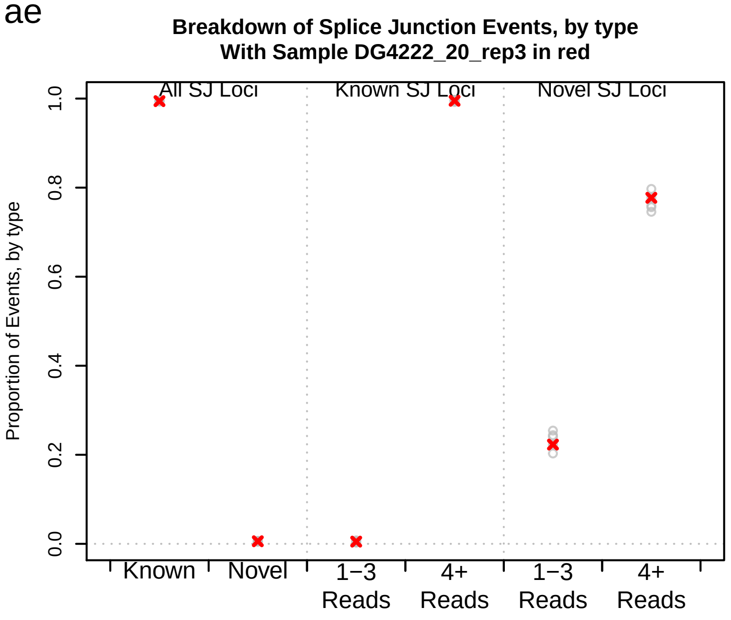

af

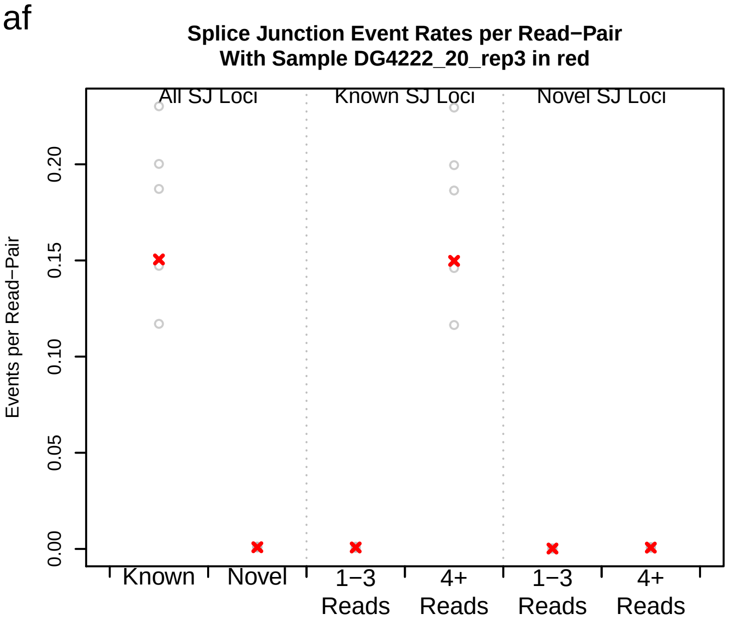

ag

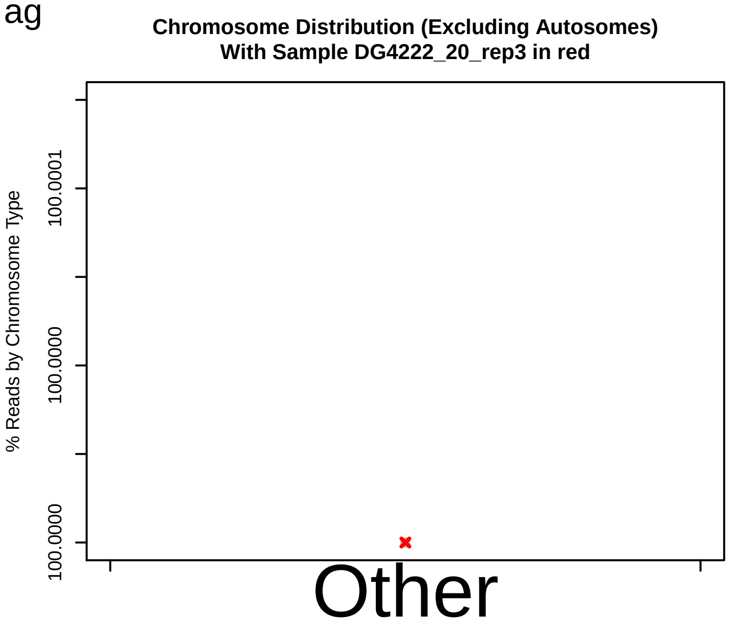

ah

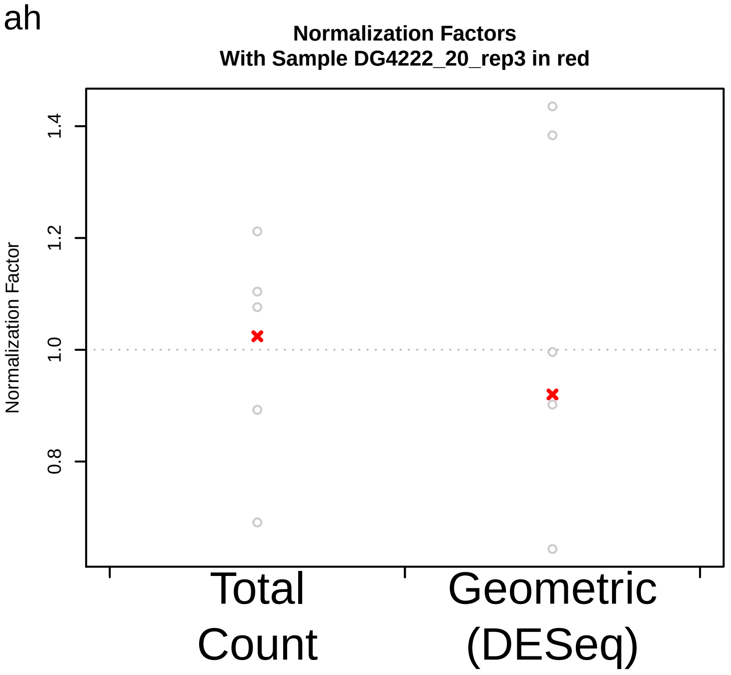

ai

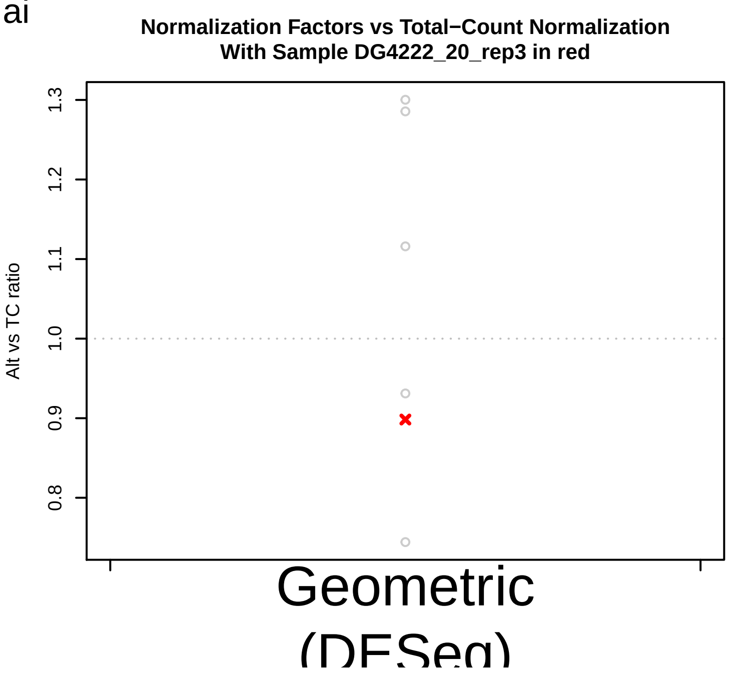

aj

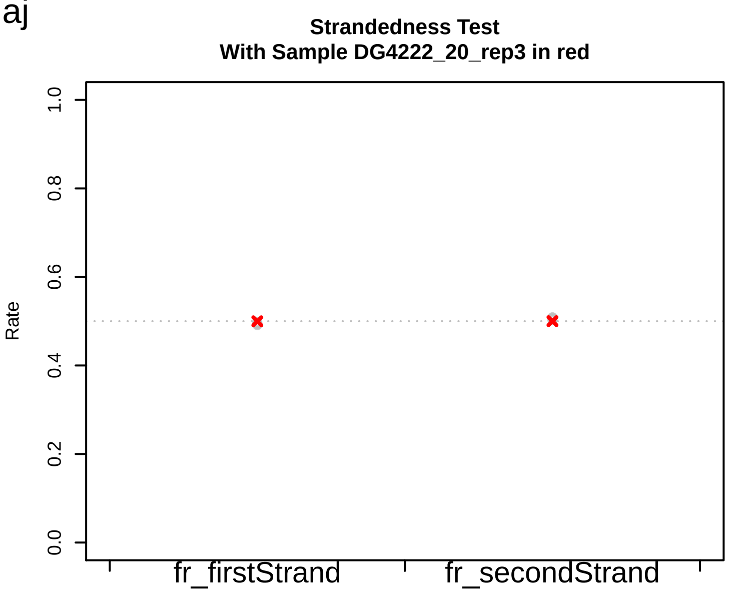

ak

Nucleotide Rate by Cycle, Leading Clipped bases (12)
With Sample DG4222_20_rep3 in red

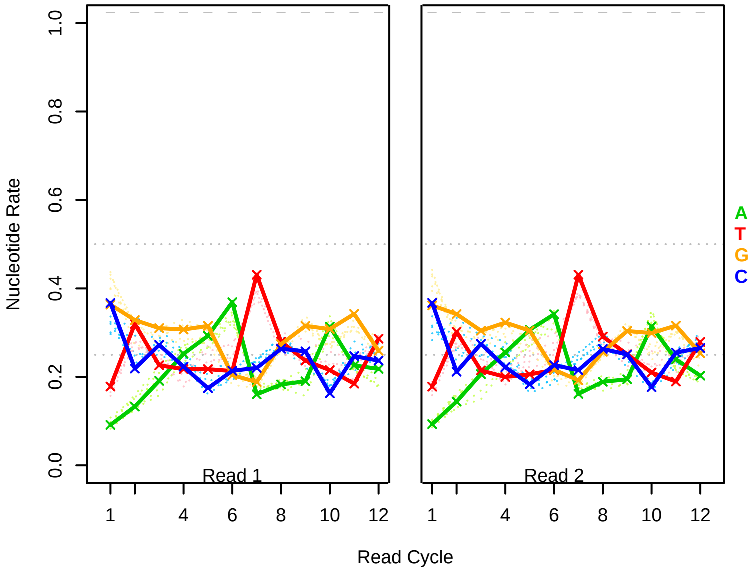

al

Nucleotide Rate by Cycle, Trailing Clipped bases (12)
With Sample DG4222_20_rep3 in red

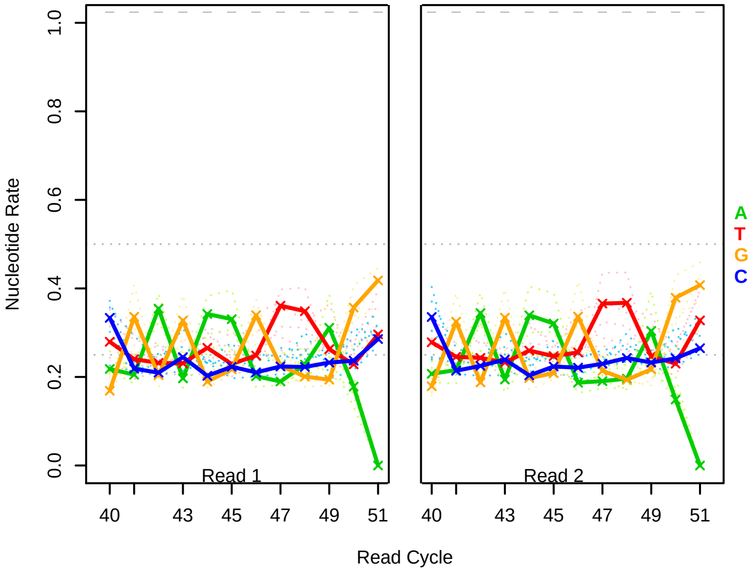

am

Raw Nucleotide Rate by Cycle
With Sample DG4222_20_rep3 in red

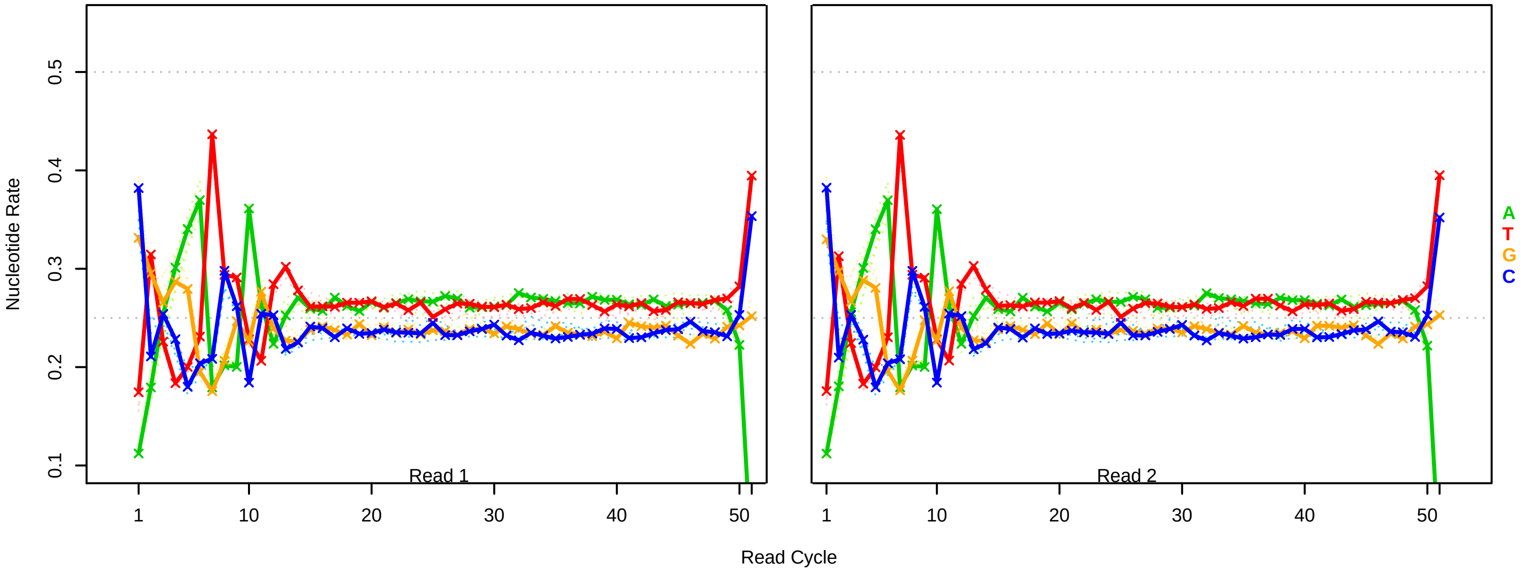

an

Nucleotide Rate by Cycle, Aligned bases only
With Sample DG4222_20_rep3 in red

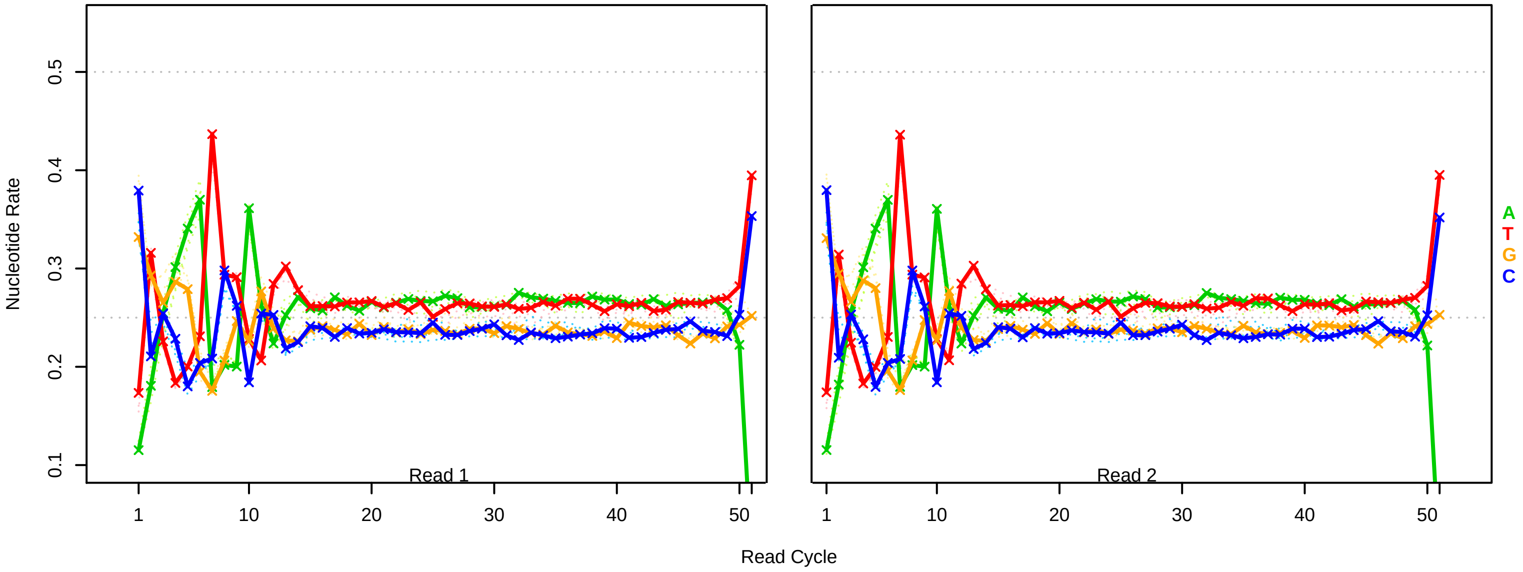

Supplement: S1 Data — (GZ) [file pgen.1012129.s007.gz › SupplementalDataSet2/OneStopRNASeq/bam_qc.zip!/bam_qc/QoRTs_MultiPlot/details/plot-sampleHL-DG4222_20_rep3.pdf]
