# Supplementary material for: The pos-1 3′ untranslated region governs germline specification and proliferation to ensure reproductive robustness
Source: PLoS Genet. 2026 Apr 27;22(4):e1012129. doi: 10.1371/journal.pgen.1012129 (PMC13132445; doi:10.1371/journal.pgen.1012129)
Supplement: S2 Data — (GZ) [file pgen.1012129.s008.gz › SupplementalDataSet1/03.Result_X202SC24112711-Z01-F001_C_elegans/result/08.VarDetect_Visualization/WRM103.Circos.pdf]

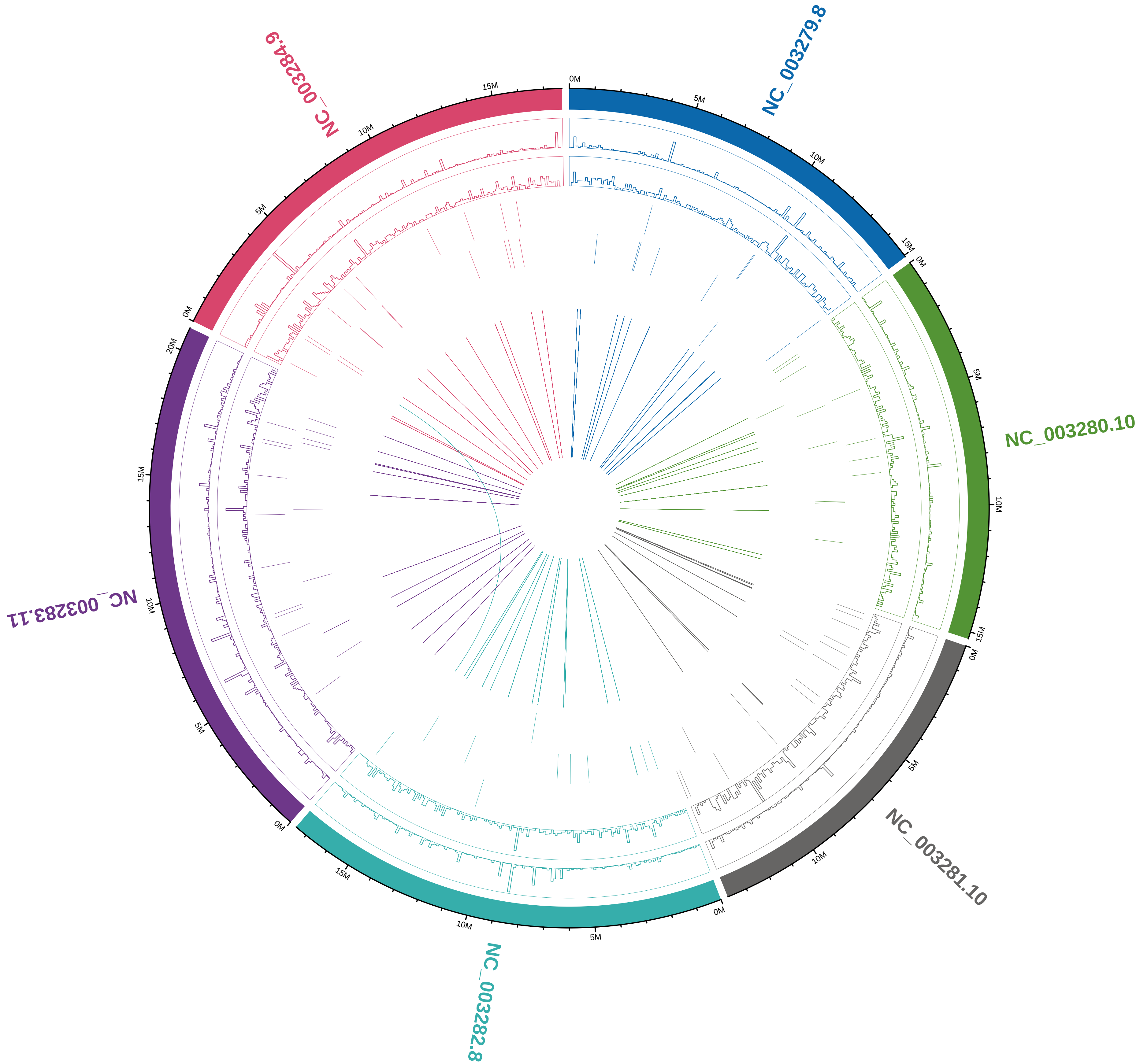
NC_003279.8
NC_003280.10
NC_003281.10
NC_003282.8
NC_003283.11
NC_003284.9
0M
5M
10M
15M
20M
