# Supplementary material for: The pos-1 3′ untranslated region governs germline specification and proliferation to ensure reproductive robustness
Source: PLoS Genet. 2026 Apr 27;22(4):e1012129. doi: 10.1371/journal.pgen.1012129 (PMC13132445; doi:10.1371/journal.pgen.1012129)
Supplement: S2 Data — (GZ) [file pgen.1012129.s008.gz › SupplementalDataSet1/03.Result_X202SC24112711-Z01-F001_C_elegans/result/05.InDel_VarDetect/VCFv4.2.pdf]

The Variant Call Format (VCF) Version 4.2 Specification

26 Jan 2015

The master version of this document can be found at `https://github.com/samtools/hts-specs`.
This printing is version cbd60fe from that repository, last modified on the date shown above.

1 The VCF specification

VCF is a text file format (most likely stored in a compressed manner). It contains meta-information lines, a header line, and then data lines each containing information about a position in the genome. The format also has the ability to contain genotype information on samples for each position.

1.1 An example

```
##fileformat=VCFv4.2
##fileDate=20090805
##source=myImputationProgramV3.1
##reference=file:///seq/references/1000GenomesPilot-NCBI36.fasta
##contig=<ID=20,length=62435964,assembly=B36,md5=f126cdf8a6e0c7f379d618ff66beb2da,species="Homo sapiens",taxonomy=x>
##phasing=partial
##INFO=<ID=NS,Number=1,Type=Integer,Description="Number of Samples With Data">
##INFO=<ID=DP,Number=1,Type=Integer,Description="Total Depth">
##INFO=<ID=AF,Number=A,Type=Float,Description="Allele Frequency">
##INFO=<ID=AA,Number=1,Type=String,Description="Ancestral Allele">
##INFO=<ID=DB,Number=0,Type=Flag,Description="dbSNP membership, build 129">
##INFO=<ID=H2,Number=0,Type=Flag,Description="HapMap2 membership">
##FILTER=<ID=q10,Description="Quality below 10">
##FILTER=<ID=s50,Description="Less than 50% of samples have data">
##FORMAT=<ID=GT,Number=1,Type=String,Description="Genotype">
##FORMAT=<ID=GQ,Number=1,Type=Integer,Description="Genotype Quality">
##FORMAT=<ID=DP,Number=1,Type=Integer,Description="Read Depth">
##FORMAT=<ID=HQ,Number=2,Type=Integer,Description="Haplotype Quality">
#CHROM POS     ID        REF    ALT     QUAL FILTER INFO                              FORMAT      NA00001        NA00002        NA00003
20     14370   rs6054257 G      A       29   PASS   NS=3;DP=14;AF=0.5;DB;H2           GT:GQ:DP:HQ 0|0:48:1:51,51 1|0:48:8:51,51 1/1:43:5:.,.
20     17330   .         T      A       3    q10    NS=3;DP=11;AF=0.017               GT:GQ:DP:HQ 0|0:49:3:58,50 0|1:3:5:65,3   0/0:41:3
20     1110696 rs6040355 A      G,T     67   PASS   NS=2;DP=10;AF=0.333,0.667;AA=T;DB GT:GQ:DP:HQ 1|2:21:6:23,27 2|1:2:0:18,2   2/2:35:4
20     1230237 .         T      .       47   PASS   NS=3;DP=13;AA=T                   GT:GQ:DP:HQ 0|0:54:7:56,60 0|0:48:4:51,51 0/0:61:2
20     1234567 microsat1 GTC    G,GTCT  50   PASS   NS=3;DP=9;AA=G                    GT:GQ:DP    0/1:35:4       0/2:17:2       1/1:40:3
```

This example shows (in order): a good simple SNP, a possible SNP that has been filtered out because its quality is below 10, a site at which two alternate alleles are called, with one of them (T) being ancestral (possibly a reference sequencing error), a site that is called monomorphic reference (i.e. with no alternate alleles), and a microsatellite with two alternative alleles, one a deletion of 2 bases (TC), and the other an insertion of one base (T). Genotype data are given for three samples, two of which are phased and the third unphased, with per sample genotype quality, depth and haplotype qualities (the latter only for the phased samples) given as well as the genotypes. The microsatellite calls are unphased.

1.2 Meta-information lines

File meta-information is included after the ## string and must be key=value pairs. It is strongly encouraged that information lines describing the INFO, FILTER and FORMAT entries used in the body of the VCF file be included in the meta-information section. Although they are optional, if these lines are present then they must be completely well-formed.

1.2.1 File format

A single 'fileformat' field is always required, must be the first line in the file, and details the VCF format version number. For example, for VCF version 4.2, this line should read:

```
##fileformat=VCFv4.2
```

1.2.2 Information field format

INFO fields should be described as follows (first four keys are required, source and version are recommended):

```
##INFO=<ID=ID,Number=number,Type=type,Description="description",Source="source",Version="version">
```

Possible Types for INFO fields are: Integer, Float, Flag, Character, and String. The Number entry is an Integer that describes the number of values that can be included with the INFO field. For example, if the INFO field contains a single number, then this value should be 1; if the INFO field describes a pair of numbers, then this value should be 2 and so on. There are also certain special characters used to define special cases:

- If the field has one value per alternate allele then this value should be 'A'.
- If the field has one value for each possible allele (including the reference), then this value should be 'R'.
- If the field has one value for each possible genotype (more relevant to the FORMAT tags) then this value should be 'G'.
- If the number of possible values varies, is unknown, or is unbounded, then this value should be '.'.

The 'Flag' type indicates that the INFO field does not contain a Value entry, and hence the Number should be 0 in this case. The Description value must be surrounded by double-quotes. Double-quote character can be escaped with backslash \ and backslash as \\. Source and Version values likewise should be surrounded by double-quotes and specify the annotation source (case-insensitive, e.g. "dbsnp") and exact version (e.g. "138"), respectively for computational use.

1.2.3 Filter field format

FILTERs that have been applied to the data should be described as follows:

```
##FILTER=<ID=ID,Description="description">
```

1.2.4 Individual format field format

Likewise, Genotype fields specified in the FORMAT field should be described as follows:

```
##FORMAT=<ID=ID,Number=number,Type=type,Description="description">
```

Possible Types for FORMAT fields are: Integer, Float, Character, and String (this field is otherwise defined precisely as the INFO field).

1.2.5 Alternative allele field format

Symbolic alternate alleles for imprecise structural variants:

```
##ALT=<ID=type,Description=description>
```

The ID field indicates the type of structural variant, and can be a colon-separated list of types and subtypes. ID values are case sensitive strings and may not contain whitespace or angle brackets. The first level type must be one of the following:

- DEL Deletion relative to the reference
- INS Insertion of novel sequence relative to the reference
- DUP Region of elevated copy number relative to the reference

- INV Inversion of reference sequence
- CNV Copy number variable region (may be both deletion and duplication)

The CNV category should not be used when a more specific category can be applied. Reserved subtypes include:

- DUP:TANDEM Tandem duplication
- DEL:ME Deletion of mobile element relative to the reference
- INS:ME Insertion of a mobile element relative to the reference

In addition, it is highly recommended (but not required) that the header include tags describing the reference and contigs backing the data contained in the file. These tags are based on the SQ field from the SAM spec; all tags are optional (see the VCF example above).

For all of the ##INFO, ##FORMAT, ##FILTER, and ##ALT metainformation, extra fields can be included after the default fields. For example:

```
##INFO=<ID=ID,Number=number,Type=type,Description="description",Source="description",Version="128">
```

In the above example, the extra fields of "Source" and "Version" are provided. Optional fields should be stored as strings even for numeric values.

1.2.6 Assembly field format

Breakpoint assemblies for structural variations may use an external file:

```
##assembly=url
```

The URL field specifies the location of a fasta file containing breakpoint assemblies referenced in the VCF records for structural variants via the BKPTID INFO key.

1.2.7 Contig field format

As with chromosomal sequences it is highly recommended (but not required) that the header include tags describing the contigs referred to in the VCF file. This furthermore allows these contigs to come from different files. The format is identical to that of a reference sequence, but with an additional URL tag to indicate where that sequence can be found. For example:.

```
##contig=<ID=ctg1,URL=ftp://somewhere.org/assembly.fa,...>
```

1.2.8 Sample field format

It is possible to define sample to genome mappings as shown below:

```
##SAMPLE=<ID=S_ID,Genomes=G1_ID;G2_ID; ...;GK_ID,Mixture=N1;N2; ...;NK,Description=S1;S2; ...;SK>
```

1.2.9 Pedigree field format

It is possible to record relationships between genomes using the following syntax:

```
##PEDIGREE=<Name_0=G0-ID,Name_1=G1-ID,...,Name_N=GN-ID>
```

or a link to a database:

```
##pedigreeDB=<url>
```

1.3 Header line syntax

The header line names the 8 fixed, mandatory columns. These columns are as follows:

1. #CHROM
2. POS
3. ID
4. REF
5. ALT
6. QUAL
7. FILTER
8. INFO

If genotype data is present in the file, these are followed by a FORMAT column header, then an arbitrary number of sample IDs. The header line is tab-delimited.

1.4 Data lines

1.4.1 Fixed fields

There are 8 fixed fields per record. All data lines are tab-delimited. In all cases, missing values are specified with a dot ('.'). Fixed fields are:

1. CHROM - chromosome: An identifier from the reference genome or an angle-bracketed ID String ("<ID>") pointing to a contig in the assembly file (cf. the ##assembly line in the header). All entries for a specific CHROM should form a contiguous block within the VCF file. The colon symbol (:) must be absent from all chromosome names to avoid parsing errors when dealing with breakends. (String, no white-space permitted, Required).

2. POS - position: The reference position, with the 1st base having position 1. Positions are sorted numerically, in increasing order, within each reference sequence CHROM. It is permitted to have multiple records with the same POS. Telomeres are indicated by using positions 0 or N+1, where N is the length of the corresponding chromosome or contig. (Integer, Required)

3. ID - identifier: Semi-colon separated list of unique identifiers where available. If this is a dbSNP variant it is encouraged to use the rs number(s). No identifier should be present in more than one data record. If there is no identifier available, then the missing value should be used. (String, no white-space or semi-colons permitted)

4. REF - reference base(s): Each base must be one of A,C,G,T,N (case insensitive). Multiple bases are permitted. The value in the POS field refers to the position of the first base in the String. For simple insertions and deletions in which either the REF or one of the ALT alleles would otherwise be null/empty, the REF and ALT Strings must include the base before the event (which must be reflected in the POS field), unless the event occurs at position 1 on the contig in which case it must include the base after the event; this padding base is not required (although it is permitted) for e.g. complex substitutions or other events where all alleles have at least one base represented in their Strings. If any of the ALT alleles is a symbolic allele (an angle-bracketed ID String "<ID>") then the padding base is required and POS denotes the coordinate of the base preceding the polymorphism. Tools processing VCF files are not required to preserve case in the allele Strings. (String, Required).

5. ALT - alternate base(s): Comma separated list of alternate non-reference alleles called on at least one of the samples. Options are base Strings made up of the bases A,C,G,T,N,*, (case insensitive) or an angle-bracketed ID String ("<ID>") or a breakend replacement string as described in the section on breakends. The '*' allele is reserved to indicate that the allele is missing due to a upstream deletion. If there are no alternative alleles, then the missing value should be used. Tools processing VCF files are not required to preserve case in the allele String, except for IDs, which are case sensitive. (String; no whitespace, commas, or angle-brackets are permitted in the ID String itself)

6. QUAL - quality: Phred-scaled quality score for the assertion made in ALT. i.e. $-10log_{10}$ prob(call in ALT is wrong). If ALT is '.' (no variant) then this is $-10log_{10}$ prob(variant), and if ALT is not '.' this is $-10log_{10}$ prob(no variant). If unknown, the missing value should be specified. (Numeric)

7. FILTER - filter status: PASS if this position has passed all filters, i.e. a call is made at this position. Otherwise, if the site has not passed all filters, a semicolon-separated list of codes for filters that fail. e.g. "q10;s50" might indicate that at this site the quality is below 10 and the number of samples with data is below 50% of the total number of samples. '0' is reserved and should not be used as a filter String. If filters have not been applied, then this field should be set to the missing value. (String, no white-space or semi-colons permitted)

8. INFO - additional information: (String, no white-space, semi-colons, or equals-signs permitted; commas are permitted only as delimiters for lists of values) INFO fields are encoded as a semicolon-separated series of short keys with optional values in the format: <key>=<data>[,data]. Arbitrary keys are permitted, although the following sub-fields are reserved (albeit optional):

    - AA : ancestral allele
    - AC : allele count in genotypes, for each ALT allele, in the same order as listed
    - AF : allele frequency for each ALT allele in the same order as listed: use this when estimated from primary data, not called genotypes
    - AN : total number of alleles in called genotypes
    - BQ : RMS base quality at this position
    - CIGAR : cigar string describing how to align an alternate allele to the reference allele
    - DB : dbSNP membership
    - DP : combined depth across samples, e.g. DP=154
    - END : end position of the variant described in this record (for use with symbolic alleles)
    - H2 : membership in hapmap2
    - H3 : membership in hapmap3
    - MQ : RMS mapping quality, e.g. MQ=52
    - MQ0 : Number of MAPQ == 0 reads covering this record
    - NS : Number of samples with data
    - SB : strand bias at this position
    - SOMATIC : indicates that the record is a somatic mutation, for cancer genomics
    - VALIDATED : validated by follow-up experiment
    - 1000G : membership in 1000 Genomes

The exact format of each INFO sub-field should be specified in the meta-information (as described above). Example for an INFO field: DP=154;MQ=52;H2. Keys without corresponding values are allowed in order to indicate group membership (e.g. H2 indicates the SNP is found in HapMap 2). It is not necessary to list all the properties that a site does NOT have, by e.g. H2=0. See below for additional reserved INFO sub-fields used to encode structural variants.

1.4.2 Genotype fields

If genotype information is present, then the same types of data must be present for all samples. First a FORMAT field is given specifying the data types and order (colon-separated alphanumeric String). This is followed by one field per sample, with the colon-separated data in this field corresponding to the types specified in the format. The first sub-field must always be the genotype (GT) if it is present. There are no required sub-fields.

As with the INFO field, there are several common, reserved keywords that are standards across the community:

- GT : genotype, encoded as allele values separated by either of / or |. The allele values are 0 for the reference allele (what is in the REF field), 1 for the first allele listed in ALT, 2 for the second allele list in ALT and so on. For diploid calls examples could be 0/1, 1 | 0, or 1/2, etc. For haploid calls, e.g. on Y, male non-pseudoautosomal X, or mitochondrion, only one allele value should be given; a triploid call might look like 0/0/1. If a call cannot be made for a sample at a given locus, '.' should be specified for each missing allele

in the GT field (for example './.' for a diploid genotype and '.' for haploid genotype). The meanings of the separators are as follows (see the PS field below for more details on incorporating phasing information into the genotypes):

  - / : genotype unphased
  - | : genotype phased

- DP : read depth at this position for this sample (Integer)
- FT : sample genotype filter indicating if this genotype was "called" (similar in concept to the FILTER field). Again, use PASS to indicate that all filters have been passed, a semi-colon separated list of codes for filters that fail, or '.' to indicate that filters have not been applied. These values should be described in the meta-information in the same way as FILTERs (String, no white-space or semi-colons permitted)
- GL : genotype likelihoods comprised of comma separated floating point log_{10}-scaled likelihoods for all possible genotypes given the set of alleles defined in the REF and ALT fields. In presence of the GT field the same ploidy is expected and the canonical order is used; without GT field, diploidy is assumed. If A is the allele in REF and B,C,... are the alleles as ordered in ALT, the ordering of genotypes for the likelihoods is given by: F(j/k) = (k*(k+1)/2)+j. In other words, for biallelic sites the ordering is: AA,AB,BB; for triallelic sites the ordering is: AA,AB,BB,AC,BC,CC, etc. For example: GT:GL 0/1:-323.03,-99.29,-802.53 (Floats)
- GLE : genotype likelihoods of heterogeneous ploidy, used in presence of uncertain copy number. For example: GLE=0:-75.22,1:-223.42,0/0:-323.03,1/0:-99.29,1/1:-802.53 (String)
- PL : the phred-scaled genotype likelihoods rounded to the closest integer (and otherwise defined precisely as the GL field) (Integers)
- GP : the phred-scaled genotype posterior probabilities (and otherwise defined precisely as the GL field); intended to store imputed genotype probabilities (Floats)
- GQ : conditional genotype quality, encoded as a phred quality $-10log_{10}$ p(genotype call is wrong, conditioned on the site's being variant) (Integer)
- HQ : haplotype qualities, two comma separated phred qualities (Integers)
- PS : phase set. A phase set is defined as a set of phased genotypes to which this genotype belongs. Phased genotypes for an individual that are on the same chromosome and have the same PS value are in the same phased set. A phase set specifies multi-marker haplotypes for the phased genotypes in the set. All phased genotypes that do not contain a PS subfield are assumed to belong to the same phased set. If the genotype in the GT field is unphased, the corresponding PS field is ignored. The recommended convention is to use the position of the first variant in the set as the PS identifier (although this is not required). (Non-negative 32-bit Integer)
- PQ : phasing quality, the phred-scaled probability that alleles are ordered incorrectly in a heterozygote (against all other members in the phase set). We note that we have not yet included the specific measure for precisely defining "phasing quality"; our intention for now is simply to reserve the PQ tag for future use as a measure of phasing quality. (Integer)
- EC : comma separated list of expected alternate allele counts for each alternate allele in the same order as listed in the ALT field (typically used in association analyses) (Integers)
- MQ : RMS mapping quality, similar to the version in the INFO field. (Integer)

If any of the fields is missing, it is replaced with the missing value. For example if the FORMAT is GT:GQ:DP:HQ then $0 \mid 0 : . : 23 : 23, 34$ indicates that GQ is missing. Trailing fields can be dropped (with the exception of the GT field, which should always be present if specified in the FORMAT field).

See below for additional genotype fields used to encode structural variants. Additional Genotype fields can be defined in the meta-information. However, software support for such fields is not guaranteed.

2 Understanding the VCF format and the haplotype representation

VCF records use a single general system for representing genetic variation data composed of:

- Allele: representing single genetic haplotypes (A, T, ATC).
- Genotype: an assignment of alleles for each chromosome of a single named sample at a particular locus.
- VCF record: a record holding all segregating alleles at a locus (as well as genotypes, if appropriate, for multiple individuals containing alleles at that locus).

VCF records use a simple haplotype representation for REF and ALT alleles to describe variant haplotypes at a locus. ALT haplotypes are constructed from the REF haplotype by taking the REF allele bases at the POS in the reference genotype and replacing them with the ALT bases. In essence, the VCF record specifies a-REF-t and the alternative haplotypes are a-ALT-t for each alternative allele.

3 INFO keys used for structural variants

When the INFO keys reserved for encoding structural variants are used for imprecise variants, the values should be best estimates. When a key reflects a property of a single alt allele (e.g. SVLEN), then when there are multiple alt alleles there will be multiple values for the key corresponding to each alelle (e.g. SVLEN=-100,-110 for a deletion with two distinct alt alleles).

The following INFO keys are reserved for encoding structural variants. In general, when these keys are used by imprecise variants, the values should be best estimates. When a key reflects a property of a single alt allele (e.g. SVLEN), then when there are multiple alt alleles there will be multiple values for the key corresponding to each alelle (e.g. SVLEN=-100,-110 for a deletion with two distinct alt alleles).

```
##INFO=<ID=IMPRECISE,Number=0,Type=Flag,Description="Imprecise structural variation">
##INFO=<ID=NOVEL,Number=0,Type=Flag,Description="Indicates a novel structural variation">
##INFO=<ID=END,Number=1,Type=Integer,Description="End position of the variant described in this record">
```

For precise variants, END is POS + length of REF allele - 1, and the for imprecise variants the corresponding best estimate.

```
##INFO=<ID=SVTYPE,Number=1,Type=String,Description="Type of structural variant">
```

Value should be one of DEL, INS, DUP, INV, CNV, BND. This key can be derived from the REF/ALT fields but is useful for filtering.

```
##INFO=<ID=SVLEN,Number=.,Type=Integer,Description="Difference in length between REF and ALT alleles">
```

One value for each ALT allele. Longer ALT alleles (e.g. insertions) have positive values, shorter ALT alleles (e.g. deletions) have negative values.

```
##INFO=<ID=CIPOS,Number=2,Type=Integer,Description="Confidence interval around POS for imprecise variants">
##INFO=<ID=CIEND,Number=2,Type=Integer,Description="Confidence interval around END for imprecise variants">
##INFO=<ID=HOMLEN,Number=.,Type=Integer,Description="Length of base pair identical micro-homology at event breakpoints">
##INFO=<ID=HOMSEQ,Number=.,Type=String,Description="Sequence of base pair identical micro-homology at event breakpoints">
##INFO=<ID=BKPTID,Number=.,Type=String,Description="ID of the assembled alternate allele in the assembly file">
```

For precise variants, the consensus sequence the alternate allele assembly is derivable from the REF and ALT fields. However, the alternate allele assembly file may contain additional information about the characteristics of the alt allele contigs.

```
##INFO=<ID=MEINFO,Number=4,Type=String,Description="Mobile element info of the form NAME,START,END,POLARITY">
##INFO=<ID=METRANS,Number=4,Type=String,Description="Mobile element transduction info of the form CHR,START,END,POLARITY">
##INFO=<ID=DGVID,Number=1,Type=String,Description="ID of this element in Database of Genomic Variation">
##INFO=<ID=DBVARID,Number=1,Type=String,Description="ID of this element in DBVAR">
##INFO=<ID=DBRIPID,Number=1,Type=String,Description="ID of this element in DBRIP">
##INFO=<ID=MATEID,Number=.,Type=String,Description="ID of mate breakends">
##INFO=<ID=PARID,Number=1,Type=String,Description="ID of partner breakend">
##INFO=<ID=EVENT,Number=1,Type=String,Description="ID of event associated to breakend">
##INFO=<ID=CILEN,Number=2,Type=Integer,Description="Confidence interval around the inserted material between breakends">
##INFO=<ID=DP,Number=1,Type=Integer,Description="Read Depth of segment containing breakend">
##INFO=<ID=DPADJ,Number=.,Type=Integer,Description="Read Depth of adjacency">
##INFO=<ID=CN,Number=1,Type=Integer,Description="Copy number of segment containing breakend">
##INFO=<ID=CNADJ,Number=.,Type=Integer,Description="Copy number of adjacency">
##INFO=<ID=CICN,Number=2,Type=Integer,Description="Confidence interval around copy number for the segment">
##INFO=<ID=CICNADJ,Number=.,Type=Integer,Description="Confidence interval around copy number for the adjacency">
```

4 FORMAT keys used for structural variants

```
##FORMAT=<ID=CN,Number=1,Type=Integer,Description="Copy number genotype for imprecise events">
##FORMAT=<ID=CNQ,Number=1,Type=Float,Description="Copy number genotype quality for imprecise events">
##FORMAT=<ID=CNL,Number=.,Type=Float,Description="Copy number genotype likelihood for imprecise events">
##FORMAT=<ID=NQ,Number=1,Type=Integer,Description="Phred style probability score that the variant is novel">
##FORMAT=<ID=HAP,Number=1,Type=Integer,Description="Unique haplotype identifier">
##FORMAT=<ID=AHAP,Number=1,Type=Integer,Description="Unique identifier of ancestral haplotype">
```

These keys are analogous to GT/GQ/GL and are provided for genotyping imprecise events by copy number (either because there is an unknown number of alternate alleles or because the haplotypes cannot be determined). CN specifies the integer copy number of the variant in this sample. CNQ is encoded as a phred quality $-10log_{10}$ p(copy number genotype call is wrong). CNL specifies a list of log_{10} likelihoods for each potential copy number, starting from zero. When possible, GT/GQ/GL should be used instead of (or in addition to) these keys.

5 Representing variation in VCF records

5.1 Creating VCF entries for SNPs and small indels

5.1.1 Example 1

For example, suppose we are looking at a locus in the genome:

Example	Sequence	Alteration
Ref	a t C g a	C is the reference base
1	a t G g a	C base is a G in some individuals
2	a t - g a	C base is deleted w.r.t. the reference sequence
3	a t CAg a	A base is inserted w.r.t. the reference sequence

Representing these as VCF records would be done as follows:

1. A SNP polymorphism of C/G $\rightarrow \{C, G\} \rightarrow$ C is the reference allele
2. A single base deletion of C $\rightarrow \{tC, t\} \rightarrow$ tC is the reference allele
3. A single base insertion of A $\rightarrow \{tC, tCA\} \rightarrow$ tC is the reference allele

#CHROM	POS	ID	REF	ALT	QUAL	FILTER	INFO
20	3	.	C	G	.	PASS	DP=100
20	2	.	TC	T	.	PASS	DP=100
20	2	.	TC	TCA	.	PASS	DP=100

5.1.2 Example 2

Suppose I see a the following in a population of individuals and want to represent these three segregating alleles:

Example	Sequence	Alteration
Ref	a t C g a	C is the reference base
1	a t G g a	C base is a G in some individuals
2	a t - g a	C base is deleted w.r.t. the reference sequence

In this case there are three segregating alleles: $\{tC, tG, t\}$ with a corresponding VCF record:

#CHROM	POS	ID	REF	ALT	QUAL	FILTER	INFO
20	2	.	TC	TG,T	.	PASS	DP=100

5.1.3 Example 3

Now suppose I have this more complex example:

Example	Sequence	Alteration
Ref	a t C g a	C is the reference base
1	a t - g a	C base is is deleted w.r.t. the reference sequence
2	a t - - a	C and G bases are deleted w.r.t. the reference sequence
3	a t CAg a	A base is inserted w.r.t. the reference sequence

There are actually four segregating alleles: $\{tCg, tg, t, tCAg\}$ over bases 2-4. This complex set of allele is represented in VCF as:

#CHROM	POS	ID	REF	ALT	QUAL	FILTER	INFO
20	2	.	TCG	TG,T,TCAG	.	PASS	DP=100

Note that in VCF records, the molecular equivalence explicitly listed above in the per-base alignment is discarded, so the actual placement of equivalent g isn't retained. For completeness, VCF records are dynamically typed, so whether a VCF record is a SNP, Indel, Mixed, or Reference site depends on the properties of the alleles in the record.

5.2 Decoding VCF entries for SNPs and small indels

5.2.1 SNP VCF record

Suppose I receive the following VCF record:

#CHROM	POS	ID	REF	ALT	QUAL	FILTER	INFO
20	3	.	C	T	.	PASS	DP=100

This is a SNP since its only single base substitution and there are only two alleles so I have the two following segregating haplotypes:

Example	Sequence	Alteration
Ref	`a t C g a`	C is the reference base
1	`a t T g a`	C base is a T in some individuals

5.2.2 Insertion VCF record

Suppose I receive the following VCF record:

#CHROM	POS	ID	REF	ALT	QUAL	FILTER	INFO
20	3	.	C	CTAG	.	PASS	DP=100

This is a insertion since the reference base C is being replaced by C [the reference base] plus three insertion bases TAG. Again there are only two alleles so I have the two following segregating haplotypes:

Example	Sequence	Alteration
Ref	`a t C - - - g a`	C is the reference base
1	`a t C T A G g a`	following the C base is an insertion of 3 bases

5.2.3 Deletion VCF record

Suppose I receive the following VCF record:

#CHROM	POS	ID	REF	ALT	QUAL	FILTER	INFO
20	2	.	TCG	T	.	PASS	DP=100

This is a deletion of two reference bases since the reference allele TCG is being replaced by just the T [the reference base]. Again there are only two alleles so I have the two following segregating haplotypes:

Example	Sequence	Alteration
Ref	`a T C G a`	T is the (first) reference base
1	`a T - - a`	following the T base is a deletion of 2 bases

5.2.4 Mixed VCF record for a microsatellite

Suppose I receive the following VCF record:

#CHROM	POS	ID	REF	ALT	QUAL	FILTER	INFO
20	4	.	GCG	G,GCGCG	.	PASS	DP=100

This is a mixed type record containing a 2 base insertion and a 2 base deletion. There are are three segregating alleles so I have the three following haplotypes:

Example	Sequence	Alteration
Ref	`a t c G C G - - a`	G is the (first) reference base
1	`a t c G - - - - a`	following the G base is a deletion of 2 bases
2	`a t c G C G C G a`	following the G base is a insertion of 2 bases

Note that in all of these examples dashes have been added to make the haplotypes clearer but of course the equivalence among bases isn't provided by the VCF. Technically the following is an equivalent alignment:

Example	Sequence	Alteration
Ref	`a t c G - - C G a`	G is the (first) reference base
1	`a t c G - - - - a`	following the G base is a deletion of 2 bases
2	`a t c G C G C G a`	following the G base is a insertion of 2 bases

5.3 Encoding Structural Variants

The following page contains examples of structural variants encoded in VCF:

VCF STRUCTURAL VARIANT EXAMPLE

```
##fileformat=VCFv4.1
##fileDate=20100501
##reference=1000GenomesPilot-NCBI36
##assembly=ftp://ftp-trace.ncbi.nih.gov/1000genomes/ftp/release/sv/breakpoint_assemblies.fasta
##INFO=<ID=BKPTID,Number=.,Type=String,Description="ID of the assembled alternate allele in the assembly file">
##INFO=<ID=CIEND,Number=2,Type=Integer,Description="Confidence interval around END for imprecise variants">
##INFO=<ID=CIPOS,Number=2,Type=Integer,Description="Confidence interval around POS for imprecise variants">
##INFO=<ID=END,Number=1,Type=Integer,Description="End position of the variant described in this record">
##INFO=<ID=HOMLEN,Number=.,Type=Integer,Description="Length of base pair identical micro-homology at event breakpoints">
##INFO=<ID=HOMSEQ,Number=.,Type=String,Description="Sequence of base pair identical micro-homology at event breakpoints">
##INFO=<ID=SVLEN,Number=.,Type=Integer,Description="Difference in length between REF and ALT alleles">
##INFO=<ID=SVTYPE,Number=1,Type=String,Description="Type of structural variant">
##ALT=<ID=DEL,Description="Deletion">
##ALT=<ID=DEL:ME:ALU,Description="Deletion of ALU element">
##ALT=<ID=DEL:ME:L1,Description="Deletion of L1 element">
##ALT=<ID=DUP,Description="Duplication">
##ALT=<ID=DUP:TANDEM,Description="Tandem Duplication">
##ALT=<ID=INS,Description="Insertion of novel sequence">
##ALT=<ID=INS:ME:ALU,Description="Insertion of ALU element">
##ALT=<ID=INS:ME:L1,Description="Insertion of L1 element">
##ALT=<ID=INV,Description="Inversion">
##ALT=<ID=CNV,Description="Copy number variable region">
##FORMAT=<ID=GT,Number=1,Type=String,Description="Genotype">
##FORMAT=<ID=GQ,Number=1,Type=Float,Description="Genotype quality">
##FORMAT=<ID=CN,Number=1,Type=Integer,Description="Copy number genotype for imprecise events">
##FORMAT=<ID=CNQ,Number=1,Type=Float,Description="Copy number genotype quality for imprecise events">
#CHROM POS     ID        REF              ALT          QUAL FILTER INFO                                                               FORMAT       NA00001
1      2827694 rs2376870 CGTGGATGCGGGGAC  C            .    PASS   SVTYPE=DEL;END=2827762;HOMLEN=1;HOMSEQ=G;SVLEN=-68                 GT:GQ        1/1:13.9
2       321682 .         T                <DEL>        6    PASS   SVTYPE=DEL;END=321887;SVLEN=-205;CIPOS=-56,20;CIEND=-10,62         GT:GQ        0/1:12
2     14477084 .         C                <DEL:ME:ALU> 12   PASS   SVTYPE=DEL;END=14477381;SVLEN=-297;CIPOS=-22,18;CIEND=-12,32       GT:GQ        0/1:12
3      9425916 .         C                <INS:ME:L1>  23   PASS   SVTYPE=INS;END=9425916;SVLEN=6027;CIPOS=-16,22                     GT:GQ        1/1:15
3     12665100 .         A                <DUP>        14   PASS   SVTYPE=DUP;END=12686200;SVLEN=21100;CIPOS=-500,500;CIEND=-500,500  GT:GQ:CN:CNQ ./.:0:3:16.2
4     18665128 .         T                <DUP:TANDEM> 11   PASS   SVTYPE=DUP;END=18665204;SVLEN=76;CIPOS=-10,10;CIEND=-10,10         GT:GQ:CN:CNQ ./.:0:5:8.3
```

The example shows in order:

1. A precise deletion with known breakpoint, a one base micro-homology, and a sample that is homozygous for the deletion.
2. An imprecise deletion of approximately 105 bp.
3. An imprecise deletion of an ALU element relative to the reference.
4. An imprecise insertion of an L1 element relative to the reference.
5. An imprecise duplication of approximately 21Kb. The sample genotype is copy number 3 (one extra copy of the duplicated sequence).
6. An imprecise tandem duplication of 76bp. The sample genotype is copy number 5 (but the two haplotypes are not known).

5.4 Specifying complex rearrangements with breakends

An arbitrary rearrangement event can be summarized as a set of novel **adjacencies**. Each adjacency ties together 2 **breakends**. The two breakends at either end of a novel adjacency are called **mates**.

There is one line of VCF (i.e. one record) for each of the two breakends in a novel adjacency. A breakend record is identified with the tag "SYTYPE=BND" in the INFO field. The REF field of a breakend record indicates a base or sequence s of bases beginning at position POS, as in all VCF records. The ALT field of a breakend record indicates a replacement for s. This "breakend replacement" has three parts:

1. The string t that replaces places s. The string t may be an extended version of s if some novel bases are inserted during the formation of the novel adjacency.
2. The position p of the mate breakend, indicated by a string of the form "chr:pos". This is the location of the first mapped base in the piece being joined at this novel adjacency.
3. The direction that the joined sequence continues in, starting from p. This is indicated by the orientation of square brackets surrounding p.

These 3 elements are combined in 4 possible ways to create the ALT. In each of the 4 cases, the assertion is that s is replaced with t, and then some piece starting at position p is joined to t. The cases are:

REF	ALT	Meaning
s	t[p[	piece extending to the right of p is joined after t
s	t]p]	reverse comp piece extending left of p is joined after t
s	]p]t	piece extending to the left of p is joined before t
s	[p[t	reverse comp piece extending right of p is joined before t

The example in Figure 1 shows a 3-break operation involving 6 breakends. It exemplifies all possible orientations of breakends in adjacencies. Notice how the ALT field expresses the orientation of the breakends.

#CHROM	POS	ID	REF	ALT	QUAL	FILTER	INFO
2	321681	bnd_W	G	G]17:198982]	6	PASS	SVTYPE=BND
2	321682	bnd_V	T	]13:123456]T	6	PASS	SVTYPE=BND
13	123456	bnd_U	C	C[2:321682[	6	PASS	SVTYPE=BND
13	123457	bnd_X	A	[17:198983[A	6	PASS	SVTYPE=BND
17	198982	bnd_Y	A	A]2:321681]	6	PASS	SVTYPE=BND
17	198983	bnd_Z	C	[13:123457[C	6	PASS	SVTYPE=BND

5.4.1 Inserted Sequence

Sometimes, as shown in Figure 2, some bases are inserted between the two breakends, this information is also carried in the ALT column:

#CHROM	POS	ID	REF	ALT	QUAL	FILTER	INFO
2	321682	bnd_V	T	]13 : 123456]AGTNNNNNCAT	6	PASS	SVTYPE=BND;MATEID=bnd_U
13	123456	bnd_U	C	CAGTNNNNNCA[2 : 321682[	6	PASS	SVTYPE=BND;MATEID=bnd_V

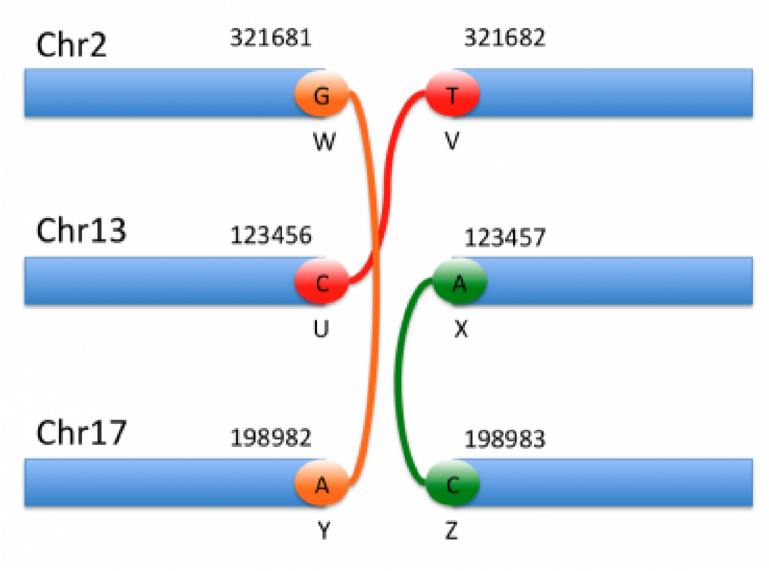

Figure 1: All possible orientations of breakends

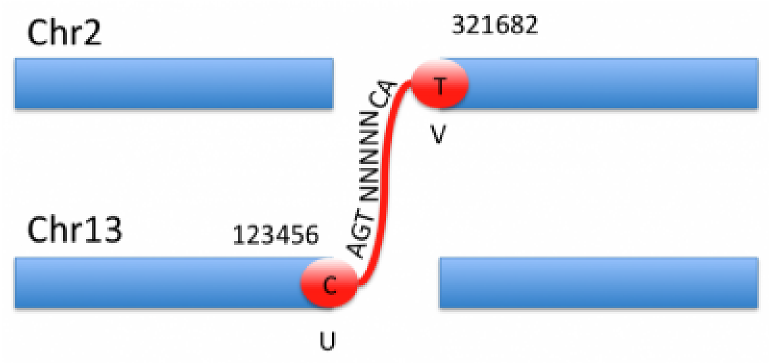

Figure 2: Inserted sequence between breakends

5.4.2 Large Insertions

If the insertion is too long to be conveniently stored in the ALT column, as in the 329 base insertion shown in Figure 3, it can be represented by a contig from the assembly file:

#CHROM	POS	ID	REF	ALT	QUAL	FILTER	INFO
13	123456	bnd_U	C	C[<ctg1>: 1[	6	PASS	SVTYPE=BND
13	123457	bnd_V	A	] <ctg1 >: 329]A	6	PASS	SVTYPE=BND

Note: In the special case of the complete insertion of a sequence between two base pairs, it is recommended to use the shorthand notation described above:

#CHROM	POS	ID	REF	ALT	QUAL	FILTER	INFO
13	321682	INS0	T	C<ctg1 >	6	PASS	SVTYPE=INS

If only a portion of <ctg1 >, say from position 7 to position 214, is inserted, the VCF would be:

#CHROM	POS	ID	REF	ALT	QUAL	FILTER	INFO
13	123456	bnd_U	C	C[<ctg1>: 7[	6	PASS	SVTYPE=BND
13	123457	bnd_V	A	] <ctg1 >: 214]A	6	PASS	SVTYPE=BND

If <ctg1 > is circular and a segment from position 229 to position 45 is inserted, i.e. continuing from position 329 on to position 1, this is represented by adding a circular adjacency:

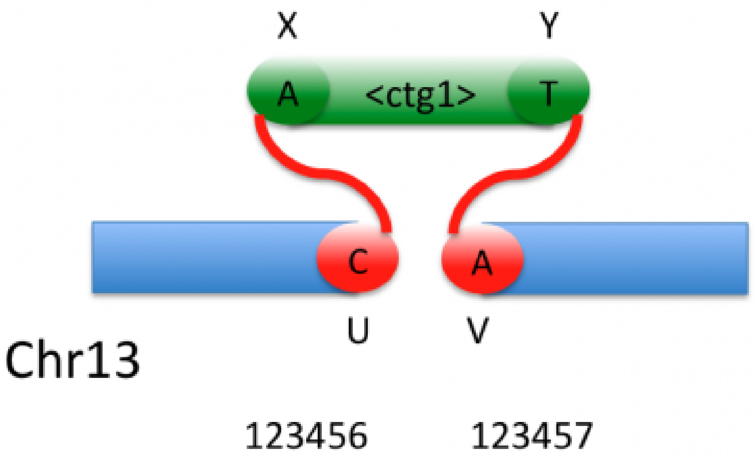

Figure 3: Inserted contig

#CHROM	POS	ID	REF	ALT	QUAL	FILTER	INFO
13	123456	bnd_U	C	C[<ctg1 >: 229[	6	PASS	SVTYPE=BND
13	123457	bnd_V	A	] <ctg1 >: 45]A	6	PASS	SVTYPE=BND
<ctg1 >	1	bnd_X	A	] <ctg1 >: 329]A	6	PASS	SVTYPE=BND
<ctg1 >	329	bnd_Y	T	T[<ctg1 >: 1[	6	PASS	SVTYPE=BND

5.4.3 Multiple mates

If a breakend has multiple mates such as in Figure 4 (either because of breakend reuse or of uncertainty in the measurement), these alternate adjacencies are treated as alternate alleles:

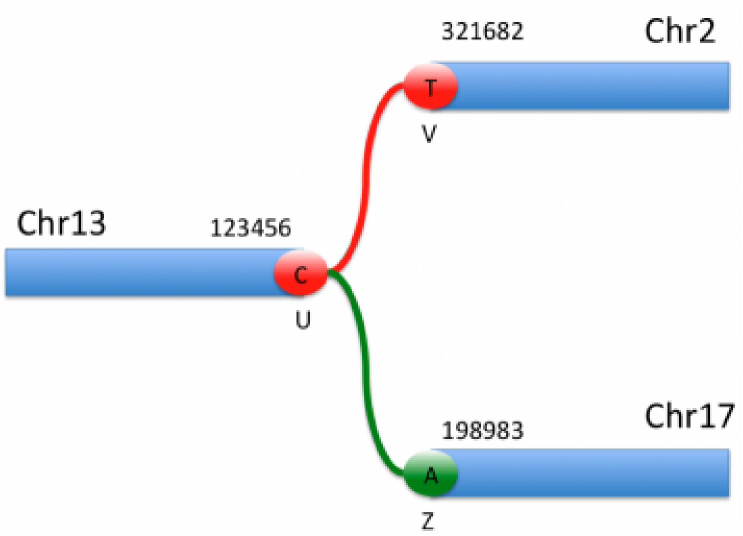

Figure 4: Breakend with multiple mates

#CHROM	POS	ID	REF	ALT	QUAL	FILTER	INFO
2	321682	bnd_V	T	]13 : 123456]T	6	PASS	SVTYPE=BND;MATEID=bnd_U
13	123456	bnd_U	C	C[2 : 321682[,C[17 : 198983[	6	PASS	SVTYPE=BND;MATEID=bnd_V,bnd_Z
17	198983	bnd_Z	A	]13 : 123456]A	6	PASS	SVTYPE=BND;MATEID=bnd_U

5.4.4 Explicit partners

Two breakends which are connected in the reference genome but disconnected in the variants are called partners. Each breakend only has one partner, typically one basepair left or right. However, it is not uncommon to observe

loss of a few basepairs during the rearrangement. It is then possible to explicitly name a breakend's partner, such as in Figure 5.:

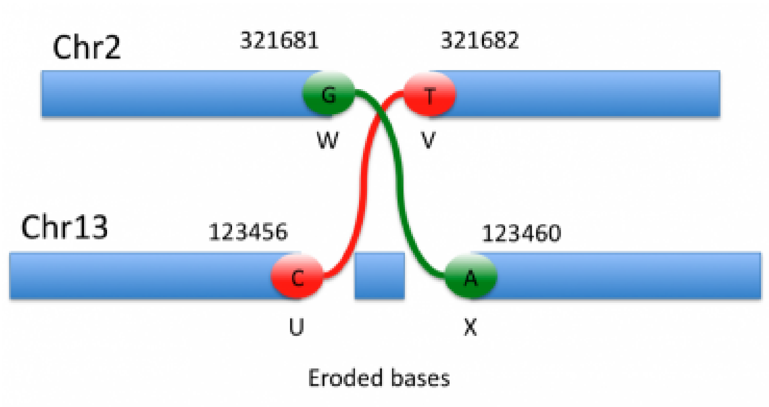

Figure 5: Partner breakends

#CHROM	POS	ID	REF	ALT	QUAL	FILTER	INFO
2	321681	bnd_W	G	G[13 : 123460[	6	PASS	PARID=bnd_V;MATEID=bnd_X
2	321682	bnd_V	T	]13 : 123456]T	6	PASS	PARID=bnd_W;MATEID=bnd_U
13	123456	bnd_U	C	C[2 : 321682[	6	PASS	PARID=bnd_X;MATEID=bnd_V
13	123460	bnd_X	A	]2 : 321681]A	6	PASS	PARID=bnd_U;MATEID=bnd_W

5.4.5 Telomeres

For a rearrangement involving the telomere end of a reference chromosome, we define a virtual telomeric breakend that serves as a breakend partner for the breakend at the telomere. That way every breakend has a partner. If the chromosome extends from position 1 to N, then the virtual telomeric breakends are at positions 0 and N+1. For example, to describe the reciprocal translocation of the entire chromosome 1 into chromosome 13, as illustrated in Figure 6:

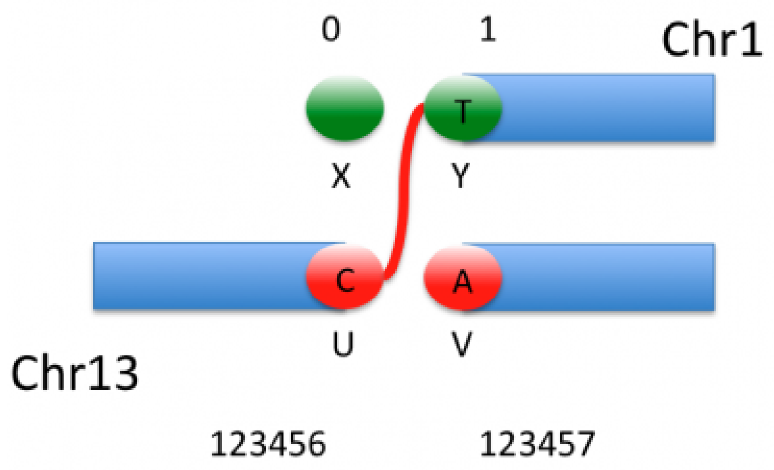

Figure 6: Telomeres

the records would look like:

#CHROM	POS	ID	REF	ALT	QUAL	FILTER	INFO
1	0	bnd_X	N	.[13 : 123457[	6	PASS	SVTYPE=BND;MATEID=bnd_V
1	1	bnd_Y	T	]13 : 123456]T	6	PASS	SVTYPE=BND;MATEID=bnd_U
13	123456	bnd_U	C	C[1 : 1[	6	PASS	SVTYPE=BND;MATEID=bnd_Y
13	123457	bnd_V	A	]1 : 0]A	6	PASS	SVTYPE=BND;MATEID=bnd_X

5.4.6 Event modifiers

As mentioned previously, a single rearrangement event can be described as a set of novel adjacencies. For example, a reciprocal rearrangement such as in Figure 7:

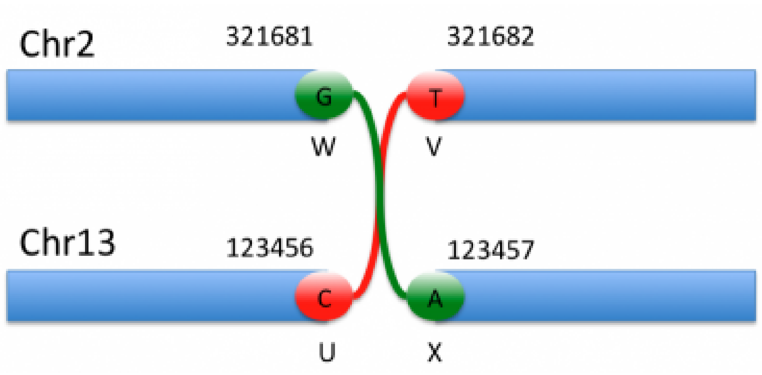

Figure 7: Rearrangements

would be described as:

#CHROM	POS	ID	REF	ALT	QUAL	FILTER	INFO
2	321681	bnd_W	G	G[13 : 123457[	6	PASS	SVTYPE=BND;MATEID=bnd_X;EVENT=RR0
2	321682	bnd_V	T	]13 : 123456]T	6	PASS	SVTYPE=BND;MATEID=bnd_U;EVENT=RR0
13	123456	bnd_U	C	C[2 : 321682[	6	PASS	SVTYPE=BND;MATEID=bnd_V;EVENT=RR0
13	123457	bnd_X	A	]2 : 321681]A	6	PASS	SVTYPE=BND;MATEID=bnd_W;EVENT=RR0

5.4.7 Inversions

Similarly an inversion such as in Figure 8:

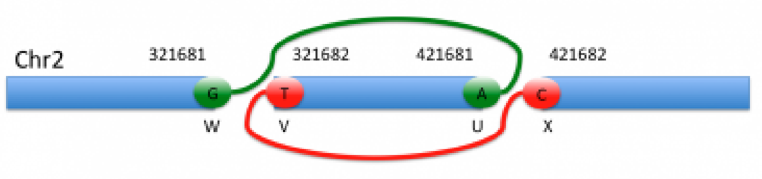

Figure 8: Inversion

can be described equivalently in two ways. Either one uses the short hand notation described previously (recommended for simple cases):

#CHROM	POS	ID	REF	ALT	QUAL	FILTER	INFO
2	321682	INV0	T	<INV>	6	PASS	SVTYPE=INV;END=421681

or one describes the breakends:

#CHROM	POS	ID	REF	ALT	QUAL	FILTER	INFO
2	321681	bnd_W	G	G]2 : 421681]	6	PASS	SVTYPE=BND;MATEID=bnd_U;EVENT=INV0
2	321682	bnd_V	T	[2 : 421682[T	6	PASS	SVTYPE=BND;MATEID=bnd_X;EVENT=INV0
2	421681	bnd_U	A	A]2 : 321681]	6	PASS	SVTYPE=BND;MATEID=bnd_W;EVENT=INV0
2	421682	bnd_X	C	[2 : 321682[C	6	PASS	SVTYPE=BND;MATEID=bnd_V;EVENT=INV0

5.4.8 Uncertainty around breakend location

It sometimes is difficult to determine the exact position of a break, generally because of homologies between the sequences being modified, such as in Figure 9. The breakend is then placed arbitrarily at the left most position, and the uncertainty is represented with the CIPOS tag. The ALT string is then constructed assuming this arbitrary breakend choice.

The figure above represents a nonreciprocal translocation with microhomology. Even if we know that breakend U is rearranged with breakend V, actually placing these breaks can be extremely difficult. The red and green dashed

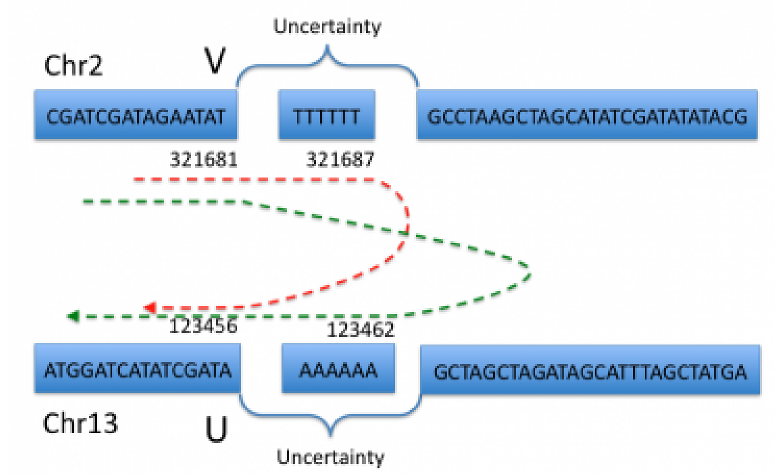

Figure 9: Homology

lines represent the most extreme possible recombination events which are allowed by the sequence evidence available. We therefore place both U and V arbitrarily within the interval of possibility:

#CHROM	POS	ID	REF	ALT	QUAL	FILTER	INFO
2	321681	bnd_V	T	T]13 : 123462]	6	PASS	SVTYPE=BND;MATEID=bnd_U;CIPOS=0,6
13	123456	bnd_U	A	A]2 : 321687]	6	PASS	SVTYPE=BND;MATEID=bnd_V;CIPOS=0,6

Note that the coordinate in breakend U's ALT string does not correspond to the designated position of breakend V, but to the position that V would take if U's position were fixed (and vice-versa). The CIPOS tags describe the uncertainty around the positions of U and V.

The fact that breakends U and V are mates is preserved thanks to the MATEID tags. If this were a reciprocal translocation, then there would be additional breakends X and Y, say with X the partner of V on Chr 2 and Y the partner of U on Chr 13, and there would be two more lines of VCF for the XY novel adjacency. Depending on which positions are chosen for the breakends X and Y, it might not be obvious that X is the partner of V and Y is the partner of U from their locations alone. This partner relation ship can be specified explicitly with the tag PARID=bnd_X in the VCF line for breakend V and PARID=bnd_Y in the VCF line for breakend U, and vice versa.

5.4.9 Single breakends

We allow for the definition of a breakend that is not part of a novel adjacency, also identified by the tag SVTYPE=BND. We call these single breakends, because they lack a mate. Breakends that are unobserved partners of breakends in observed novel adjacencies are one kind of single breakend. For example, if the true situation is known to be either as depicted back in Figure 1, and we only observe the adjacency (U,V), and no adjacencies for W, X, Y, or Z, then we cannot be sure whether we have a simple reciprocal translocation or a more complex 3-break operation. Yet we know the partner X of U and the partner W of V exist and are breakends. In this case we can specify these as single breakends, with unknown mates. The 4 lines of VCF representing this situation would be:

#CHROM	POS	ID	REF	ALT	QUAL	FILTER	INFO
2	321681	bnd_W	G	G.	6	PASS	SVTYPE=BND
2	321682	bnd_V	T	]13 : 123456]T	6	PASS	SVTYPE=BND;MATEID=bnd_U
13	123456	bnd_U	C	C[2 : 321682[	6	PASS	SVTYPE=BND;MATEID=bnd_V
13	123457	bnd_X	A	.A	6	PASS	SVTYPE=BND

On the other hand, if we know a simple reciprocal translocation has occurred as in Figure 7, then even if we have no evidence for the (W,X) adjacency, for accounting purposes an adjacency between W and X may also be recorded in the VCF file. These two breakends W and X can still be crossed-referenced as mates. The 4 VCF records describing this situation would look exactly as below, but perhaps with a special quality or filter value for the breakends W and X.

Another possible reason for calling single breakends is an observed but unexplained change in copy number along a chromosome.

#CHROM	POS	ID	REF	ALT	QUAL	FILTER	INFO
3	12665	bnd_X	A	.A	6	PASS	SVTYPE=BND;CIPOS=-50,50
3	12665	.	A	<DUP>	14	PASS	SVTYPE=DUP;END=13686;CIPOS=-50,50;CIEND=-50,50
3	13686	bnd_Y	T	T.	6	PASS	SVTYPE=BND;CIPOS=-50,50

Finally, if an insertion is detected but only the first few base-pairs provided by overhanging reads could be assembled, then this inserted sequence can be provided on that line, in analogy to paired breakends:

#CHROM	POS	ID	REF	ALT	QUAL	FILTER	INFO
3	12665	bnd_X	A	.TGCA	6	PASS	SVTYPE=BND;CIPOS=-50,50
3	12665	.	A	<DUP>	14	PASS	SVTYPE=DUP;END=13686;CIPOS=-50,50;CIEND=-50,50
3	13686	bnd_Y	T	TCC.	6	PASS	SVTYPE=BND;CIPOS=-50,50

5.4.10 Sample mixtures

It may be extremely difficult to obtain clinically perfect samples, with only one type of cell. Let's imagine that two samples are taken from a cancer patient: healthy blood, and some tumor tissue with an estimated 30% stromal contamination. This would then be expressed in the header as:

```
##SAMPLE=<ID=Blood,Genomes=Germline,Mixture=1.,Description="Patient germline genome">
##SAMPLE=<ID=TissueSample,Genomes=Germline;Tumor,Mixture=.3;.7,Description="Patient germline genome;Patient tumor genome">
```

Because of this distinction between sample and genome, it is possible to express the data along both distinctions. For example, in a first pass, a structural variant caller would simply report counts per sample. Using the example of the inversion just above, the VCF code could become:

<table>
<tr><th>#CHROM</th><th>POS</th><th>ID</th><th>REF</th><th>ALT</th><th>QUAL</th><th>FILTER</th><th>INFO</th><th>FORMAT</th><th>Blood</th><th>TissueSample</th></tr>
<tr><td>2</td><td>321681</td><td>bnd_W</td><td>G</td><td>G]2 : 421681]</td><td>6</td><td>PASS</td><td>SVTYPE=BND;MATEID=bnd_U</td><td>GT:DPADJ</td><td>0:32</td><td>0 | 1 : 9 | 21</td></tr>
<tr><td>2</td><td>321682</td><td>bnd_V</td><td>T</td><td>[2 : 421682[T</td><td>6</td><td>PASS</td><td>SVTYPE=BND;MATEID=bnd_X</td><td>GT:DPADJ</td><td>0:29</td><td>0 | 1 : 11 | 25</td></tr>
<tr><td>13</td><td>421681</td><td>bnd_U</td><td>A</td><td>A]2 : 321681]</td><td>6</td><td>PASS</td><td>SVTYPE=BND;MATEID=bnd_W</td><td>GT:DPADJ</td><td>0:34</td><td>0 | 1 : 10 | 23</td></tr>
<tr><td>13</td><td>421682</td><td>bnd_X</td><td>C</td><td>[2 : 321682[C</td><td>6</td><td>PASS</td><td>SVTYPE=BND;MATEID=bnd_V</td><td>GT:DPADJ</td><td>0:31</td><td>0 | 1 : 8 | 20</td></tr>
</table>

However, a more evolved algorithm could attempt actually deconvolving the two genomes and generating copy number estimates based on the raw data:

#CHROM	POS	ID	REF	ALT	QUAL	FILTER	INFO	FORMAT	Blood	TumorSample
2	321681	bnd_W	G	G]2 : 421681]	6	PASS	SVTYPE=BND;MATEID=bnd_U	GT:CNADJ	0:1	1:1
2	321682	bnd_V	T	[2 : 421682[T	6	PASS	SVTYPE=BND;MATEID=bnd_X	GT:CNADJ	0:1	1:1
13	421681	bnd_U	A	A]2 : 321681]	6	PASS	SVTYPE=BND;MATEID=bnd_W	GT:CNADJ	0:1	1:1
13	421682	bnd_X	C	[2 : 321682[C	6	PASS	SVTYPE=BND;MATEID=bnd_V	GT:CNADJ	0:1	1:1

5.4.11 Clonal derivation relationships

In cancer, each VCF file represents several genomes from a patient, but one genome is special in that it represents the germline genome of the patient. This genome is contrasted to a second genome, the cancer tumor genome. In the simplest case the VCF file for a single patient contains only these two genomes. This is assumed in most of the discussion of the sections below.

In general there may be several tumor genomes from the same patient in the VCF file. Some of these may be secondary tumors derived from an original primary tumor. We suggest the derivation relationships between genomes in a cancer VCF file be represented in the header with PEDIGREE tags.

Analogously, there might also be several normal genomes from the same patient in the VCF (typically double normal studies with blood and solid tissue samples). These normal genomes are then considered to be derived from the original germline genome, which has to be inferred by parsimony.

The general format of a PEDIGREE line describing asexual, clonal derivation is:

```
PEDIGREE=<Derived=ID2,Original=ID1>
```

This line asserts that the DNA in genome is asexually or clonally derived with mutations from the DNA in genome . This is the asexual analog of the VCF format that has been proposed for family relationships between genomes, i.e. there is one entry per of the form:

```
PEDIGREE=<Child=CHILD-GENOME-ID,Mother=MOTHER-GENOME-ID,Father=FATHER-GENOME-ID>
```

Let's consider a cancer patient VCF file with 4 genomes: germline, primary_tumor, secondary_tumor1, and secondary_tumor2 as illustrated in Figure 10. The primary_tumor is derived from the germline and the secondary tumors are each derived independently from the primary tumor, in all cases by clonal derivation with mutations. The PEDIGREE lines would look like:

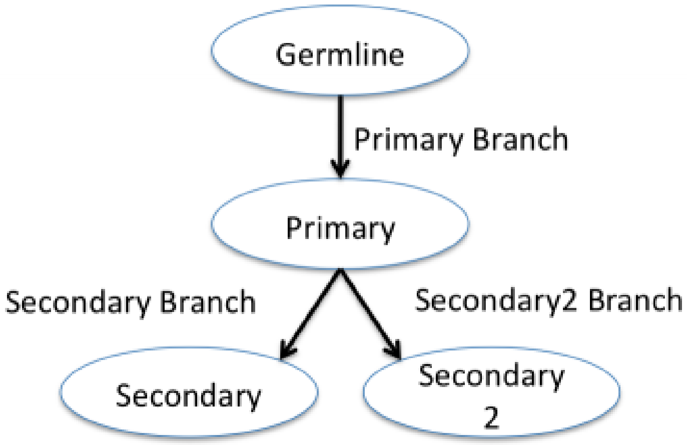

Figure 10: Pegigree example

```
##PEDIGREE=<Derived=PRIMARY-TUMOR-GENOME-ID,Original=GERMLINE-GENOME-ID>
##PEDIGREE=<Derived=SECONDARY1-TUMOR-GENOME-ID,Original=PRIMARY-TUMOR-GENOME-ID>
##PEDIGREE=<Derived=SECONDARY2-TUMOR-GENOME-ID,Original=PRIMARY-TUMOR-GENOME-ID>
```

Alternately, if data on the genomes is compiled in a database, a simple pointer can be provided:

```
##pedigreeDB=<url>
```

The most general form of a pedigree line is:

```
##PEDIGREE=<Name_0=G0-ID,Name_1=G1-ID,...,Name_N=GN-ID>
```

This means that the genome Name_0 is derived from the N $\geq$ 1 genomes Name_1, ..., Name_N. Based on these derivation relationships two new pieces of information can be specified.

Firstly, we wish to express the knowledge that a variant is novel to a genome, with respect to its parent genome. Ideally, this could be derived by simply comparing the features on either genomes. However, insufficient data or sample mixtures might prevent us from clearly determining at which stage a given variant appeared. This would be represented by a mutation quality score.

Secondly, we define a **haplotype** as a set of variants which are known to be on the same chromosome in the germline genome. Haplotype identifiers must be unique across the germline genome, and are conserved along clonal lineages, regardless of mutations, rearrangements, or recombination. In the case of the duplication of a region within a haplotype, one copy retains the original haplotype identifier, and the others are considered to be novel haplotypes with their own unique identifiers. All these novel haplotypes have in common their **haplotype ancestor** in the parent genome.

5.4.12 Phasing adjacencies in an aneuploid context

In a cancer genome, due to duplication followed by mutation, there can in principle exist any number of haplotypes in the sampled genome for a given location in the reference genome. We assume each haplotype that the user chooses to name is named with a numerical haplotype identifier. Although it is difficult with current technologies to associate haplotypes with novel adjacencies, it might be partially possible to deconvolve these connections in the near future. We therefore propose the following notation to allow haplotype-ambiguous as well as haplotype-unambiguous connections to be described. The general term for these haplotype-specific adjacencies is **bundles**.

The diagram in Figure 11 will be used to support examples below:

In this example, we know that in the sampled genome:

1. A reference bundle connects breakend U, haplotype 5 on chr13 to its partner, breakend X, haplotype 5 on chr13,

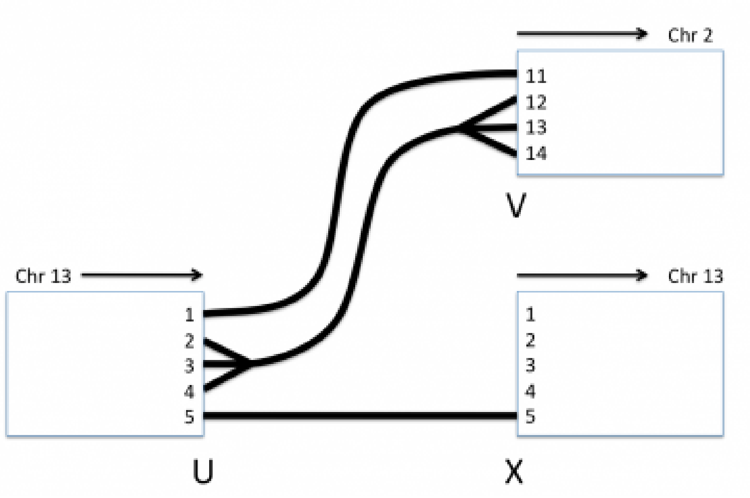

Figure 11: Phasing

2. A novel bundle connects breakend U, haplotype 1 on chr13 to its mate breakend V, haplotype 11 on chr2, and finally,

3. A novel bundle connects breakend U, haplotypes 2, 3 and 4 on chr13 to breakend V, haplotypes 12, 13 or 14 on chr2 without any explicit pairing.

These three are the bundles for breakend U. Each such bundle is referred to as a haplotype of the breakend U. Each allele of a breakend corresponds to one or more haplotypes. In the above case there are two alleles: the 0 allele, corresponding to the adjacency to the partner X, which has haplotype (1), and the 1 allele, corresponding to the two haplotypes (2) and (3) with adjacency to the mate V.

For each haplotype of a breakend, say the haplotype (2) of breakend U above, connecting the end of haplotype 1 on a segment of Chr 13 to a mate on Chr 2 with haplotype 11, in addition to the list of haplotype-specific adjacencies that define it, we can also specify in VCF several other quantities. These include:

1. The depth of reads on the segment where the breakend occurs that support the haplotype, e.g. the depth of reads supporting haplotype 1 in the segment containing breakend U

2. The estimated copy number of the haplotype on the segment where the breakend occurs

3. The depth of paired-end or split reads that support the haplotype-specific adjacencies, e.g. that support the adjacency between haplotype 1 on Chr 13 to haplotype 11 on Chr 2

4. The estimated copy number of the haplotype-specific adjacencies

5. An overall quality score indicating how confident we are in this asserted haplotype

These are specified using the using the DP, CN, BDP, BCN, and HQ subfields, respectively. The total information available about the three haplotypes of breakend U in the figure above may be visualized in a table as follows.

Allele	1	1	0
Haplotype	1>11	2,3,4>12,13,14	5>5
Segment Depth	5	17	4
Segment Copy Number	1	3	1
Bundle Depth	4	0	3
Bundle Copy Number	1	3	1
Haplotype quality	30	40	40

6 BCF specification

VCF is very expressive, accommodates multiple samples, and is widely used in the community. Its biggest drawback is that it is big and slow. Files are text and therefore require a lot of space on disk. A normal batch of 1̃00 exomes is a few GB, but large-scale VCFs with thousands of exome samples quickly become hundreds of GBs. Because the file is text, it is extremely slow to parse.

Overall, the idea behind is BCF2 is simple. BCF2 is a binary, compressed equivalent of VCF that can be indexed with tabix and can be efficiently decoded from disk or streams. For efficiency reasons BCF2 only supports a subset of VCF, in that all info and genotype fields must have their full types specified. That is, BCF2 requires that if e.g. an info field `AC` is present then it must contain an equivalent VCF header line noting that `AC` is an allele indexed array of type integer.

6.1 Overall file organization

A BCF2 file is composed of a mandatory header, followed by a series of BGZF compressed blocks of binary BCF2 records. The BGZF blocks allow BCF2 files to be indexed with tabix.

BGZF blocks are composed of a VCF header with a few additional records and a block of records. Following the last BGZF BCF2 record block is an empty BGZF block (a block containing zero type of data), indicating that the records are done.

A BCF2 header follows exactly the specification as VCF, with a few extensions/restrictions:

- All BCF2 files must have fully specified contigs definitions. No record may refer to a contig not present in the header itself.
- All INFO and GENOTYPE fields must be fully typed in the BCF2 header to enable type-specific encoding of the fields in records. An error should be thrown when converting a VCF to BCF2 when an unknown or not fully specified field is encountered in the records.

6.2 Header

The BCF2 header begins with the "BCF2 magic" 5 bytes that encode `BCF`*XY* where *X* and *Y* are bytes indicating the major number (currently 2) and the minor number (currently 1). This magic can be used to quickly examine the file to determine that it's a BCF2 file. Immediately following the BCF2 magic is the standard VCF header lines in text format, beginning with `##fileformat=VCFvX.Y`. Because the type is encoded directly in the header, the recommended extension for BCF2 formatted files is *.bcf*. BCF2 supports encoding values in a dictionary of strings. The string map is provided by the keyword `##dictionary=S0,S1,...,SN` as a comma-separate ordered list of strings. See the "Dictionary of strings" section for more details.

6.2.1 Dictionary of strings

Throughout the BCF file most string values are be specified by integer reference to their dictionary values. For example, the following VCF record:

```
##INFO=<ID=ASP,Number=0,Type=Flag,Description="X">
##INFO=<ID=RSPOS,Number=1,Type=Integer,Description="Y">
##INFO=<ID=dbSNPBuildID,Number=1,Type=Integer,Description="Z">
##contig=<ID=20,length=62435964,assembly=B36,md5=f126cdf8a6e0c7f379d618ff66beb2da,species="Homo sapiens">
#CHROM POS ID REF ALT QUAL FILTER INFO
20 10144 rs144773400 TA T . PASS ASP;RSPOS=10145,dbSNPBuildID=134
20 10228 rs143255646 TA T . PASS ASP;RSPOS=10229;dbSNPBuildID=134
```

would be encoded inline in BCF2 by reference to the relative position of the header line in the header (ASP=1, RSPOS=2, dbSNPBuildID=3, and PASS implicitly encoded in the last offset PASS=4)

```
##INFO=<ID=ASP,Number=0,Type=Flag,Description="X">
##INFO=<ID=RSPOS,Number=1,Type=Integer,Description="Y">
##INFO=<ID=dbSNPBuildID,Number=1,Type=Integer,Description="Z">
##contig=<ID=20,length=62435964,assembly=B36,md5=f126cdf8a6e0c7f379d618ff66beb2da,species="Homo sapiens">
#CHROM POS ID REF ALT QUAL FILTER INFO
0 10144 rs144773400 TA T . s0 s1;s2=10145;s3=134
0 10228 rs143255646 TA T . s0 s1;s2=10229;s3=134
```

Note that the dictionary encoding has the magic prefix 's' here to indicate that the field's value is actually in the dictionary entry giving by the subsequent offset. This representation isn't actually the one used in BCF2 records but it provides a clean visual guide for the above example. Note also how the contig has been recoded as a offset into the list of contig declarations.

Note that "PASS" is always implicitly encoded as the first entry in the header dictionary. This is because VCF allows FILTER fields to be PASS without explicitly listing this in the FILTER field itself.

6.2.2 Dictionary of contigs

The CHROM field in BCF2 is encoded as an integer offset into the list of `##contig` field headers in the VCF header. The offsets begin, like the dictionary of strings, at 0. So for example if in BCF2 the contig value is 10, this indicates that the actual chromosome is the 11th element in the ordered list of `##contig` elements. Here's a more concrete example:

```
##contig=<ID=20,length=62435964,assembly=B36,md5=f126cdf8a6e0c7f379d618ff66beb2da,species="Homo sapiens">
##contig=<ID=21,length=62435964,assembly=B36,md5=f126cdf8a6e0c7f379d618ff66beb2da,species="Homo sapiens">
##contig=<ID=22,length=62435964,assembly=B36,md5=f126cdf8a6e0c7f379d618ff66beb2da,species="Homo sapiens">
#CHROM POS ID REF ALT QUAL FILTER INFO
20 1 . T A . PASS .
21 2 . T A . PASS .
22 3 . T A . PASS .
```

the actual CHROM field values in the encoded BCF2 records would be 0, 1, and 2 corresponding to the first (offset 0) `##contig` element, etc.

6.3 BCF2 records

In BCF2, the original VCF records are converted to binary and encoded as BGZF blocks. Each record is conceptually two parts. First is the site information (chr, pos, INFO field). Immediately after the sites data is the genotype data for every sample in the BCF2 file. The genotype data may be omitted entirely from the record if there is no genotype data in the VCF file. Note that it's acceptable to not BGZF compress a BCF2 file, but not all readers may handle this uncompressed encoding.

6.3.1 Site encoding

BCF2 site information encoding Field Type Notes l_shared uint32_t Data length from CHROM to the end of INFO l_indiv uint32_t Data length of FORMAT and individual genotype fields CHROM int32_t Given as an offset into the mandatory contig dictionary POS int32_t 0-based leftmost coordinate rlen int32_t Length of the record as projected onto the reference sequence. May be the actual length of the REF allele but for symbolic alleles should be the declared length respecting the END attribute n_allele_info int32_t n_info, where n_allele is the number of REF+ALT alleles in this record, and n_info is the number of VCF INFO fields present in this record n_fmt_sample uint32_t n_sample, where n_fmt is the number of format fields for genotypes in this record, and n_samples is the number of samples present in this sample. Note that the number of samples must be equal to the number of samples in the header QUAL float Variant quality; 0x7F800001 for a missing value ID typed string REF+ALT list of n_allele typed strings the first allele is REF (mandatory) followed by n_alleles - 1 ALT alleles, all encoded as typed strings FILTER Typed vector of integers a vector of integer offsets into dictionary, one for each FILTER field value. "." is encoded as MISSING INFO field key/value pairs n_info pairs of typed vectors The first value must be a typed atomic integer giving the offset of the INFO field key into the dictionary. The second value is a typed vector giving the value of the field Genotype value block see below see below

6.3.2 Genotype encoding

Genotype fields are encoded not by sample as in VCF but rather by field, with a vector of values for each sample following each field. In BCF2, the following VCF line:

FORMAT	NA00001	NA00002	NA00003
GT:GQ:DP	0/0:48:1	0/1:9:8	1/1:43:5

would encoded as the equivalent of:

GT=0/0,0/1,1/1   GQ=48,9,43   DP=1,8,5

Suppose there are i genotype fields in a specific record. Each i is encoded by a triplet:
BCF2 site information encoding

Field	Type	Notes
fmt_key	typed int	Format key as an offset into the dictionary
fmt_type	uint8_t+	Typing byte of each individual value, possibly followed by a typed int for the vector length. In effect this is the same as the typing value for a single vector, but for genotype values it appears only once before the array of genotype field values
fmt_values (by fmt type)	Array of values	The information of each individual is concatenated in the vector. Every value is of the same fmt type. Variable-length vectors are padded with missing values; a string is stored as a vector of char

The value is always implicitly a vector of N values, where N is the number of samples. The type byte of the value field indicates the type of each value of the N length vector. For atomic values this is straightforward (size = 1). But if the type field indicates that the values are themselves vectors (as often occurs, such as with the PL field) then each of the N values in the outer vector is itself a vector of values. This encoding is efficient when every value in the genotype field vector has the same length and type.

Note that the specific order of fields isn't defined, but it's probably a good idea to respect the ordering as specified in the input VCF/BCF2 file.

If there are no sample records (genotype data) in this VCF/BCF2 file, the size of the genotypes block will be 0.

6.3.3 Type encoding

In BCF2 values are all strongly typed in the file. The type information is encoded in a prefix byte before the value, which contains information about the low-level type of the value(s) such as int32 or float, as well as the number of elements in the value. The encoding is as follows:

BCF2 type descriptor byte

Bit	Meaning
5,6,7,8 bits	The number of elements of the upcoming type. For atomic values, the size must be 1. If the size is set to 15, this indicates that the vector has 15 or more elements, and that the subsequent BCF2 byte stream contains a typed Integer indicating the true size of the vector. If the size is between 2-14, then this Integer is omitted from the stream and the upcoming stream begins immediately with the first value of the vector. A size of 0 indicates that the value is MISSING.
1,2,3,4 bits	Type

The final four bits encodes an unsigned integer that indicates the type of the upcoming value in the data stream.
BCF2 types

Lowest 4 bits	Hexadecimal encoding	Corresponding atomic type
1	0x?1	Integer [8 bit]
2	0x?2	Integer [16 bit]
3	0x?3	Integer [32 bit]
5	0x?5	Float [32 bit]
7	0x?7	Character, ASCII encoded in 8 bits

Note this is not used in BCF2, but its type is reserved in case this becomes necessary. In BCF2 characters are simply represented by strings with a single element 0,4,6,8-15 reserved for future use.

Integers may be encoded as 8, 16, or 32 bit values, in little-endian order. It is up to the encoder to determine the appropriate ranged value to use when writing the BCF2 file. For each integer size, the value with all bits set (0x80, 0x8000, 0x80000000) for 8, 16, and 32 bit values, respectively) indicates that the field is a missing value.

Floats are encoded as single-precision (32 bit) in the basic format defined by the IEEE-754-1985 standard. This is the standard representation for floating point numbers on modern computers, with direct support in programming languages like C and Java (see Java's Double class for example). BCF2 supports the full range of values from -Infinity to +Infinity, including NaN. BCF2 needs to represent missing values for single precision floating point numbers. This is accomplished by writing the NaN value as the quiet NaN (qNaN), while the MISSING value is encoded as a signaling NaN. From the NaN wikipedia entry, we have:

For example, a bit-wise example of a IEEE floating-point standard single precision (32-bit) NaN would be: s111 1111 1axx xxxx xxxx xxxx xxxx xxxx where s is the sign (most often ignored in applications), a determines the type of NaN, and x is an extra payload (most often ignored in applications). If a = 1, it is a quiet NaN; if a is zero and the payload is nonzero, then it is a signaling NaN.

A good way to understand these values is to play around with the IEEE encoder webiste.

BCF2 bit representation for floating point NaN and MISSING

Value	32-bit precision	Hexadecimal representation
NaN	0b0111 1111 1100 0000 0000 0000 0000 0000	0x7FC00000
MISSING	0b0111 1111 1000 0000 0000 0000 0000 0001	0x7F800001

Character values are not explicitly typed in BCF2. Instead, VCF Character values should be encoded by a single character string. As with Strings, UNICODE characters are not supported.

Flags values – which can only appear in INFO fields – in BCF2 should be encoded by any non-MISSING value. The recommended best practice is to encode the value as an 1-element INT8 (type 0x11) with value of 1 to indicate present. Because FLAG values can only be encoded in INFO fields, BCF2 provides no mechanism to encode FLAG values in genotypes, but could be easily extended to do so if allowed in a future VCF version.

String values have two basic encodings. For INFO, FORMAT, and FILTER keys these are encoded by integer offsets into the header dictionary. For string values, such as found in the ID, REF, ALT, INFO, and FORMAT fields, strings are encoded as typed array of ASCII encoded bytes. The array isn't terminated by a null byte. The length of the string is given by the length of the type descriptor.

Suppose you want to encode the string ACAC. First, we need the type descriptor byte, which is the string type 0x07 or'd with inline size (4) yielding the type byte of 0x40 — 0x07 = 0x47. Immediately following the type byte is the four byte ASCII encoding of "ACAC" 0x41 0x43 0x41 0x43. So the final encoding is:

0x47 0x41 0x43 0x41 0x43	String type with inline size of 4 followed by ACAC in ASCII

Suppose you want to encode the string MarkDePristoWorksAtTheBroad, a string of size 27. First, we need the type descriptor byte, which is the string type 0x07. Because the size exceeds the inline size (27 > 15) we set the size to overflow, yielding the type byte of 0xF0 — 0x07 = 0xF7. Immediately following the type byte is the typed size of 27, which we encode by the atomic INT8 value: 0x11 followed by the actual size 0x1B. Finally comes the actual bytes of the string: 0x4D 0x61 0x72 0x6B 0x44 0x65 0x50 0x72 0x69 0x73 0x74 0x6F 0x57 0x6F 0x72 0x6B 0x73 0x41 0x74 0x54 0x68 0x65 0x42 0x72 0x6F 0x61 0x64. So the final encoding is:

0xF7	string with overflow size
0x11 0x1B	overflow size encoded as INT8 with value 27
0x4D 0x61 0x72 0x6B 0x44 0x65 0x50 0x72 0x69 0x73 0x74 0x6F 0x57 0x6F 0x72 0x6B 0x73 0x41 0x74 0x54 0x68 0x65 0x42 0x72 0x6F 0x61 0x64	message in ASCII

Suppose you want to encode the missing value '.'. This is simply a string of size 0 = 0x07.

In VCF there are sometimes fields of type list of strings, such as a number field of unbounded size encoding the amino acid changes due to a mutation. Since BCF2 doesn't directly support vectors of strings (a vector of character is already a string) we collapse the list of strings into a single comma-separated string, encode it as a regular BCF2 vector of characters, and on reading explode it back into the list of strings. This works because strings in VCF cannot contain ' , ' (it's a field separator) and so we can safely use ' , ' to separate the individual strings. For efficiency reasons we put a comma at the start of the collapsed string, so that just the first character can be examined to determine if the string is collapsed.

To be concrete, suppose we have a info field around X=[A,B,C,D]. This is encoded in BCF2 as a single string ",A,B,C,D" of size 8, so it would have type byte 0x87 followed by the ASCII encoding 0x2C 0x41 0x2C 0x42 0x2C

0x43 0x2C 0x44.

Vectors — The BCF2 type byte may indicate that the upcoming data stream contains not a single value but a fixed length vector of values. The vector values occur in order (1st, 2nd, 3rd, etc) encoded as expected for the type declared in the vector's type byte. For example, a vector of 3 16-bit integers would be layed out as first the vector type byte, followed immediately by 3 2-byte values for each integer, including a total of 7 bytes.

Missing values in vectors are handled slightly differently from atomic values. There are two possibilities for missing values:

One (or more) of the values in the vector may be missing, but others in the vector are not. Here each value should be represented in the vector, and each corresponding BCF2 vector value either set to its present value or the type equivalent MISSING value. Alternatively the entire vector of values may be missing. In this case the correct encoding is as a type byte with size 0 and the appropriate type MISSING. Suppose we are encoding the record "AC=[1,2,3]" from the INFO field. The AC key is encoded in the standard way. This would be immediately followed by a typed 8-bit integer vector of size 3, which is encoded by the type descriptor 0x31. The type descriptor is immediately followed by the three 8-bit integer values: 0x01 0x02 0x03, for a grant total of 4 bytes: 0x31010203.

Suppose we are at a site with many alternative alleles so AC=[1,2,3,4,5,6,7,8,9,10,11,12,13,14,15,16]. Since there are 16 values, we have to use the long vector encoding. The type of this field is 8 bit integer with the size set to 15 to indicate that the size is the next stream value, so this has type of 0xF1. The next value in the stream is the size, as a typed 8-bit atomic integer: 0x11 with value 16 0x10. Each integer AC value is represented by it's value as a 8 bit integer. The grand total representation here is:

0xF1 0x01 0x10	8 bit integer vector with overflow size
0x01 0x02 0x03 0x04 0x05 0x06 0x07 0x08 0x09 0x0A 0x0B 0x0C 0x0D 0x0E 0x0F 0x10	1-16 as hexadecimal 8 bit integers

Suppose this INFO field contains the "AC=.", indicating that the AC field is missing from a record with two alt alleles. The correct representation is as the typed pair of AC followed by a MISSING vector of type 8-bit integer: 0x01.

Vectors of mixed length — In some cases genotype fields may be vectors whose length differs among samples. For example, some CNV call sets encode different numbers of genotype likelihoods for each sample, given the large number of potential copy number states, rather padding all samples to have the same number of fields. For example, one sample could have CN0:0,CN1:10 and another CN0:0,CN1:10,CN2:10. In the situation when a genotype field contain vector values of different lengths, these are represented in BCF2 by a vector of the maximum length per sample, with all values in the each vector aligned to the left, and MISSING values assigned to all values not present in the original vector. The BCF2 encoder / decoder must automatically add and remove these MISSING values from the vectors.

For example, suppose I have two samples, each with a FORMAT field X. Sample A has values [1], while sample B has [2,3]. In BCF2 this would be encoded as [1, MISSING] and [2, 3]. Diving into the complete details, suppose X is at offset 3 in the dictionary, which is encoded by the typed INT8 descriptor 0x11 followed by the value 0x03. Next we have the type of the each format field, which here is a 2 element INT8 vector: 0x21. Next we have the encoding for each sample, A = 0x01 0x80 followed by B = 0x02 0x03. All together we have:

0x11 0x03	X dictionary offset
0x21	each value is a 2 element INT8 value
0x01 0x80	A is [1, MISSING]
0x02 0x03	B is [2, 3]

Note that this means that it's illegal to encode a vector VCF field with missing values; the BCF2 codec should signal an error in this case.

A **Genotype (GT) field** is encoded in a typed integer vector (can be 8, 16, or even 32 bit if necessary) with the number of elements equal to the maximum ploidy among all samples at a site. For one individual, each integer in the vector is organized as $(allele + 1) << 1 \mid phased$ where allele is set to -1 if the allele in GT is a dot '.' (thus the higher bits are all 0). The vector is padded with missing values if the GT having fewer ploidy.

Examples:

0/1	in standard format $(0+1) << 1 \mid 0$ followed by $(1+1) << 1 \mid 0$	0x02 0x04
0/1, 1/1, and 0/0	three samples encoded consecutively	0x020404040202
0 | 1	$(1+1) << 1 \mid 1 = $ 0x05 preceded by the standard first byte value 0x04	0x0405
./.	where both alleles are missing	0x00 0x00
0	as a haploid it is represented by a single byte	0x02
1	as a haploid it is represented by a single byte	0x04
0/1/2	is tetraploid, with alleles	0x02 0x04 0x06
0/1 | 2	is tetraploid with a single phased allele	0x02 0x04 0x07
0 and 0/1	pad out the final allele for the haploid individual	0x04 0x80 0x02 0x04

The final example is something seen on chrX when we have a haploid male and a diploid female. The spare male allele is just assigned the missing value.

Misc. notes
A type byte value of 0x00 is an allowed special case meaning MISSING but without an explicit type provided.

6.4 Encoding a VCF record example

Let's encode a realistic (but made-up) VCF record. This is a A/C SNP in HM3 (not really) called in 3 samples. In this section we'll build up the BCF2 encoding for this record.

```
#CHROM POS ID REF ALT QUAL FILTER INFO FORMAT NA00001 NA00002 NA00003
chr1 101 rs123 A C 30.1 PASS HM3;AC=3;AN=6;AA=C GT:GQ:DP:AD:PL 0/0:10:32:32,0:0,10,100 0/1:10:48:32,16:10,0,100 1/1:10:64:0,64:100,10,0
```

6.4.1 Encoding CHROM and POS

First, let's assume that `chr1` is the second chromosome to appear in the contig list—right after `chrM` (`MT`). So its offset is 1. The `POS` BCF2 field value is 101 (obviously). Because these are both typed values in the BCF2 record, we encode both in their most compact 8-bit value form. The type byte for an atomic 8-bit integer is 0x11. The value for the contig offset is 1 = 0x01. The value 101 is encoded as the single byte 0x65. So in total these are represented as:

0x01000000	CHROM offset is at 1 in 32 bit little endian
0x64000000	POS in 0 base 32 bit little endian
0x01000000	rlen = 1 (it's just a SNP)

6.4.2 Encoding QUAL

The QUAL field value is 30.1, which we encode as an untyped single precision 32-bit float:

0x41 0xF0 0xCC 0xCD	QUAL = 30.1 as 32-bit float

6.4.3 Encoding ID

For ID type byte would is a 5-element string (type descriptor 0x59), which would then be followed by the five bytes for the string of `0x72 0x73 0x31 0x32 0x33`. The full encoding is:

0x59 0x72 0x73 0x31 0x32 0x33	ID

6.4.4 Encoding REF/ALT fields

We encode each of REF and ALT as typed strings, first REF followed immediately by ALT. Each is a 1 element string (0x19), which would then be followed by the single bytes for the bases of 0x43 and 0x41:

0x19 0x41	REF A
0x19 0x43	ALT C

Just for discussion, suppose instead that ALT was ALT=C,T. The only thing that could change is that there would be another typed string following immediately after C encoding 0x19 (1 element string) with the value of 0x54.

6.4.5 Encoding FILTER

"PASS" is implicitly encoded as the last entry in the header dictionary (see dictionary of strings). Here we encode the PASS FILTER field as a vector of size 1 of type 8-bit, which has type byte is 0x11. The value is the offset 0:

0x11 0x00	FILTER field PASS

6.4.6 Encoding the INFO fields

HM3;AC=3;AN=6;AA=C Let's assume that the header dictionary elements for HM3, AC, AN, and AA are at 80, 81, 82, and 83 respectively. All of these can be encoded by 1-element INT8 values (0x11), with associated hex values of 0x50, 0x51, 0x52, and 0x53 respectively.

First is HM3. The entry begins with the key: 0x11 0x50. Next we have a Flag value to indicate the field is present, represented as a 1 element INT8 value of 1. Altogether we have:

0x11 0x50 0x11 0x01	HM3 flag is present

Now let's encode the two atomic 8-bit integer fields AC and AN:

0x11 0x51	AC key
0x11 0x03	with value of 3
0x11 0x52	AN key
0x11 0x06	with value of 6

The ancestral allele (AA) tell us that among other primates the original allele is C, a Character here. Because we represent Characters as single element strings in BCF2 (0x19) with value 0x43 (C). So the entire key/value pair is:

0x11 0x51	AA key
0x19 0x43	with value of C

6.4.7 Encoding Genotypes

Continuing with our example:

FORMAT	NA00001	NA00002	NA00003
GT:GQ:DP:AD:PL	0/0:10:32:32,0:0,10,100	0/1:10:48:32,16:10,0,100	1/1:10:64:0,64:100,10,0

Here we have the specially encoded GT field. We have two integer fields GQ and DP. We have the AD field, which is a vector of 2 values per sample. And finally we have the PL field which is 3 values per sample. Let's say that the FORMAT keys for GT, GQ, DP, AD, and PL are at offsets 1, 2, 3, and 4, 5, respectively. Now let's encode each of the genotype fields in order of the VCF record (GT, GQ, DP, AD, and then PL):

GT triplet begins with the key: 0x1101. Next is the type of the field, which will be a 2-element (diploid) INT8 type: 0x21. This is followed by 3 2-byte arrays of values 0x0202 0x0204 0x0404 (see genotype encoding example for details). The final encoding is 0x1101 0x21 0x020202040404

GQ triplet begins with the key 0x1102. Because these values are small, we encode them as 8 bit atomic integers with type code 0x11. As each value is the same (10 = 0x0A) the GQ field is encoded as 0x1102 0x11 0x0A0A0A

DP almost identical to GQ. First is the 0x1103 key, followed by 3 8-bit atomic integers encoded as 0x11 (the type) 0x20 (DP=32), 0x30 (DP=48) and 0x40 (DP=64). So we have: 0x1103 0x11203040

AD is more complex. The key is simple, just like the others, with 0x1104. Because the AD field is a vector of 2 values for each genotype, the value of key/value pair a vector type. Because the integer values in each AD field of each sample are small they are encoded by 8 bit values. So the value type is = 0x21. For sample one there are two values: 32,0 which are 0x30 and 0x00. Samples two and three are 0x30 0x20 and 0x00 0x40 respectively. So ultimately this field is encoded as 0x1104 0x21 0x300030200040

PL is just like AD but with three values per sample. The key is 0x1105. Because the PL field is a vector of 3 values for each genotype, the value of key/value pair a vector type, and because the size is 3 it's encoded in the size field of the type. Again, because the integer values in each PL field of each sample are small they are encoded by 8 bit values. So the value type 0x31. For sample one there are three values: 0, 10, and 100 which are 0x00, 0x0A, and 0x64. Samples two and three have the same values but in a slightly different order. So ultimately the PL field is encoded as 0x1105 0x31 0x000A64 0x0A0064 0x640A00

So the genotype block contains:

0x1101 0x21 0x020202040404	GT
0x1102 0x11 0x0A0A0A	GQ
0x1103 0x11 0x203040	DP
0x1104 0x21 0x300030200040	AD
0x1105 0x31 0x000A640A0064640A00	PL

Putting it all together
We need to determine a few values before writing out the final block:
l_shared = 54 (Data length from CHROM to the end of INFO)
l_indiv = 42 (Data length of FORMAT and individual genotype fields)
n_allele_info = n_allele<< 16 |n_info = 2 << 16 | 4 = 0x00020004
n_fmt_samples = n_fmt<< 24 |n_sample = 5 << 24 | 3 = 0x05000003

0x36000000	l_shared as little endian hex
0x2A000000	l_indiv as little endian hex
0x01000000	CHROM offset is at 1 in 32 bit little endian
0x64000000	POS in 0 base 32 bit little endian
0x01000000	rlen = 1 (it's just a SNP)
0x41 0xF0 0xCC 0xCD	QUAL = 30.1 as 32-bit float
0x00020004	n_allele_info
0x05000003	n_fmt_samples
0x59 0x72 0x73 0x31 0x32 0x33	ID
0x19 0x41	REF A
0x19 0x43	ALT C
0x11 0x00	FILTER field PASS
0x11 0x50 0x11 0x01	HM3 flag is present
0x11 0x51	AC key
0x11 0x03	with value of 3
0x11 0x52	AN key
0x11 0x06	with value of 6
0x11 0x51	AA key
0x19 0x43	with value of C
0x1101 0x21 0x020202040404	GT
0x1102 0x11 0x0A0A0A	GQ
0x1103 0x11 0x203040	DP
0x1104 0x21 0x300030200040	AD
0x1105 0x31 0x000A640A0064640A00	PL

That's quite a lot of information encoded in only 96 bytes!

6.5 BCF2 block gzip and indexing

These raw binary records may be subsequently encoded into BGZF blocks following the BGZF compression format, section 3 of the SAM format specification. BCF2 records can be raw, though, in cases where the decoding/encoding costs of bgzipping the data make it reasonable to process the data uncompressed, such as streaming BCF2s through pipes with samtools and bcftools. Here the files should be still compressed with BGZF but with compression 0. Note that currently the GATK generates raw BCF2 files (not BGZF compression at all) but this will change in the near future.

BCF2 files are expected to be indexed through the same index scheme, section 4 as BAM files and other block-compressed files with BGZF.
