# Supplementary material for: The pos-1 3′ untranslated region governs germline specification and proliferation to ensure reproductive robustness
Source: PLoS Genet. 2026 Apr 27;22(4):e1012129. doi: 10.1371/journal.pgen.1012129 (PMC13132445; doi:10.1371/journal.pgen.1012129)
Supplement: S2 Data — (GZ) [file pgen.1012129.s008.gz › SupplementalDataSet1/03.Result_X202SC24112711-Z01-F001_C_elegans/result/05.InDel_VarDetect/picture_in_reports/WRM101.InDel.table.pdf]

WRM101

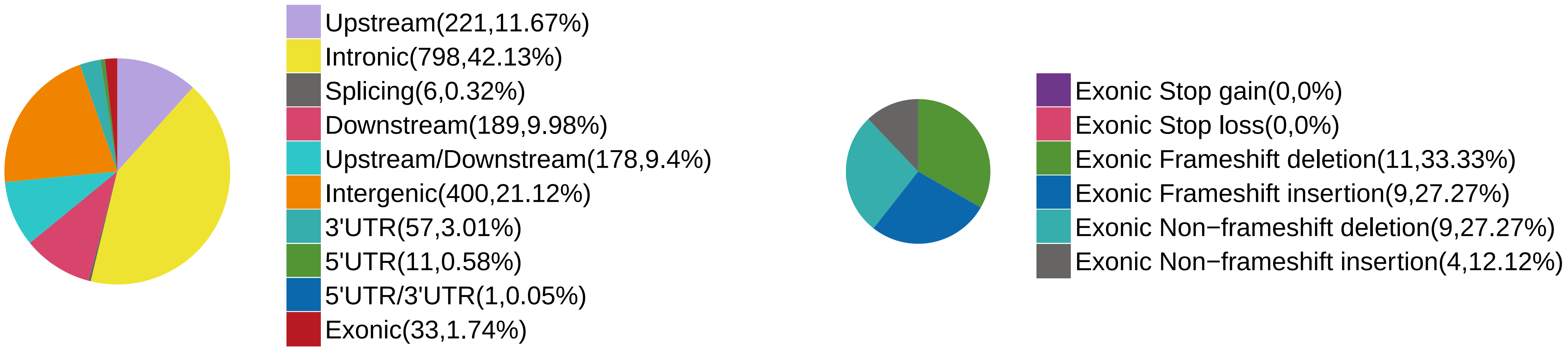
Upstream(221,11.67%)
Intronic(798,42.13%)
Splicing(6,0.32%)
Downstream(189,9.98%)
Upstream/Downstream(178,9.4%)
Intergenic(400,21.12%)
3'UTR(57,3.01%)
5'UTR(11,0.58%)
5'UTR/3'UTR(1,0.05%)
Exonic(33,1.74%)
Exonic Stop gain(0,0%)
Exonic Stop loss(0,0%)
Exonic Frameshift deletion(11,33.33%)
Exonic Frameshift insertion(9,27.27%)
Exonic Non−frameshift deletion(9,27.27%)
Exonic Non−frameshift insertion(4,12.12%)
