# Supplementary material for: The pos-1 3′ untranslated region governs germline specification and proliferation to ensure reproductive robustness
Source: PLoS Genet. 2026 Apr 27;22(4):e1012129. doi: 10.1371/journal.pgen.1012129 (PMC13132445; doi:10.1371/journal.pgen.1012129)
Supplement: S2 Data — (GZ) [file pgen.1012129.s008.gz › SupplementalDataSet1/03.Result_X202SC24112711-Z01-F001_C_elegans/result/05.InDel_VarDetect/picture_in_reports/WRM103.InDel.table.pdf]

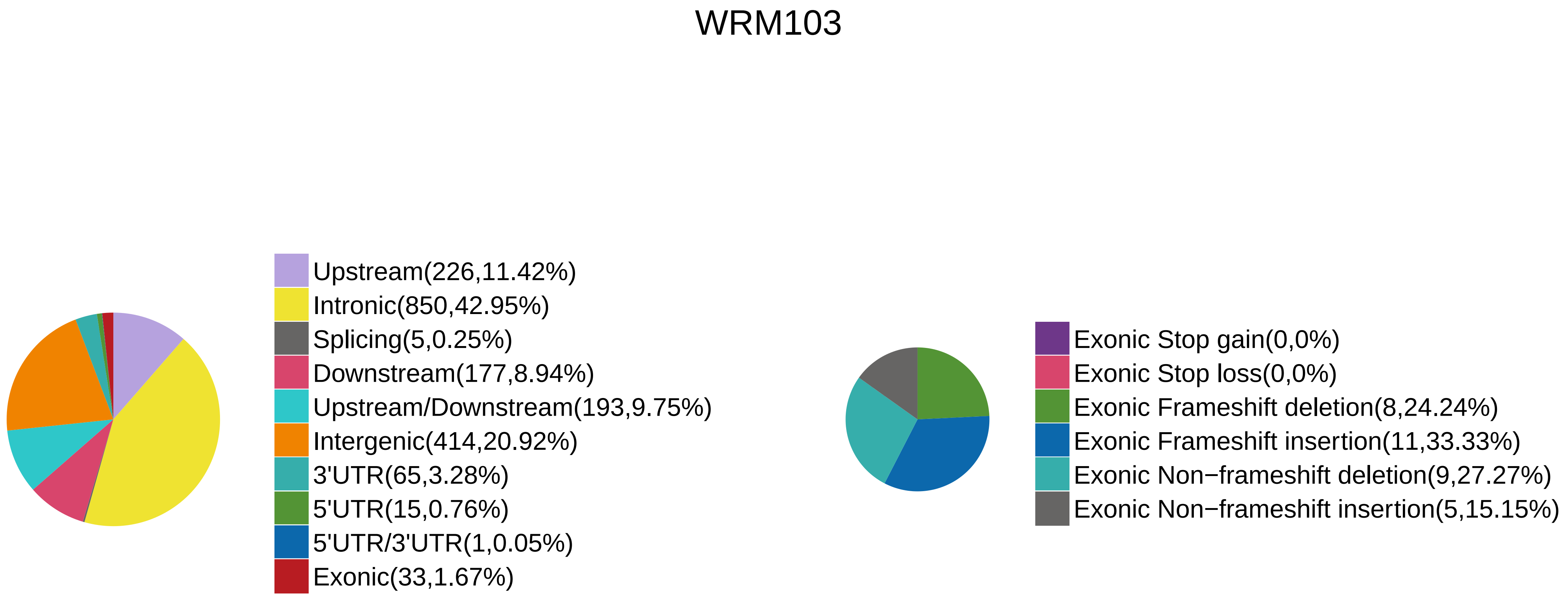
WRM103
Upstream(226,11.42%)
Intronic(850,42.95%)
Splicing(5,0.25%)
Downstream(177,8.94%)
Upstream/Downstream(193,9.75%)
Intergenic(414,20.92%)
3'UTR(65,3.28%)
5'UTR(15,0.76%)
5'UTR/3'UTR(1,0.05%)
Exonic(33,1.67%)
Exonic Stop gain(0,0%)
Exonic Stop loss(0,0%)
Exonic Frameshift deletion(8,24.24%)
Exonic Frameshift insertion(11,33.33%)
Exonic Non−frameshift deletion(9,27.27%)
Exonic Non−frameshift insertion(5,15.15%)
