# Supplementary material for: The pos-1 3′ untranslated region governs germline specification and proliferation to ensure reproductive robustness
Source: PLoS Genet. 2026 Apr 27;22(4):e1012129. doi: 10.1371/journal.pgen.1012129 (PMC13132445; doi:10.1371/journal.pgen.1012129)
Supplement: S2 Data — (GZ) [file pgen.1012129.s008.gz › SupplementalDataSet1/03.Result_X202SC24112711-Z01-F001_C_elegans/result/05.InDel_VarDetect/picture_in_reports/DG4222.InDel.table.pdf]

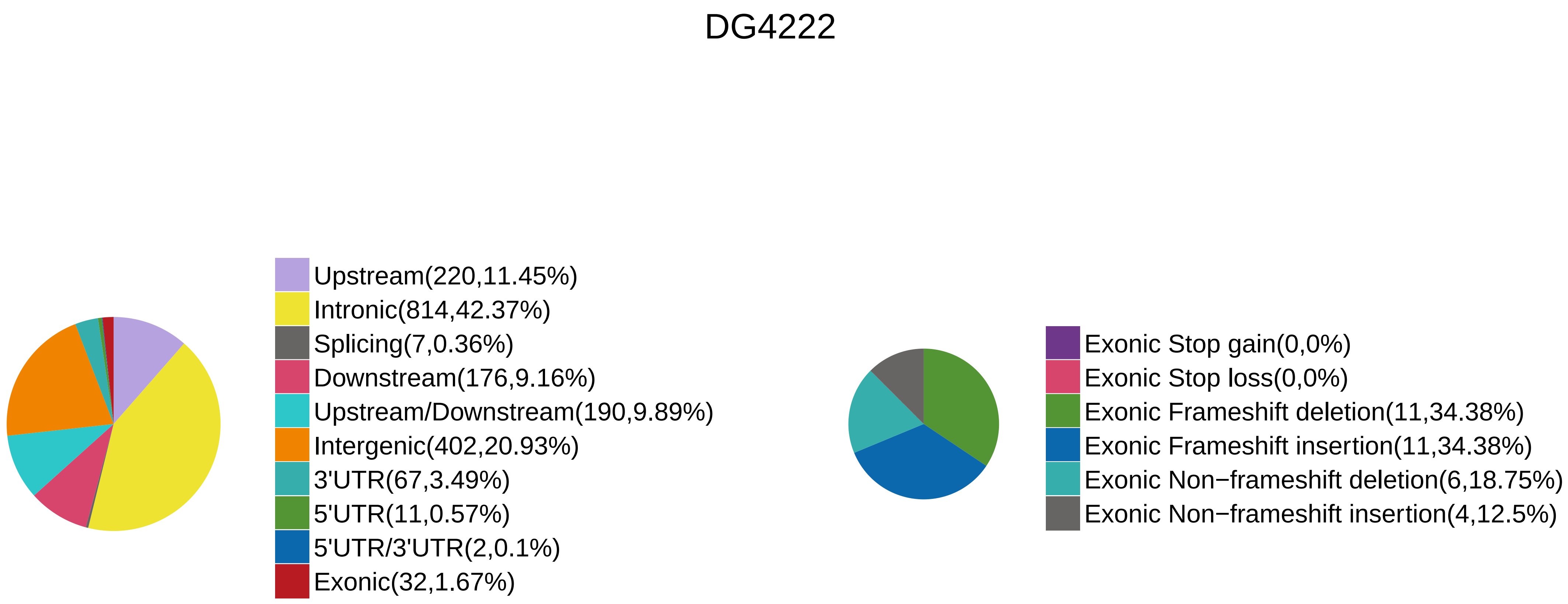
DG4222
Upstream(220,11.45%)
Intronic(814,42.37%)
Splicing(7,0.36%)
Downstream(176,9.16%)
Upstream/Downstream(190,9.89%)
Intergenic(402,20.93%)
3'UTR(67,3.49%)
5'UTR(11,0.57%)
5'UTR/3'UTR(2,0.1%)
Exonic(32,1.67%)
Exonic Stop gain(0,0%)
Exonic Stop loss(0,0%)
Exonic Frameshift deletion(11,34.38%)
Exonic Frameshift insertion(11,34.38%)
Exonic Non−frameshift deletion(6,18.75%)
Exonic Non−frameshift insertion(4,12.5%)
