# Supplementary material for: The pos-1 3′ untranslated region governs germline specification and proliferation to ensure reproductive robustness
Source: PLoS Genet. 2026 Apr 27;22(4):e1012129. doi: 10.1371/journal.pgen.1012129 (PMC13132445; doi:10.1371/journal.pgen.1012129)
Supplement: S2 Data — (GZ) [file pgen.1012129.s008.gz › SupplementalDataSet1/03.Result_X202SC24112711-Z01-F001_C_elegans/result/05.InDel_VarDetect/picture_in_reports/WRM102.InDel.table.pdf]

WRM102

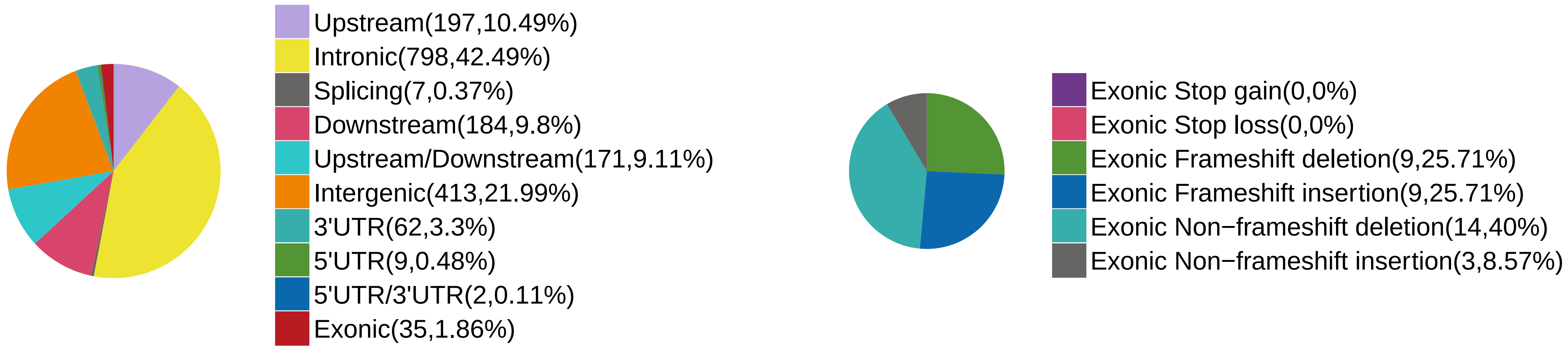
Upstream(197,10.49%)
Intronic(798,42.49%)
Splicing(7,0.37%)
Downstream(184,9.8%)
Upstream/Downstream(171,9.11%)
Intergenic(413,21.99%)
3'UTR(62,3.3%)
5'UTR(9,0.48%)
5'UTR/3'UTR(2,0.11%)
Exonic(35,1.86%)
Exonic Stop gain(0,0%)
Exonic Stop loss(0,0%)
Exonic Frameshift deletion(9,25.71%)
Exonic Frameshift insertion(9,25.71%)
Exonic Non−frameshift deletion(14,40%)
Exonic Non−frameshift insertion(3,8.57%)
