# Supplementary material for: The pos-1 3′ untranslated region governs germline specification and proliferation to ensure reproductive robustness
Source: PLoS Genet. 2026 Apr 27;22(4):e1012129. doi: 10.1371/journal.pgen.1012129 (PMC13132445; doi:10.1371/journal.pgen.1012129)
Supplement: S2 Data — (GZ) [file pgen.1012129.s008.gz › SupplementalDataSet1/03.Result_X202SC24112711-Z01-F001_C_elegans/result/07.CNV_VarDetect/picture_in_reports/DG4222.CNV.table.pdf]

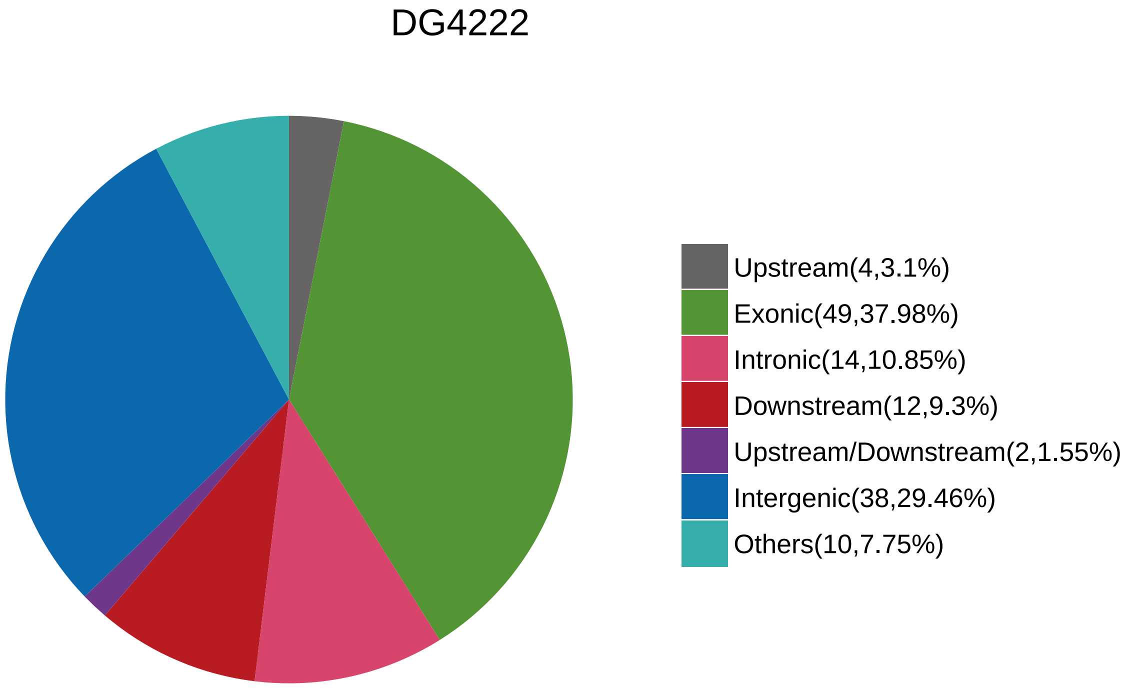

DG4222
Upstream(4,3.1%)
Exonic(49,37.98%)
Intronic(14,10.85%)
Downstream(12,9.3%)
Upstream/Downstream(2,1.55%)
Intergenic(38,29.46%)
Others(10,7.75%)
