# Supplementary material for: The pos-1 3′ untranslated region governs germline specification and proliferation to ensure reproductive robustness
Source: PLoS Genet. 2026 Apr 27;22(4):e1012129. doi: 10.1371/journal.pgen.1012129 (PMC13132445; doi:10.1371/journal.pgen.1012129)
Supplement: S2 Data — (GZ) [file pgen.1012129.s008.gz › SupplementalDataSet1/03.Result_X202SC24112711-Z01-F001_C_elegans/result/07.CNV_VarDetect/picture_in_reports/WRM101.CNV.table.pdf]

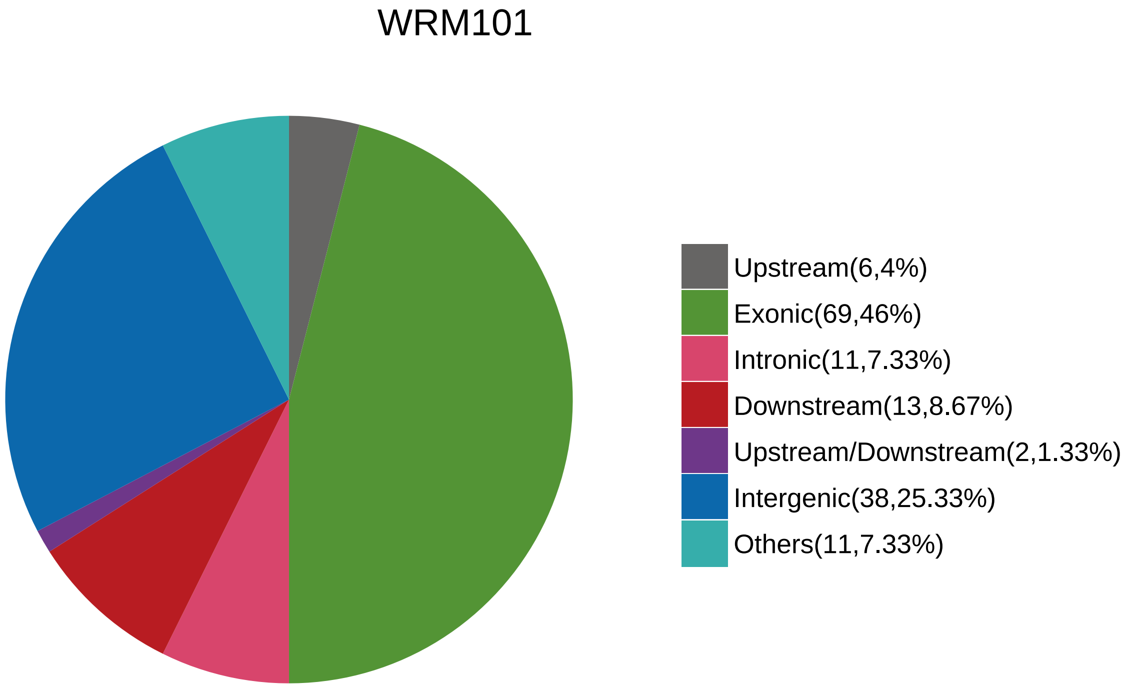
WRM101
Upstream(6,4%)
Exonic(69,46%)
Intronic(11,7.33%)
Downstream(13,8.67%)
Upstream/Downstream(2,1.33%)
Intergenic(38,25.33%)
Others(11,7.33%)
