# Supplementary material for: The pos-1 3′ untranslated region governs germline specification and proliferation to ensure reproductive robustness
Source: PLoS Genet. 2026 Apr 27;22(4):e1012129. doi: 10.1371/journal.pgen.1012129 (PMC13132445; doi:10.1371/journal.pgen.1012129)
Supplement: S2 Data — (GZ) [file pgen.1012129.s008.gz › SupplementalDataSet1/03.Result_X202SC24112711-Z01-F001_C_elegans/result/06.SV_VarDetect/picture_in_reports/SV_length_distribution.pdf]

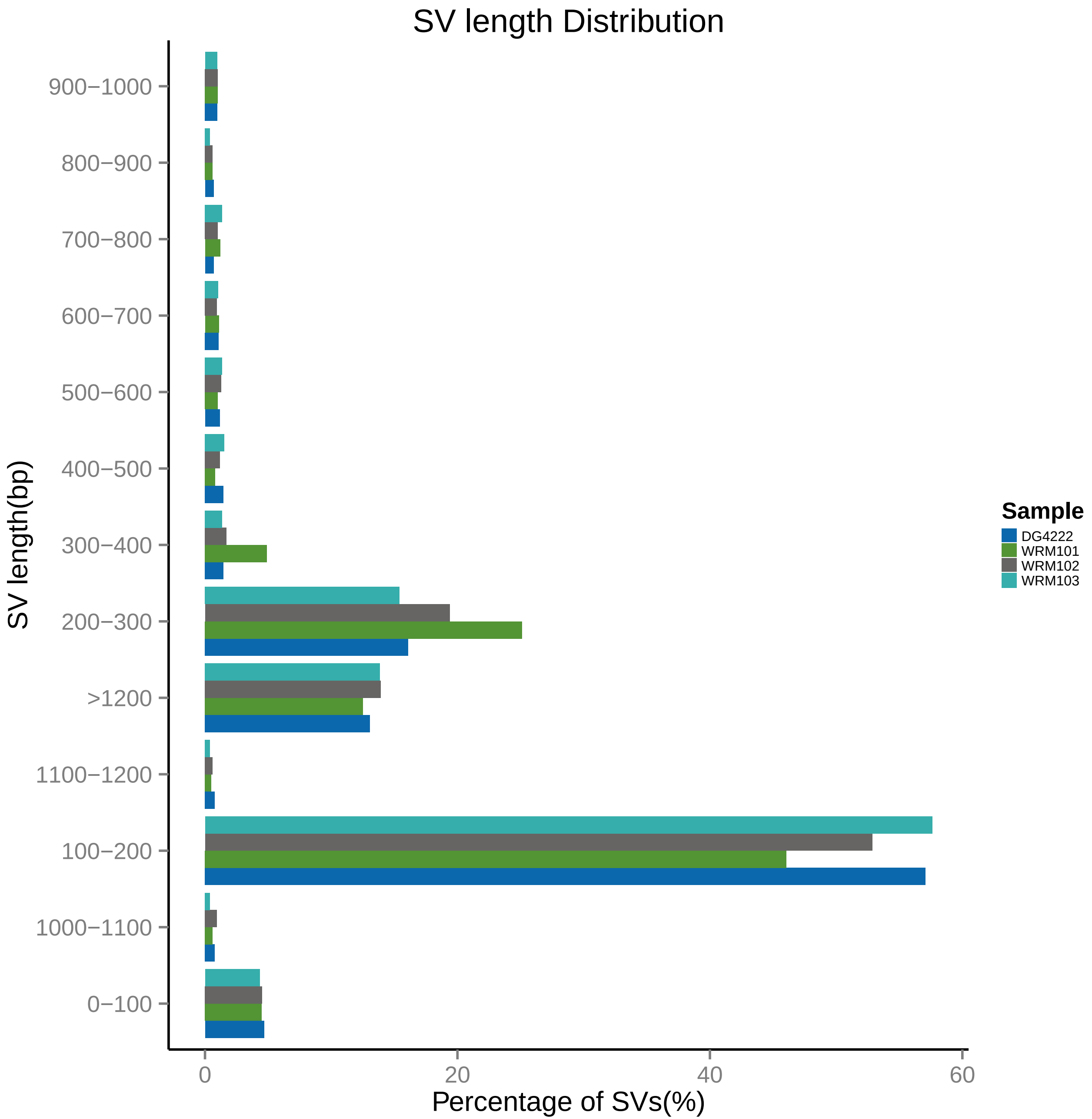

SV length Distribution
SV length(bp)
900−1000
800−900
700−800
600−700
500−600
400−500
300−400
200−300
>1200
1100−1200
100−200
1000−1100
0−100
0
20
40
60
Percentage of SVs(%)
Sample
DG4222
WRM101
WRM102
WRM103
