# Supplementary material for: The pos-1 3′ untranslated region governs germline specification and proliferation to ensure reproductive robustness
Source: PLoS Genet. 2026 Apr 27;22(4):e1012129. doi: 10.1371/journal.pgen.1012129 (PMC13132445; doi:10.1371/journal.pgen.1012129)
Supplement: S2 Data — (GZ) [file pgen.1012129.s008.gz › SupplementalDataSet1/03.Result_X202SC24112711-Z01-F001_C_elegans/result/06.SV_VarDetect/picture_in_reports/DG4222.SV.table.pdf]

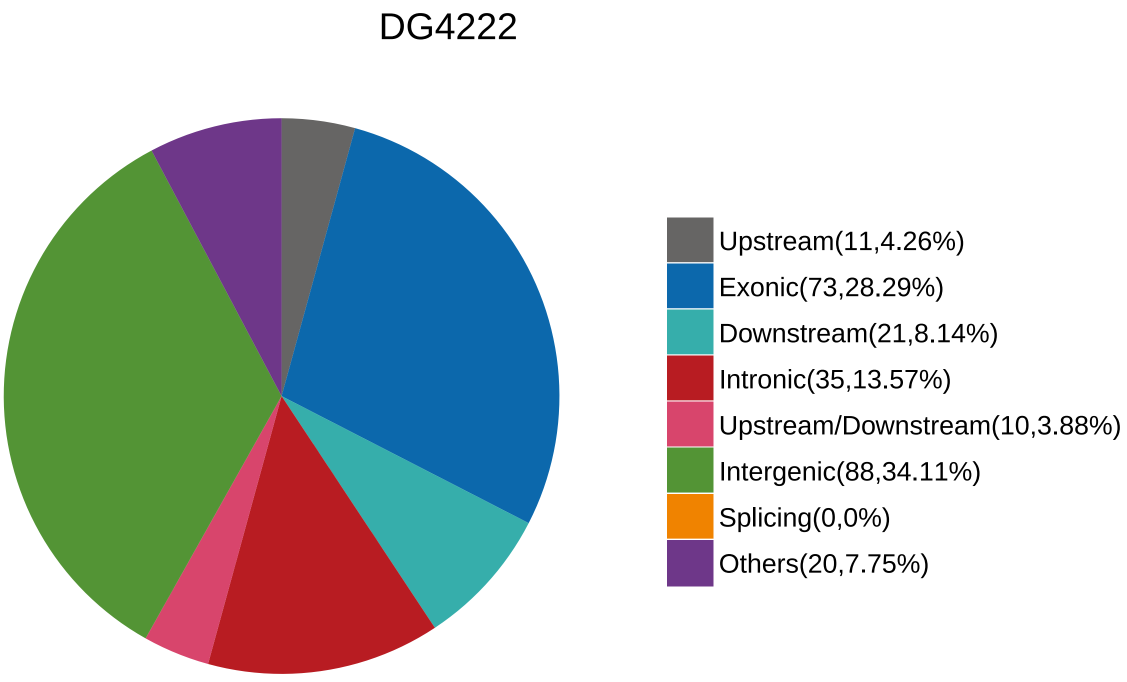
DG4222
Upstream(11,4.26%)
Exonic(73,28.29%)
Downstream(21,8.14%)
Intronic(35,13.57%)
Upstream/Downstream(10,3.88%)
Intergenic(88,34.11%)
Splicing(0,0%)
Others(20,7.75%)
