# Supplementary material for: The pos-1 3′ untranslated region governs germline specification and proliferation to ensure reproductive robustness
Source: PLoS Genet. 2026 Apr 27;22(4):e1012129. doi: 10.1371/journal.pgen.1012129 (PMC13132445; doi:10.1371/journal.pgen.1012129)
Supplement: S2 Data — (GZ) [file pgen.1012129.s008.gz › SupplementalDataSet1/03.Result_X202SC24112711-Z01-F001_C_elegans/result/06.SV_VarDetect/picture_in_reports/WRM101.SV.table.pdf]

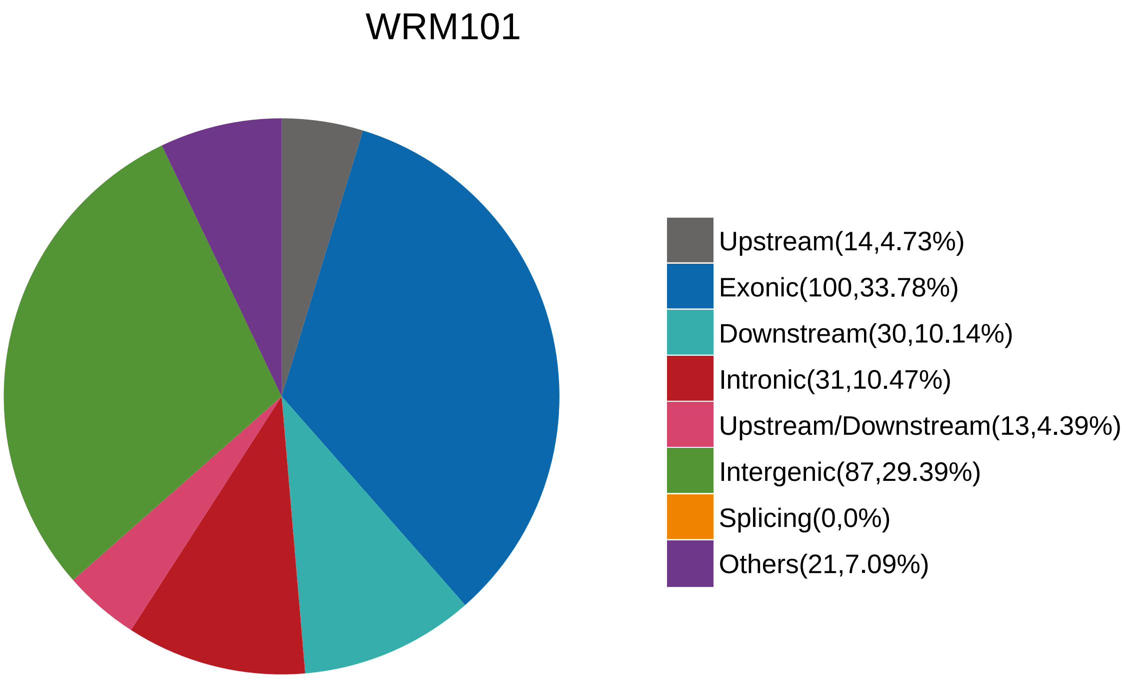
WRM101
Upstream(14,4.73%)
Exonic(100,33.78%)
Downstream(30,10.14%)
Intronic(31,10.47%)
Upstream/Downstream(13,4.39%)
Intergenic(87,29.39%)
Splicing(0,0%)
Others(21,7.09%)
