# Supplementary material for: The pos-1 3′ untranslated region governs germline specification and proliferation to ensure reproductive robustness
Source: PLoS Genet. 2026 Apr 27;22(4):e1012129. doi: 10.1371/journal.pgen.1012129 (PMC13132445; doi:10.1371/journal.pgen.1012129)
Supplement: S2 Data — (GZ) [file pgen.1012129.s008.gz › SupplementalDataSet1/03.Result_X202SC24112711-Z01-F001_C_elegans/result/06.SV_VarDetect/picture_in_reports/SV_ann_Variation_type_statistics_distribution.pdf]

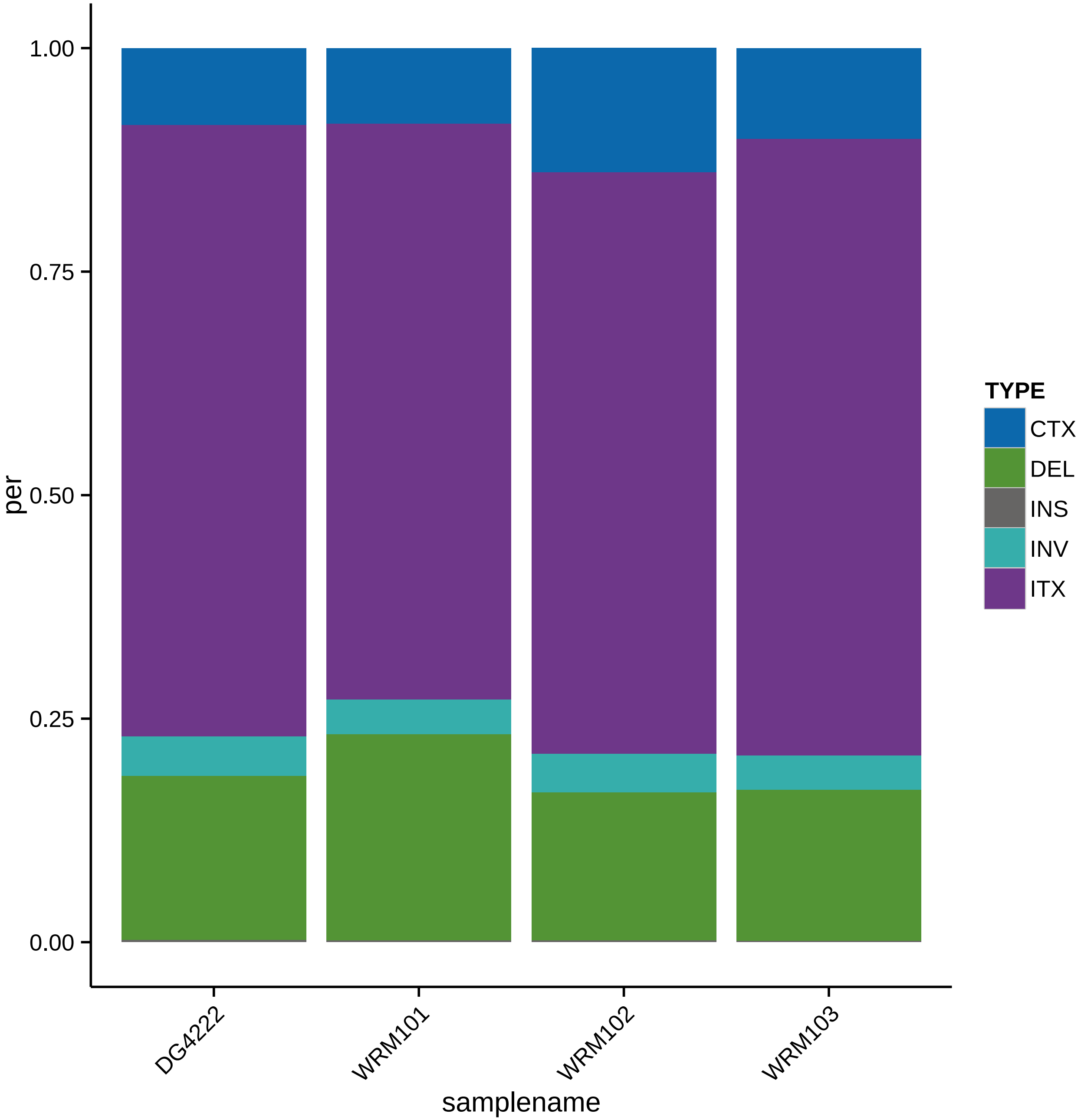
1.00
0.75
0.50
0.25
0.00
per
DG4222
WRM101
WRM102
WRM103
samplename
TYPE
CTX
DEL
INS
INV
ITX
