# Supplementary material for: The pos-1 3′ untranslated region governs germline specification and proliferation to ensure reproductive robustness
Source: PLoS Genet. 2026 Apr 27;22(4):e1012129. doi: 10.1371/journal.pgen.1012129 (PMC13132445; doi:10.1371/journal.pgen.1012129)
Supplement: S2 Data — (GZ) [file pgen.1012129.s008.gz › SupplementalDataSet1/03.Result_X202SC24112711-Z01-F001_C_elegans/result/06.SV_VarDetect/picture_in_reports/WRM102.SV.table.pdf]

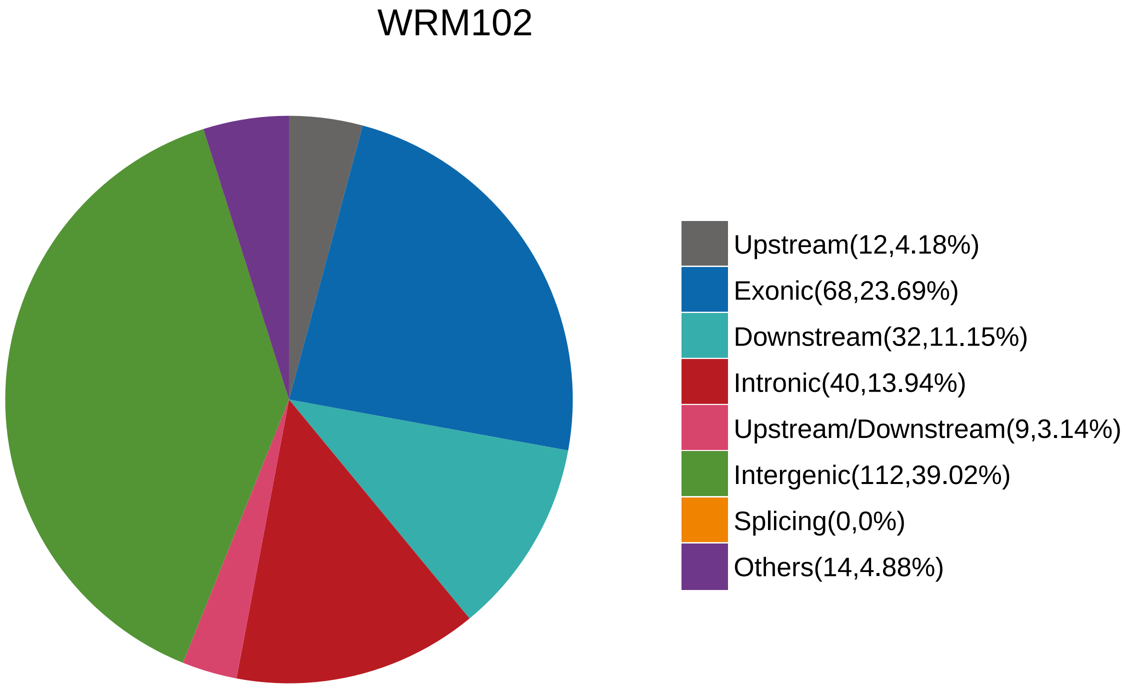
WRM102
Upstream(12,4.18%)
Exonic(68,23.69%)
Downstream(32,11.15%)
Intronic(40,13.94%)
Upstream/Downstream(9,3.14%)
Intergenic(112,39.02%)
Splicing(0,0%)
Others(14,4.88%)
