# Supplementary material for: The pos-1 3′ untranslated region governs germline specification and proliferation to ensure reproductive robustness
Source: PLoS Genet. 2026 Apr 27;22(4):e1012129. doi: 10.1371/journal.pgen.1012129 (PMC13132445; doi:10.1371/journal.pgen.1012129)
Supplement: S2 Data — (GZ) [file pgen.1012129.s008.gz › SupplementalDataSet1/03.Result_X202SC24112711-Z01-F001_C_elegans/result/04.SNP_VarDetect/picture_in_reports/SNP.frequency.pdf]

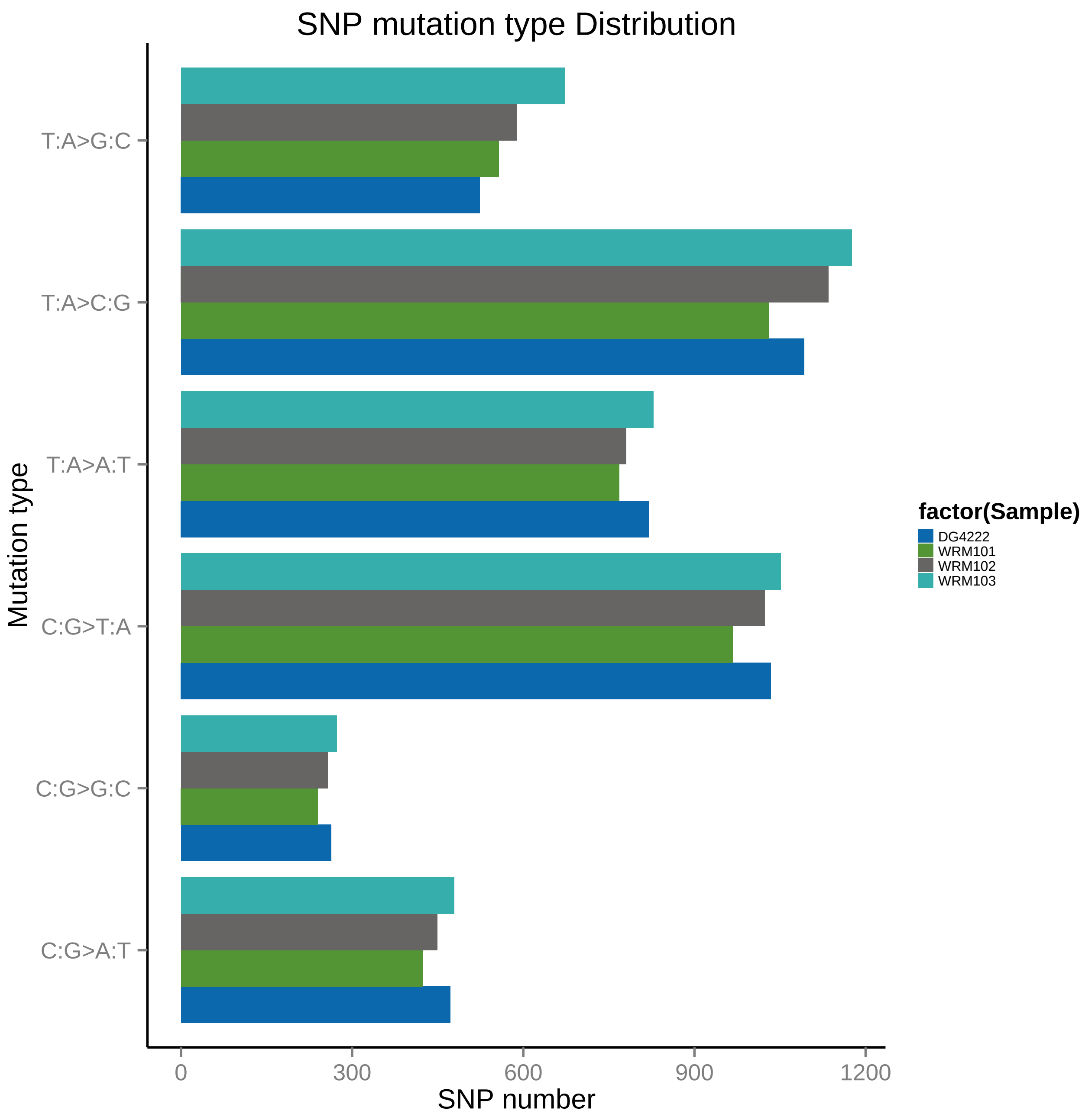
SNP mutation type Distribution
T:A>G:C
T:A>C:G
T:A>A:T
C:G>T:A
C:G>G:C
C:G>A:T
Mutation type
0
300
600
900
1200
SNP number
factor(Sample)
DG4222
WRM101
WRM102
WRM103
