# Supplementary material for: The pos-1 3′ untranslated region governs germline specification and proliferation to ensure reproductive robustness
Source: PLoS Genet. 2026 Apr 27;22(4):e1012129. doi: 10.1371/journal.pgen.1012129 (PMC13132445; doi:10.1371/journal.pgen.1012129)
Supplement: S2 Data — (GZ) [file pgen.1012129.s008.gz › SupplementalDataSet1/03.Result_X202SC24112711-Z01-F001_C_elegans/result/04.SNP_VarDetect/picture_in_reports/SNP.frequency.xls.barplot.pdf]

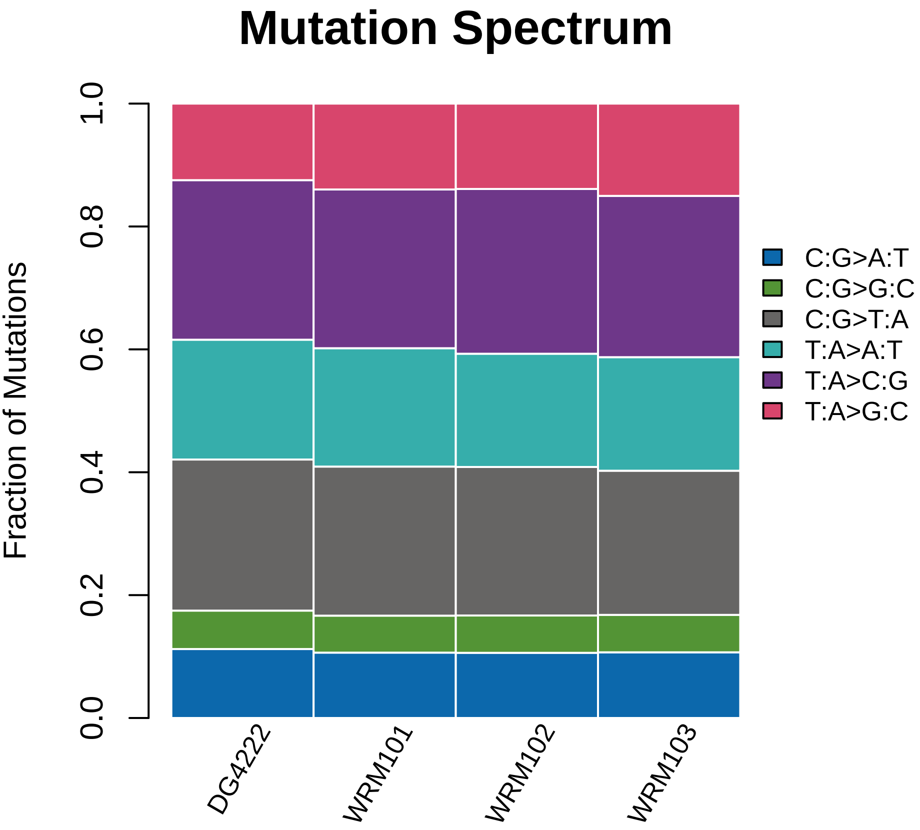

Mutation Spectrum
Fraction of Mutations
0.0
0.2
0.4
0.6
0.8
1.0
DG4222
WRM101
WRM102
WRM103
C:G>A:T
C:G>G:C
C:G>T:A
T:A>A:T
T:A>C:G
T:A>G:C
