# Supplementary material for: The pos-1 3′ untranslated region governs germline specification and proliferation to ensure reproductive robustness
Source: PLoS Genet. 2026 Apr 27;22(4):e1012129. doi: 10.1371/journal.pgen.1012129 (PMC13132445; doi:10.1371/journal.pgen.1012129)
Supplement: S2 Data — (GZ) [file pgen.1012129.s008.gz › SupplementalDataSet1/03.Result_X202SC24112711-Z01-F001_C_elegans/result/04.SNP_VarDetect/picture_in_reports/WRM103.SNP.table.pdf]

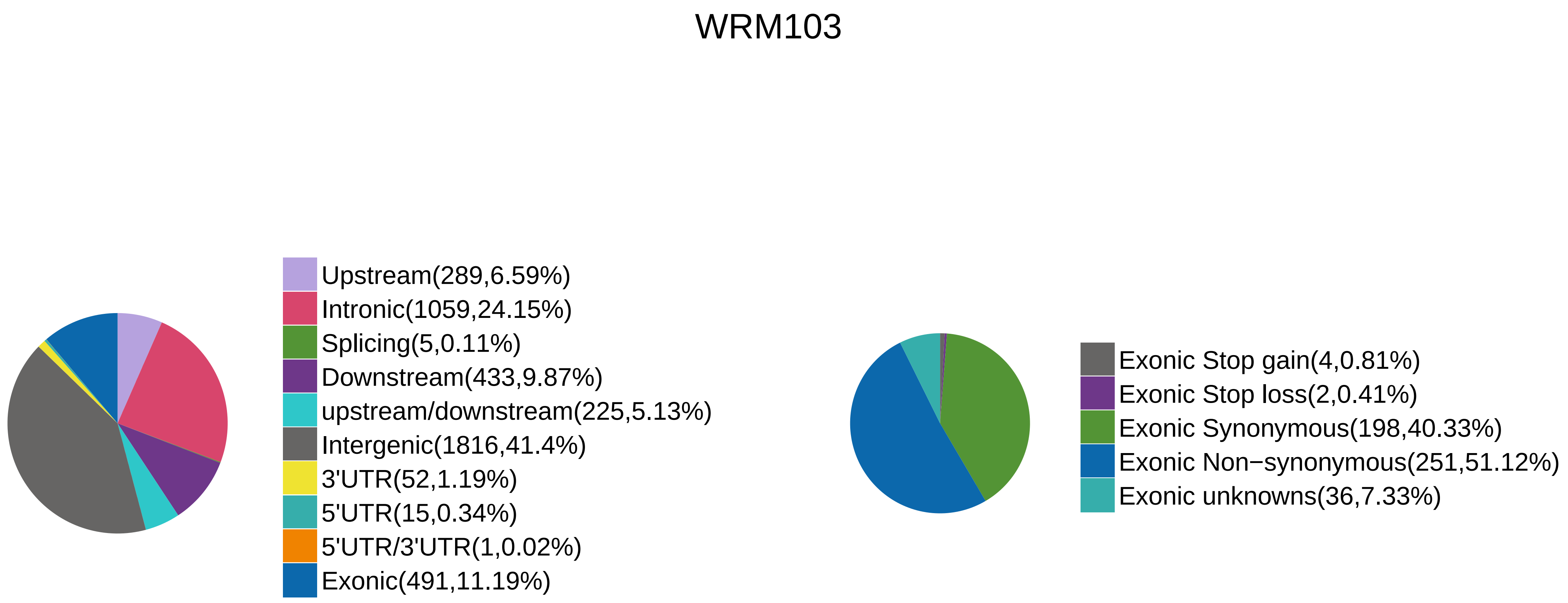
WRM103
Upstream(289,6.59%)
Intronic(1059,24.15%)
Splicing(5,0.11%)
Downstream(433,9.87%)
upstream/downstream(225,5.13%)
Intergenic(1816,41.4%)
3'UTR(52,1.19%)
5'UTR(15,0.34%)
5'UTR/3'UTR(1,0.02%)
Exonic(491,11.19%)
Exonic Stop gain(4,0.81%)
Exonic Stop loss(2,0.41%)
Exonic Synonymous(198,40.33%)
Exonic Non−synonymous(251,51.12%)
Exonic unknowns(36,7.33%)
