# Supplementary material for: The pos-1 3′ untranslated region governs germline specification and proliferation to ensure reproductive robustness
Source: PLoS Genet. 2026 Apr 27;22(4):e1012129. doi: 10.1371/journal.pgen.1012129 (PMC13132445; doi:10.1371/journal.pgen.1012129)
Supplement: S2 Data — (GZ) [file pgen.1012129.s008.gz › SupplementalDataSet1/03.Result_X202SC24112711-Z01-F001_C_elegans/result/04.SNP_VarDetect/picture_in_reports/DG4222.SNP.table.pdf]

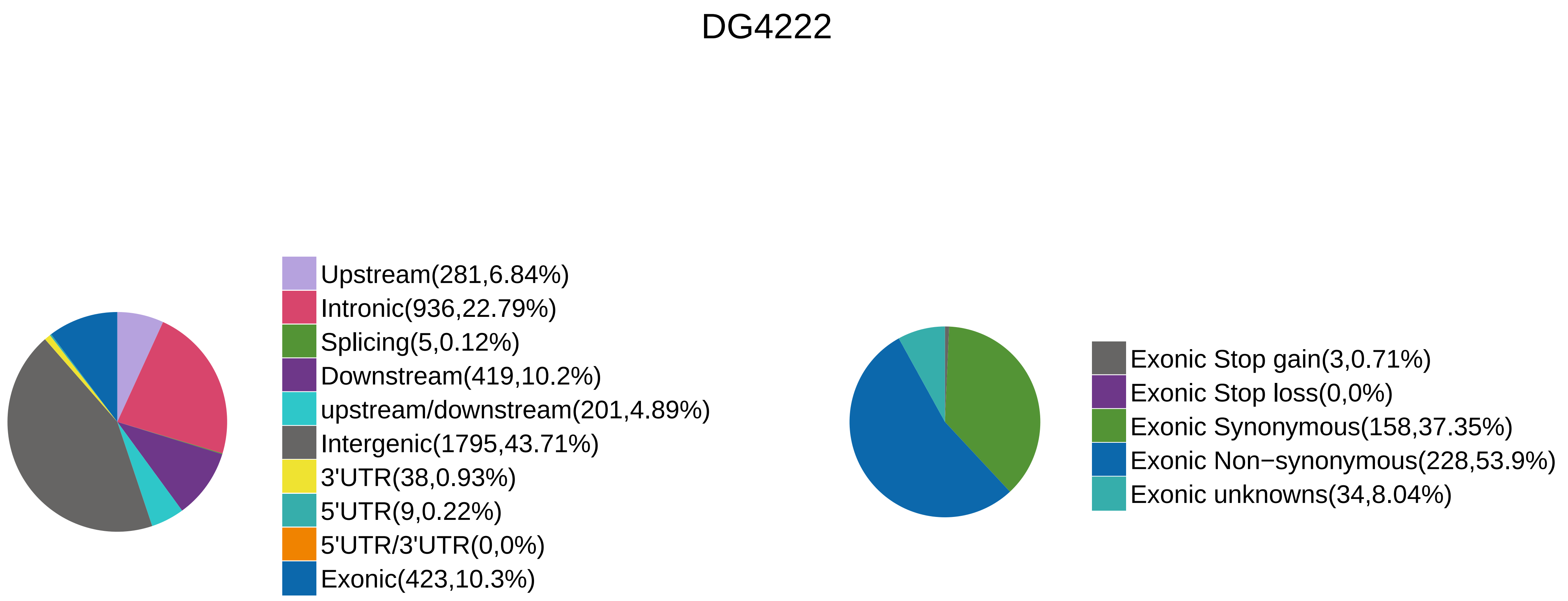
DG4222
Upstream(281,6.84%)
Intronic(936,22.79%)
Splicing(5,0.12%)
Downstream(419,10.2%)
upstream/downstream(201,4.89%)
Intergenic(1795,43.71%)
3'UTR(38,0.93%)
5'UTR(9,0.22%)
5'UTR/3'UTR(0,0%)
Exonic(423,10.3%)
Exonic Stop gain(3,0.71%)
Exonic Stop loss(0,0%)
Exonic Synonymous(158,37.35%)
Exonic Non−synonymous(228,53.9%)
Exonic unknowns(34,8.04%)
