# Supplementary material for: The pos-1 3′ untranslated region governs germline specification and proliferation to ensure reproductive robustness
Source: PLoS Genet. 2026 Apr 27;22(4):e1012129. doi: 10.1371/journal.pgen.1012129 (PMC13132445; doi:10.1371/journal.pgen.1012129)
Supplement: S2 Data — (GZ) [file pgen.1012129.s008.gz › SupplementalDataSet1/03.Result_X202SC24112711-Z01-F001_C_elegans/result/04.SNP_VarDetect/picture_in_reports/WRM102.SNP.table.pdf]

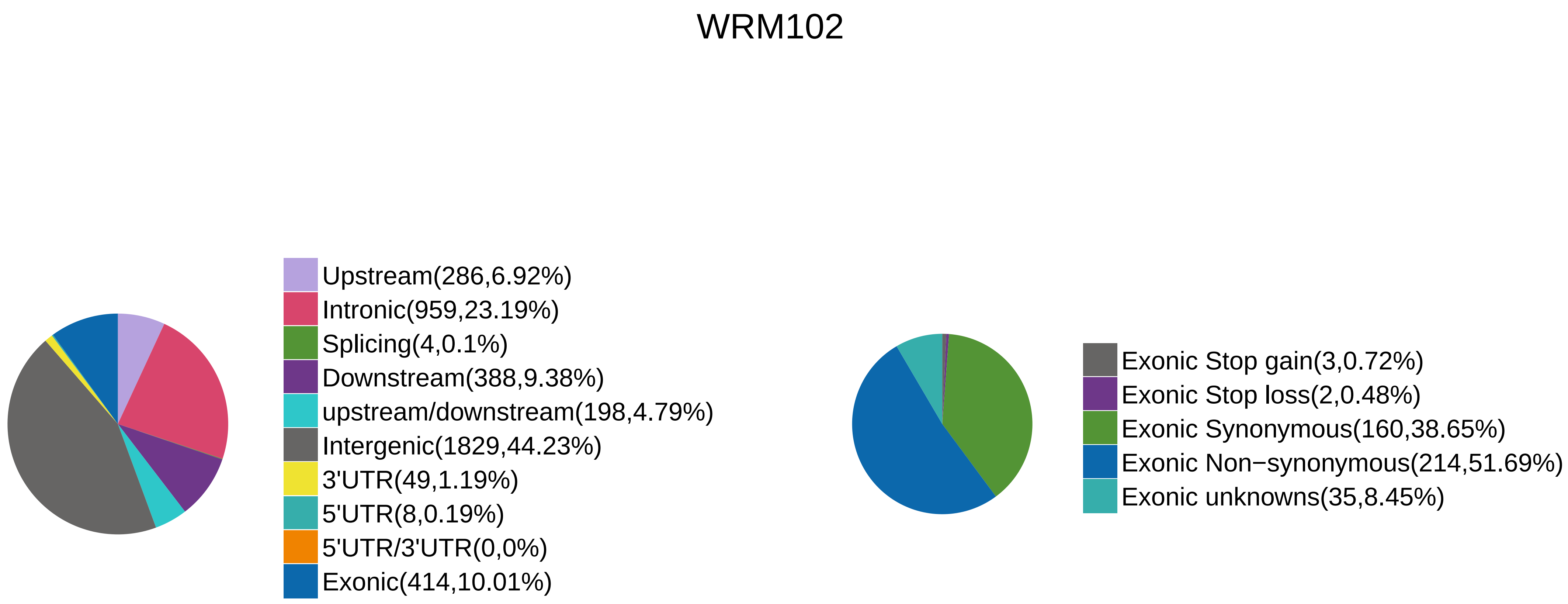
WRM102
Upstream(286,6.92%)
Intronic(959,23.19%)
Splicing(4,0.1%)
Downstream(388,9.38%)
upstream/downstream(198,4.79%)
Intergenic(1829,44.23%)
3'UTR(49,1.19%)
5'UTR(8,0.19%)
5'UTR/3'UTR(0,0%)
Exonic(414,10.01%)
Exonic Stop gain(3,0.72%)
Exonic Stop loss(2,0.48%)
Exonic Synonymous(160,38.65%)
Exonic Non−synonymous(214,51.69%)
Exonic unknowns(35,8.45%)
