# Supplementary material for: The pos-1 3′ untranslated region governs germline specification and proliferation to ensure reproductive robustness
Source: PLoS Genet. 2026 Apr 27;22(4):e1012129. doi: 10.1371/journal.pgen.1012129 (PMC13132445; doi:10.1371/journal.pgen.1012129)
Supplement: S2 Data — (GZ) [file pgen.1012129.s008.gz › SupplementalDataSet1/03.Result_X202SC24112711-Z01-F001_C_elegans/result/04.SNP_VarDetect/picture_in_reports/WRM101.SNP.table.pdf]

WRM101

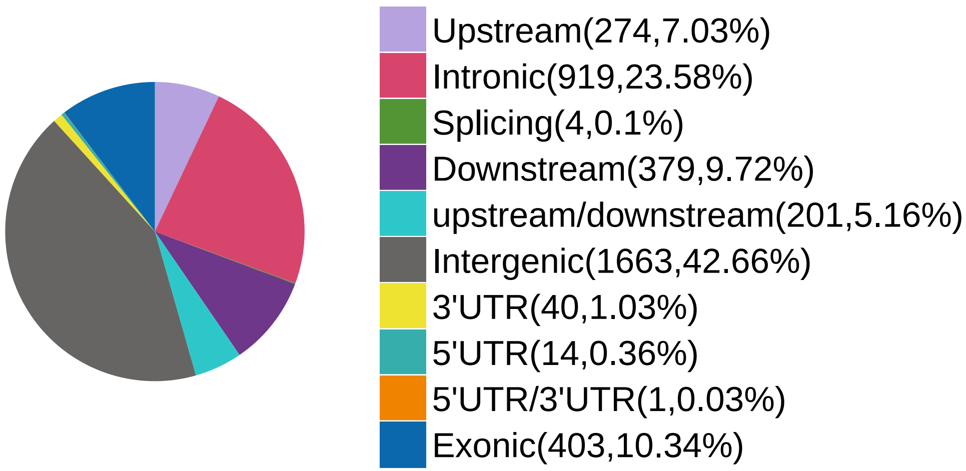
Upstream(274,7.03%)
Intronic(919,23.58%)
Splicing(4,0.1%)
Downstream(379,9.72%)
upstream/downstream(201,5.16%)
Intergenic(1663,42.66%)
3'UTR(40,1.03%)
5'UTR(14,0.36%)
5'UTR/3'UTR(1,0.03%)
Exonic(403,10.34%)

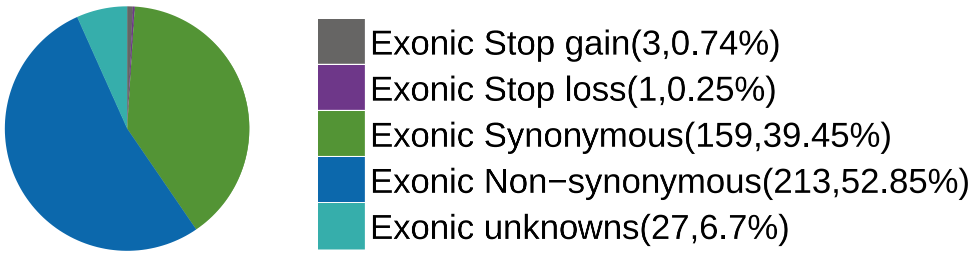
Exonic Stop gain(3,0.74%)
Exonic Stop loss(1,0.25%)
Exonic Synonymous(159,39.45%)
Exonic Non−synonymous(213,52.85%)
Exonic unknowns(27,6.7%)
