# Supplementary material for: The pos-1 3′ untranslated region governs germline specification and proliferation to ensure reproductive robustness
Source: PLoS Genet. 2026 Apr 27;22(4):e1012129. doi: 10.1371/journal.pgen.1012129 (PMC13132445; doi:10.1371/journal.pgen.1012129)
Supplement: S2 Data — (GZ) [file pgen.1012129.s008.gz › SupplementalDataSet1/03.Result_X202SC24112711-Z01-F001_C_elegans/result/04.SNP_VarDetect/picture_in_reports/SNP_distance_cumulative_distribution.pdf]

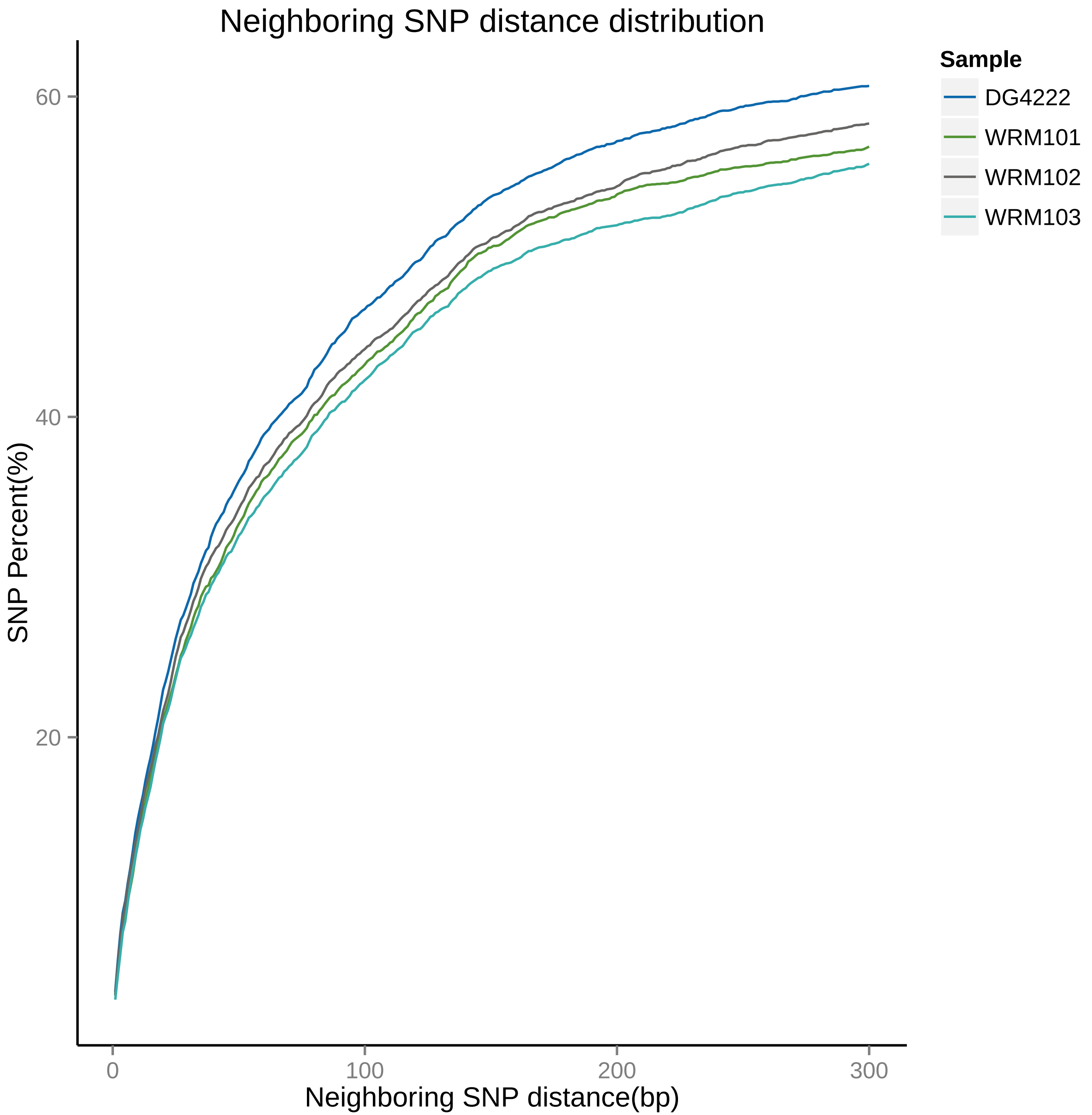

Neighboring SNP distance distribution
SNP Percent(%)
Neighboring SNP distance(bp)
60
40
20
0
100
200
300
Sample
DG4222
WRM101
WRM102
WRM103
