# Supplementary material for: The pos-1 3′ untranslated region governs germline specification and proliferation to ensure reproductive robustness
Source: PLoS Genet. 2026 Apr 27;22(4):e1012129. doi: 10.1371/journal.pgen.1012129 (PMC13132445; doi:10.1371/journal.pgen.1012129)

Classification of Raw Reads
(WRM102_CKDN240021611−1A_22HVGTLT4_L1)

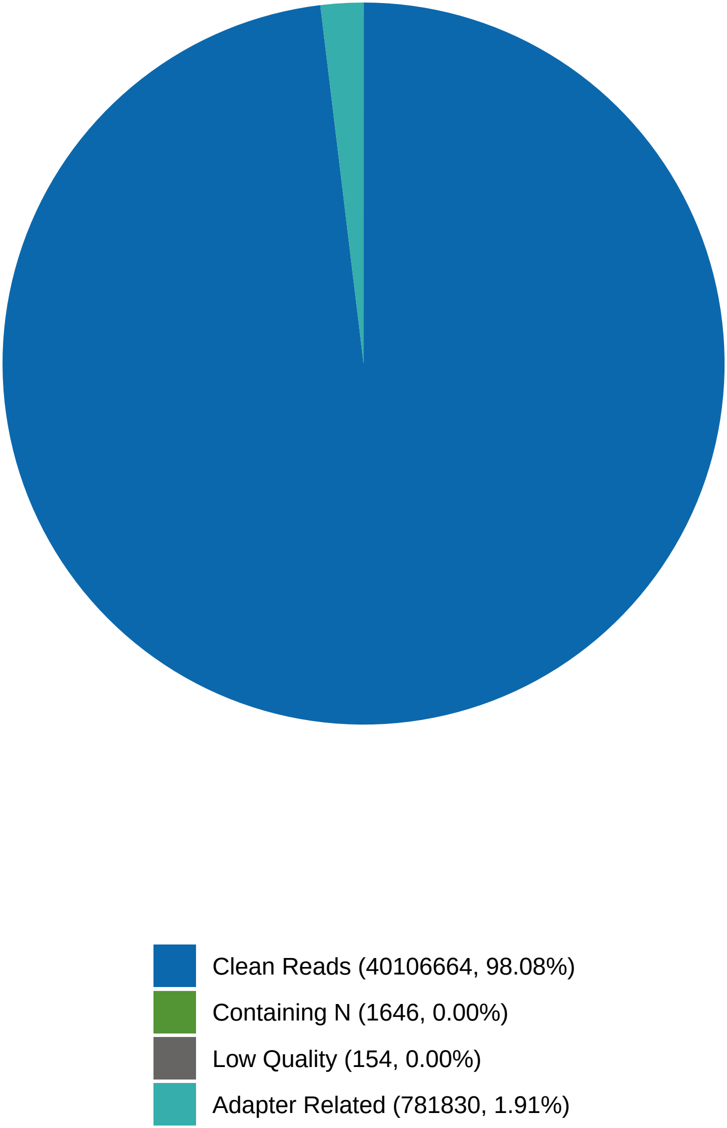

Supplement: S2 Data — (GZ) [file pgen.1012129.s008.gz › SupplementalDataSet1/03.Result_X202SC24112711-Z01-F001_C_elegans/result/02.QualityControl/ReadsClassification/WRM102_CKDN240021611-1A_22HVGTLT4_L1.pie3d.pdf]
