# Supplementary material for: The pos-1 3′ untranslated region governs germline specification and proliferation to ensure reproductive robustness
Source: PLoS Genet. 2026 Apr 27;22(4):e1012129. doi: 10.1371/journal.pgen.1012129 (PMC13132445; doi:10.1371/journal.pgen.1012129)
Supplement: S2 Data — (GZ) [file pgen.1012129.s008.gz › SupplementalDataSet1/03.Result_X202SC24112711-Z01-F001_C_elegans/result/02.QualityControl/ErrorRate/WRM101_CKDN240021610-1A_22HTVFLT4_L1.Error.pdf]

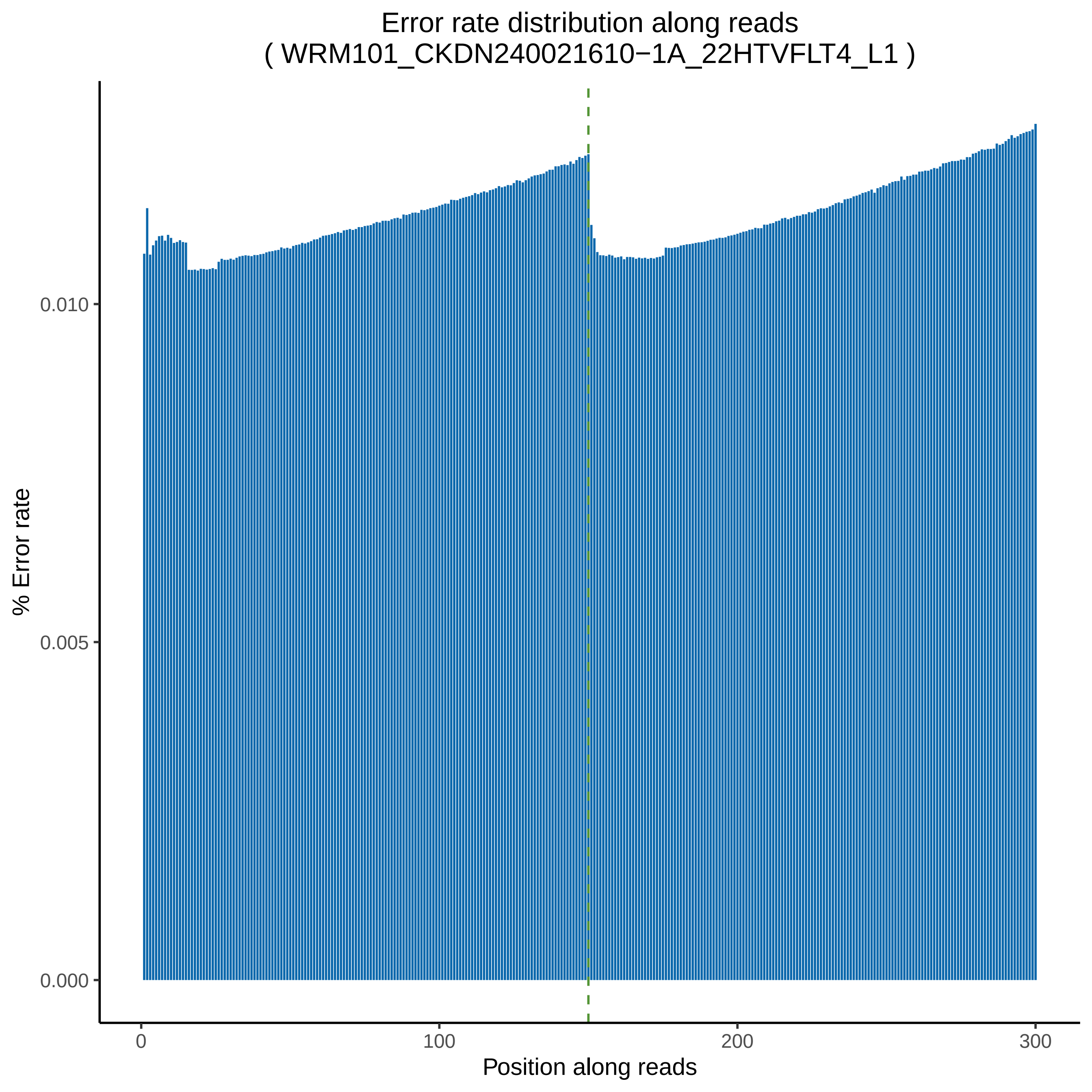
Error rate distribution along reads
(WRM101_CKDN240021610−1A_22HTVFLT4_L1)
% Error rate
0.010
0.005
0.000
0
100
200
300
Position along reads
